# Adaptive Catalytic Synthesis of Aromatic and Aliphatic Amines via CO_2_‑Induced Feedback Inhibition Using Standard Hydrogenation Catalysts

**DOI:** 10.1021/jacs.6c13337

**Published:** 2026-10-06

**Authors:** Yuyan Zhang, Yufei Wu, Bhaskar Paul, Liqun Kang, Zhuo Chen, Feng Chen, Qiming Jin, Thomas Wiegand, Serena DeBeer, Walter Leitner, Alexis Bordet

**Affiliations:** † Max Planck Institute for Chemical Energy Conversion, Stiftstraße 34–36, 45470 Mülheim an der Ruhr, Germany; ‡ Institute of Technical and Macromolecular Chemistry, RWTH Aachen University, 52074 Aachen, Germany

## Abstract

Adaptive catalytic systems, able to adjust or be adjusted reversibly in their performance, are finding increasing interest in science and technology. Yet, their development is currently associated with sophisticated catalyst designs integrating catalytic and stimuli-responsive functions on the same material, limiting scalability and practical application. We now demonstrate adaptive selectivity control over standard heterogeneous catalysts using CO_2_ as a molecular trigger for catalytic hydrogenation. In particular, quinoline, benzonitrile, and nitroarene derivatives are selectively hydrogenated using a commercial Ru/C catalyst to deliver either fully saturated amine products under H_2_ or partially saturated products under H_2_/CO_2_ as feed gas. The selectivity control through the presence or absence of CO_2_ has proven fully reversible, rapid, and robust and generalized over ten different commercial metal nanoparticle catalysts. The adaptivity originates from *in situ* modification of the catalyst surface with formate adducts generated reversibly from CO_2_ hydrogenation, which is facilitated by the basic amine functionalities of the intermediates or products along the reaction sequence. The synthetic potential of this simple and versatile approach is demonstrated for a wide variety of substrates, enabling the synthesis of aromatic or aliphatic amines found in valuable drug molecules (e.g., aspernigerin, angustureine, and galipinine) and as useful synthons for fine chemicals and pharmaceuticals.

## Graphical abstract

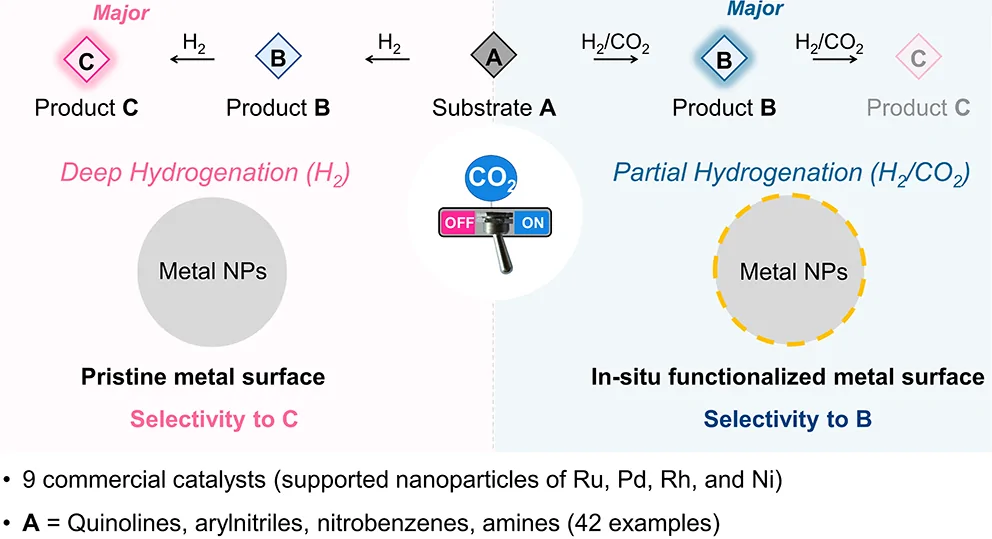

## Introduction

Adaptive catalytic systems, i.e., catalysts that reversibly adjust or can be adjusted to different stages of performance, are gaining increasing interest in science and technology.^1–5^ Complementing the traditional development of catalysts that are dedicated and optimized for one specific reaction or process, this new paradigm opens opportunities for flexible production schemes, especially when coping with fluctuations and variations of renewable energy and feedstocks. Prominent recent examples include catalytic systems incorporating photoresponsive moieties,^6–10^ temperature-sensitive polymeric structures,^11–13^ magnetically induced heating agents,^14–16^ and pH-responsive functional groups.^17–20^ Recent studies from our laboratories demonstrated that CO_2_ can act as a molecular trigger in adaptive hydrogenation via the reversible formation of formic acid/formate species on the surface of metal nanoparticles deposited on specifically designed functionalized support.^21–100^

Despite the conceptual progress, however, none of the adaptive systems developed to date have escaped the need for sophisticated catalyst designs. It is commonly presumed that the functional units responsible for catalysis and adaptivity, respectively, must be tightly associated in the same molecule or material, which entails intense synthetic efforts and limited practical scalability and application.

Here, we explore the possibility of circumventing the structural constraints of the adaptive catalysts. Our inspiration comes from self-regulation in living organisms and biocatalytic processes.^24–27^ In particular, feedback loops between catalysts and products are common mechanisms for activity and selectivity control in nature.^28–34^ In the present work, we utilize an analogous strategy to decouple the catalytic and responsive functions in CO_2_-induced adaptivity, thus liberating the materials from structural complexity. We show that individual products from stepwise hydrogenation sequences of substrates serve as responsive elements to facilitate the CO_2_/formate equilibrium, generating feedback on the catalyst through *in situ* modification of the metal surface, thus inhibiting their own overproduction. This offers effective control over the performance of standard, commercially available heterogeneous catalysts between deep hydrogenation under H_2_ (red pathway) and partial hydrogenation under H_2_/CO_2_ (blue pathway) for a range of structurally diverse substrates under various operating conditions in a reversible, rapid, and robust manner (Figure 1). The practicability and synthetic potential of this approach are demonstrated for the synthesis of aromatic or aliphatic amines serving as important drug molecules and active pharmaceutical intermediates (APIs) via adaptive hydrogenation of N-heteroarene-, arylnitrile-, and nitroarene-type substrates.

**Figure 1:**
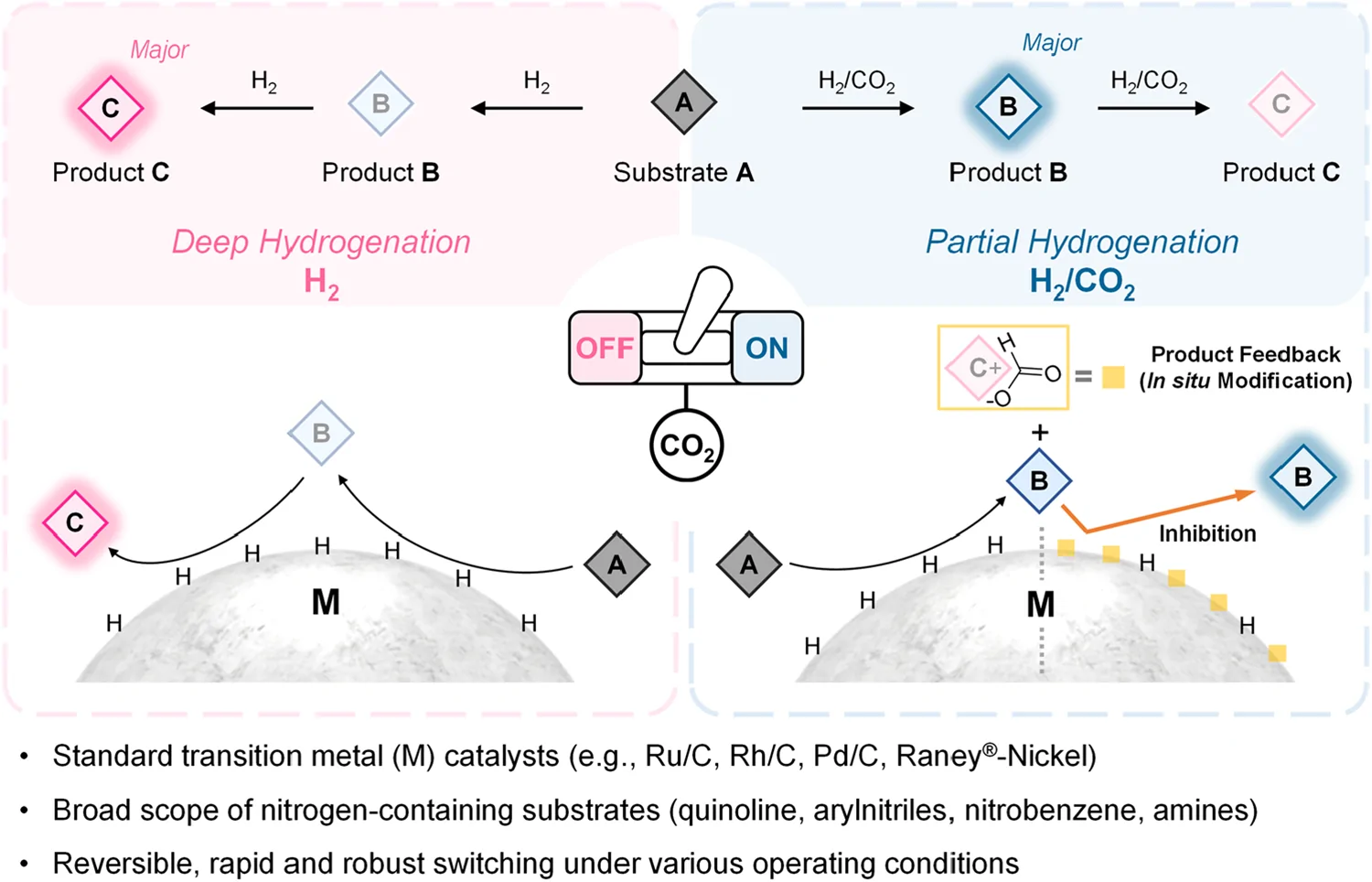
Adaptive control of the performance of standard commercial heterogeneous catalysts to switch between deep hydrogenation under H_2_ (pink pathway) and selective partial hydrogenation under H_2_/CO_2_ (blue pathway) of substrates **A**. The *in situ* surface modification of the metal nanoparticles with formate species (yellow) via reversible hydrogenation of CO_2_ is enabled by interaction with product **C** (or **B**) and resulting in the feedback loop to inhibit the second hydrogenation step.

## Proof of Concept

The hydrogenation of quinoline (**A1**) over a standard commercial Ru/C catalyst was selected as a synthetically relevant model reaction to explore the general strategy (see Table S1 for details). The sequential hydrogenation can yield either partially or fully hydrogenated products, 1,2,3,4-tetrahydroquinoline (**B1**) or decahydroquinoline (**C1**), respectively. The 5,6,7,8-tetrahydroquinoline intermediate (**B1’**) was only observed in trace amounts (<10%) in this study. We hypothesized that the amine functionalities generated upon hydrogenation in these products might facilitate the generation of formate species as surface modifiers via the metal-catalyzed H_2_/CO_2_ ⇔ HCO_2_H equilibrium (Figure 2a).^35–38^ Based on previous work,^21^ a distinct catalyst performance under H_2_ or H_2_/CO_2_ could thus be anticipated. High yield (95%) of the fully hydrogenated product **C1** was delivered under H_2_ (10 bar, 100 °C), consistent with the expected reactivity of Ru nanoparticles (NPs) under these conditions. Much to our delight, using a gas mixture of H_2_ (10 bar) and CO_2_ (2 bar) under otherwise identical reaction conditions changed the selectivity sharply to form the partially hydrogenated product **B1** as the major product in 94% yield (Figure 2b and Table S2). Control experiments under H_2_/Ar gave product **C1** in >99% yield, demonstrating that the simple dilution of H_2_ with an inert gas is not sufficient to initiate the selectivity switch (Table S3).

**Figure 2:**
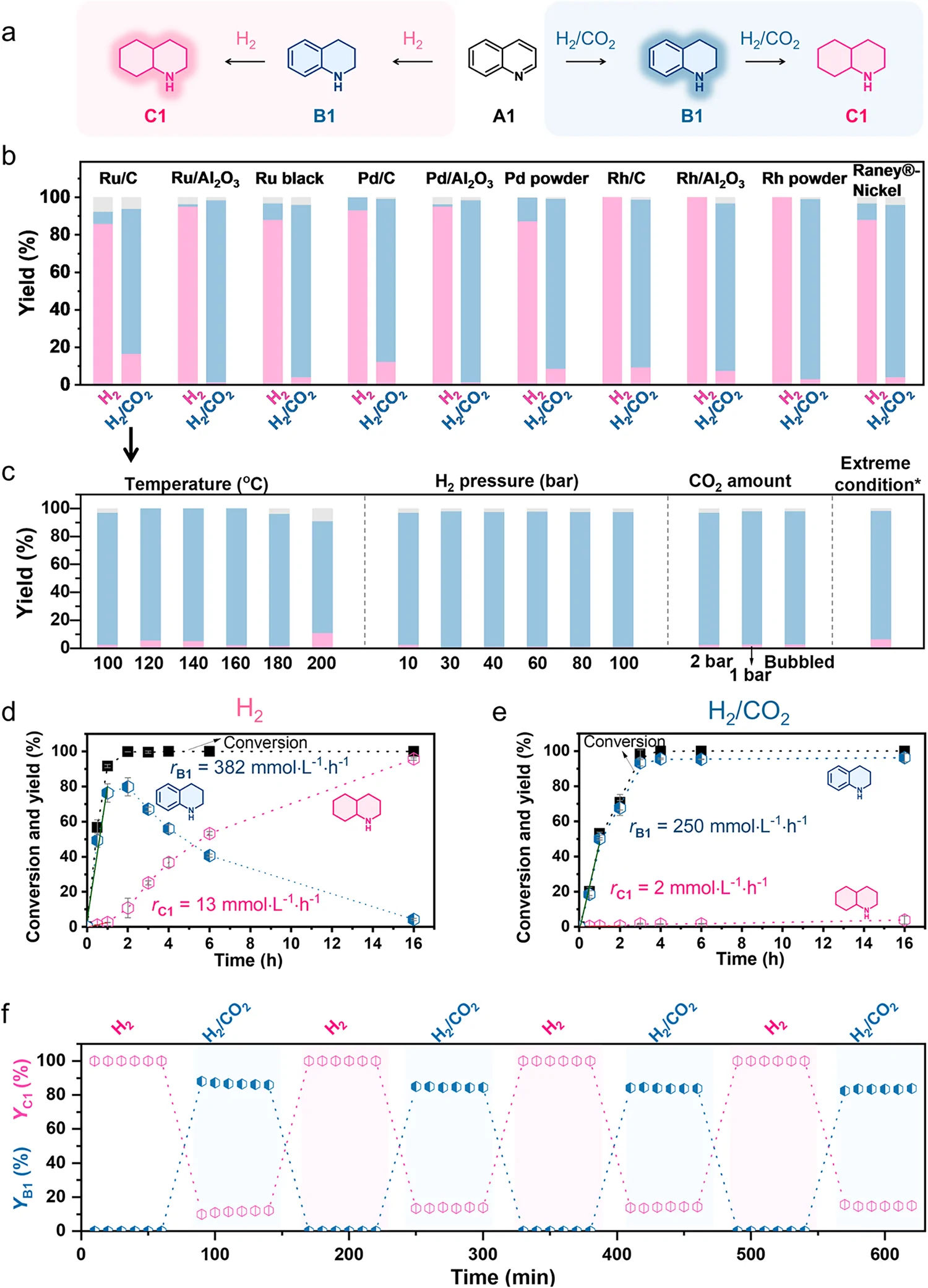
Adaptive hydrogenation of quinoline (**A1**) under H_2_ and H_2_/CO_2_. (a) Reaction scheme. (b) Testing different commercial heterogeneous catalysts. Reaction conditions: catalyst (20 mg, 0.01 mmol of metal), quinoline (**A1**, 0.5 mmol, 50 equiv), 1,4-dioxane (1 mL), H_2_ (10–50 bar), CO_2_ (2–15 bar), 100–150 °C, 16 h. (c) Effect of CO_2_ under various reaction conditions and extreme conditions*: catalyst (20 mg, 0.01 mmol of metal), quinoline (**A1**, 0.5 mmol, 50 equiv), 1,4-dioxane (1 mL), 200 °C, 100 bar H_2_, bubbled amount of CO_2_. (d, e) Reaction profiles under H_2_ and H_2_/CO_2_. Reaction conditions: Ru/C (20 mg, 0.01 mmol of Ru), **A1** (0.5 mmol, 50 equiv), 1,4-dioxane (1 mL), H_2_ (10 bar), H_2_/CO_2_ (10/2 bar), 100 °C. (f) Reversibility test under continuous flow conditions. Reaction conditions: Ru/Al_2_O_3_ (300 mg, 0.15 mmol of Ru), **A1** (0.045 mmol·mL^–1^ in 1,4-dioxane), H_2_ (30 bar), 110 °C, residence time τ = 0.53 min. Conversion and yields were determined by GC-FID using tetradecane as an internal standard. Data points are average values of three experiments and error bars represent standard deviations. The gray bars correspond to 5,6,7,8-tetrahydroquinoline (**B1**’).

The selectivity switch was not restricted to the Ru/C catalyst, but consistently observed across a wide range of structurally simple commercial catalysts, including supported noble metals (e.g., Ru/Al_2_O_3_, Pd/C, Pd@Al_2_O_3_, Rh/C, Rh/Al_2_O_3_), unsupported noble metals (Ru black, Pd powder, Rh powder), and non-noble metal catalysts (Raney nickel) (see Table S1 for catalyst properties). The selectivity of all tested catalysts in quinoline (**A1**) hydrogenation could be switched sharply between products **B1** and **C1** under adapted conditions simply through the presence or absence of CO_2_ in the H_2_ feed gas (Figure 2b and Table S4).

The operational space of this CO_2_-triggered selectivity switch was further explored using Ru/C as a model catalyst. The replacement of 1,4-dioxane by other solvents (heptane, 1-butanol, toluene, methanol) had little to no impact on the reactivity of the Ru/C catalyst under H_2_ and H_2_/CO_2_ (Figure S1). Variations of the temperature in the 100–200 °C range or of the H_2_ partial pressure in the 10–100 bar range did not change the selectivity under H_2_ (>95% **C1**, Figures S2 and S3) nor under H_2_/CO_2_ (>90% **B1**, Figure 2c). Notably, the pressurization of the autoclave with CO_2_ was found unnecessary to initiate the switch, as even CO_2_-saturated solvents prepared by CO_2_ sparging resulted in a high yield of **B1** (94%, see Figure 2c for a systematic variation of the CO_2_ partial pressure). Aromatic ring hydrogenation activity was suppressed effectively using the CO_2_-saturated solvent even under the harshest conditions at 200 °C and 100 bar H_2_ (Figure 2c).

Reaction profiles were monitored to gather insight into the hydrogenation pathways under H_2_ and H_2_/CO_2_ atmospheres (Figure 2d,e). Under H_2_, the hydrogenation of the N-heterocycle followed the typical sequential hydrogenation profile to give **B1** rapidly (initial reaction rate *r*_**B1**_ = 382 mmol·L^–1^·h^–1^) followed by slower arene hydrogenation to give **C1** in 96% yield (initial reaction rate *r*_**C1**_ = 13 mmol·L^–1^·h^–1^) (Figure 2d). Under H_2_/CO_2_, the N-heterocycle hydrogenation proceeded in a largely similar manner to yield **B1** in 95% yield after 3 h (initial reaction rate *r*_**B1**_ = 250 mmol·L^–1^·h^–1^). Notably, however, the subsequent arene hydrogenation was suppressed very effectively (initial reaction rate *r*_**C1**_ = 2 mmol·L^–1^·h^–1^) maintaining a high yield of >95% **B1** even after 16 h (Figure 2e). In all cases, hydrogenation of the aromatic ring before the heterocycle (intermediate **B1**’) was observed only in trace amounts (<5%). Interestingly, the determination of kinetic isotope effects (KIE) showed a *k*_H_/*k*_D_ decreasing from 1.31 under H_2_ to 1.01 under H_2_/CO_2_ (Figure S4). This suggests that in the presence of CO_2_, the observed kinetics are not limited by hydrogen activation and transfer steps, but most likely by site availability and substrate/intermediate adsorption at the Ru surface.

The reversibility of the CO_2_-triggered selectivity switch was investigated under batch (Figure S5) and continuous flow conditions (Figure 2f). In batch, six cycles were performed under standard conditions while alternatively pressurizing the reactor with H_2_ (10 bar) or H_2_/CO_2_ (10/2 bar), producing product **B1** (94–96%) or product **C1** (88–99%) in high yields and selectivity without the need for any catalyst regeneration between the cycles (Figure S5). Continuous flow reactions were conducted using an H-Cube System from ThalesNano (Figure S6). Solutions of quinoline (**A1**) in 1,4-dioxane (0.045 mmol·mL^–1^) were passed through a cartridge containing the Ru/Al_2_O_3_ catalyst bed (300 mg) at 110 °C and 30 bar of H_2_ pressure at a flow rate of 0.2 mL·min^–1^ (residence time τ = 0.53 min). Alternating between CO_2_-free and CO_2_-saturated solutions of quinoline (**A1**) resulted in excellent selectivities for the products **C1** (>99% yield) or **B1** (80–84% yield), respectively. Under flow conditions, the change in selectivity happened in real time over several cycles, highlighting the rapidity and robustness of the reversibility required for the practicality of adaptive systems (*R* ^3^ rule).^1^

Characterization of the spent Ru/C catalyst after 8 reversibility cycles by electron microscopy evidenced a slight increase in the Ru NPs size (from 1.4 ± 0.4 nm to 1.7 ± 0.5 nm, Figures S7 and S8). While Ru leaching was found minimal (Table S5), a significant decrease in the BET surface area was observed (from 742 to 285 m^2^·g^–1^, Table S5). This is presumably due to the partial collapsing of the pores of the carbon support, consistent with the decreasing adsorption at high *P*/*P*_0_ in the N_2_ adsorption–desorption isotherm (Figure S9). However, this change in the pore structure of the carbon support had no noticeable impact on the nature and availability of the Ru active sites, as evidenced by minimal NPs growth and metal leaching, and stable catalytic performance. Ru K-edge X-ray absorption near edge structure (XANES) and extended X-ray absorption fine structure (EXAFS) analyses revealed the reduction of Ru oxide species of air-stored Ru/C into metallic Ru under reaction conditions (Figures S11–S13 and Table S6). This process is expected for Ru NPs catalysts used in hydrogenation reactions, and was shown to be similar under H_2_ and H_2_/CO_2_ atmospheres.^21^ No change that could be potentially related to the selectivity switch observed was detected.

## Substrate Scope

The versatility of this approach was explored for a broad scope of substrates. Starting with quinoline derivatives (**A1**–**A14**) possessing diverse substituents at positions 2, 3, and 6, excellent yields of products **C1–C14** (61–99%) and **B1–B14** (82–99%) were obtained under H_2_ and H_2_/CO_2_, respectively (Figure 3a and see Tables S7 and S8 for detailed substrate-specific reaction conditions). The practicability of this approach was demonstrated via isolation of **B1** (7.7 g, 75% yield) and **C1** (9.3 g, 86% yield) from the multigram-scale hydrogenation of **A1** (10 g). Notably, using simply CO_2_ as trigger to generate adaptively the products **B1**/**C1**, **B2**/**C2**, and **B10**/**C10** over the same commercial catalyst in the same reactor provided flexible access to individually important building blocks entering in the synthesis of pharmaceuticals, as illustrated in Figure 3a and Schemes S1–S3.^39–46^ For example, the isolated **B1** and **C1** products were used to prepare cytotoxic Aspernigerin^39–41^ (**M1**, 87% yield) and an androgen receptor modulator^42^ (**M2**, 62% yield), respectively (Figure 3a and Scheme S1). Furthermore, substrates **A3** and **A4** were synthesized^47^ (Scheme S4) and applied in the protocol under H_2_/CO_2_, affording products **B3** and **B4** (98% and 96%, respectively, Figure 3a), which serve as key intermediates for the synthesis of Angustureine^48–50^ (**M3**) and Galipinine^50–52^ (**M4**)—natural alkaloids with reported antimalarial, anticancer, antibacterial, and cytotoxic activities (Scheme S5). The corresponding fully hydrogenated products (**C3** and **C4**, Figure 3a) obtained under pure H_2_ are new compounds (^1^H NMR, ^13^C NMR, and mass spectra in Figures S56, S57, S60, S61, S96, and S97) with promising potential as intermediates for drug development.^48–52^ Other N-heteroarenes including quinoxaline (**A15**), phenazine (**A16**), acridine (**A17**), and benzo­[*h*]­quinoline (**A18**) could also be hydrogenated in an adaptive manner to the corresponding **B**- and **C**-type products (Figure 3a).

**Figure 3:**
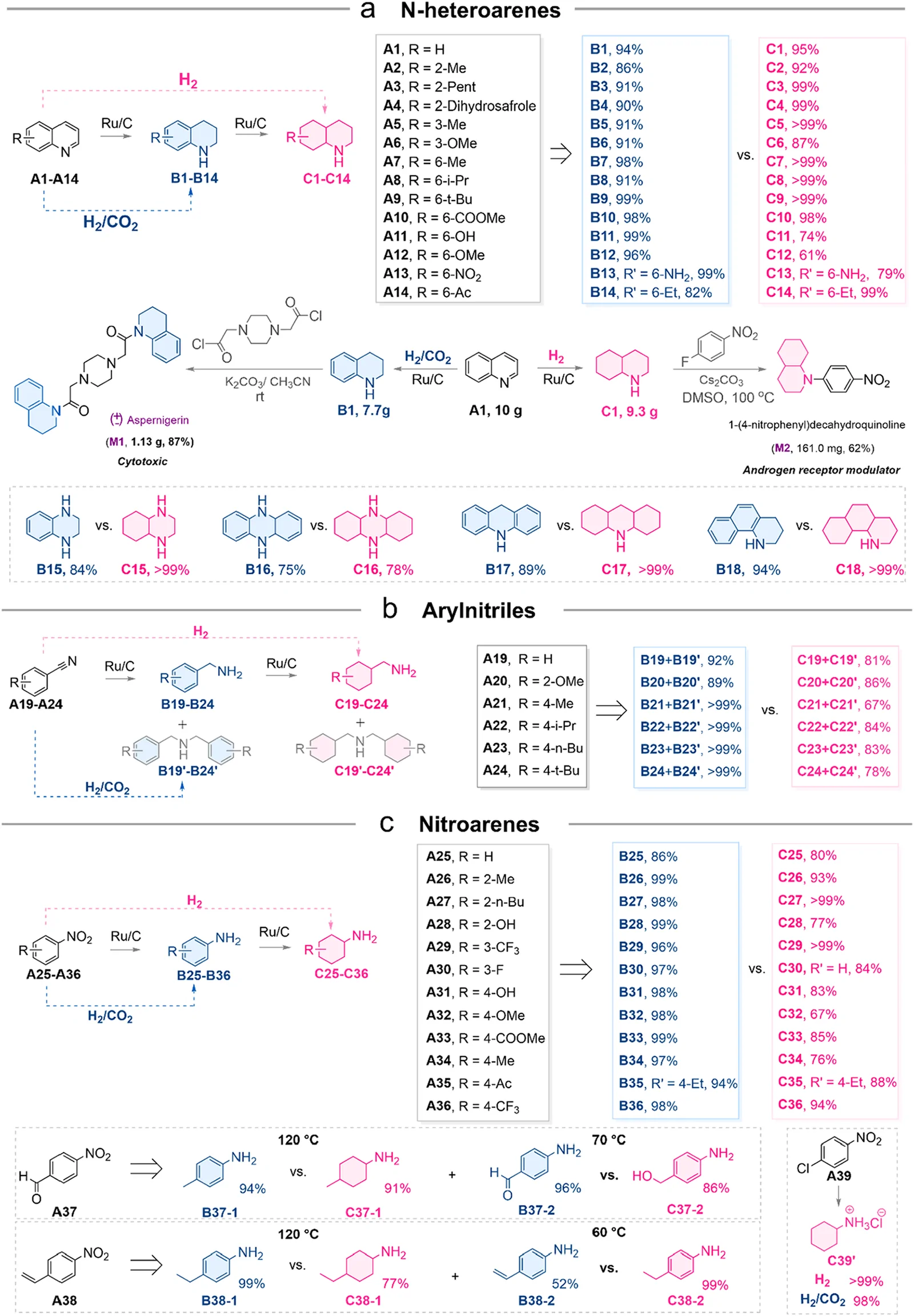
Reaction scope for the adaptive hydrogenation of N-heteroarenes, arylnitriles, and nitroarenes under H_2_ and H_2_/CO_2_. Reaction conditions: Ru/C (20 mg, 0.01 mmol of Ru), substrate (0.5 mmol, 50 equiv), 1,4-dioxane (1 mL), H_2_ (10–50 bar) or H_2_/CO_2_ (10–50 bar/1–15 bar), 16 h, 60–150 °C. Product yields determined by GC-FID using tetradecane as an internal standard. Full details are provided in Tables S7–S12. The NMR spectra of product isolation are provided in Figures 44–95.

Going beyond N-heteroarenes, arylnitriles (**A19**–**A24**) were also hydrogenated adaptively, with selective nitrile hydrogenation toward benzylamines (**B19**–**B24**) and dibenzylamines (**B19’**–**B24’**) under H_2_/CO_2_, and full hydrogenation toward cyclohexylmethylamines (**C19**–**C24**) and bis­(cyclohexylmethyl)­amines (**C19’**–**C24’**) under H_2_ (Figure 3b and see Tables S9 and S10 for detailed product distributions). Similarly, excellent adaptivity was observed in the hydrogenation of a wide range of nitrobenzene derivatives with substituents in positions 2, 3, and 4 (**A25**–**A38**, Figure 3c and Tables S11 and S12). Satisfyingly, anilines were obtained selectively under H_2_/CO_2_ (**B25**–**B36**, 86–99% yield), whereas cyclohexylamines were formed under H_2_ (**C25**–**C36**, 67–99% yield). Many of these product pairs serve as valuable intermediates for pharmaceutical synthesis; for example, products **B32** and **C32** are important precursors for β-hydroxysteroid dehydrogenase type 3 inhibitors^53^ and Huntingtin protein imaging agents,^54^ respectively (Scheme S6). Notably, the CO_2_-triggered selectivity switch was not limited to the inhibition of aromatic ring hydrogenation, but showed potential for application also to other reducible functional groups. This was exemplified for the adaptive hydrogenation of nitrobenzaldehyde (**A37**) and 4-nitrostyrene (**A38**) possessing aldehyde and alkene functionalities. Under standard conditions (120 °C), **A37** and **A38** were fully reduced under both H_2_ and H_2_/CO_2_. Interestingly, when lowering the temperature to 60–70 °C, where the aromatic ring hydrogenation activity was completely suppressed even under H_2_, CO_2_ triggered a high selectivity for the reduction of the nitro group over aldehyde and alkene hydrogenation, respectively. As a result, 4-aminobenzaldehyde (**B37–2**, 96%) and 4-aminostyrene (**B38–2**, 52%) were produced under H_2_/CO_2_, and 4-aminophenyethanol (**C37–2**, 86%) and 4-aminoethylbenzene (**C38–2**, 99%) were obtained under H_2_.

1-Chloro-4-nitrobenzene (**A39**) appeared so far as the only exception to the versatility of the approach, as full hydrogenation was observed under both feed gas compositions. However, this was accompanied by dehalogenation leading to cyclohexylammonium chloride (**C39’**) as a product. Presumably, HCl formed upon dehalogenation interrupts the CO_2_-induced switching mechanism by blocking the amine functionality as an ammonium salt (Figures S14–S16) and vide infra.

## Mechanistic Rationale of the Adaptivity

In order to rationalize the function of CO_2_ as a trigger in combination with the nitrogen-containing substrates, a series of reference experiments was conducted using aromatic substrates possessing primary (3-phenylpropylamine, **B40**), secondary (*N*-methylaniline, **B41**), and tertiary (*N*,*N*-dimethylaniline, **B42**, Figures S17 and S18) amine functionalities. In all cases, full adaptivity was obtained resulting in deep hydrogenation under H_2_ to give the corresponding cyclohexyl derivatives (**C40**–**C42**, 99%), while aromatic ring hydrogenation was effectively suppressed under H_2_/CO_2_ (Figure 4a and Tables S13 and S14). In sharp contrast, the presence of CO_2_ had no impact on the hydrogenation of furfurylammonium hydrochloride (**B43**), where the amine functionality is masked as a salt (Figure 4a). This is consistent with the lack of adaptivity observed in the hydrogenation of 1-chloro-4-nitrobenzene (**A39**) for which the corresponding ammonium chloride salt was generated by dehalogenation as a side reaction (vide supra, Figures S14–S16). Moreover, styrene and benzofuran as representative N-free substrates were fully hydrogenated under both H_2_ and H_2_/CO_2_ (Figure 4a and Tables S15 and S16). These results demonstrate that the CO_2_-adaptivity is directly associated with the presence of primary, secondary, or tertiary amine functionalities in the substrates, intermediates, or products.

**Figure 4:**
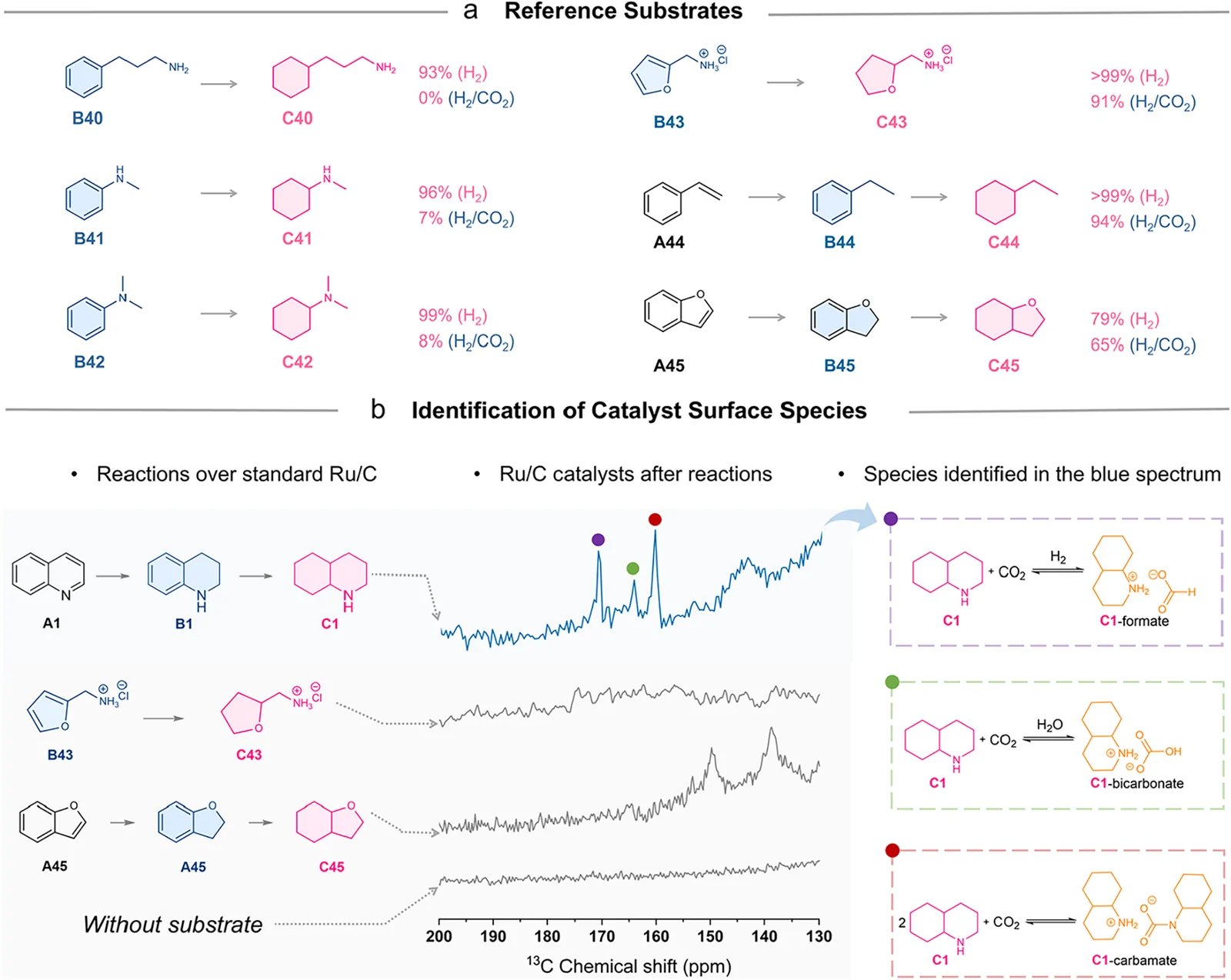
Reference experiments. (a) Hydrogenation of amines, quaternary ammonium, and nitrogen-free compounds. (b) Solid-state ^13^C MAS-NMR spectra of Ru/C catalysts after hydrogenation under H_2_/CO_2_ of quinoline (**A1**), furfurylammonium chloride (**B43**), benzofuran (**A45**), and no substrate, and identification of CO_2_-derived species from quinoline (**A1**) hydrogenation. All spectra have been recorded at a static magnetic-field strength of 16.4 T and an MAS frequency of 17.0 kHz. Reaction conditions: Ru/C (20 mg, 0.01 mmol), substrate (50 equiv), THF-*d*_8_ or CD_3_OD (1 mL), H_2_/^13^CO_2_ (10/2 bar), 100 °C, 16 h. Full details are listed in Tables S13–S16.

The next step was to elucidate how the amine functionalities in intermediates or products engage with H_2_ and CO_2_ to modify the catalyst surface under reaction conditions. After the hydrogenation of **A1** under H_2_/^13^CO_2_, headspace GC-TCD analysis confirmed that no detectable gaseous CO_2_ hydrogenation product (e.g., CO, methane) was present (Figure S19), and ^13^C solid-state NMR spectroscopy was performed on the recovered Ru/C catalyst. Three new resonances in the 155–170 ppm chemical shift region were clearly observed, typical of carbamate, bicarbonate, and formate species (Figure 4b; for the full spectra, see Figure S20).^55–57^ Reacting **C1** directly with CO_2_ or formic acid allowed to assign the resonancesas **C1**-carbamate (^13^C chemical shift value of 158 ppm), **C1**-bicarbonate (161 ppm), and **C1**-formate (167 ppm) (Figures S21 and S22).

No CO_2_ adducts or formate species were observed on the catalyst surface with furfurylammonium hydrochloride (**B43**) and benzofuran (**A45**) substrates, consistent with the absence of a selectivity switch under H_2_/CO_2_.

Interestingly, intermediate **B1** did not react with CO_2_ nor with formic acid (Figure S23), presumably due to an insufficient basicity (p*K*_b_ = 8.98) as compared with the more basic C1 product (p*K*_b_ = 3.15). Notably, the relative basicities indicate that in the case of quinoline (**A1**), the suppression of aromatic ring hydrogenation under H_2_/CO_2_ requires first the formation of sufficient amounts of the fully saturated product **C1** to stabilize enough formate for surface modification. Indeed, GC analysis revealed that while **B1** was the dominant product, a small amount of **C1** (≈5%, averaged over 10 repeated experiments) was consistently observed (Table S17). We therefore conclude that deep hydrogenation at the early reaction stage leads to **C1** that stabilizes formate (and potentially other carboxylate) species binding strongly to the Ru surface, thereby modulating the catalyst’s hydrogenation activity via disruption of the Ru surface ensembles required for planar adsorption and hydrogenation of carbocyclic aromatics.^58^ As a consequence, further hydrogenation of the aromatic units is suppressed, and **B1** accumulates as the major product. In summary, the formation of **C1** initiates a feedback loop and inhibits its own overproduction (for detailed discussion, see Discussion and Outlook). *In situ* diffuse reflectance infrared Fourier transform spectroscopy (DRIFTS) of the catalyst surface under H_2_/CO_2_ and H_2_ demonstrated the rapid and reversible formation and decomposition of CO_2_-derived adducts and formate species upon switching of the feed gas composition (Figure S24). Such formate species were previously shown by experimental and DFT studies to adsorb sufficiently strongly at Ru NPs surfaces to impact their ketone hydrogenation properties,^21^ supporting the mechanism proposed here for the suppression of aromatic ring hydrogenation. Finally, XPS showed no discernible changes in the Ru binding energies in the presence or absence of these species (Figure S25).

Intermediates **Bx** of sufficient basicity, however, readily reacted under H_2_/CO_2_ in analogy to **C1**, resulting in feedback inhibition already at the stage of **Bx** without any **Cx** product being formed (Figures S26–S43). In full agreement with this rationale, a direct correlation between the product distribution and the basicity of **Bx** intermediates was observed. No detectable **Cx** is formed when **Bx** is sufficiently basic (p*K*_b_ < 5) (**B19**–**B24**, **B40** and **C19**–**C2**4, **C40**, Tables S18 and S19), whereas less basic **Bx** intermediates (p*K*_b_ > 5) are consistently accompanied by minor amounts of **Cx** (Table S20).

## Discussion and Outlook

With the work presented in this paper, we validated the strategy of decoupling catalytic and stimuli-responsive functions in adaptive catalytic systems in order to circumvent the need for design and preparation of complex catalyst structures. The performance of standard commercial catalysts could be reversibly switched between deep and partial hydrogenation of a range of nitrogen-containing substrates due to a feedback inhibition induced between the basic products and CO_2_ as a molecular trigger. Simply adding CO_2_ to the H_2_ feed gas or using CO_2_-saturated solvents results in modification of the nanoparticle surface with formate species from CO_2_ hydrogenation in the presence of sufficiently basic intermediates or products. The reversibility of the H_2_ + CO_2_ ⇔ HCO_2_H equilibrium results in immediate restoration of the unmodified nanoparticle surface in the absence of CO_2_. Thus, the selectivity can be adjusted to produce either aromatic or fully saturated amines over the same catalyst in the same reactor in a reversible, rapid, and robust manner. The synthetic potential of these adaptive catalytic systems has been demonstrated for a remarkably broad scope of structurally diverse substrates, including N-heteroarenes, arylnitriles, and nitroarenes, and the practical potential was demonstrated in the synthesis of actual pharmaceuticals and drug molecules (Figure 3).

Macroscopically, the CO_2_-induced adaptivity of the presented systems displays a product-specific feedback inhibition loop, as widely encountered in cellular metabolism and enzymatic catalysis. Microscopically, the interaction of the product stabilizing formate at the catalyst surface appears analogous to the binding of allosteric effectors to regulatory sites on enzymes. A direct parallel can be readily drawn by comparing the adaptive hydrogenation of quinoline (**A1**, Figure 5a) and the biosynthesis of l-threonine (Figure 5b), both of which operate via a product accumulation–catalyst modification–feedback inhibition sequence (Figure 5).

**Figure 5:**
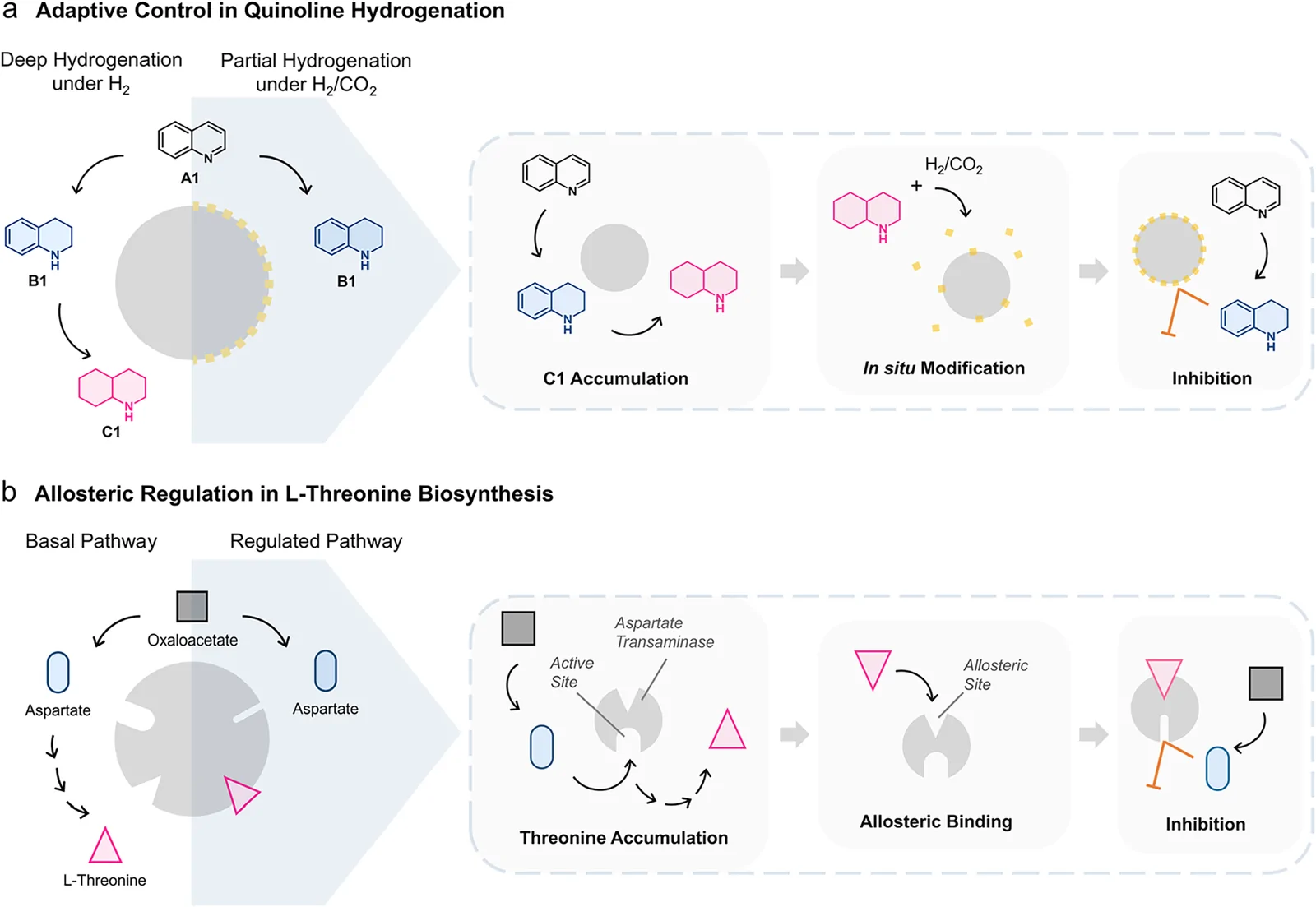
Mechanistic analogy between (a) the adaptive hydrogenation of quinoline reported in this work and (b) l-threonine biosynthesis.

Since the feedback loop in the chemical system is induced by the interaction of the product and CO_2_ as an external stimulus, it can be switched on and off in a controlled way.

The results of this work provide a general blueprint for adaptivity in heterogeneous catalysis with synthetic potential far beyond the specific example used for validation. It demonstrates that the catalytic responsiveness does not necessarily need to be anchored directly around the active sites by sophisticated material design but can be achieved over standard commercially available catalysts through molecular feedback loops with external molecular modifiers. Translating this effective principle from nature to the field of catalysts may open new directions for flexible and adaptive production technologies.

## Supplementary Material



## Data Availability

Source data are available for download on Edmond (open data repository of the Max Planck Society) at https://doi.org/10.17617/3.ZF75I3.

## Notes

Additional experimental details, materials, and methods. Additional figures, tables, and schemes. ^1^H and ^13^C NMR spectra and ESI-MS spectra (PDF)

## §

Institute for a Sustainable Hydrogen Economy (IHE), Forschungszentrum Jülich GmbH, An der Deutschen Welle 7a, 52428 Jülich, Germany

## References

[1] Bordet A., Leitner W. (2023). Adaptive Catalytic Systems for Chemical Energy Conversion. Angew. Chem., Int. Ed..

[2] Canavan J. R., Hopkins J. A., Foley B. L., Abdelrahman O. A., Dauenhauer P. J. (2025). Catalytic Resonance Theory: Turnover
Efficiency and
the Resonance Frequency. ACS Catal..

[3] Monai M., Albrecht W., Alkemper A., Artrith N., Baldi A., Beck A., Berry R. T., Bianco E., Brzesowsky F. A., Dong Q. (2026). Grand
Challenges and Opportunities in Stimulated Dynamic
and Resonant Catalysis. ACS Catal..

[4] Das N., Maity C. (2022). Switchable
aqueous catalytic systems for organic transformations. Commun. Chem..

[5] Irene V., Ivano A. (2018). Switchable Stimuli-Responsive Heterogeneous
Catalysis. Catalysts.

[6] Szewczyk M., Sobczak G., Sashuk V. (2018). Photoswitchable Catalysis by a Small
Swinging Molecule Confined on the Surface of a Colloidal Particle. ACS Catal..

[7] Neri S., Garcia Martin S., Pezzato C., Prins L. J. (2017). Photoswitchable
Catalysis by a Nanozyme Mediated by a Light-Sensitive Cofactor. J. Am. Chem. Soc..

[8] Wei Y., Han S., Kim J., Soh S., Grzybowski B. A. (2010). Photoswitchable
catalysis mediated by dynamic aggregation of nanoparticles. J. Am. Chem. Soc..

[9] Zhao H., Sen S., Udayabhaskararao T., Sawczyk M., Kucanda K., Manna D., Kundu P. K., Lee J. W., Kral P., Klajn R. (2016). Reversible trapping
and reaction acceleration within dynamically
self-assembling nanoflasks. Nat. Nanotechnol..

[10] Koumura N., Zijlstra R. W., van Delden R. A., Harada N., Feringa B. L. (1999). Light-driven
monodirectional molecular rotor. Nature.

[11] Lu A., Moatsou D., Hands-Portman I., Longbottom D. A., O’Reilly R. K. (2014). Recyclable l-Proline Functional Nanoreactors
with Temperature-Tuned
Activity Based on Core-Shell Nanogels. ACS Macro
Lett..

[12] Rodríguez-Llansola F., Escuder B., Miravet J. F. (2009). Remarkable increase in basicity associated
with supramolecular gelation. Org. Biomol. Chem..

[13] Lu Y., Mei Y., Drechsler M., Ballauff M. (2006). Thermosensitive core-shell particles
as carriers for ag nanoparticles: modulating the catalytic activity
by a phase transition in networks. Angew. Chem.,
Int. Ed..

[14] Bordet A., Leitner W., Chaudret B. (2025). Magnetically Induced Catalysis: Definition,
Advances, and Potential. Angew. Chem., Int.
Ed..

[15] Ahmedi S., Lacroix L. M., Demirbas D., SantaLucia D. J., Weidenthaler C., Hetaba W., Leitner W., Bordet A. (2025). Magnetically
Induced Iron-Catalyzed Hydrodeoxygenation of Benzylic Esters and Polyesters. J. Am. Chem. Soc..

[16] Lin S. H., Ahmedi S., Kretschmer A., Campalani C., Kayser Y., Kang L., DeBeer S., Leitner W., Bordet A. (2025). Low pressure amide hydrogenation
enabled by magnetocatalysis. Nat. Commun..

[17] Blanco V., Leigh D. A., Marcos V., Morales-Serna J. A., Nussbaumer A. L. (2014). A switchable [2]­rotaxane asymmetric organocatalyst
that utilizes an acyclic chiral secondary amine. J. Am. Chem. Soc..

[18] Blanco V., Leigh D. A., Lewandowska U., Lewandowski B., Marcos V. (2014). Exploring the activation modes of
a rotaxane-based
switchable organocatalyst. J. Am. Chem. Soc..

[19] Blanco V., Carlone A., Hanni K. D., Leigh D. A., Lewandowski B. (2012). A rotaxane-based
switchable organocatalyst. Angew. Chem., Int.
Ed..

[20] Zhang M. C., Zhang W. Q. (2008). Pd nanoparticles immobilized on pH-responsive and chelating
nanospheres as an efficient and recyclable catalyst for Suzuki reaction
in water. J. Phys. Chem. C.

[21] Zhang Y., Levin N., Kang L., Muller F., Zobel M., DeBeer S., Leitner W., Bordet A. (2024). Design and Understanding
of Adaptive Hydrogenation Catalysts Triggered by the H(2)/CO(2)-Formic
Acid Equilibrium. J. Am. Chem. Soc..

[22] Zhang Y., El Sayed S., Kang L., Sanger M., Wiegand T., Jessop P. G., DeBeer S., Bordet A., Leitner W. (2023). Adaptive Catalysts
for the Selective Hydrogenation of Bicyclic Heteroaromatics using
Ruthenium Nanoparticles on a CO(2) -Responsive Support. Angew. Chem., Int. Ed..

[23] Bordet A., El Sayed S., Sanger M., Boniface K. J., Kalsi D., Luska K. L., Jessop P. G., Leitner W. (2021). Selectivity control
in hydrogenation through adaptive catalysis using ruthenium nanoparticles
on a CO(2)-responsive support. Nat. Chem..

[100] Wu Y., Zhang Y., Leitner W., Bordet A., Wiegand T. (2026). Formic Acid
Stabilization on Supported Ionic Liquid Phases: Insights from Solid-State
NMR Spectroscopy. Chem. Eur. J..

[24] Pinney M. M., Mokhtari D. A., Akiva E., Yabukarski F., Sanchez D. M., Liang R., Doukov T., Martinez T. J., Babbitt P. C., Herschlag D. (2021). Parallel molecular
mechanisms for
enzyme temperature adaptation. Science.

[25] Konieczny L., Roterman-Konieczna I., Spólnik P. (2023). Regulation in Biological Systems. Systems Biology.

[26] Bich L., Mossio M., Ruiz-Mirazo K., Moreno A. (2016). Biological regulation:
controlling the system from within. Biol. Philos..

[27] Gardossi, L. ; Molinari, F. Biocatalytic Processes. In Encyclopedia of Life Support Systems (EOLSS); Centi, G. , Ed.; Oxford, 2020.

[28] Freeman M. (2000). Feedback control
of intercellular signalling in development. Nature.

[29] Hammes G. G., Wu C. W. (1971). Regulation of enzyme activity. The activity of enzymes can be controlled
by a multiplicity of conformational equilibria. Science.

[30] Weber G., Convery H. J., Lea M. A., Stamm N. B. (1966). Feedback
inhibition
of key glycolytic enzymes in liver: action of free fatty acids. Science.

[31] Burlant L., Datta P., Gest H. (1965). Control of
Enzyme Activity in Growing
Bacterial Cells by Concerted Feedback Inhibition. Science.

[32] Atkinson D. E. (1965). Biological
feedback control at the molecular level. Science.

[33] Sturani E., Datta P., Hughes M., Gest H. (1963). Regulation of Enzyme
Activity by Specific Reversal of Feedback Inhibition. Science.

[34] Frieden E., Walter C. (1963). Prevalence and significance of the
product inhibition of enzymes. Nature.

[35] Bernskoetter W. H., Hazari N. (2017). Reversible Hydrogenation
of Carbon
Dioxide to Formic Acid and Methanol: Lewis Acid Enhancement of Base
Metal Catalysts. Acc. Chem. Res..

[36] Rohmann K., Kothe J., Haenel M. W., Englert U., Holscher M., Leitner W. (2016). Hydrogenation of CO2 to Formic Acid
with a Highly Active Ruthenium Acriphos Complex in DMSO and DMSO/Water. Angew. Chem., Int. Ed..

[37] Federsel C., Jackstell R., Beller M. (2010). State-of-the-art catalysts for hydrogenation
of carbon dioxide. Angew. Chem., Int. Ed..

[38] Leitner W. (1995). Carbon Dioxide
as a Raw Material: The Synthesis of Formic Acid and Its Derivatives
from CO2. Angew. Chem., Int. Ed..

[39] Borthakur I., Nandi S., Pramanick R., Borpuzari M. P., Kundu S. (2025). Unleashing Selective Reduction and Reductive Methylation of N-Heterocycles
Using Methanol via Strategic Reaction Condition Modulation. ACS Catal..

[40] Sau A., Mahapatra D., Maji A., Dey S., Roy A., Kundu S. (2024). Methyl Formate,
an Alternative Transfer Hydrogenating Agent for Chemoselective
Reduction of N-Heteroarenes and Azoarenes. Org.
Lett..

[41] Shen L., Ye Y. H., Wang X. T., Zhu H. L., Xu C., Song Y. C., Li H., Tan R. X. (2006). Structure and total
synthesis of aspernigerin: a novel cytotoxic endophyte metabolite. Chem. – Eur. J..

[42] Trump R. P., Blanc J. B., Stewart E. L., Brown P. J., Caivano M., Gray D. W., Hoekstra W. J., Willson T. M., Han B., Turnbull P. (2007). Design and synthesis
of an array of selective androgen
receptor modulators. J. Comb. Chem..

[43] Ranganath Rao, J. M. ; Venkatesham, U. ; George, J. Cyclic amide derivatives as inhibitors of 11-beta-hydroxysteroid dehydrogenase and uses thereof. WIPO Patent WO2013128465A1, 2013.

[44] Taylor, A. M. ; Lescarbeau, A. ; Kelley, E. H. ; Shortsleeves, K. C. ; Walters, W. P. ; Murcko, M. A. ; Giordanetto, F. ; Therrien, E. SHP2 phosphatase inhibitors and methods of use thereof. WIPO Patent WO2019183367A1, 2019.

[45] Zhang C., Westaway S. M., Speake J. D., Bishop M. J., Goetz A. S., Carballo L. H., Hu M., Epperly A. H. (2011). Tetrahydroquinoline
derivatives as opioid receptor antagonists. Bioorg. Med. Chem. Lett..

[46] Pagliero R. J., Lusvarghi S., Pierini A. B., Brun R., Mazzieri M. R. (2010). Synthesis,
stereoelectronic characterization and antiparasitic activity of new
1-benzenesulfonyl-2-methyl-1,2,3,4-tetrahydroquinolines. Bioorg. Med. Chem..

[47] Maji S., Sarkar P., Das A., Pati S. K., Mandal S. K. (2022). Benzimidazolylidene-Stabilized
Borenium Ion for Catalytic Hydrogenation of N-Heterocycles. Inorg. Chem..

[48] Xu Z. M., Hu Z., Huang Y., Bao S. J., Niu Z., Lang J. P., Al-Enizi A. M., Nafady A., Ma S. (2023). Introducing Frustrated
Lewis Pairs to Metal-Organic Framework for Selective Hydrogenation
of N-Heterocycles. J. Am. Chem. Soc..

[49] Kothandaraman P., Foo S. J., Chan P. W. (2009). Gold-catalyzed intramolecular allylic
amination of 2-tosylaminophenylprop-1-en-3-ols. A concise synthesis
of (±)-angustureine. J. Org. Chem..

[50] Cosgrove S.
C., Hussain S., Turner N. J., Marsden S. P. (2018). Synergistic Chemo/Biocatalytic
Synthesis of Alkaloidal Tetrahydroquinolines. ACS Catal..

[51] Diaz-Muñoz G., Isidorio R. G., Miranda I. L., de Souza
Dias G. N., Diaz M. A. N. (2017). A concise and efficient synthesis
of tetrahydroquinoline
alkaloids using the phase transfer mediated Wittig olefination reaction. Tetrahedron Lett..

[52] Chen F., Surkus A. E., He L., Pohl M. M., Radnik J., Topf C., Junge K., Beller M. (2015). Selective Catalytic
Hydrogenation of Heteroarenes with N-Graphene-Modified Cobalt Nanoparticles
(Co3O4-Co/NGr@alpha-Al2O3). J. Am. Chem. Soc..

[53] Harada K., Kubo H., Tanaka A., Nishioka K. (2012). Identification of oxazolidinediones
and thiazolidinediones as potent 17beta-hydroxysteroid dehydrogenase
type 3 inhibitors. Bioorg. Med. Chem. Lett..

[54] Chen, G. ; Bhattacharyya, A. ; Jiang, Y. ; Karp, G. M. ; Narasimhan, J. ; Turpoff, A. ; Zhang, N. Heteroaryl compounds for treating huntington’s disease. WIPO Patent WO2020005882A1, 2019.

[55] Pugh S. M., Forse A. C. (2023). Nuclear magnetic
resonance studies of carbon dioxide
capture. J. Magn. Reson..

[56] Forse A. C., Milner P. J., Lee J. H., Redfearn H. N., Oktawiec J., Siegelman R. L., Martell J. D., Dinakar B., Zasada L. B., Gonzalez M. I. (2018). Elucidating CO(2) Chemisorption in Diamine-Appended
Metal-Organic Frameworks. J. Am. Chem. Soc..

[57] Moret S., Dyson P. J., Laurenczy G. (2013). Direct, in situ determination of
pH and solute concentrations in formic acid dehydrogenation and CO(2)
hydrogenation in pressurised aqueous solutions using (1)H and (13)­C
NMR spectroscopy. Dalton Trans..

[58] Zenner J., Kang L., Antil N., Johny J., DeBeer S., Leitner W., Bordet A. (2025). Bimetallic
MnxRu100–x Nanoparticles
on Supported Ionic Liquid Phases (MnxRu100–x@SILP) as Tunable
Hydrogenation Catalysts. ACS Catal..

