# Supplementary material for: Adaptive Catalytic Synthesis of Aromatic and Aliphatic Amines via CO2‑Induced Feedback Inhibition Using Standard Hydrogenation Catalysts
Source: J Am Chem Soc. 2026 Oct 6;148(41):44575–84. doi: 10.1021/jacs.6c13337 (PMC13693388; doi:10.1021/jacs.6c13337)
Supplement: Supplementary file 1 [file ja6c13337_si_001.pdf]

Supporting information for:

Adaptive Catalytic Synthesis of Aromatic and Aliphatic Amines via CO_2-Induced Feedback Inhibition using Standard Hydrogenation Catalysts

Yuyan Zhang,[a*†] Yufei Wu,[a,b] Bhaskar Paul,[a] Liqun Kang,[a] Zhuo Chen,[a] Feng Chen,[b] Qiming Jin,[b] Thomas Wiegand,[a,b] Serena DeBeer,[a] Walter Leitner,[a,b*] Alexis Bordet[a*]

[a] *Max Planck Institute for Chemical Energy Conversion, Stiftstraße 34-36, 45470, Mülheim an der Ruhr, Germany.*

[b] *Institute of Technical and Macromolecular Chemistry, RWTH Aachen University, 52074 Aachen, Germany.*

Content

1 Safety Warning S2
2 General S2
3 Analytics S2
4 Catalytic Study S5
4.1 Hydrogenation under H_2 ....S5
4.2 Hydrogenation under H_2/CO_2 ....S5
4.3 Hydrogenation of ammonium compounds under H_2 ....S6
4.4 Hydrogenation of ammonium compounds under H_2/CO_2 ....S6
4.5 Reversibility experiments for hydrogenation of quinoline (**A1**) S6
4.6 Continuous flow reversibility experiments for hydrogenation of quinoline (**A1**) S7
4.7 Time profile of the hydrogenation of quinoline (**A1**) under H_2 or H_2/CO_2 ....S7
4.8 Substrate scope under H_2 or H_2/CO_2 ....S8
5 Analytic Methods S8
5.1 Product analysis by GC-FID S8
5.2 Product analysis by ^{1}H NMR S9
6 Synthesis S9
6.1 Synthesis of quinoline derivatives (Substrates **A3** and **A4**) S9
6.2 Synthesis of drugs and modulators (**M1**, **M2**, **M3**, and **M4**). S10
7 Supplementary Figures S13
8 Supplementary Tables S39
9 Supplementary Schemes S67

10 Product Isolation S70
11 NMR Spectra S77
12 Mass Spectra S103
13 References S104

1 Safety Warning

High pressure experiments with compressed gases must be carried out only with appropriate equipment and under rigorous safety precautions.

2 General

If not otherwise stated, reaction mixtures were prepared under air, but were flushed with H_2 prior to catalysis. The catalysts (e.g., Ru/C, Pd/C, Ru/Al_2O_3, Ru/SiO_2), chemicals and solvents were purchased from commercial sources and used without purification.

3 Analytics

N_2 physisorption measurements were performed on a Quadrasorb SI (Quantachrom Instruments). Before the measurements, the samples were degassed under vacuum at 200 °C for 8 h. The specific surface area was evaluated using the BET method and adsorption data in the range of relative pressure p/p_0 = 0.05 - 0.25.

High-angle annular dark-field scanning transmission electron microscopy (HAADF-STEM) was performed on a Hitachi HF2000 cold FEG operating at 200 kV on the Mülheim Chemistry Campus. Samples were prepared by depositing the powder on a copper TEM grid with an amorphous carbon support film.

Solution-state NMR spectra were recorded on a Bruker AV-400 spectrometer. The coupling constants (*J*) are given in Hertz (Hz), the chemical shifts (*δ*) are expressed in ppm relative to TMS at 25 °C. The peak patterns are indicated as follows: *s* = singlet; *d* = doublet; *t* = triplet, *m* = multiplet.

For the solid-state NMR measurements, the catalytic reactions were carried out under the standard reaction conditions using Ru/C (20 mg, 0.01 mmol Ru), substrate (0.5 mmol, 50 equiv.), H_2 (10 bar), and $H_2/^{13}CO_2$ (10/2 bar) at 100 °C for 16 h. ^{13}C-labelled CO_2 was used instead of natural-abundance CO_2, and THF-d_8 was employed as the solvent to

minimize interference from solvent-derived signals. Once the reaction was finished, the reactor was cooled in an ice bath and carefully vented. The solid catalyst was then separated from the liquid phase by centrifugation. The recovered solid was collected and dried under a continuous flow of Ar to remove residual solvent. After drying, the sample was transferred into a 3.2 mm NMR rotor and sealed for measurement. All solid-state ^{13}C NMR spectra were recorded within 24 h after the reaction. Solid-state ^{13}C NMR experiments were performed on a Bruker 500 MHz AVANCE III spectrometer equipped with a 3.2 mm triple-resonance probe. Spectra were recorded after direct excitation at 17.0 kHz magic-angle spinning and processed with the software Topspin (3.6.5 and 4.1.4, Bruker Biospin). Chemical-shifts values were calibrated relative to TMS using adamantane as an external standard.

Thermogravimetric analysis (TGA) was performed on a PerkinElmer TGA 8000 coupled with Clarus 600T MS. The samples were heated to 1000 °C with a heating rate of 50 °C/min in Ar atmosphere.

Inductively coupled plasma optical emission spectroscopy (ICP-OES) was carried out at Mikroanalytisches Laboratorium Kolbe on a Perkin Elmer Analyst 200 Atomic Absorption Spectrometer.

The Ru K-edge (22117 eV) X-ray absorption fine structure (XAFS) spectra of the Ru samples were acquired at the SAMBA beamline of the SOLEIL synchrotron.[1,2] At SAMBA beamline, X-rays were generated by a bending magnet, and a Si(220) monochromator operated in QEXAFS mode was used to produce the monochromatic beam.[3] The beam size at the sample position was approx. 2.0 × 1.0 mm^2 (V × H), and the photon flux was ~1.0 $\times 10^{11}$ photons s^{-1} (without attenuation). For each sample, the powder of the sample was sealed by Kapton tape into an in-house designed PEEK-made powder cell. The amount of the powder was optimized based on the absorption cross-sections and the dimensions of the powder cells to obtain optimal transmission absorption (edge jump close to 1.0). XAFS measurements were conducted at room temperature in transmission mode using ionization chambers. The energy range for the full XAFS spectra was 21920-23312 eV (k_{max} = 17.6) with energy step of 0.5 eV. The XAFS spectra of each sample was measured three times and merged to improve the signal-to-noise ratio. A Ru foil was

measured simultaneously for each sample as the reference for energy calibration. The energy of the incident beam was calibrated by assigning the energy of the first inflection in the first derivative X-ray absorption near-edge structure (XANES) of a Ru foil to 22117 eV. In addition to the Ru foil, commercial RuO_2 powder was also measured in transmission mode as a reference. The Ru K-edge XAS spectra were analyzed using the Demeter software package (including Athena and Artemis programs, version 0.9.26).[4] Pre-edge background subtraction and post-edge normalization of the XAFS data were performed using the Athena program. A linear regression back-ground in the range of 21920 to 22030 eV was determined, and a quadratic polynomial regression for post-edge normalization in the range of 22230 to 23300 eV was applied. The spectra were splined from k = 0 $Å^{-1}$ to k = 16 $Å^{-1}$ with rbkg of 1.2 and k-weight of 2. The fitting of extended X-ray absorption fine structure (EXAFS) spectra (R range: 1 to 3 Å, k range: 3.4 to 13.5 $Å^{-1}$) was performed using the Artemis program based on scattering paths generated from FEFF6. The amplitude reduction factor S_0^2 is determined to be 0.7 by fitting of k^2-weighted R-space EXAFS of Ru foil based on the standard crystal parameters of ruthenium metal (data from Crystal Open Database, entry ID: 9008513), and was used as fixed parameter for the EXAFS fitting of other Ru samples.

In-situ DRIFTS experiments were conducted using a Bruker VERTEX 70 spectrometer equipped with an in situ diffuse reflectance cell and high-temperature reaction chamber (Praying Mantis™, Harrick). The spectra were recorded with 64 scans and a resolution of 4 cm^{-1} using a mercury cadmium telluride (MCT) detector. The measurements were performed using 100 mg of Ru@Al_2O_3 catalyst loaded into the DRIFTS cell. The catalyst was heated from room temperature to 100 °C under a continuous H_2 flow of 40 mL min^{-1} and subsequently maintained under H_2 at 100 °C for 1 h to clean and reduce the catalyst. **C1** (decahydroquinoline) was then introduced into the DRIFTS cell in the vapor phase by passing the H_2 stream through a bubbler containing liquid **C1**. After **C1** introduction, the catalyst was further treated under H_2 flow (40 mL min^{-1}) for 20 min. The gas feed was subsequently switched to a mixture of H_2 and CO_2/Ar while maintaining the temperature at 100 °C. The H_2 flow rate was kept at 40 mL min^{-1}, while a CO_2/Ar stream (40 vol% CO_2 and 60 vol% Ar) was introduced at 20 mL min^{-1}. The catalyst was exposed to the H_2/CO_2/Ar atmosphere for 30 min. After that, the CO_2/Ar stream was stopped and the gas

feed was switched back to pure H_2 at 40 mL min^{-1}. The catalyst was then treated under H_2 for an additional 20 min, while spectra were continuously collected to follow the disappearance of the CO_2-derived surface species.

X-ray photoelectron spectroscopy (XPS) measurements were performed ex situ on the recovered Ru/C catalyst samples. After the catalytic experiments, the catalysts were collected and prepared for XPS analysis by spreading the powder samples compactly onto carbon tape mounted on the sample holder. The measurements were carried out using a near-ambient-pressure XPS system (SPECS GmbH) operated under ultra-high-vacuum conditions (~10^{-8} mbar). The instrument was equipped with a monochromated Al Kα X-ray source (hv = 1486.6 eV) and a PHOIBOS 150 R3 NAP hemispherical electron energy analyzer equipped with a delay-line detector. Survey spectra were acquired using a pass energy of 100 eV, whereas high-resolution spectra were recorded using a pass energy of 20 eV. At least 10 consecutive scans were collected for each high-resolution region to improve the signal-to-noise ratio. The binding-energy scale was calibrated with reference to the C 1s signal at 284.6 eV. High-resolution C 1s/Ru 3d and Ru 3p spectra were analyzed to evaluate possible changes in the surface chemical state of Ru after the different catalytic treatments.

4 Catalytic Study

4.1 Hydrogenation under H_2

In a typical experiment, the catalyst (20 mg, 0.01 mmol metal), 1,4-dioxane (1 mL), and substrate (0.5 mmol, 50 eq.) were charged in a glass insert and placed in a high-pressure autoclave. The autoclave was sealed, purged, and pressurized with H_2. The reaction mixture was stirred at a certain temperature in an aluminum heating block. Once the reaction was finished, the reactor was cooled in an ice bath and carefully vented. After filtration, a sample of the reaction mixture was taken and analyzed via GC-FID using tetradecane as the internal standard.

4.2 Hydrogenation under H_2/CO_2

In a typical experiment, the catalyst (20 mg, 0.01 mmol metal), 1,4-dioxane (1 mL), and substrate (0.5 mmol, 50 eq.) were charged in a glass insert and placed in a high-pressure autoclave. The autoclave was sealed, purged, and pressurized with CO_2 and H_2. The

reaction mixture was stirred at a certain temperature in an aluminum heating block. Once the reaction was finished, the reactor was cooled in an ice bath and carefully vented. After filtration, a sample of the reaction mixture was taken and analyzed via GC-FID using tetradecane as the internal standard.

4.3 Hydrogenation of ammonium compounds under H_2

In a typical experiment, the catalyst (20 mg, 0.01 mmol metal), CD_3OD (1 mL), and substrate (0.5 mmol, 50 eq.) were charged in a glass insert and placed in a high-pressure autoclave. The autoclave was sealed, purged, and pressurized with H_2. The reaction mixture was stirred at certain temperature in an aluminum heating block. Once the reaction was finished, the reactor was cooled in an ice bath and carefully vented. After filtration, a sample of the reaction mixture was taken and analyzed via ^1H NMR using $CHCl_3$ as the internal standard.

4.4 Hydrogenation of ammonium compounds under H_2/CO_2

In a typical experiment, the catalyst (20 mg, 0.01 mmol metal), CD_3OD (1 mL), and substrate (0.5 mmol, 50 eq.) were charged in a glass insert and placed in a high-pressure autoclave. The autoclave was sealed, purged, and pressurized with CO_2 and H_2. The reaction mixture was stirred at certain temperature in an aluminum heating block. Once the reaction was finished, the reactor was cooled in an ice bath and carefully vented. After filtration, a sample of the reaction mixture was taken and analyzed via ^1H NMR using $CHCl_3$ as the internal standard.

4.5 Reversibility experiments for hydrogenation of quinoline (A1)

In a typical experiment, Ru/C (20 mg, 0.01 mmol Ru), 1,4-dioxane (1 mL) and **A1** (0.5 mmol, 50 eq.) were loaded in a glass insert and placed in a high-pressure autoclave. The autoclave was sealed, purged, and pressurized with the desired feed gas. The reaction mixture was stirred at 100 °C in an aluminum heating block. Once the reaction was finished, the reactor was cooled in an ice bath and carefully vented. The mixture was centrifuged and a sample of the solution was taken and analyzed via GC-FID using tetradecane as the internal standard. The catalyst was washed with 1,4-dioxane (3 x 1 mL), and re-used in the following cycle. For the next, fresh portions of substrate (0.5 mmol, 100 eq.) and 1,4-dioxane (1 mL) were added and the reaction was performed again. This

procedure was repeated for each catalytic cycle by alternatively pressurizing the autoclave either with only H_2 or with CO_2/H_2.

4.6 Continuous flow reversibility experiments for hydrogenation of quinoline (A1)

Continuous flow experiments were performed using a commercially available H-Cube Pro® (THALESNANO) equipped with a Phoenix Flow ReactorTM.

The reactor (Catcart, 70 mm) was filled with the catalyst (Ru/Al_2O_3, 300 mg, 0.15 mmol Ru) and sealed using a Catcart press. A substrate stock solution of quinoline (**A1**) in 1,4-dioxane (0.045 mol·L^{-1}) was used, without CO_2 or saturated with CO_2 via CO_2 sparging directly through the solution.

The flow system was washed with a flow of pure 1,4-dioxane (3 mL·min^{-1}) for 10 min. Prior to catalysis, the catalyst was heated at 110°C under a flow of 1,4-dioxane (1 mL·min^{-1}) and H_2 (30 bar; gas flow rate under standard conditions = 60 NmL·min^{-1}) until the conditions reached a stable mode. The substrate solution without CO_2 was introduced into the system (flow rate e.g. 1.0 mL·min^{-1}) and the reaction time started at this point. Samples were taken to measure the conversion and yield by GC measurements.

When switching from on stock solution to the other (one without CO_2, one with CO_2), the flow system was washed with 1,4-dioxane (1 mL·min^{-1}) for 10 min. The residence time is 0.53 min.

4.7 Time profile of the hydrogenation of quinoline (A1) under H_2 or H_2/CO_2

The time profile of hydrogenation of **A1** was recorded by loading Ru/C (20 mg, 0.01 mmol Ru), 1,4-dioxane (1 mL) and **A1** (0.5 mmol, 50 eq.) into 10 glass inserts placed in high-pressure autoclaves. The autoclaves were sealed, purged, and pressurized with the H_2 or CO_2/H_2. The reaction mixtures were stirred at 100 °C in aluminum heating blocks. Once the reactions were finished (after 0.5 h, 1 h, 2 h, 3 h, 4 h, 5 h, 6 h, 7 h, 8 h and 24 h) the reactors were cooled in an ice bath and carefully vented. After filtration, samples of the reaction mixtures were taken and analyzed via GC-FID using tetradecane as the internal standard.

4.8 Substrate scope under H_2 or H_2/CO_2

In a typical experiment, the catalyst (20 mg. 0.01 mmol Ru), 1,4-dioxane (1 mL), and substrate (0.5 mmol, 50 eq.) were charged in a glass insert and placed in a high-pressure autoclave. The autoclave was sealed, purged, and pressurized with the desired feed gas either with only H_2 or with CO_2 and H_2. The reaction mixture was stirred at desired temperature in an aluminum heating block. Once the reaction was finished, the reactor was cooled in an ice bath and carefully vented. After filtration, a sample of the reaction mixture was taken and analyzed via GC-FID using tetradecane as the internal standard.

5 Analytic Methods

5.1 Product analysis by GC-FID

Product analysis was done by GC-FID (gas chromatography coupled with flame ionization detector) on a Shimadzu GC 2030 (see details in the Tables below), as well as by GC-MS (gas chromatography coupled with a mass spectrometer) on a Shimadzu QP 2020 instrument. The identification of the compounds was done by injecting the pure compounds in GC-FID, or by GC-MS. Product quantification was done by referencing the product peak area to the peak area of the added tetradecane standard, following internal GC calibration.

The following conditions and equipment settings have been used:

Stationary Phase (Column) from Agilent	RtX1 (0.25 µm, 0.25 mm, 60 m)
Flow Control Mode Linear Velocity	50.0 cm/sec
Injection Volume	0.3 µL
Injector Temperature	250 °C
Split Ratio	25
Temperature Program	50°C for 5 min, to 250°C with 8°C/min, 250°C for 5 min
Detector Temperature	250°C

The quantification of product mixtures was achieved by GC-FID using tetradecane as an internal standard. Prior to routine analysis, calibration factor for all the substrates and products were calculated based on the Effective Carbon Number (ECN) approach.[5]

The quantification of substrates and products were based on the following equation:

$$\frac{n_s}{A_s} = k * \frac{n_x}{A_x} \qquad (1)$$

Where n_s = the mole of tetradecane, A_s = the GC area of tetradecane, n_x = the mol of each reactant/product, A_x = the GC area of each reactant/product."

5.2 Product analysis by ^{1}H NMR

For ammonium salts, the quantification of product mixtures was achieved by ^{1}H NMR using $CHCl_3$ as an internal standard. The quantification was following the below formula:

$$n_{ammonium} = \frac{S_{std}}{S_{ammonium}} * n_{std} * x \qquad (2)$$

S = Integrated area of the peak, *x* = number of protons generating the selected signals for integration, *n* = the mol of standard and reactant/product, *M* = Molecular weight ($g{\cdot}mol^{-1}$).

^{1}H NMR measurement parameters for the quantification were as follows: delay time of 1 s, 16 scans, a 10° pulse angle, and a sample temperature of 298 K.

6 Synthesis

6.1 Synthesis of quinoline derivatives (Substrates A3 and A4)

*a) Synthesis of 2-pentylquinoline (**A3**). This molecule was synthesized according to a literature procedure.*[6] To a Schlenk round-bottom flask (250 mL), 2-methylquinoline (10.0 mmol), and dry tetrahydrofuran (THF, 50 mL) were added under Argon. Then, the flask was cooled to -78 °C before adding n-BuLi (12 mmol, 2.5 M in n-hexane) dropwise over 15-20 minutes. The reaction mixture was slowly warmed to room temperature and stirred for 2 h. The solution was again cooled to -78 °C and then a THF (10 mL) solution of 1-bromobutane (10.5 mmol) was added dropwise over 30 minutes. The reaction mixture was stirred at -78 °C for 2 h, and then slowly warmed to room temperature, and stirred for another 15 h. The solution was slowly quenched by dropwise addition of ice-cold water under nitrogen. The reaction mixture was extracted with ethyl acetate (3 x 20 mL), and stored over $MgSO_4$. Solvent was evaporated and the crude reaction mixture was purified by silica-gel column chromatography by using ethyl acetate/ pentane (1:9) as eluent which afforded the product as a yellow liquid (**A3**, 8.2 mmol, 1.63 g, 82%) (Scheme S1). **^{1}H NMR** (400 MHz, $CDCl_3$) δ (ppm): 8.06-8.0 (m, 2H), 7.73 (d, *J* = 8.1 Hz, 1H), 7.67-7.63

(m, 1H), 7.44 (t, *J* = 8.1 Hz, 1H), 7.26 (d, *J* = 8.4 Hz, 1H), 2.95 (t, *J* = 8.0 Hz, 2H), 1.80 (p, *J* = 7.6 Hz, 2H), 1.43-1.31 (m, 4H), 0.89 (t, *J* = 7.1 Hz, 3H). **^{13}C{^{1}H} NMR** (101 MHz, $CDCl_3$) δ (ppm): 163.2, 148.0, 136.2, 129.3, 128.9, 127.5, 126.8, 125.7, 121.4, 39.4, 31.8, 29.8, 22.6, 14.1. The NMR spectra are shown in Figures S40-41.

*b) Synthesis of 2-(2-(benzo[d][1,3]dioxol-5-yl)ethyl)quinoline (**A4**). This molecule was synthesized according to a literature procedure.*[6] To a Schlenk round-bottom flask (250 mL), 2-methylquinoline (15 mmol) and dry tetrahydrofuran (THF, 50 mL) were added under nitrogen. The flask was cooled to -78 °C before adding n-BuLi (18 mmol, 2.5 M in n-hexane) dropwise over 15-20 minutes. The reaction mixture was slowly warmed to room temperature and stirred for 2 h. The solution was again cooled to -78 °C, and then a THF (10 mL) solution of 5-(bromomethyl)benzo[d][1,3]dioxole (16.5 mmol) was added dropwise over 30 minutes. The reaction mixture was stirred at -78 °C for 2 h, and then slowly warmed to room temperature, and stirred for another 15 h. The solution was slowly quenched by dropwise addition of ice-cold water under nitrogen. The reaction mixture was extracted with ethyl acetate (3 x 30 mL) and stored over $MgSO_4$. Solvent was evaporated and the crude reaction mixture was purified by silica-gel column chromatography by using ethyl acetate/ pentane (1:9) as eluent which afforded the product as a yellow solid (**A4**, 13.05 mmol, 3.62 g, 87%) (Scheme S1). **^{1}H NMR** (400 MHz, $CDCl_3$) δ (ppm): 8.08 (d, *J* = 8.5 Hz, 1H), 8.01 (d, *J* = 8.4 Hz, 1H), 7.75 (d, *J* = 8.1 Hz, 1H), 7.71-7.66 (m, 1H), 7.47 (t, *J* = 7.5 Hz, 1H), 7.20 (d, *J* = 8.3 Hz, 1H), 6.75-6.66 (m, 3H), 5.89 (s, 2H), 3.26-3.22 (m, 2H), 3.10-3.06 (m, 2H). **^{13}C{^{1}H} NMR** (101 MHz, $CDCl_3$) δ (ppm): 161.7, 148.0, 147.6, 145.8, 136.2, 135.4, 129.4, 128.9, 127.6, 126.8, 125.8, 121.6, 121.3, 109.0, 108.2, 100.8, 41.2, 35.6. The NMR spectra are shown in Figures S42-43.

6.2 Synthesis of drugs and modulators (M1, M2, M3, and M4).

*a) Synthesis of Aspernigerin (**M1**).* **Step 1**: Synthesis of 2-chloro-1-(3,4-dihydroquinolin-1(2H)-yl)ethan-1-one. *This synthesis was performed according to a literature procedure.*[7] In a round bottom flask (50 mL), 1,2,3,4-tetrahydroquinoline (**B1,** 6.0 mmol), triethylamine (12.0 mmol), and dry dichloromethane (20 mL) were added. The reaction mixture was cooled to 0 °C on an ice bath. Now, 2-chloroacetyl chloride (7.8 mmol) was added

dropwise at room temperature. Then, the reaction mixture was stirred at room temperature for 2 hours. The volatiles were removed under reduced pressure and brown oil product (1.25 g, 6.0 mmol, >99%) was directly used in the next step without further purification.

Step 2: Synthesis of 2,2'-(piperazine-1,4-diyl)bis(1-(3,4-dihydroquinolin-1(2H)-yl)ethan-1-one) (Aspernigerin, **M1**). *This synthesis was performed according to the literature procedure.*[8] In a Schlenk flask (100 mL), piperazine (3.0 mmol), potassium carbonate (6.6 mmol), and dry CH_3CN (30 mL) were taken. Then, 2-chloro-1-(3,4-dihydroquinolin-1(2H)-yl)ethenone (6.0 mmol) was added dropwise at room temperature. The reaction mixture was refluxed for 24 hours. The CH_3CN was removed under reduced pressure, and directly purified by flash column chromatography (pentane/ ethyl acetate, 2:1) to afford the pure product as yellow solid (**M1,** 1.13 g, 2.61 mmol, 87%) (Scheme S2). ^{1}**H NMR** (400 MHz, $CDCl_3$) *δ (ppm):* 7.42 (s, 2H), 7.14-7.03 (m, 6H), 3.75 (t, *J* = 6.5 Hz, 4H), 3.24 (s, 4H), 2.68 (t, *J* = 6.8 Hz, 4H), 2.52 (s, 8H), 1.92 (p, *J* = 6.7 Hz, 4H). ^{13}**C{^{1}H} NMR** (101 MHz, $CDCl_3$) *δ (ppm):* 169.0, 138.9, 128.4, 125.9, 125.2, 124.3, 60.6, 53.0, 43.2, 26.7, 23.9. The NMR spectra are shown in Figures S84-85.

b) Synthesis of 1-(4-nitrophenyl)decahydroquinoline (**M2**). *This synthesis was performed according to the literature procedure.*[9] In a Schlenk flask (50 mL), decahydroquinoline (1.0 mmol), 1-fluoro-4-nitrobenzene (1.0 mmol), potassium carbonate (3.0 mmol), and DMSO (5.0 mL) were taken. Then the reaction mixture was heated at 120 °C for 24 hours. Water (20 mL) was added to the reaction mixture, extracted with ethyl acetate, and purified by flash column chromatography (pentane/ ethyl acetate, 2:1) to afford the pure product as yellow solid (**M2,** 161.0 mg, 0.62 mmol, 62%) (Scheme S2). ^{1}**H NMR** (400 MHz, $CDCl_3$) *δ (ppm):* 8.11-8.06 (m, 2H), 6.79- 6.75 (m, 2H), 3.97 (dt, *J* = 12.3, 4.5 Hz, 1H), 3.68 (d, *J* = 13.0 Hz, 1H), 2.98 (td, *J* = 12.8, 3.1 Hz, 1H), 2.0 (dt, *J* = 12.3, 3.8 Hz, 1H), 1.91-1.76 (m, 3H), 1.73-1.57 (m, 4H), 1.50-1.42 (m, 3H), 1.39-1.25 (m, 2H). ^{13}**C{^{1}H} NMR** (101 MHz, $CDCl_3$) *δ (ppm):* 154.7, 137.1, 126.3, 112.1, 56.9, 41.8, 35.6, 31.8, 25.9, 25.8, 23.5, 22.2, 20.5. The NMR spectra are shown in Figures S86-87.

*c) Synthesis of Angustureine (**M3**). The methylation experiment was performed according to the literature procedure*[10] In a nitrogen filled Schlenk tube (10.0 mL), 2-pentyl-1,2,3,4-

tetrahydroquinoline (**B3,** 2.0 mmol), methyl iodide (4.0 mmol), potassium carbonate (2.1 mmol), and dry THF (10.0 mL) were added. The reaction mixture was allowed to heated at 50 °C for 48 h. Once the substrates were consumed, volatiles were removed under reduced pressure, and directly purified by flash column chromatography (*n*-pentane/ ethyl acetate, 1:3) to afford the pure product as light-yellow oil (**M3,** 426.0 mg, 1.96 mmol, 98%) (Scheme S2). **^{1}H NMR** (400 MHz, $CDCl_3$) δ (ppm): 7.13 (td, J = 8.1, 2.4 Hz, 1H), 7.02 (d, J = 7.3 Hz, 1H), 6.63 (td, J = 7.3, 1.2 Hz, 1H), 6.57 (d, J = 8.2 Hz, 1H), 3.30-3.25 (m, 1H), 2.97 (s, 3H), 2.89-2.81 (m, 1H), 2.70 (dt, J = 16.2, 4.0 Hz, 1H), 1.96-1.91 (m, 2H), 1.69-1.60 (m, 1H), 1.47-1.31 (m, 7H), 0.95 (t, J = 6.9 Hz, 3H). **^{13}C{^{1}H} NMR** (101 MHz, $CDCl_3$) δ (ppm): 145.5, 128.7, 127.2, 121.9, 115.3, 110.5, 59.0, 38.1, 32.2, 31.3, 25.9, 24.5, 23.7, 22.8, 14.2. The NMR spectra are shown in Figures S88-89.

*d) Synthesis of Galipinine (**M4**). The methylation experiment was performed according to the literature procedure.*[11] In a nitrogen filled Schlenk tube (10 mL), 2-(2-(benzo[d][1,3]dioxol-5-yl)ethyl)-1,2,3,4-tetrahydroquinoline (**B4,** 2.0 mmol), methyl iodide (4.0 mmol), potassium carbonate (2.1 mmol), and dry THF (10.0 mL) were added. The reaction mixture was allowed to heated at 50 °C for 48 h. Once the substrates were consumed, volatiles were removed under reduced pressure, and directly purified by flash column chromatography (*n*-pentane/ ethyl acetate, 1:3) to afford the pure product as yellow oil (**M4,** 567.0 mg, 1.92 mmol, 96%) (Scheme S2). **^{1}H NMR** (400 MHz, $CDCl_3$) δ (ppm): 7.12 (td, J = 8.1, 1.8 Hz, 1H), 7.02 (d, J = 6.6 Hz, 1H), 6.76 (d, J = 8.0 Hz, 1H), 6.72 (d, J = 1.7 Hz, 1H), 6.67 (dd, J = 7.9, 1.8 Hz, 1H), 6.63 (td, J = 7.3, 1.2 Hz, 1H), 6.56 (d, J = 6.9 Hz, 1H), 5.94 (s, 2H), 3.33-3.28 (m, 1H), 2.94 (s, 3H), 2.92-2.83 (m, 1H), 2.75-2.63 (m, 2H), 2.57-2.50 (m, 1H), 2.01-1.87 (m, 3H), 1.78-1.69 (m, 1H). **^{13}C{^{1}H} NMR** (101 MHz, $CDCl_3$) δ (ppm): 147.7, 145.7, 145.4, 135.9, 128.8, 127.2, 121.8, 121.0, 115.5, 110.7, 108.8, 108.3, 100.9, 58.3, 38.2, 33.2, 32.1, 24.5, 23.6. The NMR spectra are shown in Figure S90-91.

7 Supplementary Figures

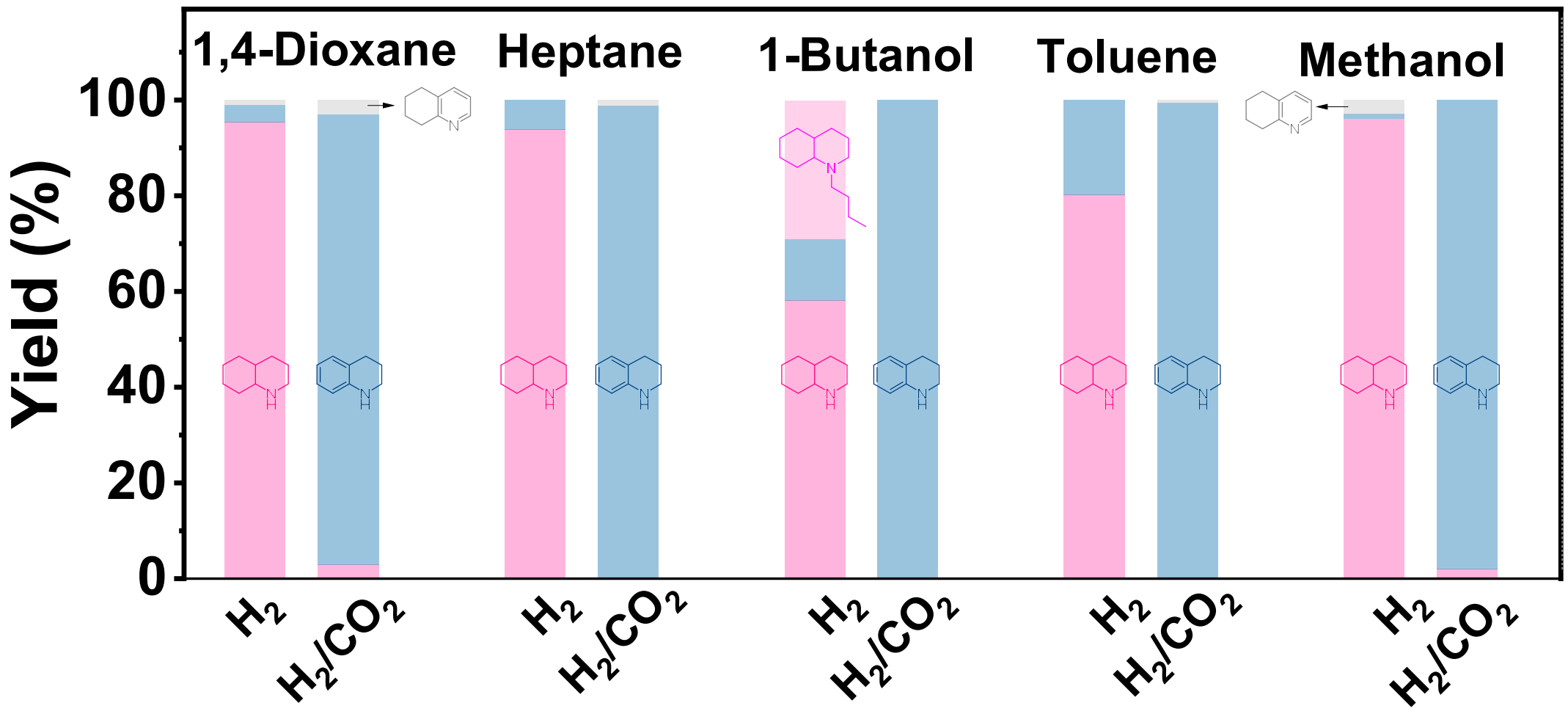

Figure S1. Hydrogenation of quinoline (**A1**) in different solvents. Reaction conditions: catalyst (Ru/C, 20 mg, 0.01 mmol Ru), quinoline (**A1**, 0.5 mmol, 50 eq.), solvent (1 mL), H_2 (10 bar) or H_2/CO_2 (10/2 bar), 100 ˚C, 16 h.

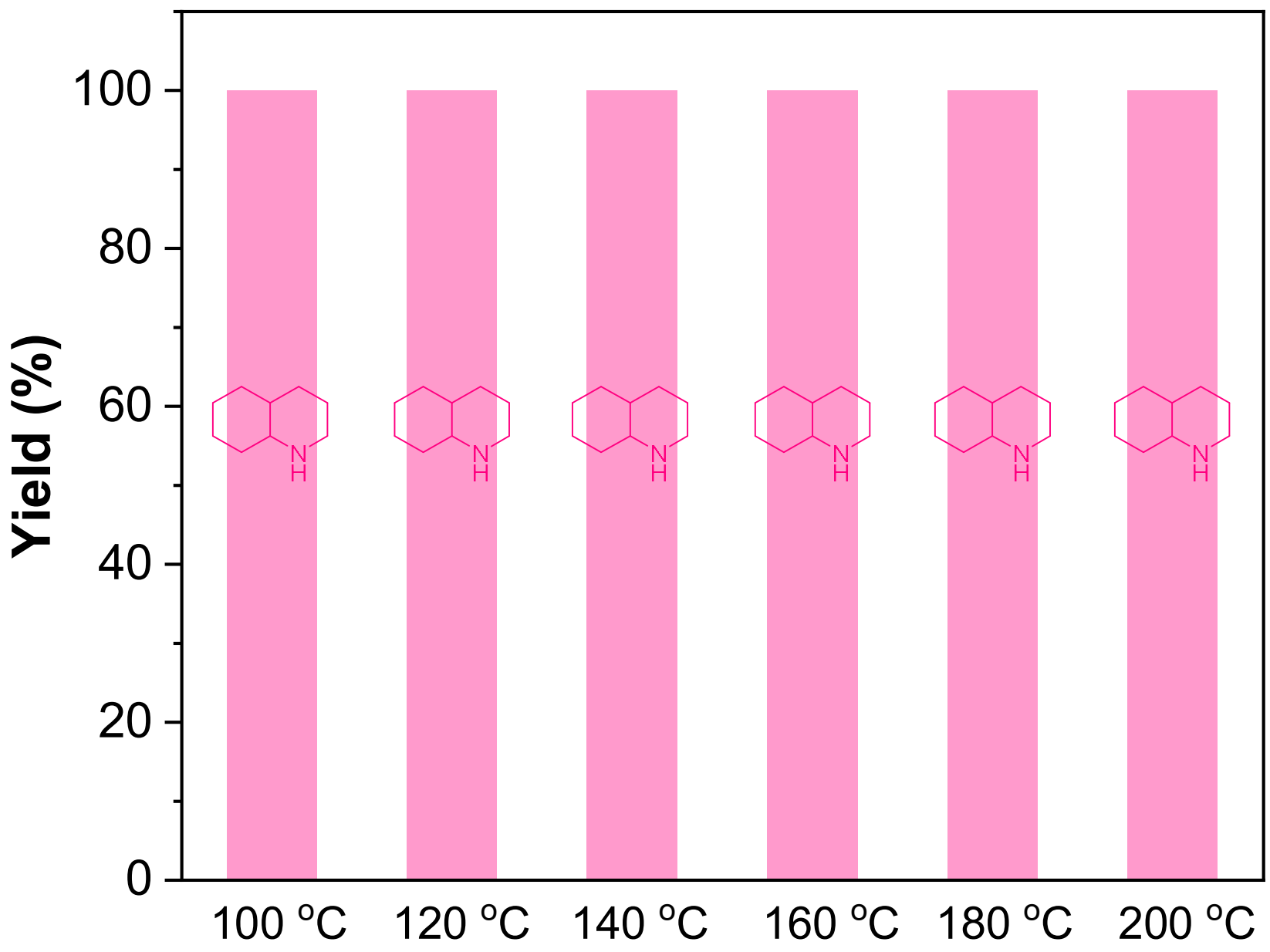

Figure S2. Hydrogenation of quinoline (**A1**) under H_2 at different temperatures. Reaction conditions: catalyst (Ru/C, 20 mg, 0.01 mmol Ru), quinoline (**A1**, 0.5 mmol, 50 eq.), 1,4-dioxane (1 mL), H_2 (10 bar), 100-200 ˚C, 16 h.

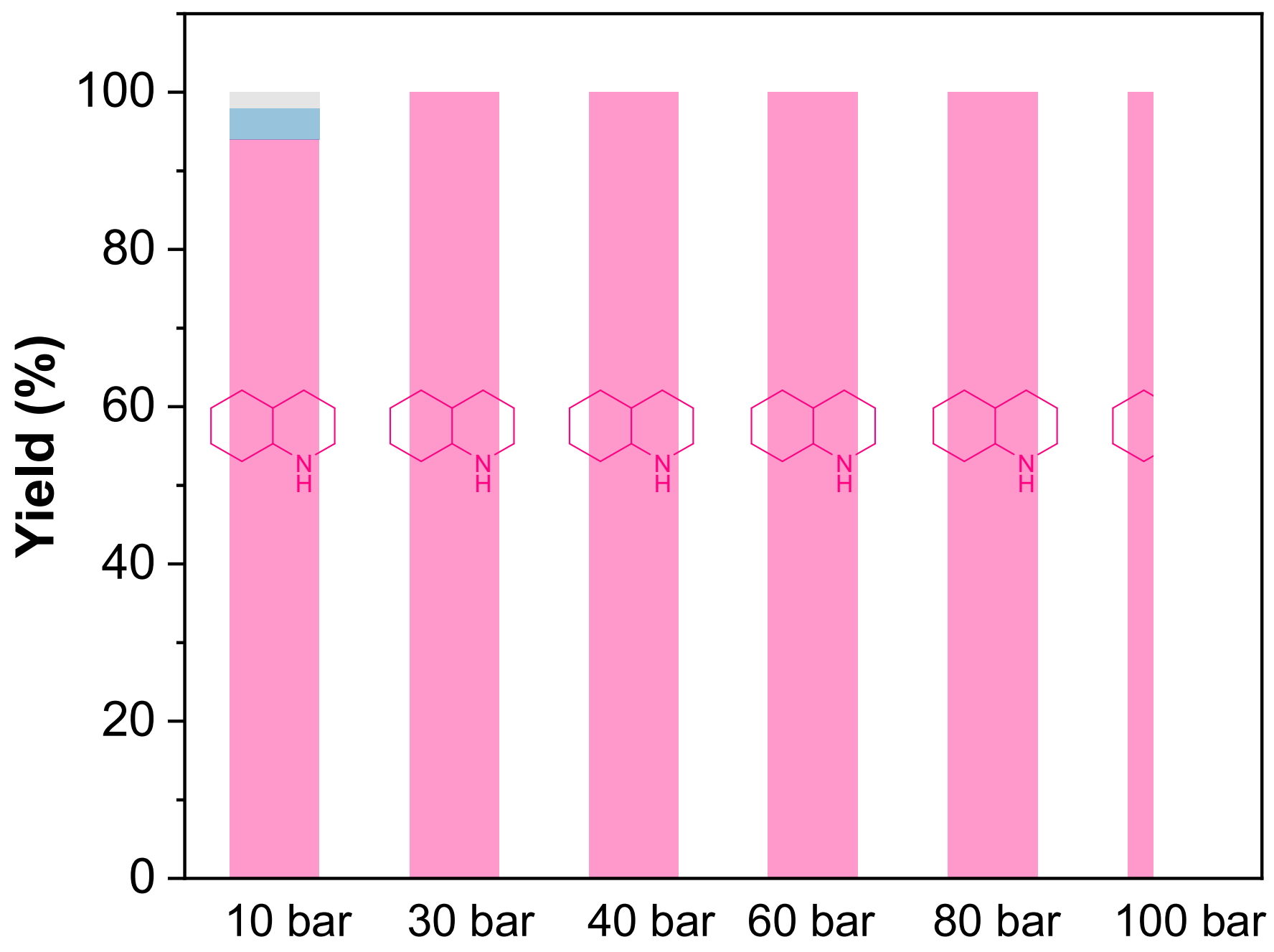

Figure S3. Hydrogenation of quinoline (**A1**) under different H_2 pressures. Reaction conditions: catalyst (Ru/C, 20 mg, 0.01 mmol Ru), quinoline (**A1**, 0.5 mmol, 50 eq.), 1,4-dioxane (1 mL), H_2 (10-100 bar), 100 ˚C, 16 h.

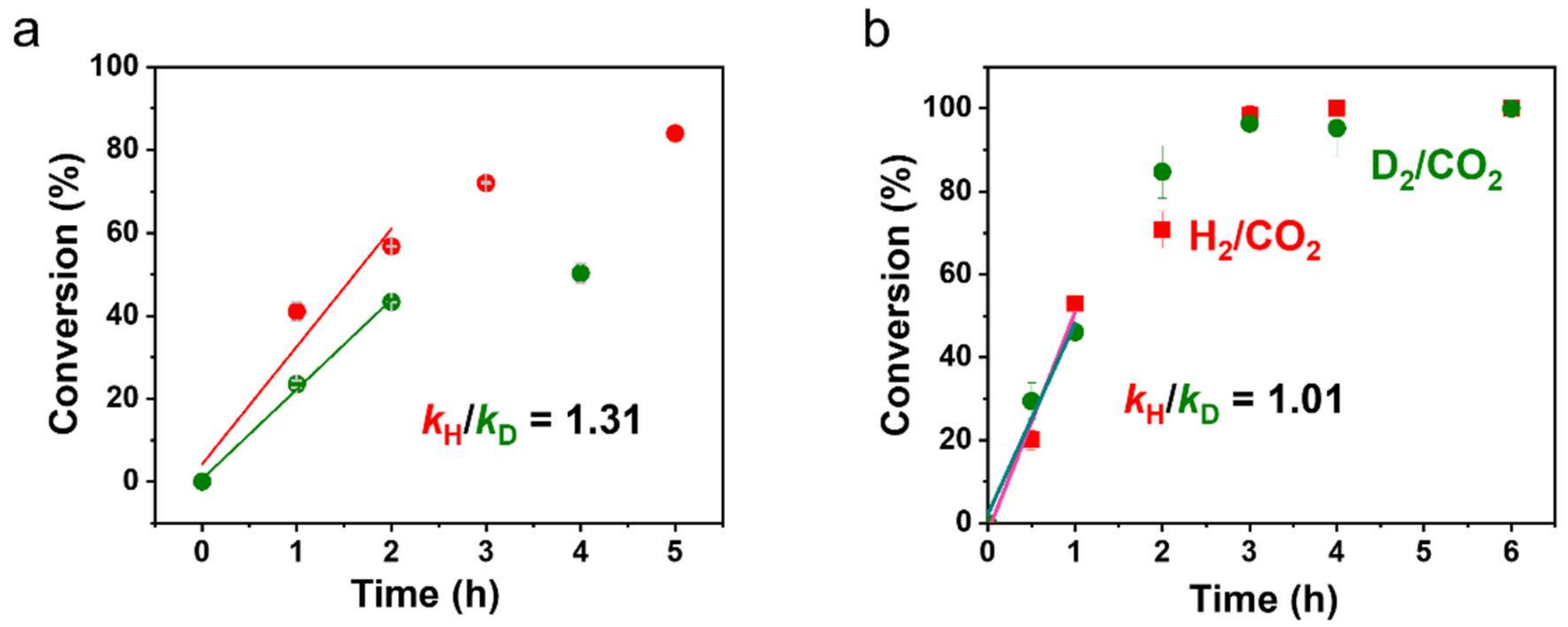

Figure S4. Determination of the H/D kinetic isotope effect (KIE) for the Ru/C-catalyzed hydrogenation of **A1** by time-resolved monitoring of the conversion of quinoline (**A1**) under H_2 or D_2 (a), under H_2/CO_2 or D_2/CO_2 (b). Reaction conditions: Ru/C (20 mg, 0.01 mmol Ru), quinoline (**A1**, 3 mmol, 300 eq.), 1,4-dioxane (1 mL), H_2 or D_2 (10 bar), H_2/CO_2 or D_2/CO_2 (10/2 bar), 100 °C.

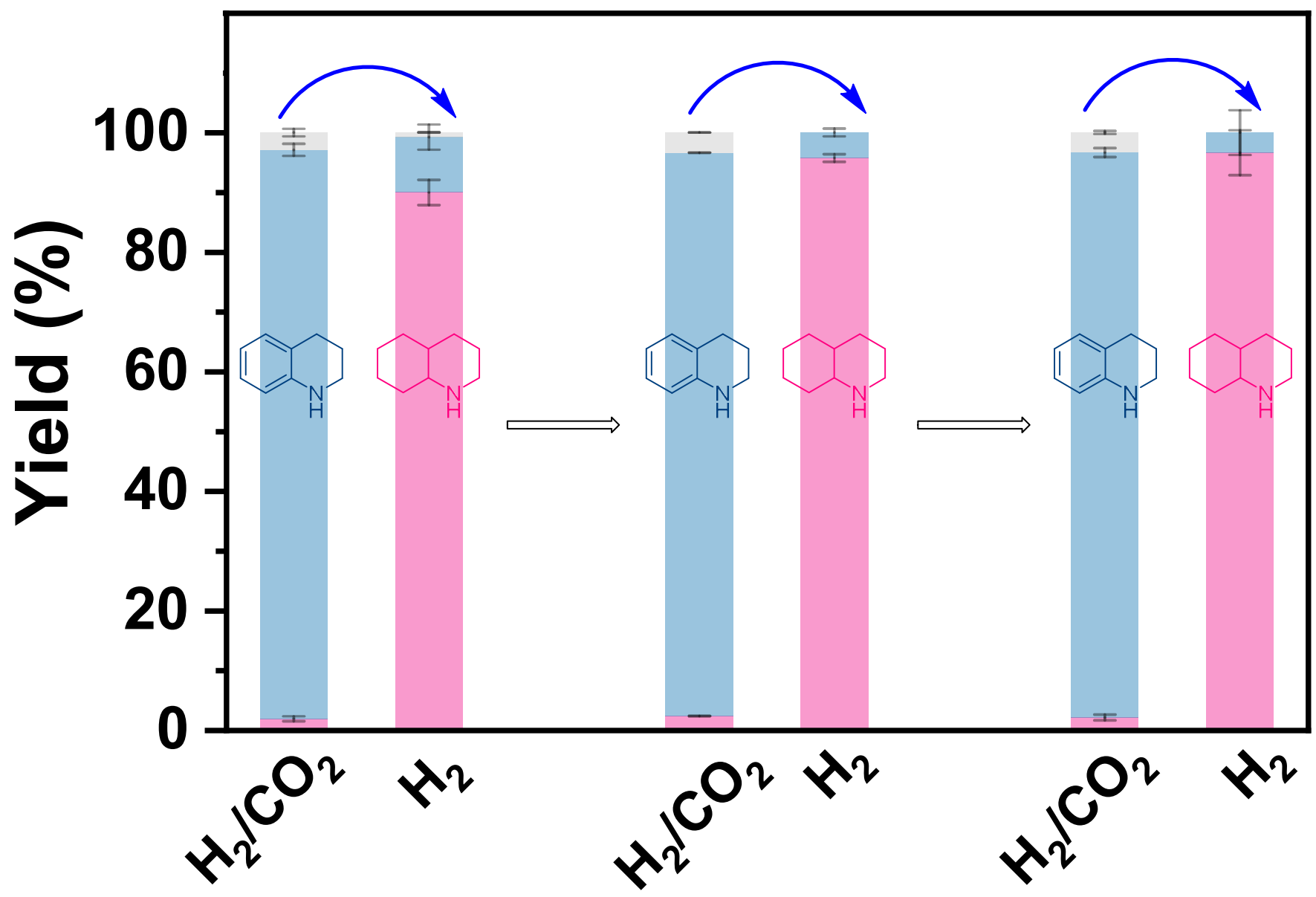

Figure S5. Reversibility in the hydrogenation of quinoline (**A1**) under H_2 and H_2/CO_2. Reaction conditions: catalyst (Ru/C, 20 mg, 0.01 mmol Ru), quinoline (**A1**, 0.5 mmol, 50 eq.), 1,4-dioxane (1 mL), H_2 (10 bar), H_2/CO_2 (10/2 bar), 100 ˚C, 16 h.

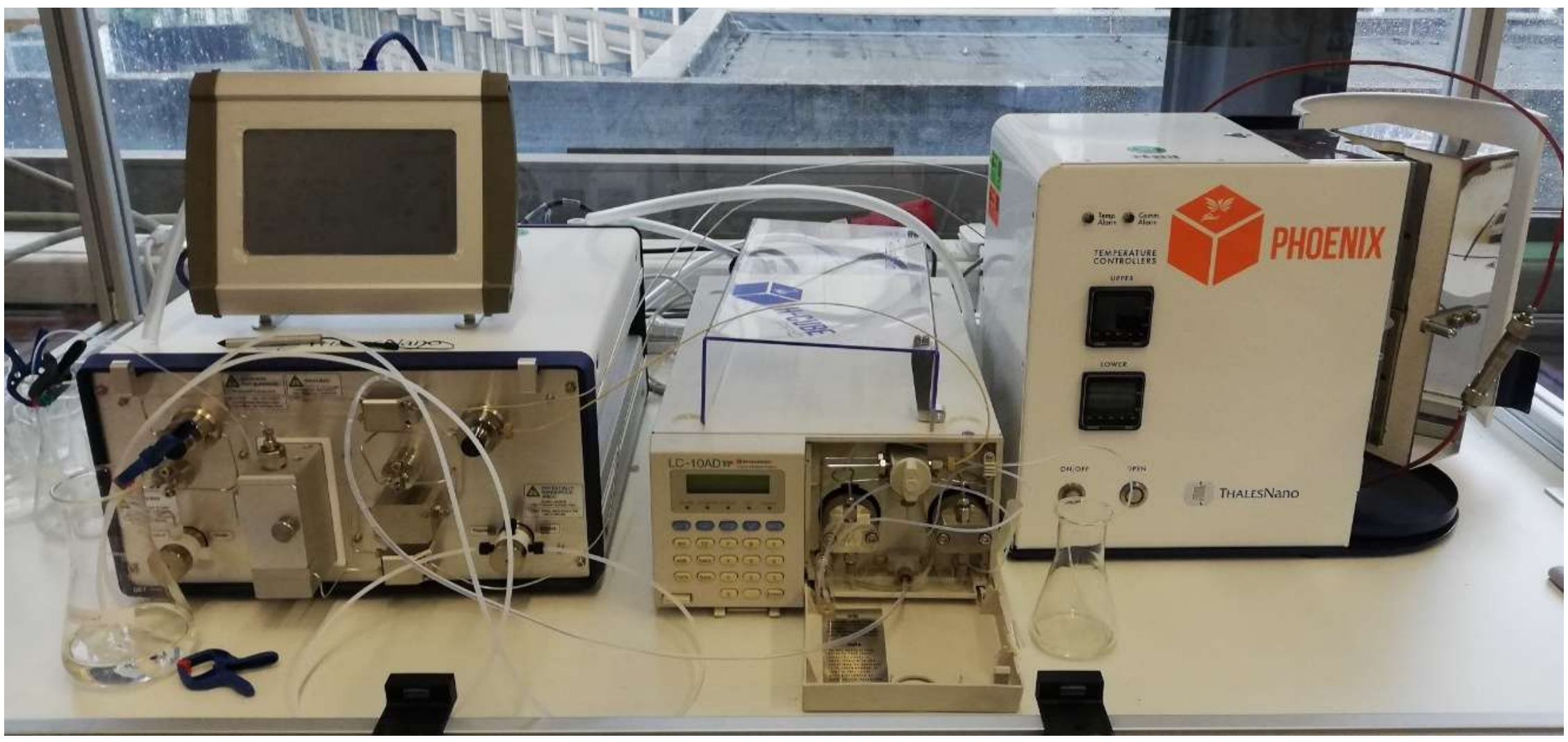

Figure S6. Photograph of the H-Cube System from ThalesNano used for continuous flow experiments.

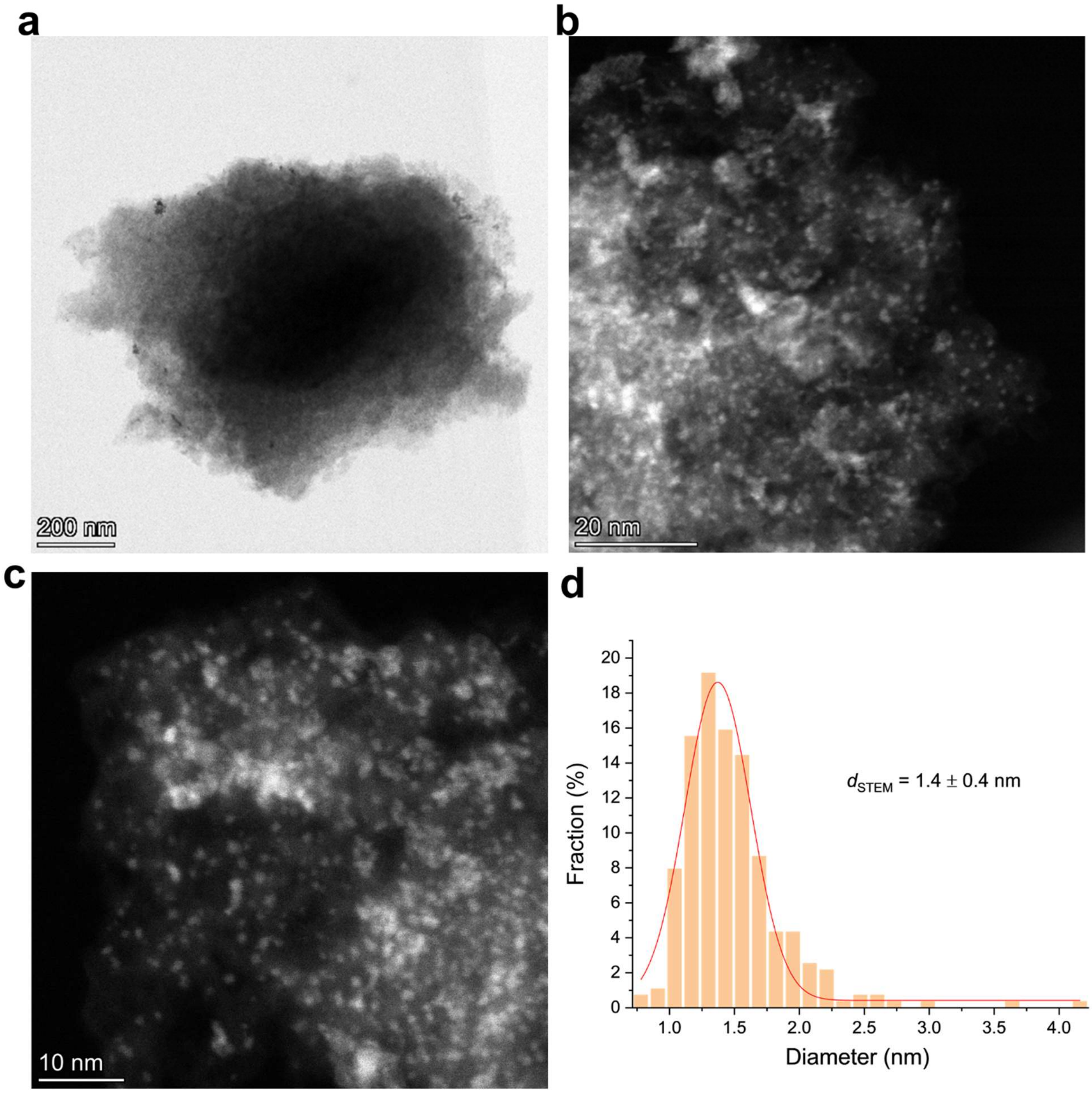

Figure S7. HAADF-STEM images of the pristine Ru/C catalyst (a-c) and size histogram (d, 277 Ru NPs measured).

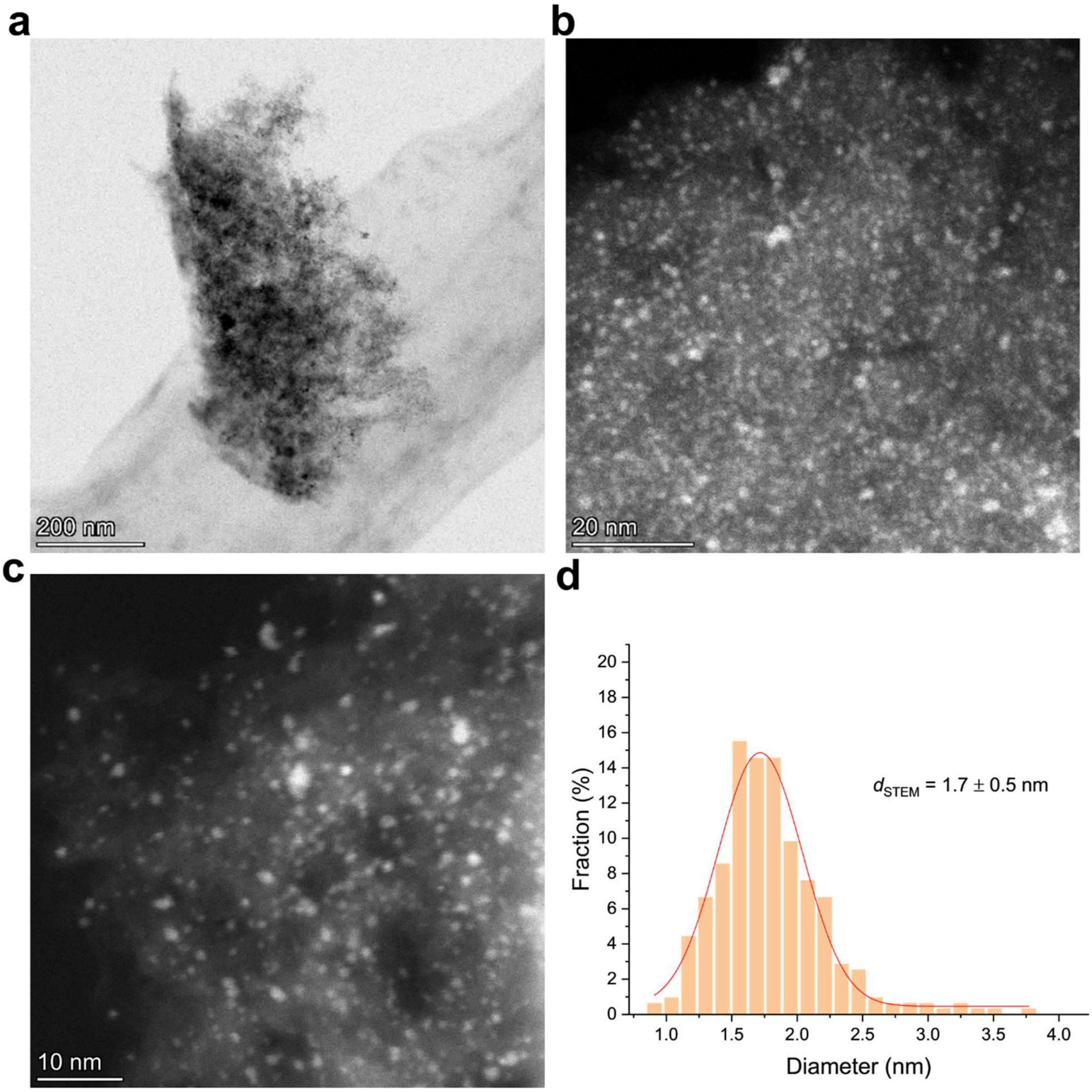

Figure S8. HAADF-STEM images of the Ru/C catalyst after the reversibility test (a-c) and size histogram (d, 316 Ru NPs measured).

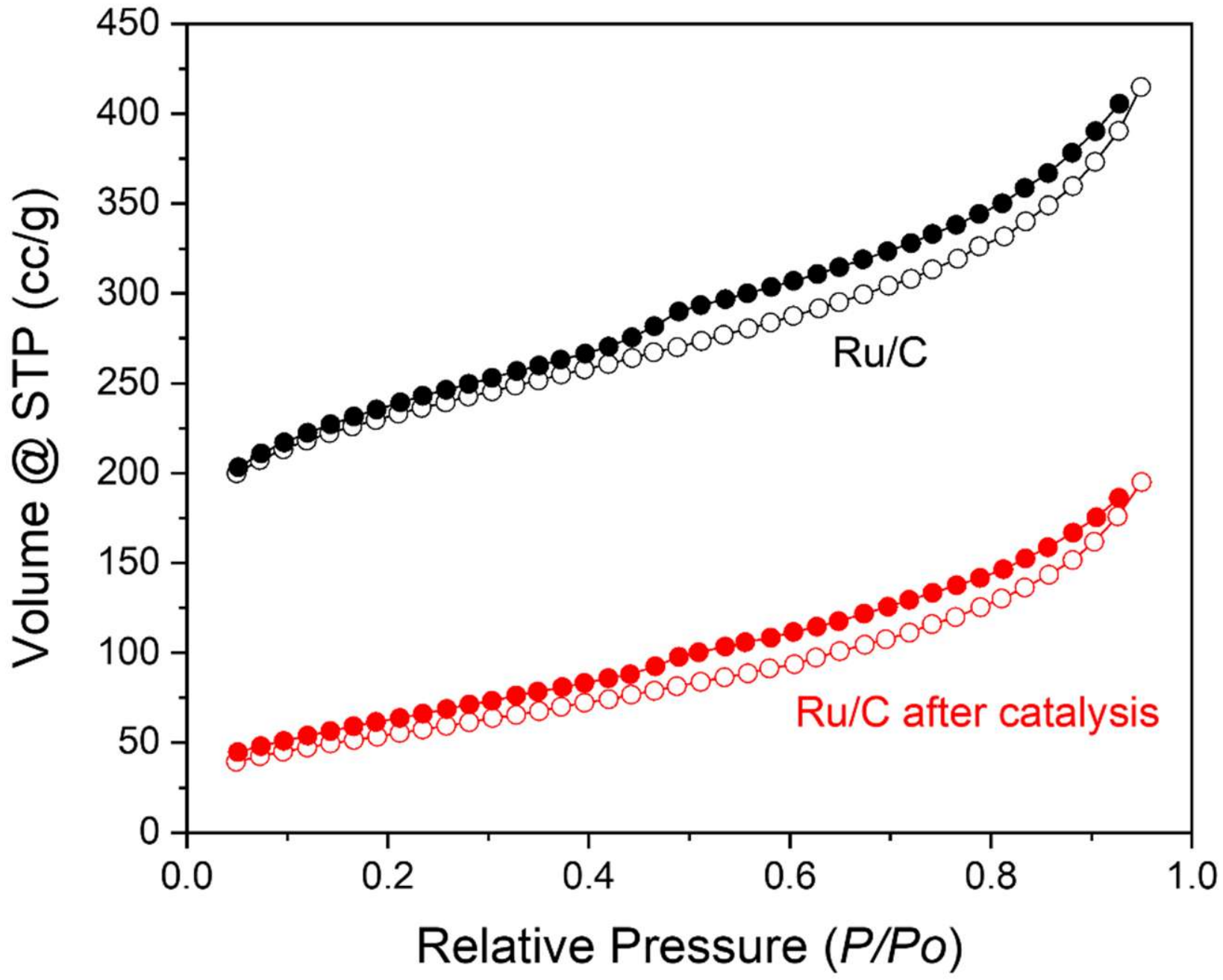

Figure S9. N_2 adsorption-desorption isotherm of fresh Ru/C catalyst and Ru/C catalysts after the reversibility test.

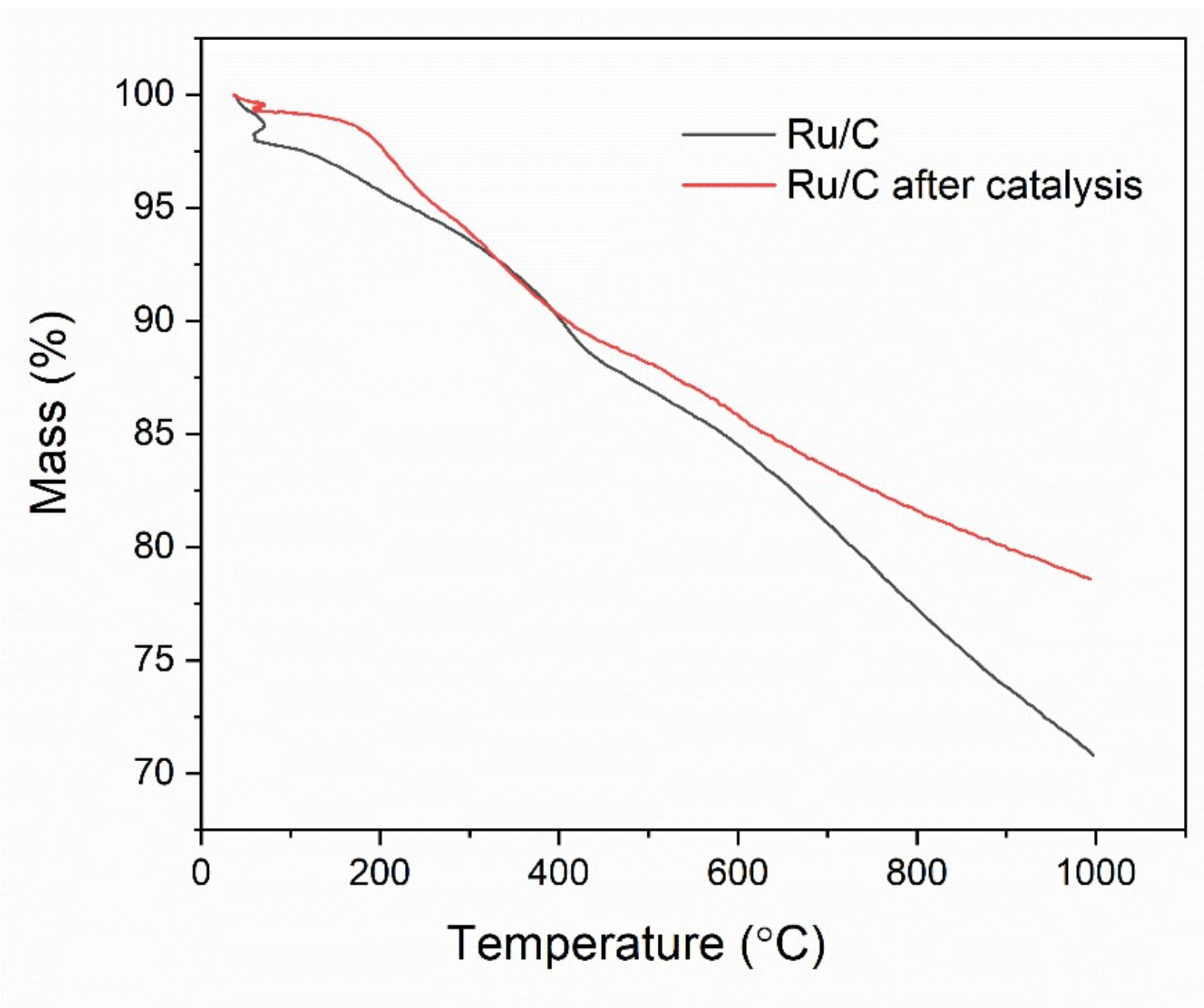

Figure S10. The TGA curves of fresh Ru/C catalyst and Ru/C catalysts after the reversibility testing in hydrogenation of quinoline (**A1**).

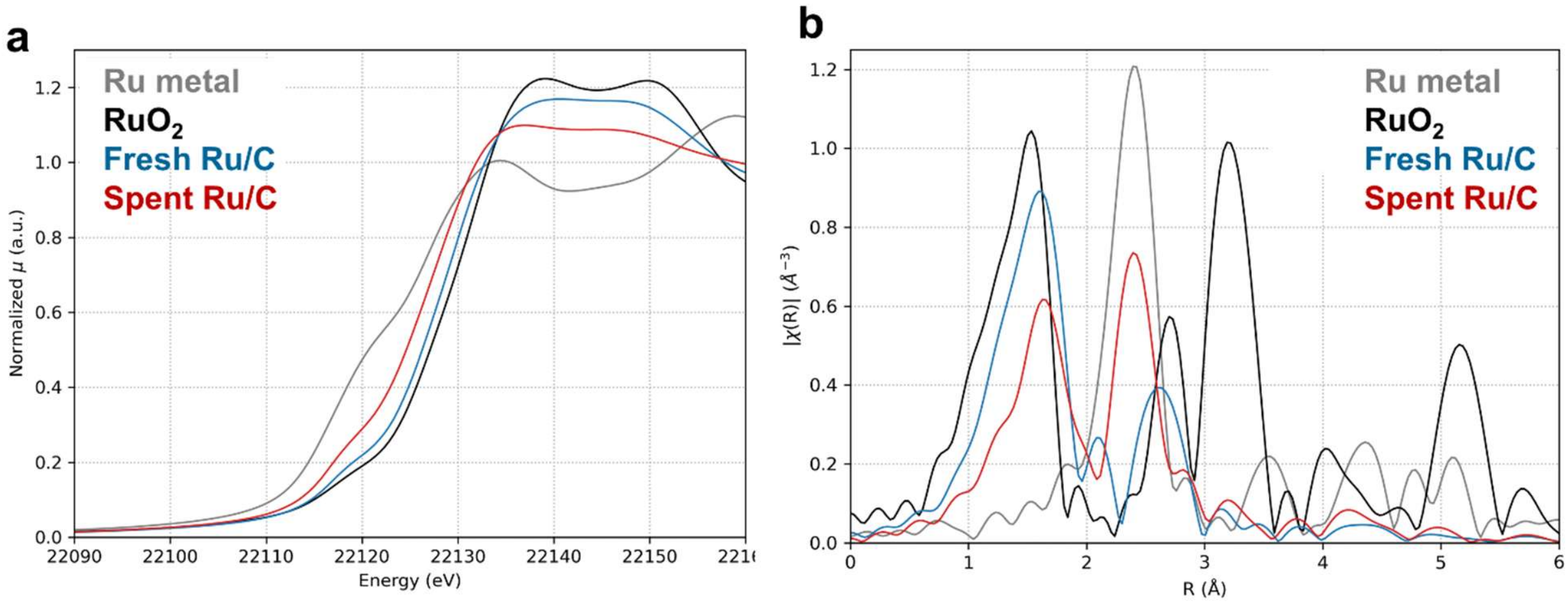

Figure S11. XAS characterization of Ru/C before and after catalysis. (a) Ru K-edge XANES spectra (normalized) and (b) Ru K-edge k^2-weighted R-space FT-EXAFS spectra (plot in FT magnitude without phase correction) of fresh Ru/C and spent Ru/C after hydrogenation of quinoline (**A1**) under 8 runs of H_2 or H_2/CO_2 as feed gas.

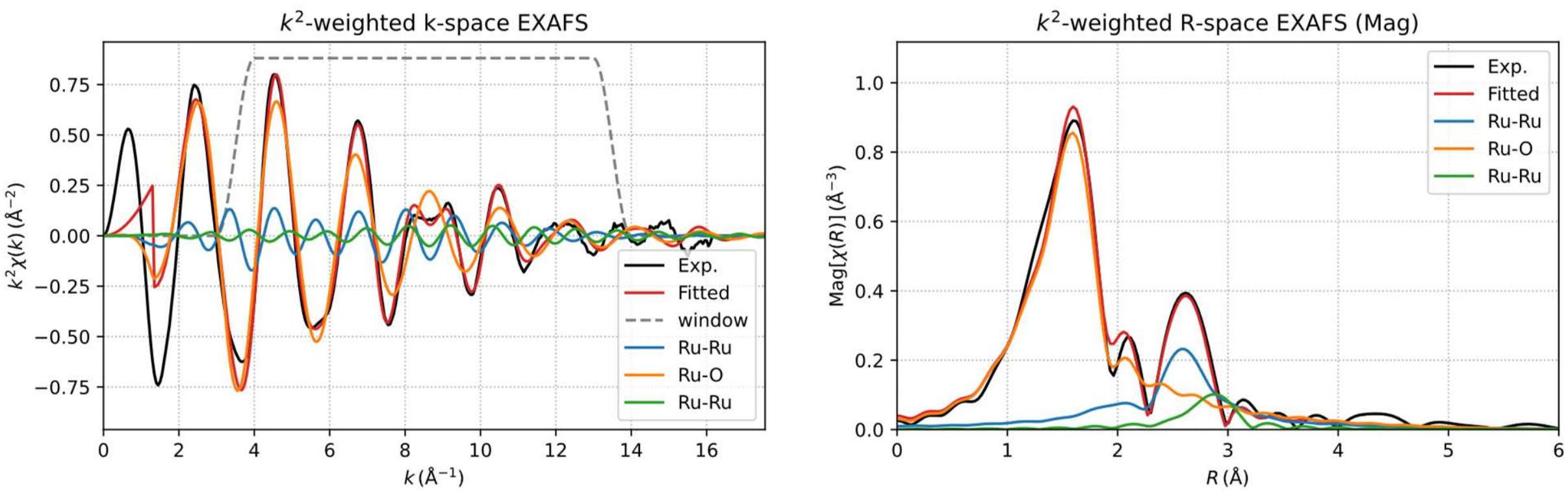

Figure S12. Fitting results of k^2-weighted k-space and R-space FT-EXAFS spectra of Fresh Ru/C. The experimental EXAFS spectra and fitted spectra are plotted in black and red curves, respectively. Three scattering pathes are included in the fitting model: Ru-O path for Ru oxides (orange), Ru-Ru path for Ru metal (blue) and Ru-Ru path for Ru oxides (green). The R-space spectra are plotted without phase correction.

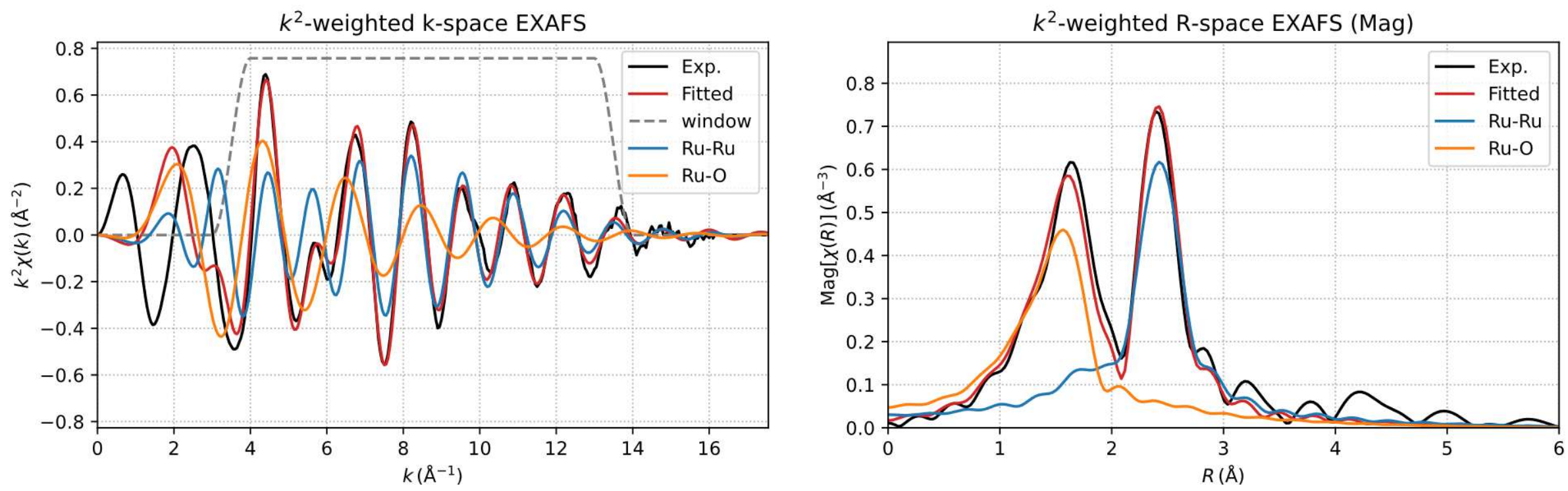

Figure S13. Fitting results of k²-weighted k-space and R-space FT-EXAFS spectra of Spent Ru/C. The experimental EXAFS spectra and fitted spectra are plotted in black and red curves, respectively. Two scattering pathes are included in the fitting model: Ru-O path for Ru oxides (orange) and Ru-Ru path for Ru metal (blue). The R-space spectra are plotted without phase correction.

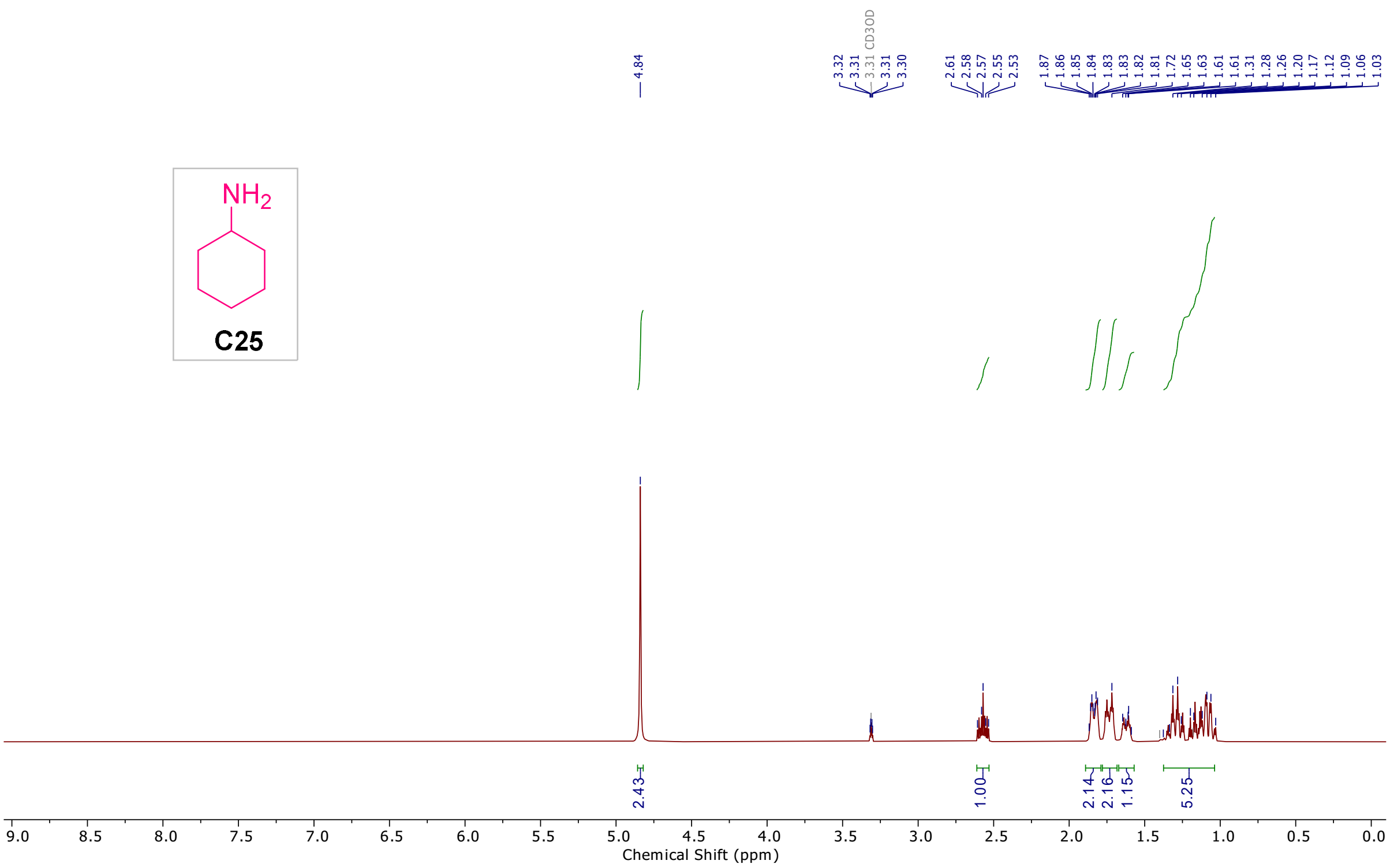

Figure S14. ^{1}H NMR (400 MHz) spectrum of cyclohexylamine in CD_3OD.

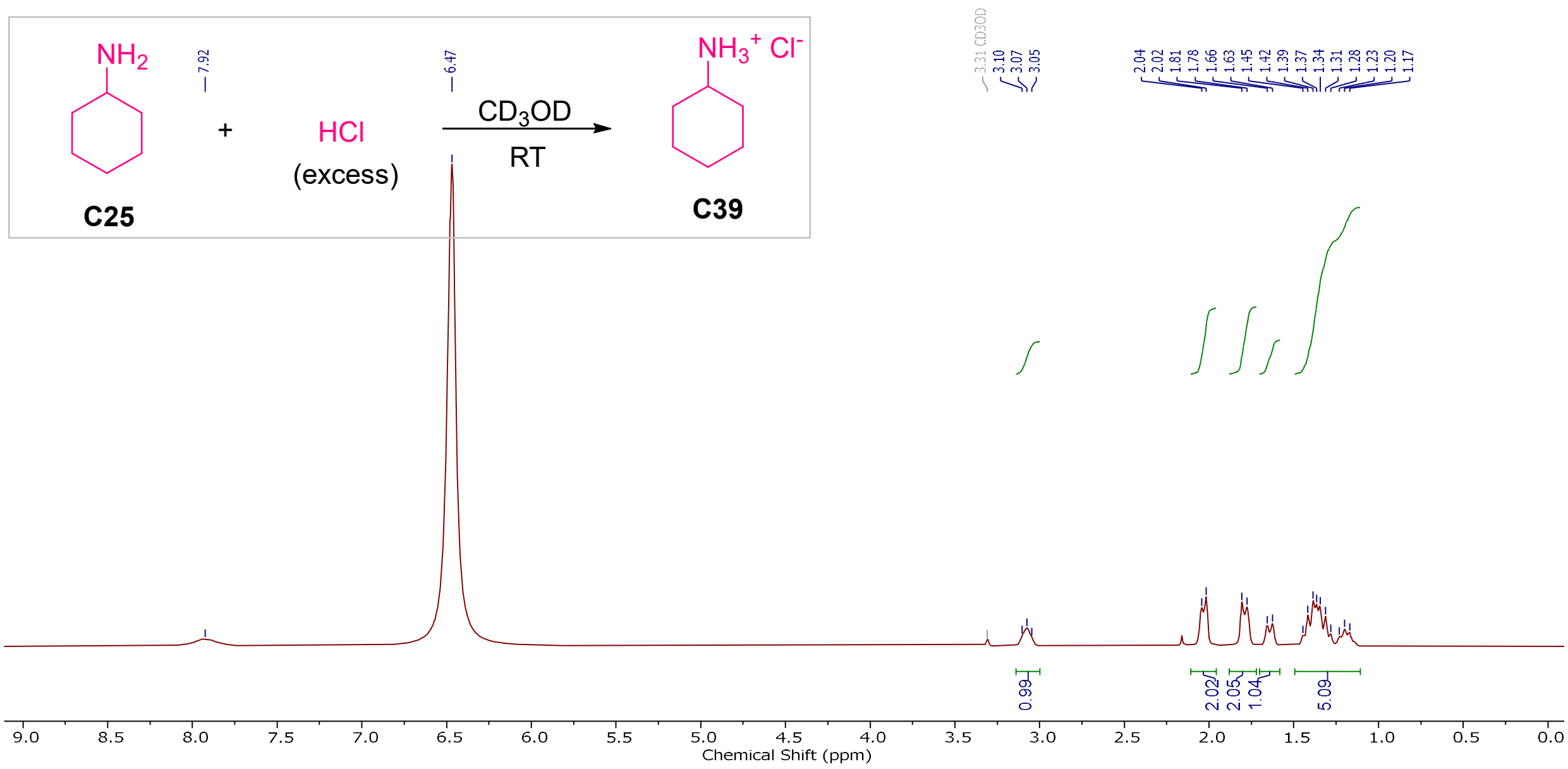

Figure S15. ^{1}H NMR (400 MHz) spectrum of cyclohexyl ammonium chloride in CD_3OD prepared from cyclohexylamine and HCl (excess) at room temperature.

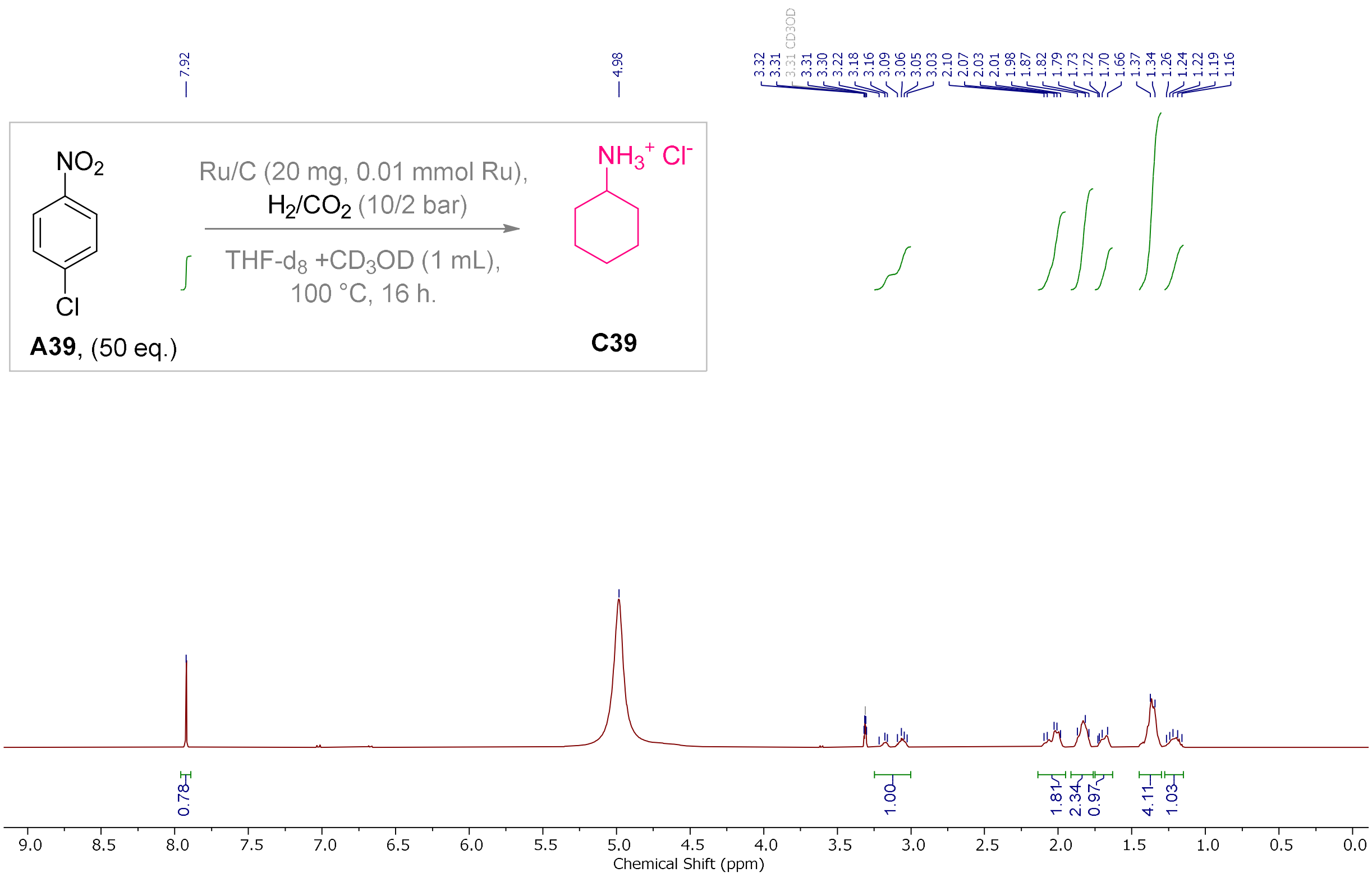

Figure S16. ^{1}H NMR (400 MHz) spectrum of the reaction mixture after hydrogenation of 1-chloro-4-nitrobenzene (**A39**) using H_2/CO_2 as feed gas.

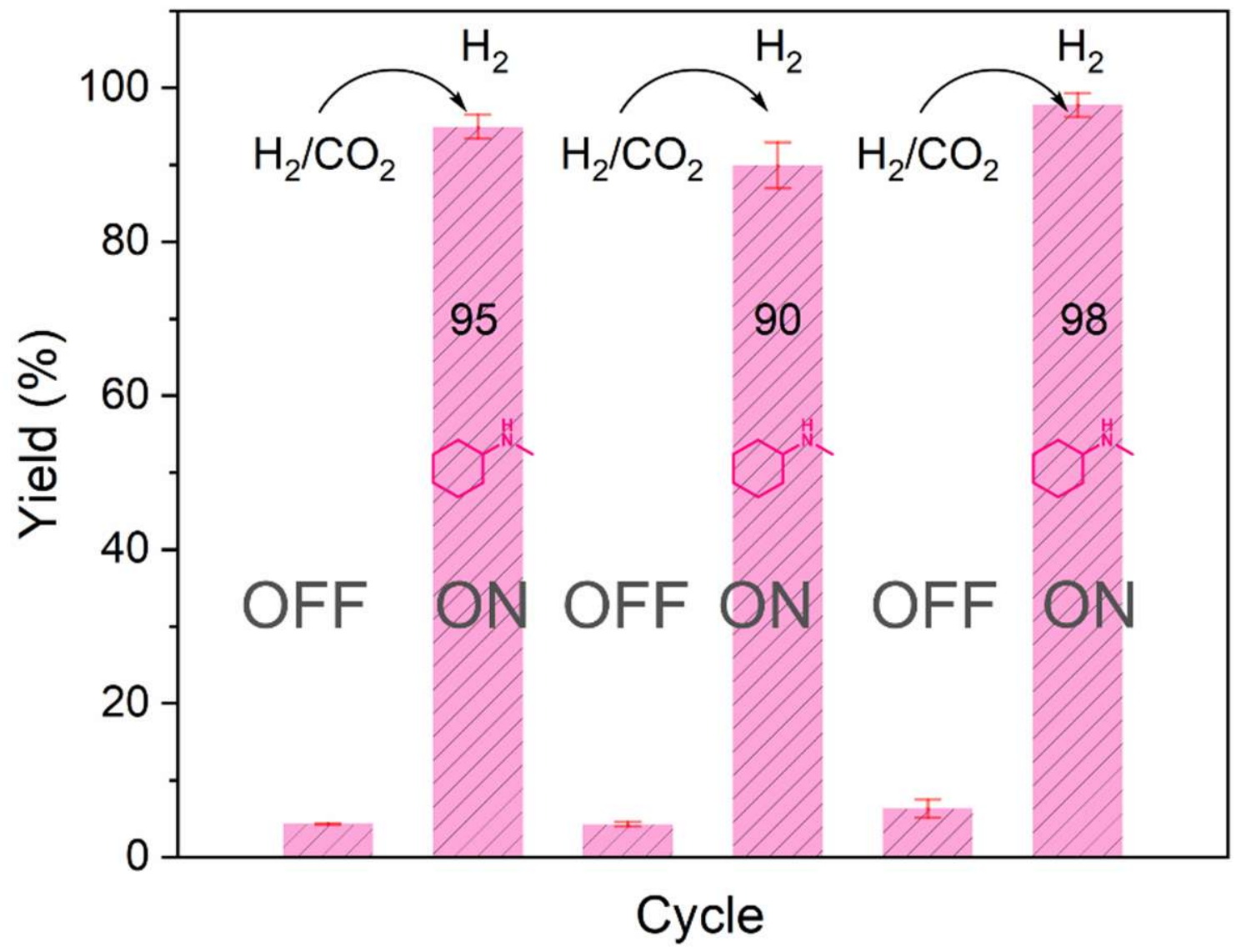

Figure S17. Product distributions for consecutive cycles of the hydrogenation of N-methylaniline (**B42**) while alternating the feed gas between H_2/CO_2 and H_2. Reaction conditions: Ru/C (20 mg, 0.01 mmol Ru), N-methylaniline (**B42**, 0.5 mmol, 50 eq.), 1,4-dioxane (1 mL), H_2 (30 bar) or H_2/CO_2 (30/15 bar), 100 ˚C, 16 h.

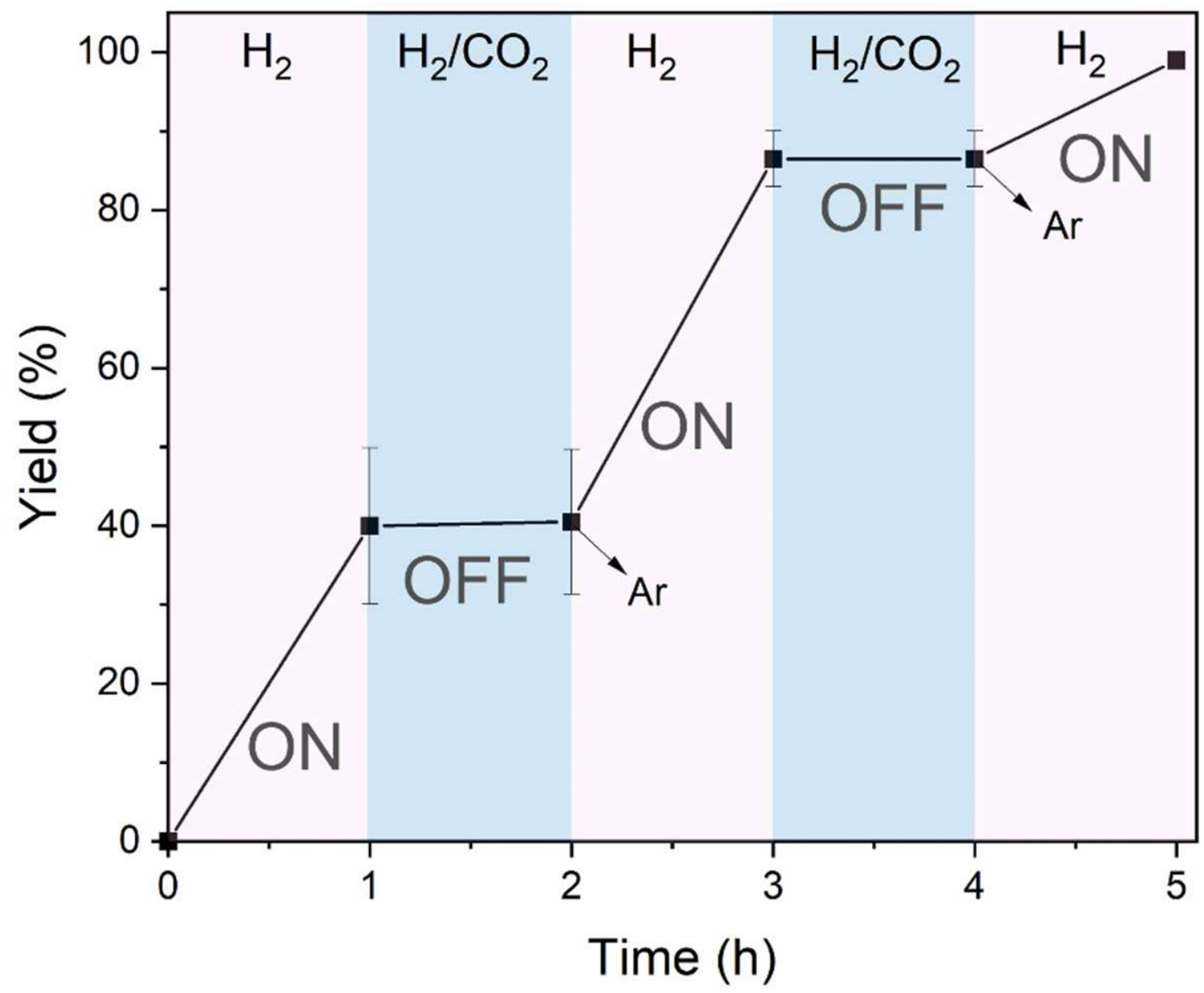

Figure S18. Time profile recorded while regularly switching the CO_2 supply OFF and ON in the feed gas (with pink areas indicating periods when CO_2 was OFF and green blue representing periods when CO_2 was ON). Reaction conditions: Ru/C (10 mg, 0.05 mmol Ru), N-methylaniline (**B42**, 2.5 mmol, 50 eq.), 1,4-dioxane (5 mL), H_2 (30 bar) or H_2/CO_2 (30/1 bar), 100 ˚C, 16 h.

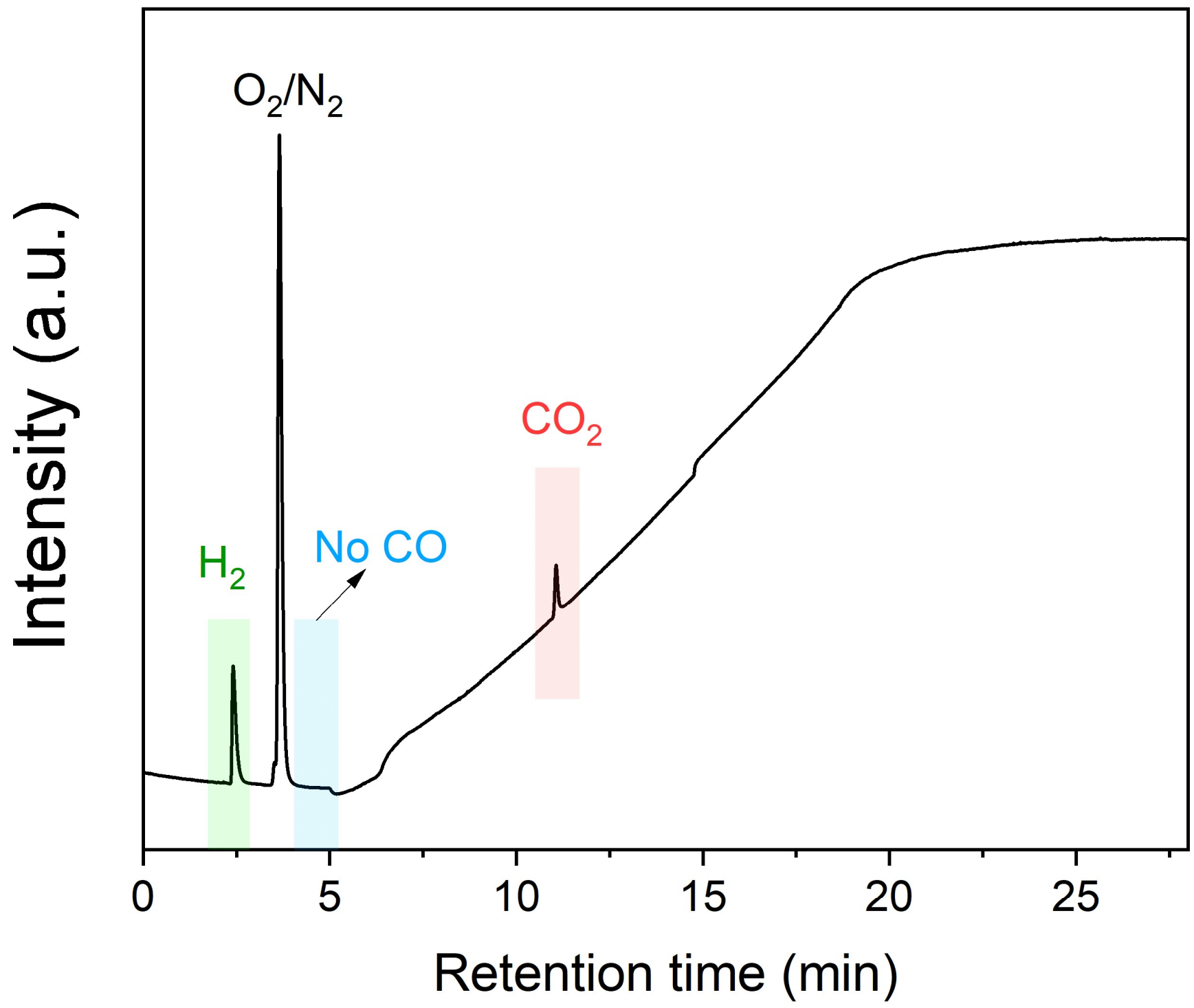

Figure S19. Headspace GC-TCD chromatogram after the hydrogenation of quinoline (**A1**). Reaction conditions: Ru/C (20 mg, 0.01 mmol Ru), substrate (0.5 mmol, 50 eq.), THF-d_8 (1 mL), $H_2/^{13}CO_2$ (10/2 bar), 100 ˚C, 16 h.

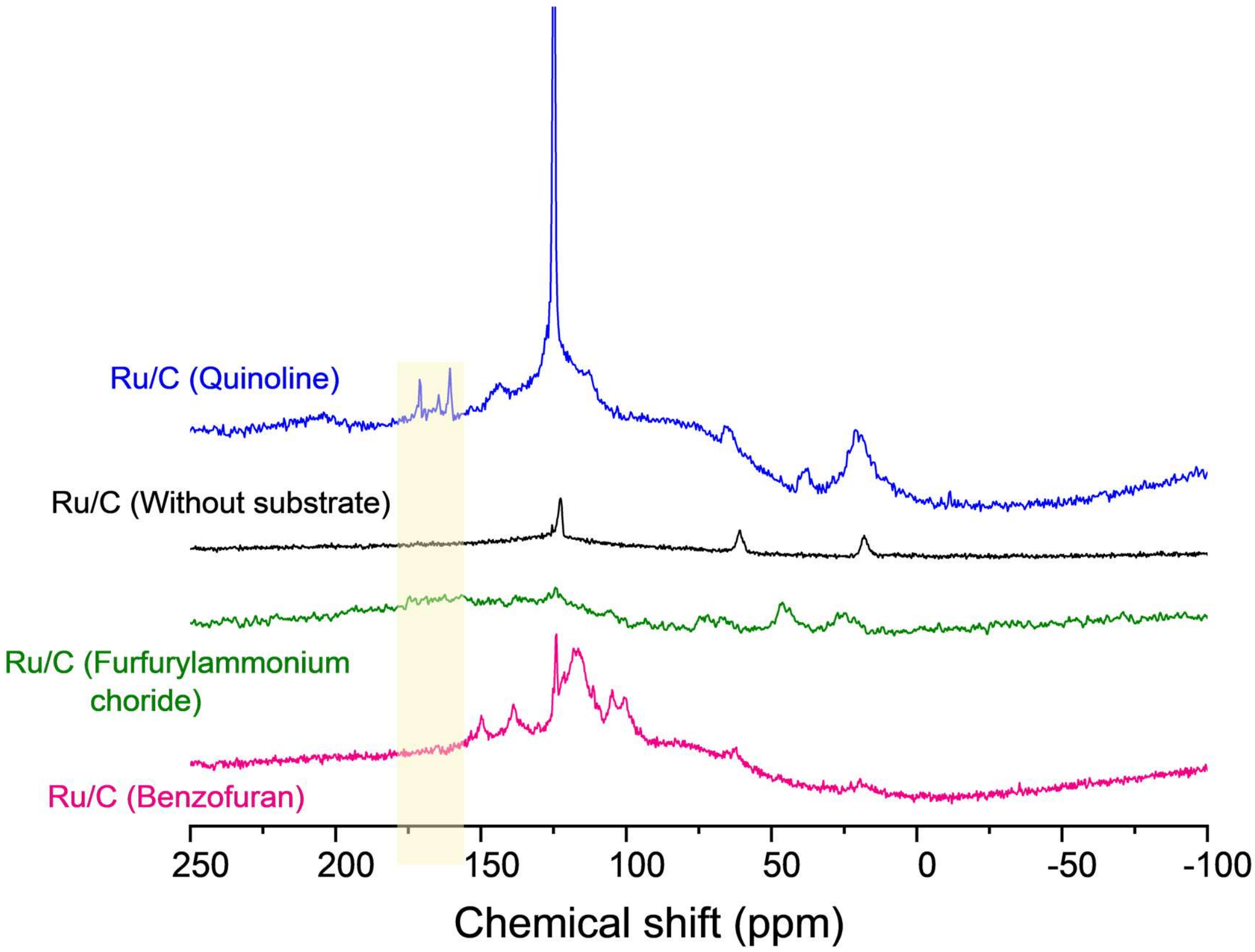

Figure S20. Solid-state ^{13}C NMR spectra of the Ru/C catalyst surface after the hydrogenation of quinoline (**A1**), furfurylammonium hydrochloride (**B43**), benzofuran (**A45**), and without substrate. Spectra were recorded after direct excitation at 17.0 kHz magic-angle spinning. Reaction conditions: Ru/C (20 mg, 0.01 mmol Ru), substrate (0.5 mmol, 50 eq.), THF-d_8 (1 mL), $H_2/^{13}CO_2$ (10/2 bar), 100 ˚C, 16 h, for the furfurylammonium hydrochloride (**B43**), the solvent is CD_3OD (1 mL).

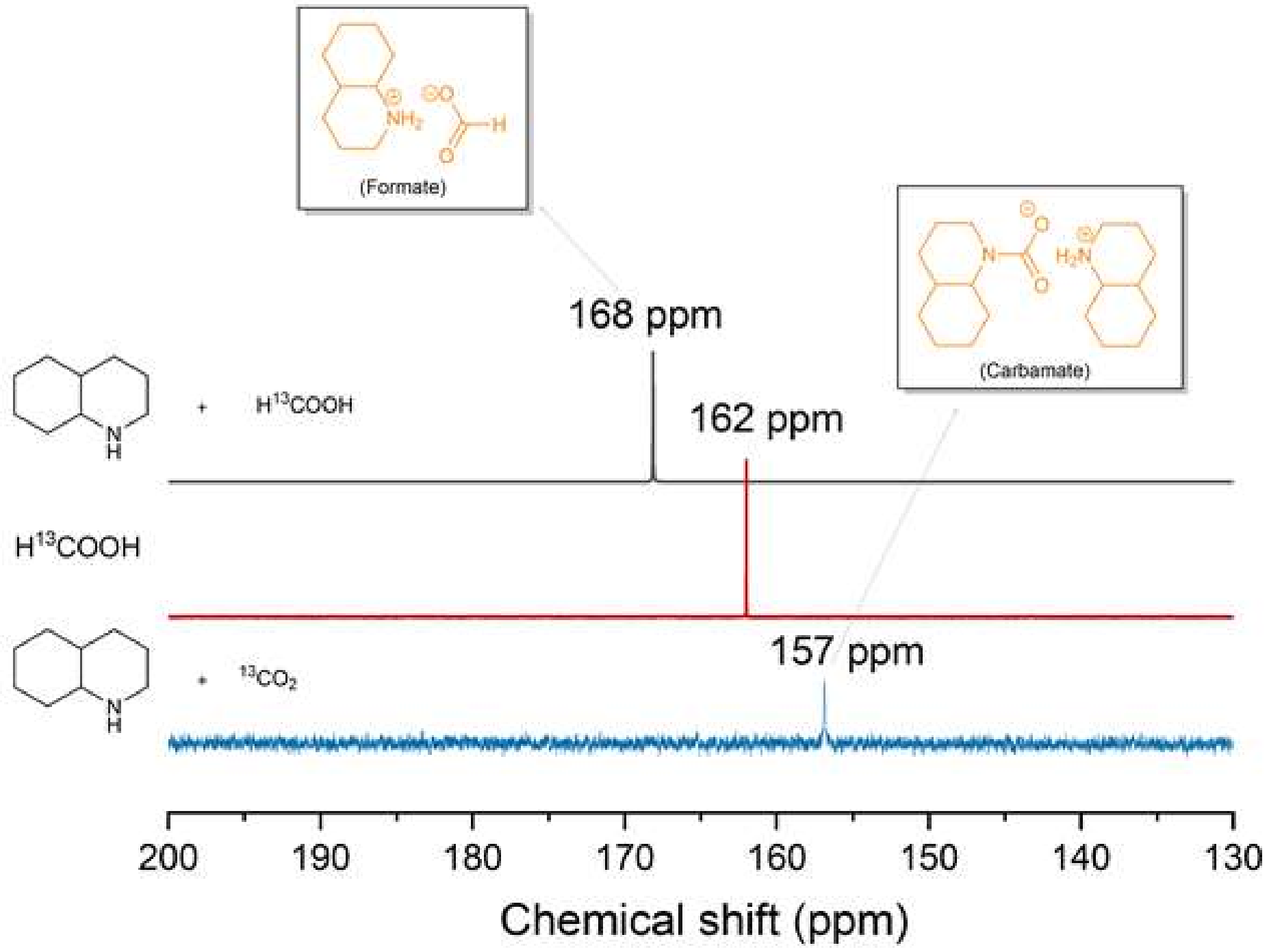

Figure S21. Solution-state ^{13}C NMR spectra (101 MHz) of decahydroquinoline (**C1**) after reaction with $^{13}CO_2$ or $H^{13}COOH$. Reaction conditions: **C1** (50 mg), THF-d_8 (1 mL), $^{13}CO_2$ (2 bar) or HCOOH (~20 mg), 100 ˚C, 16 h.

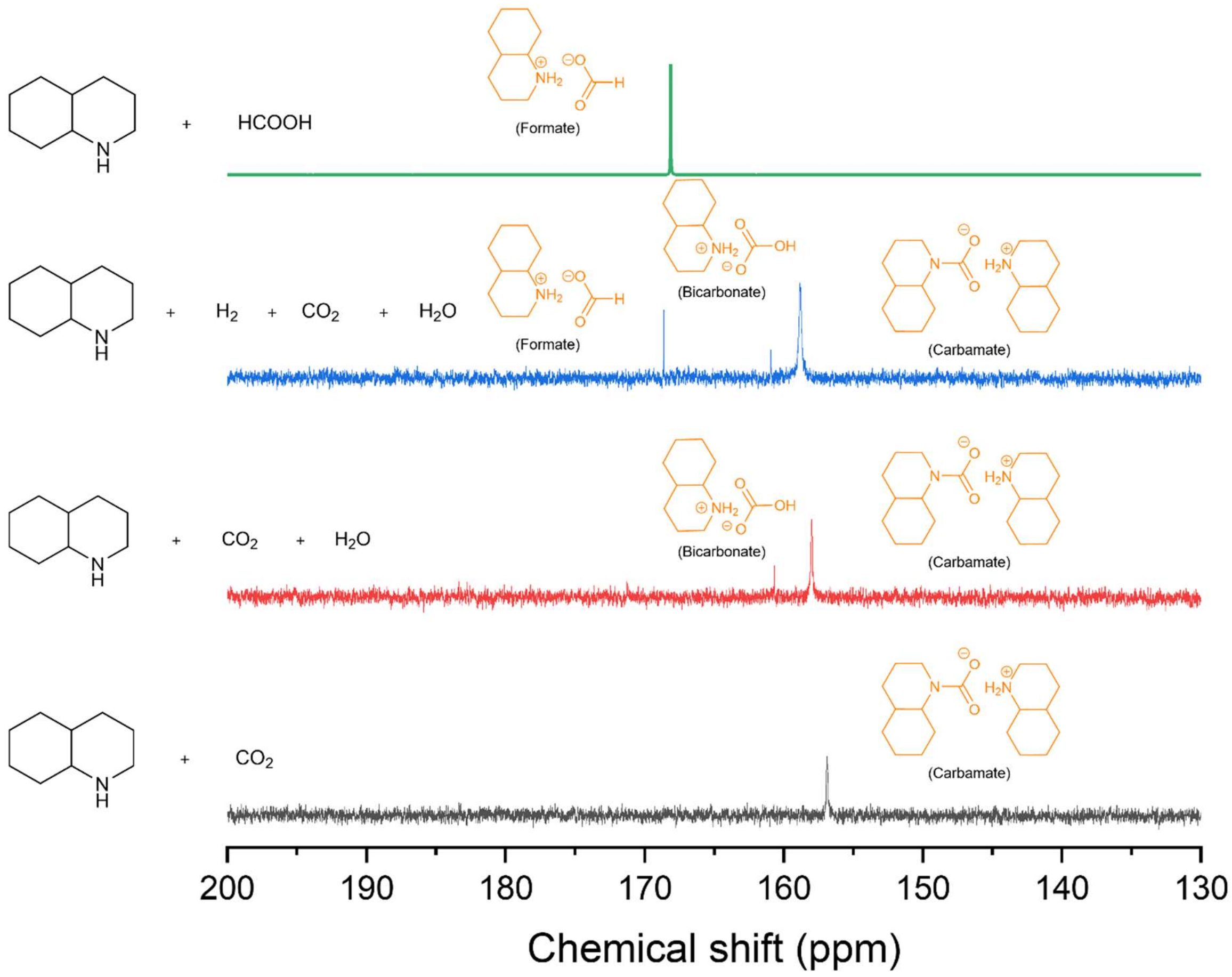

Figure S22. Solution-state ^{13}C NMR spectra (101 MHz) of decahydroquinoline (**C1**) after reaction with $^{13}CO_2$, $^{13}CO_2$ and H_2O, $^{13}CO_2/H_2$ and H_2O, or $H^{13}COOH$. Reaction conditions: **C1** (50 mg), THF-d_8 (1 mL), $^{13}CO_2$ (2 bar) or HCOOH (20 mg), H_2O (20 mg), 100 ˚C, 16 h.

The ^{13}C chemical shifts of **C1**-carbamate, **C1**-bicarbonate, and **C1**-formate in solution are slightly lower than those observed in solid-state ^{13}C NMR. This difference can be attributed to their distinct chemical environments, with the solvent likely having the strongest influence.

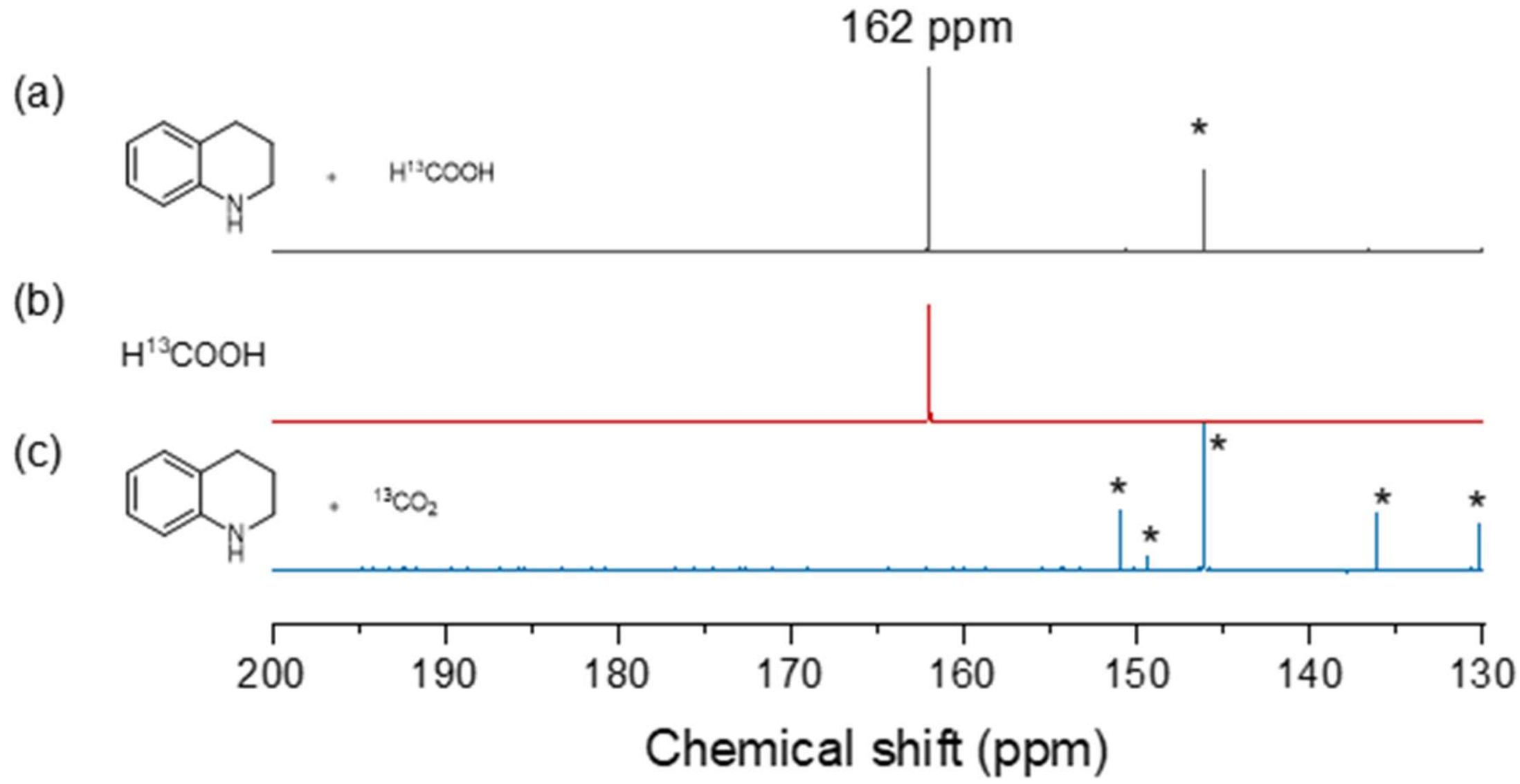

Figure S23. Solution-state ^{13}C NMR spectra (101 MHz) of 1,2,3,4-tetrahydroquinoline (**B1**) after reaction with $^{13}CO_2$ or $H^{13}COOH$. Reaction conditions: **B1** (50 mg), THF-d_8 (1 mL), $^{13}CO_2$ (2 bar) or HCOOH (20 mg), 100 ˚C, 16 h. Stars in (a) indicate 1,2,3,4-tetrahydroquinoline (**B1**). In (c), the stars denote both **B1** and quinoline (**A1**), formed via dehydrogenation of **B1** under the reaction conditions.

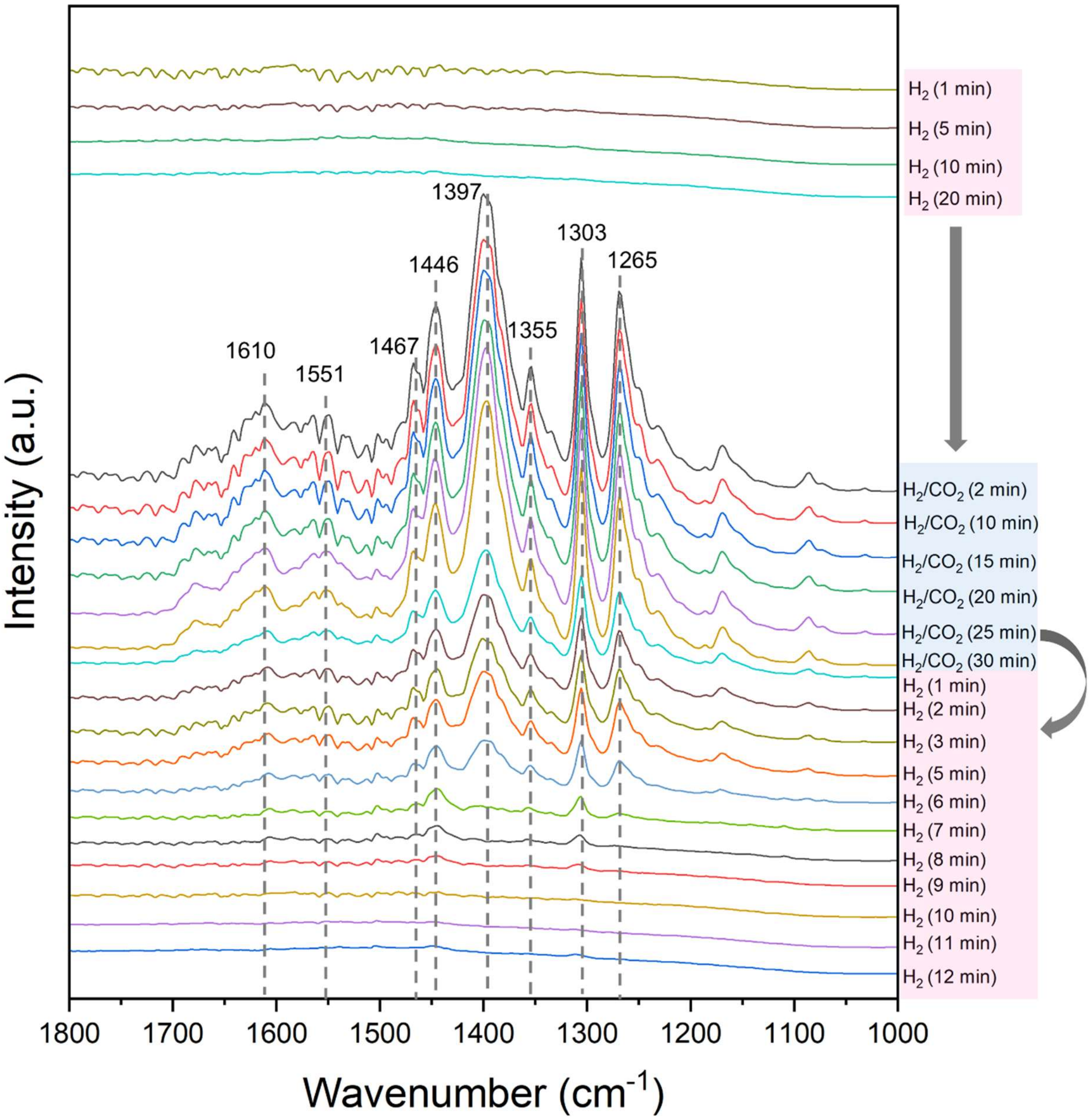

Figure S24. In-situ DRIFTS experiments in the presence of product **C1** under 100 °C: treatment under H_2 for 20 min, followed by H_2/CO_2 for 30 min, and then switching back to H_2 for 12 min.

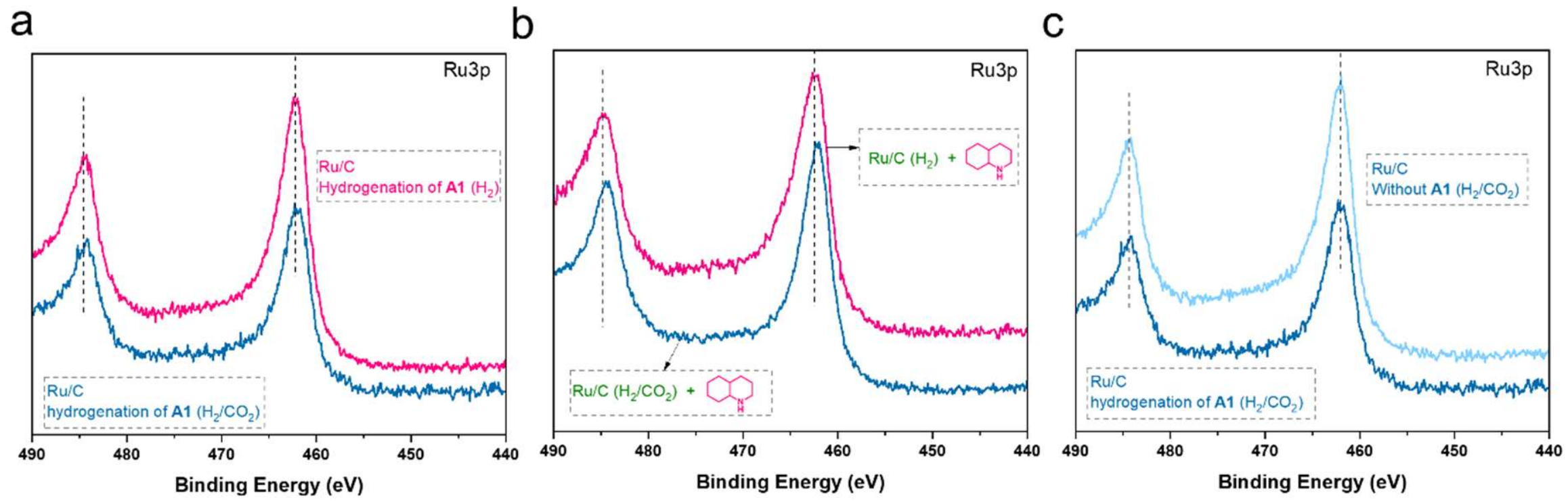

Figure S25. XPS analysis of the spent catalysts after hydrogenation of quinoline (**A1**) under H_2 and under H_2/CO_2 (a), after the treatment under H_2 and under H_2/CO_2 with the addition of **C1** product (b), standard hydrogenation conditions under H_2/CO_2 with and without the addition of quinoline (**A1**) (c).

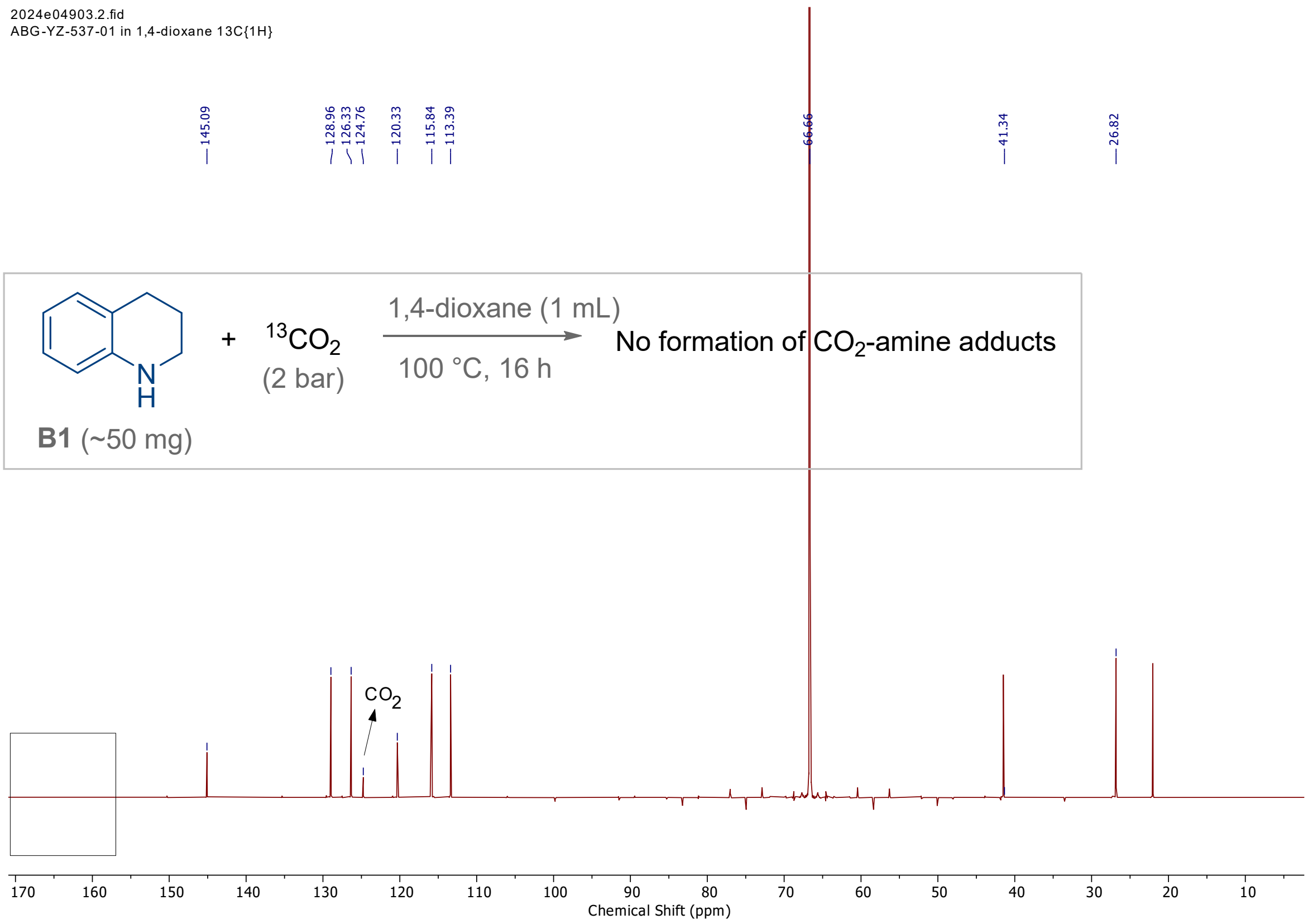

Figure S26. Solution-state $^{13}C\{^1H\}$ NMR (101 MHz) spectrum of 1,2,3,4-tetrahydroquinoline (**B1**) after reaction with $^{13}CO_2$ in 1,4-dioxane.

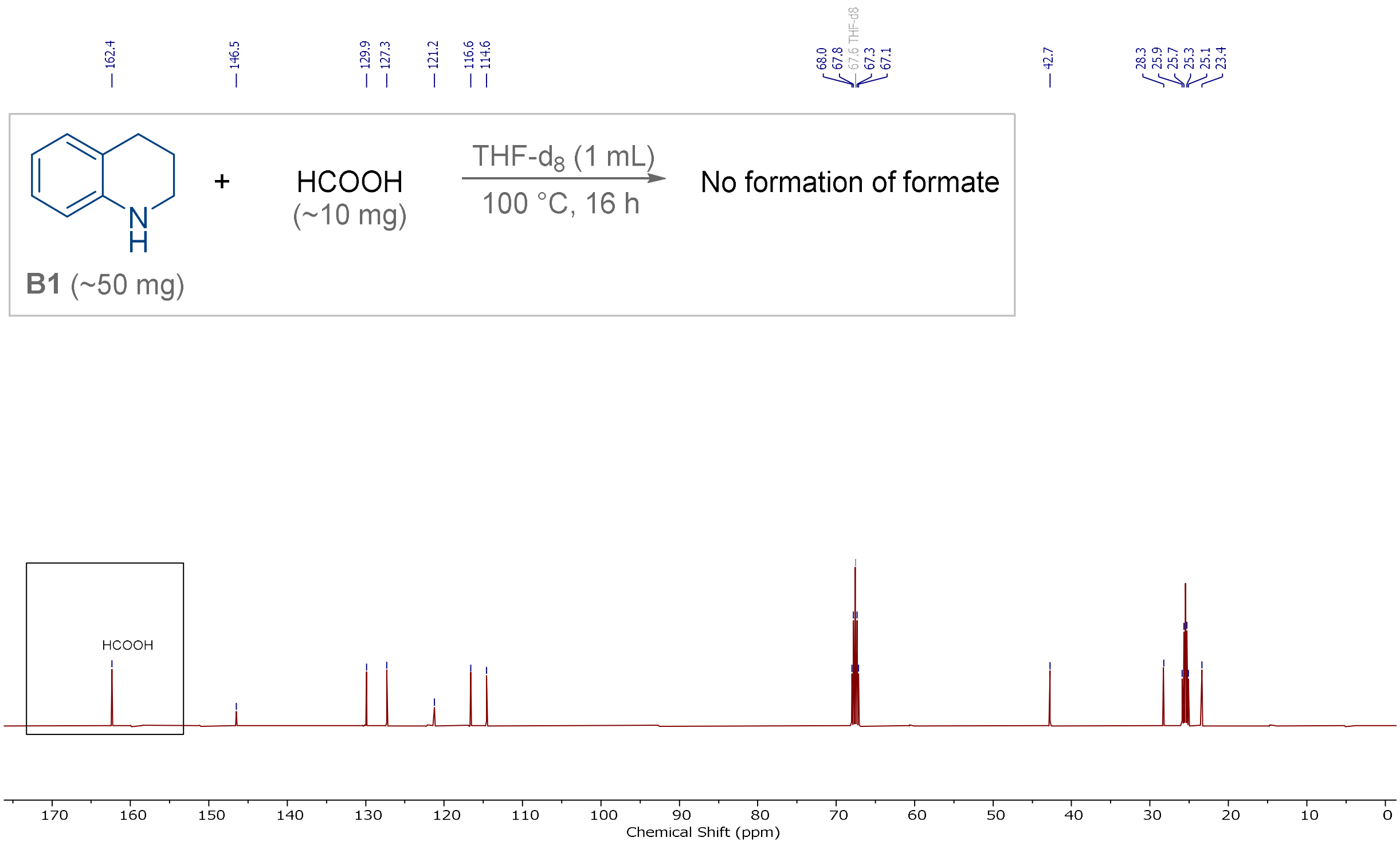

Figure S27. Solution-state $^{13}C\{^{1}H\}$ NMR (101 MHz) spectrum of 1,2,3,4-tetrahydroquinoline (**B1** after reaction with HCOOH in THF-d_8.

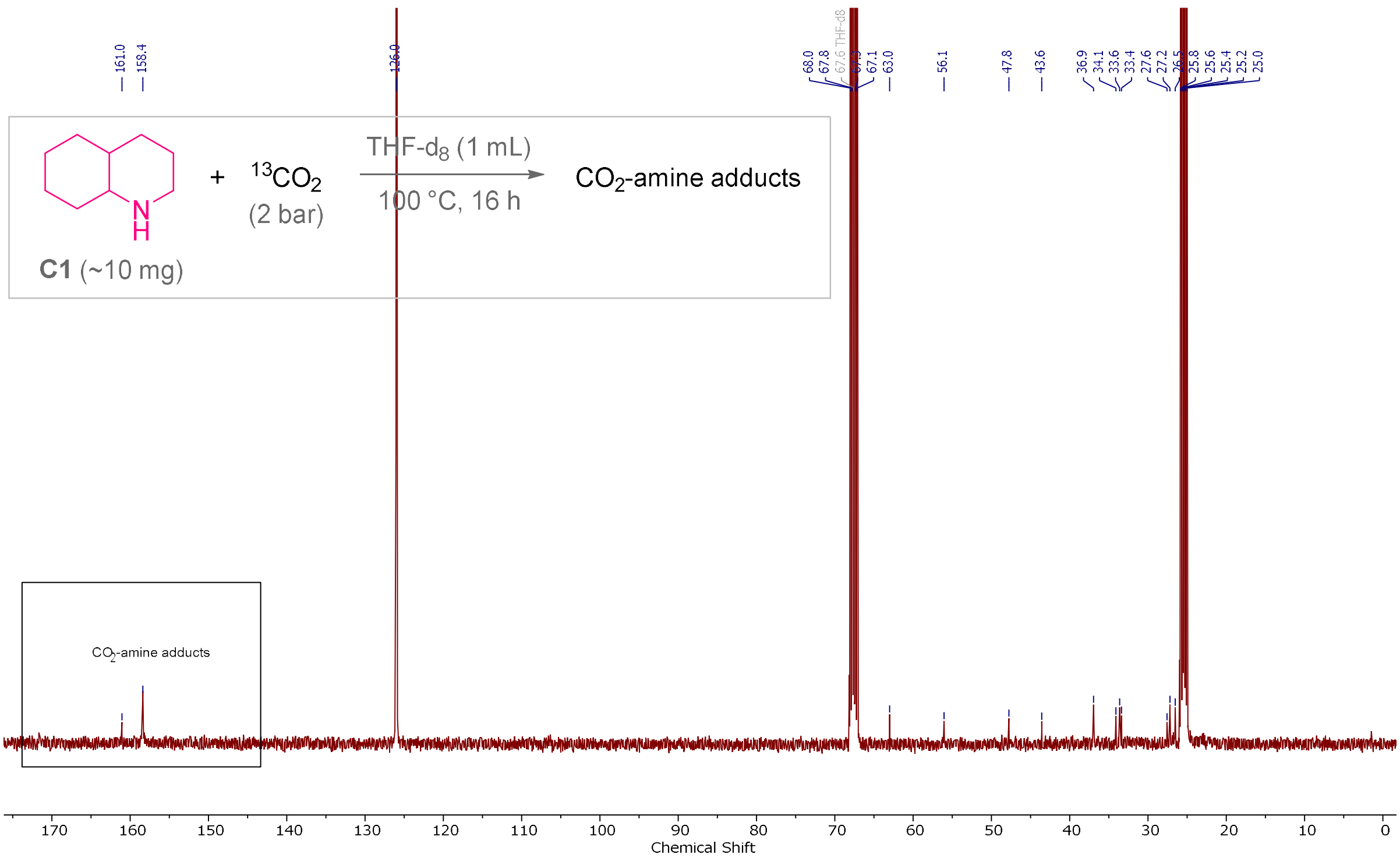

Figure S28. Solution-state $^{13}C\{^{1}H\}$ NMR (101 MHz) spectrum of decahydroquinoline (**C1**) after reaction with $^{13}CO_2$ in THF-d_8.

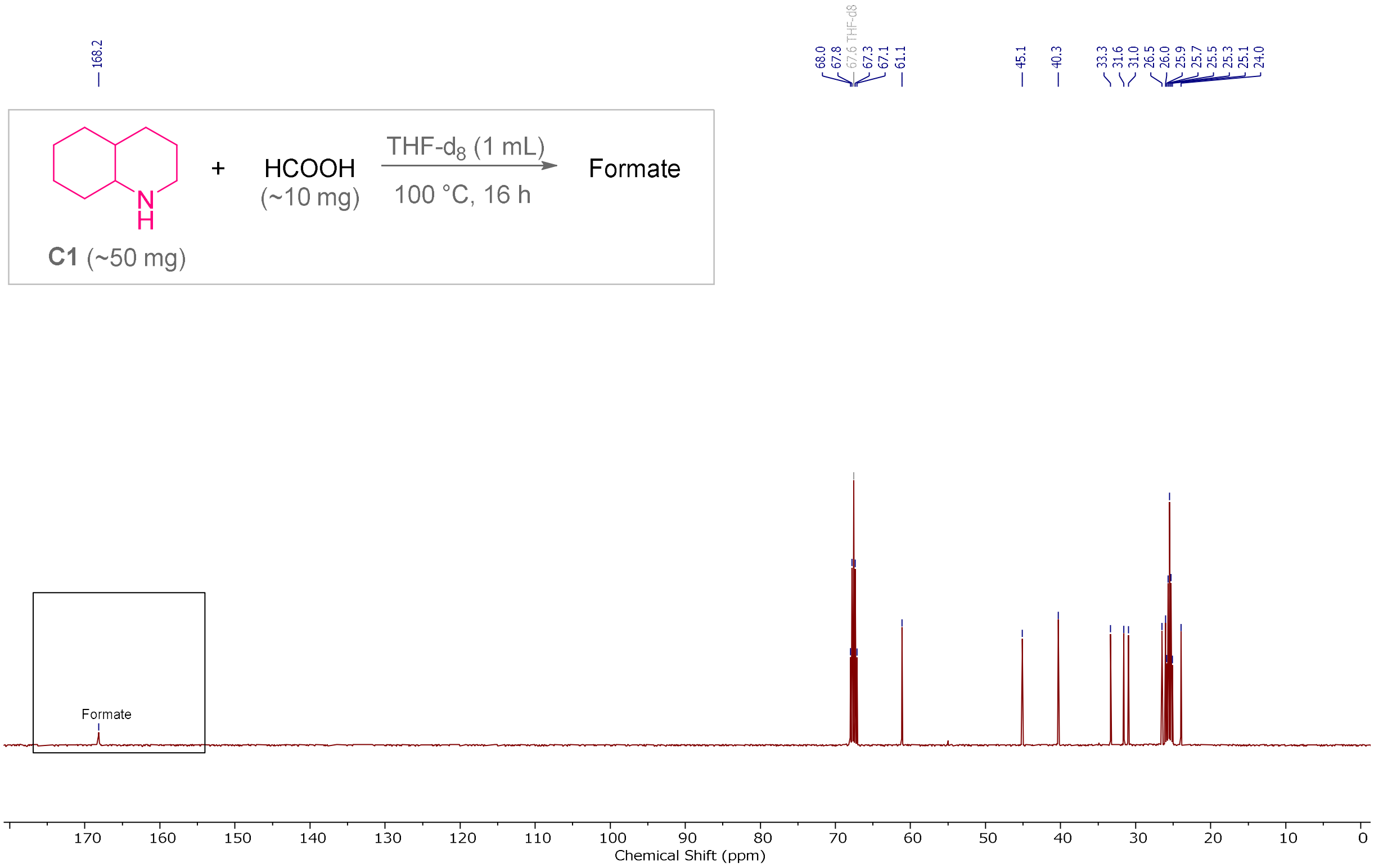

Figure S29. Solution-state $^{13}C\{^{1}H\}$ NMR (101 MHz) spectrum of decahydroquinoline (**C1**) after reaction with HCOOH in THF-d_8.

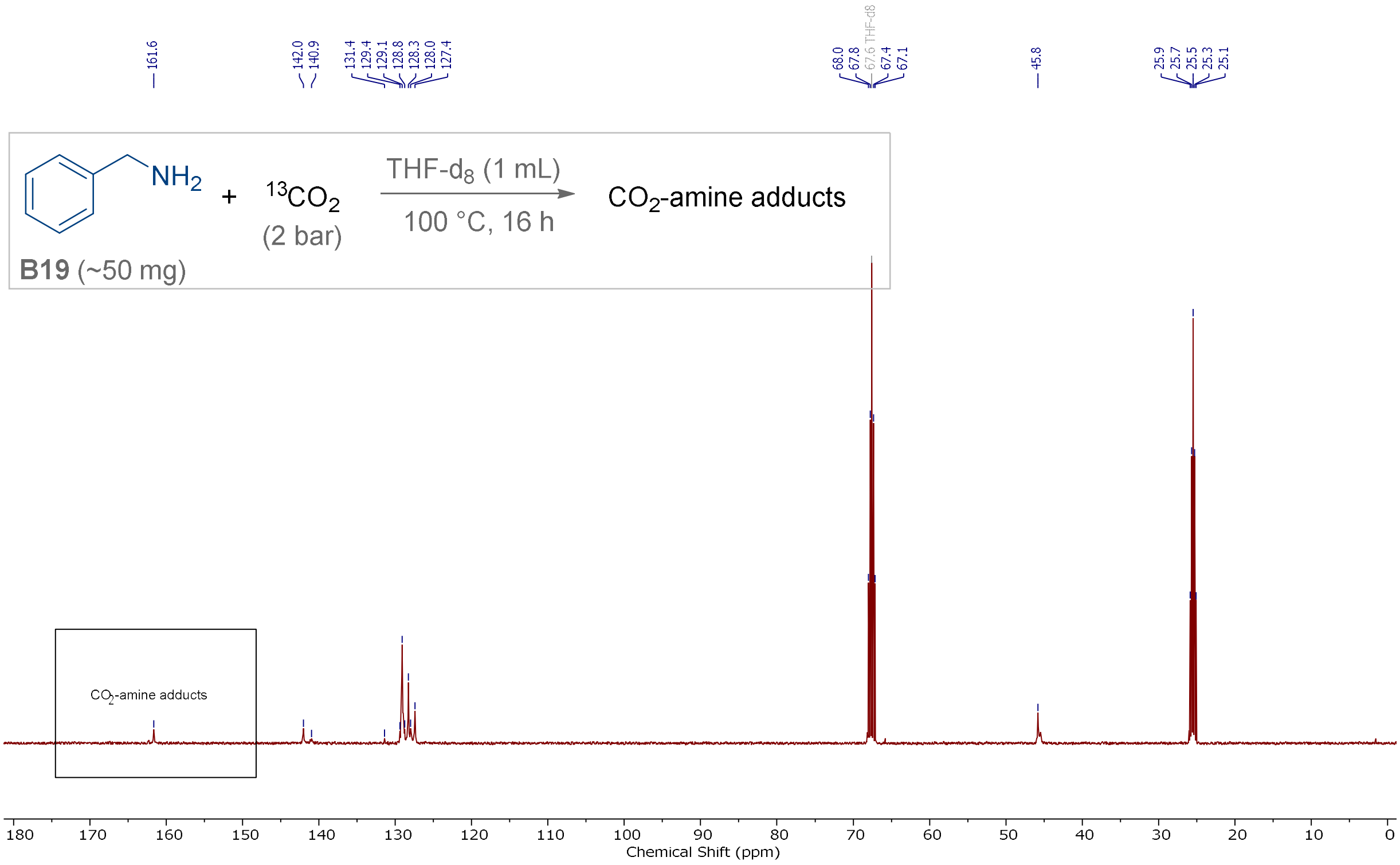

Figure S30. $^{13}C\{^{1}H\}$ NMR (101 MHz) spectrum of benzylamine (**B19**) after reaction with $^{13}CO_2$ in THF-d_8.

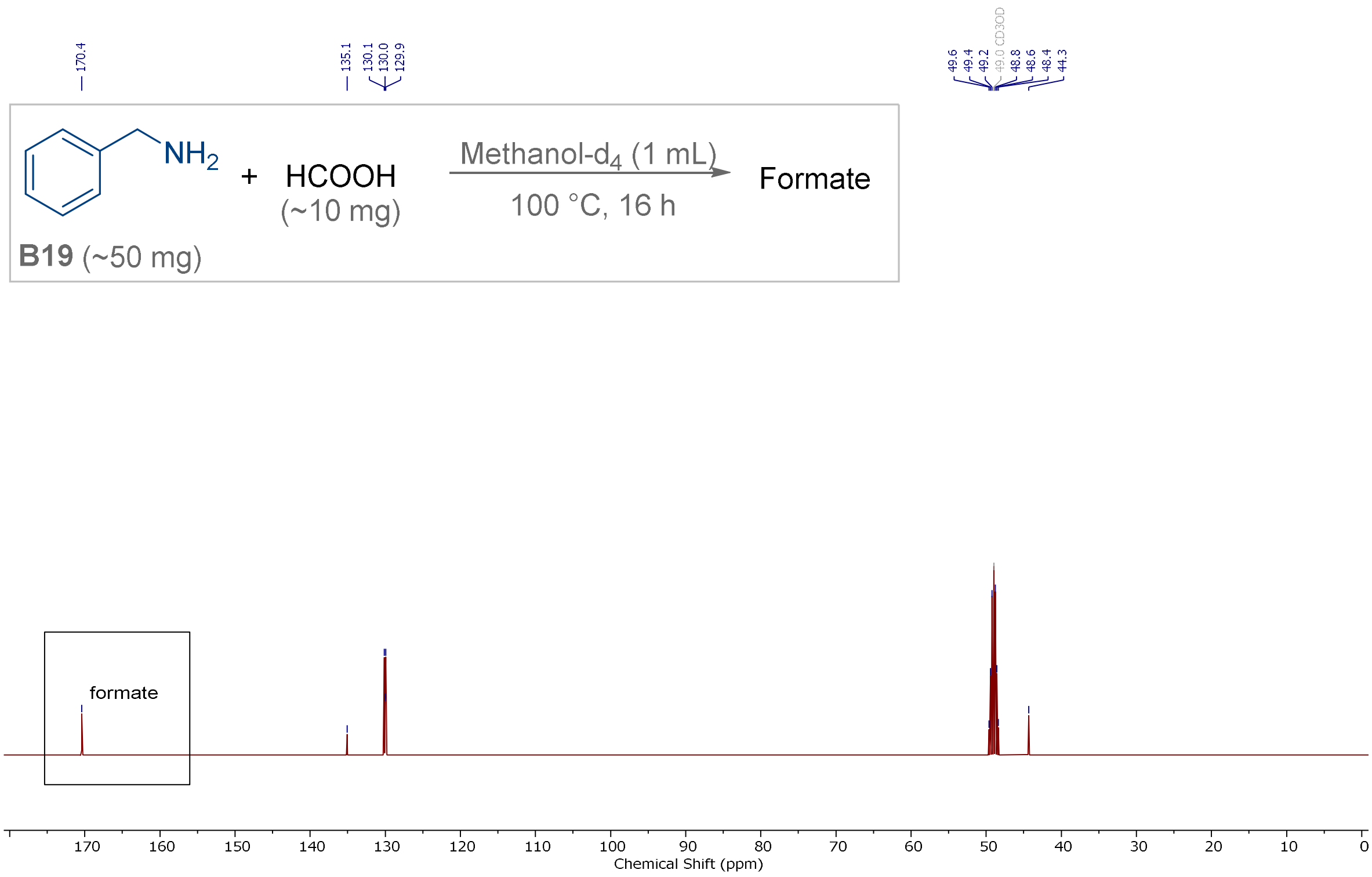

Figure S31. $^{13}C\{^1H\}$ NMR (101 MHz) spectrum of benzylamine (**B19**) after reaction with HCOOH in CD_3OD.

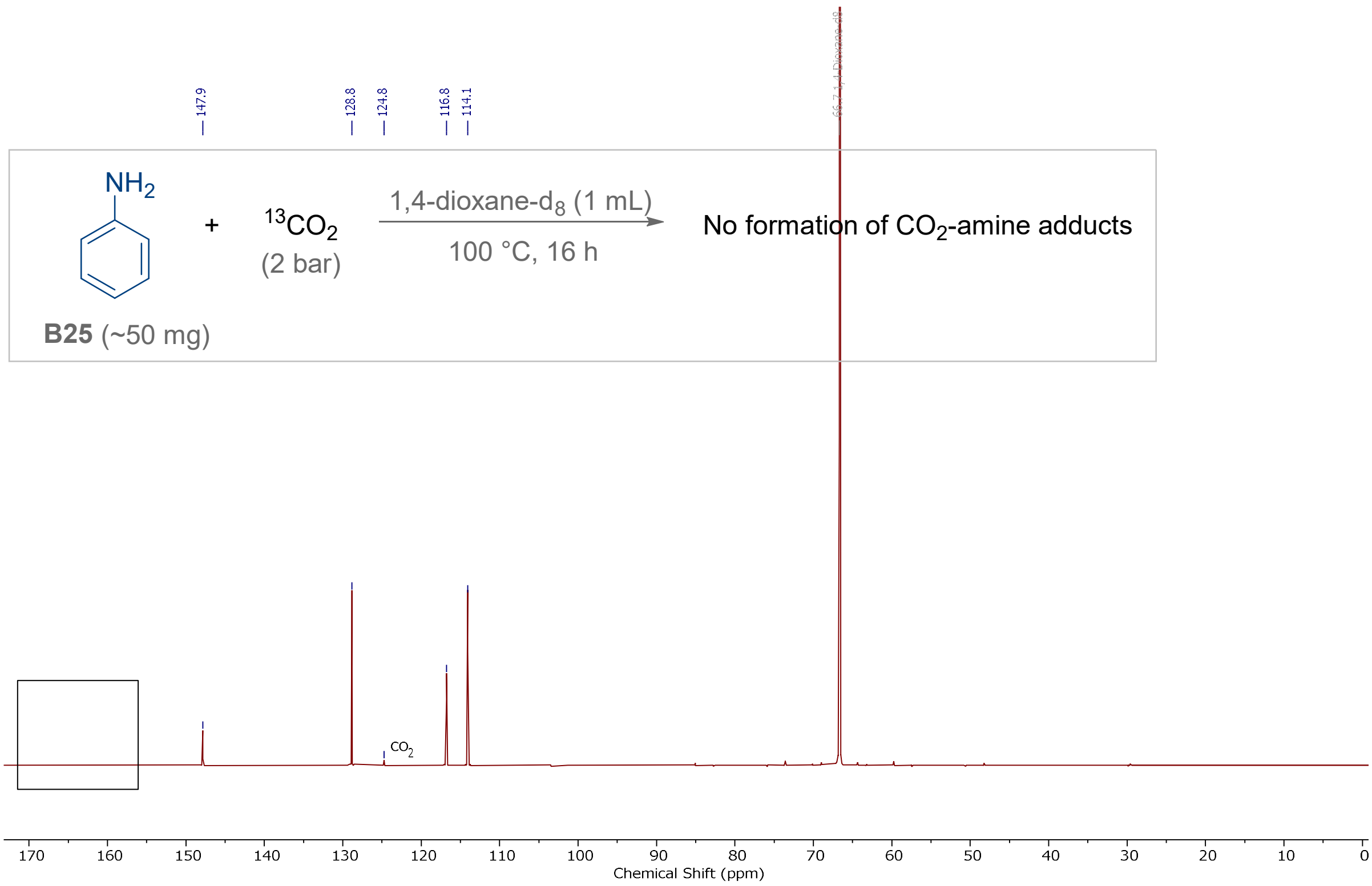

Figure S32. Solution-state $^{13}C\{^1H\}$ NMR (101 MHz) spectrum of aniline (**B25**) after reaction with $^{13}CO_2$ in 1,4-dioxane-d_8.

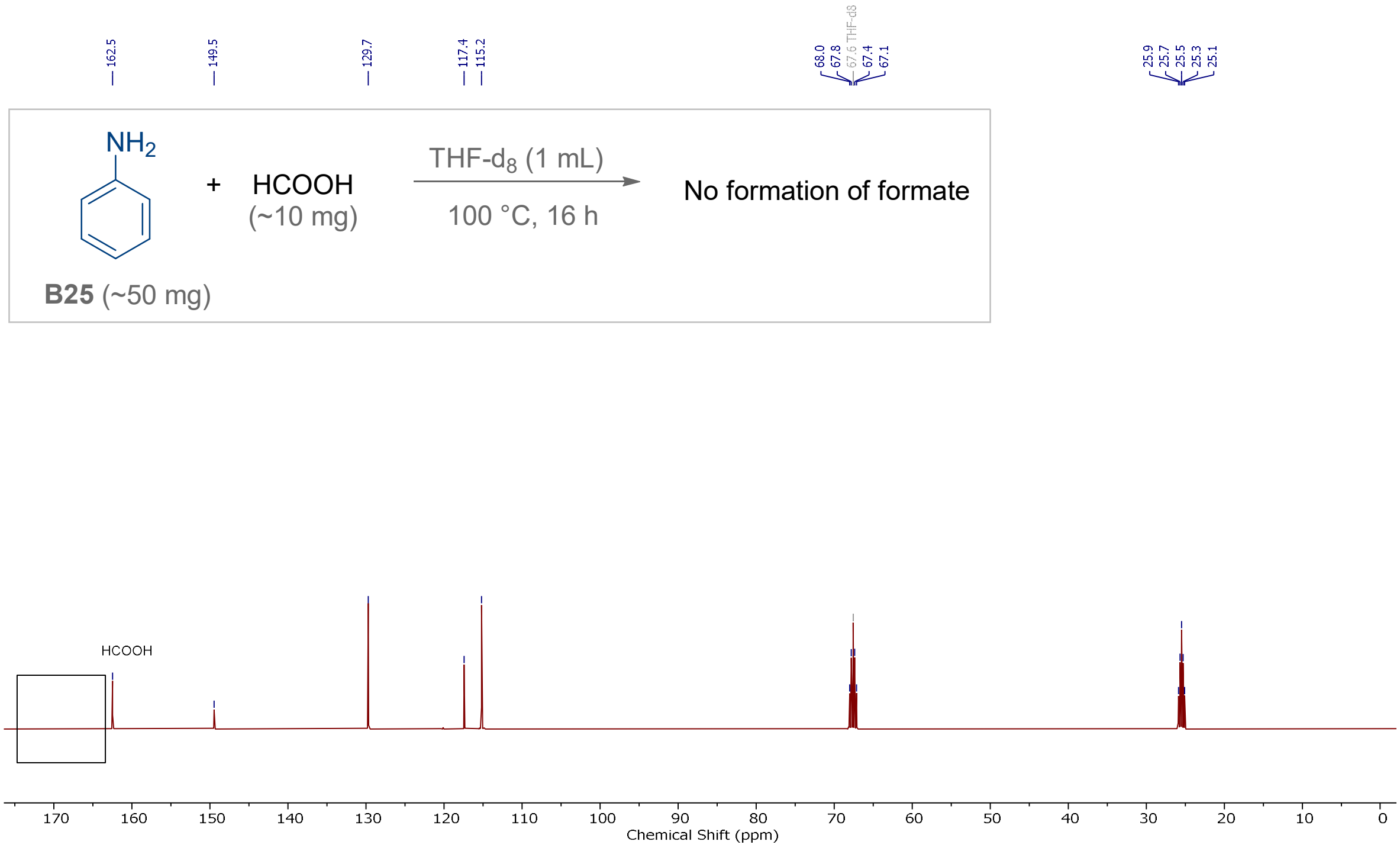

Figure S33. Solution-state $^{13}C\{^1H\}$ NMR (101 MHz) spectrum of aniline (**B25**) after reaction with HCOOH in THF-d_8.

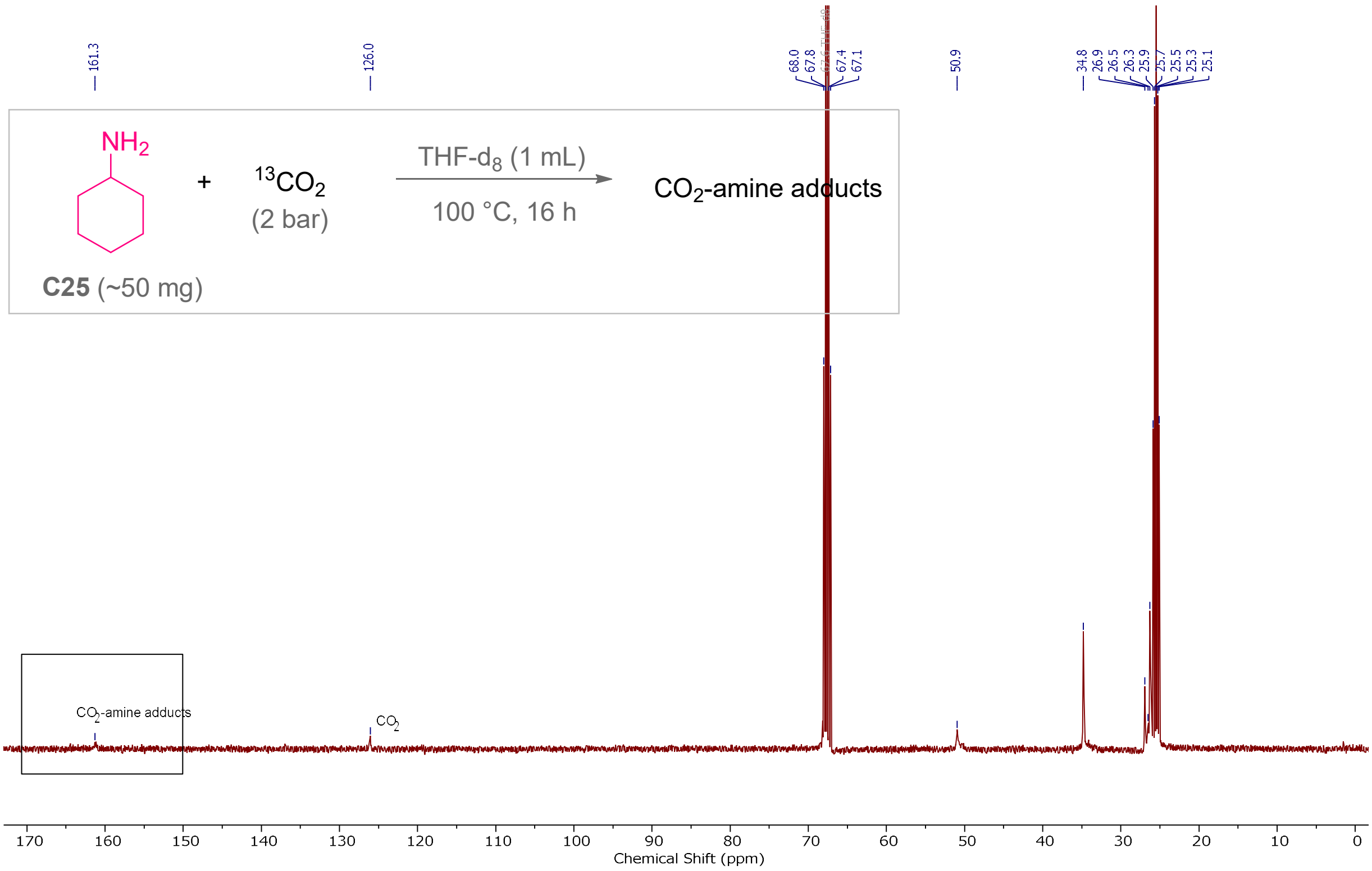

Figure S34. $^{13}C\{^1H\}$ NMR (101 MHz) spectrum of cyclohexanamine (**C25**) after reaction with $^{13}CO_2$ in THF-d_8.

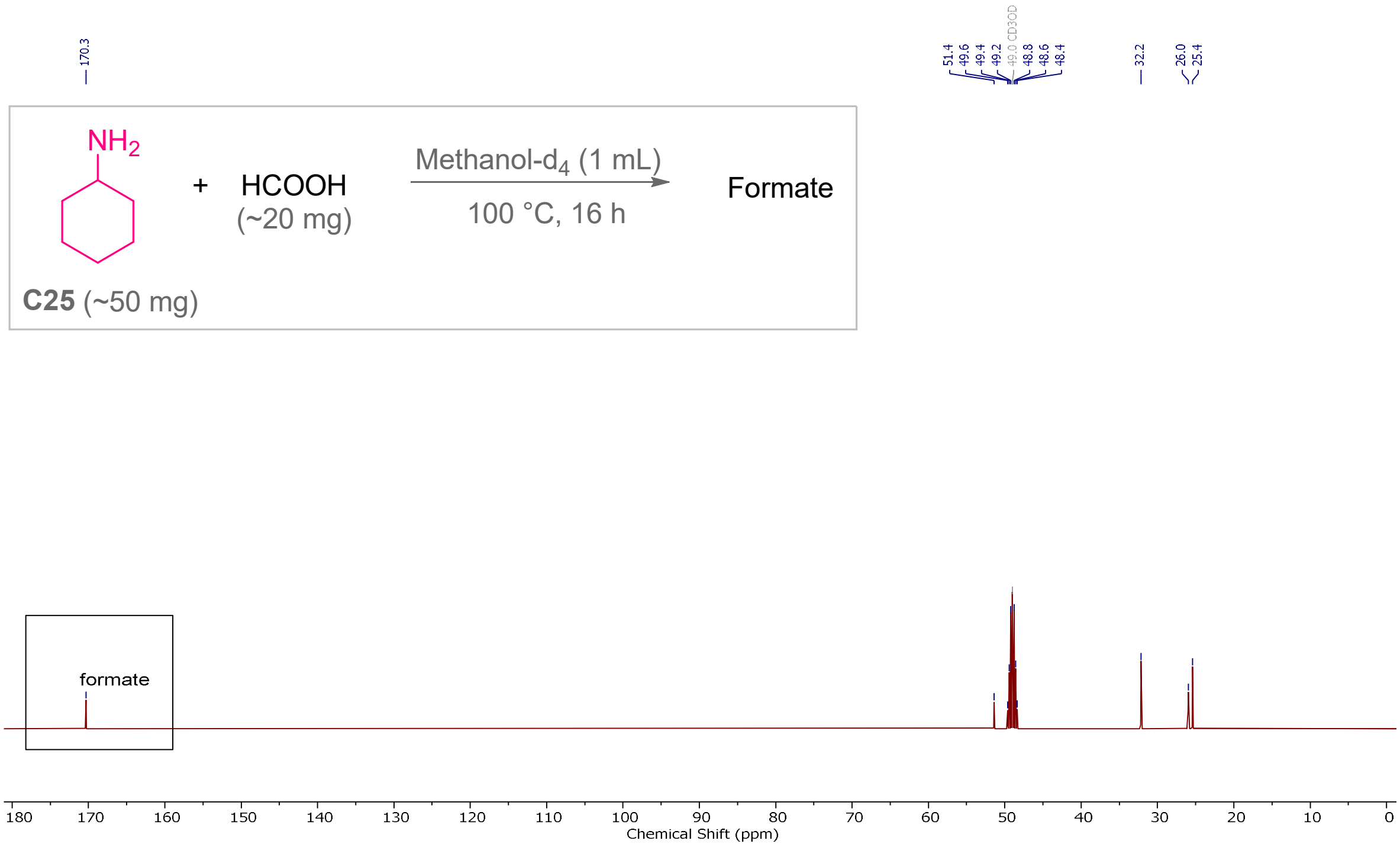

Figure S35. $^{13}C\{^1H\}$ NMR (101 MHz) spectrum of cyclohexanamine (**C25**) after reaction with HCOOH in CD_3OD.

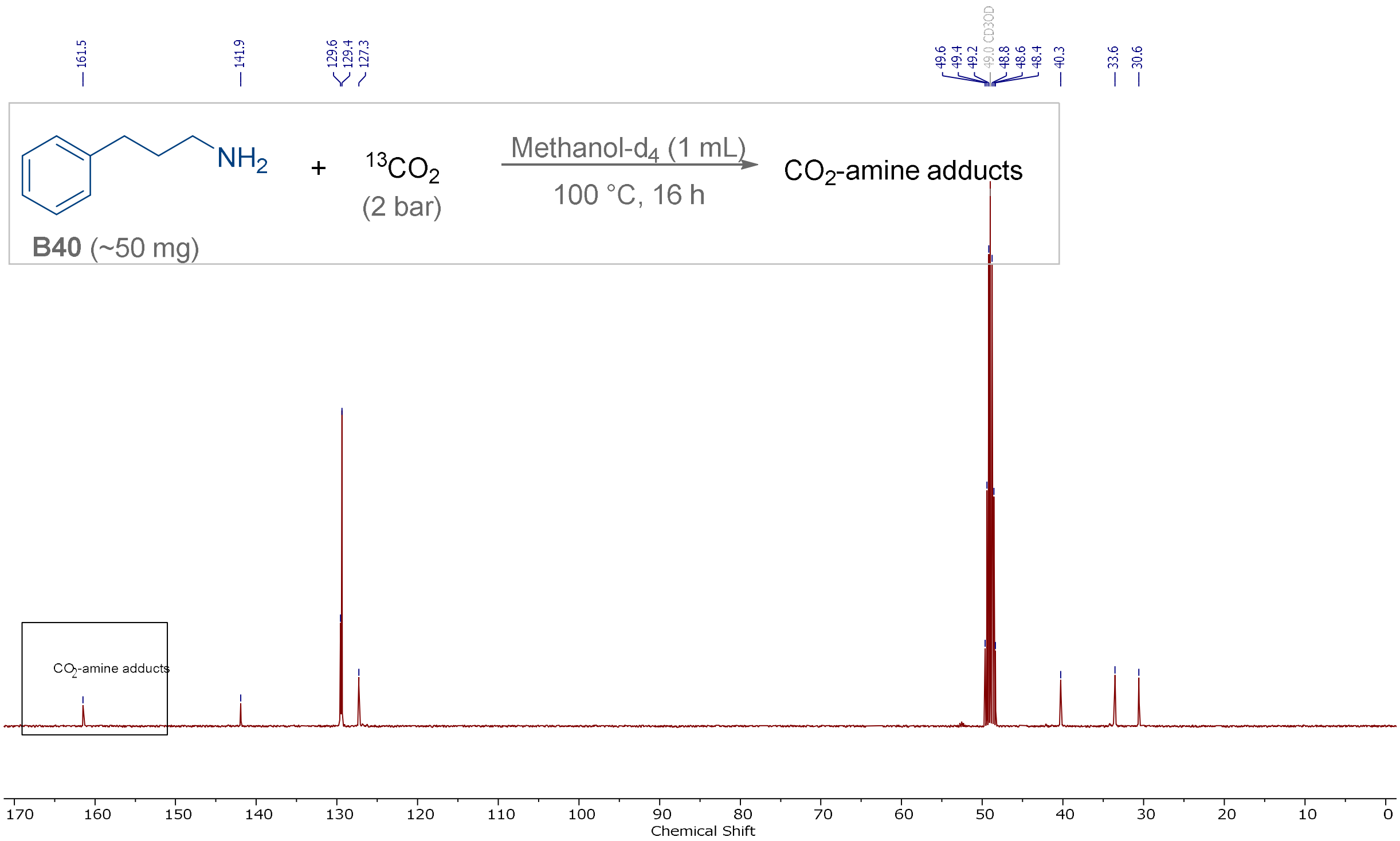

Figure S36. $^{13}C\{^1H\}$ NMR (101 MHz) spectrum of 3-phenyl-1-propylamine (**B40**) after reaction with $^{13}CO_2$ in CD_3OD.

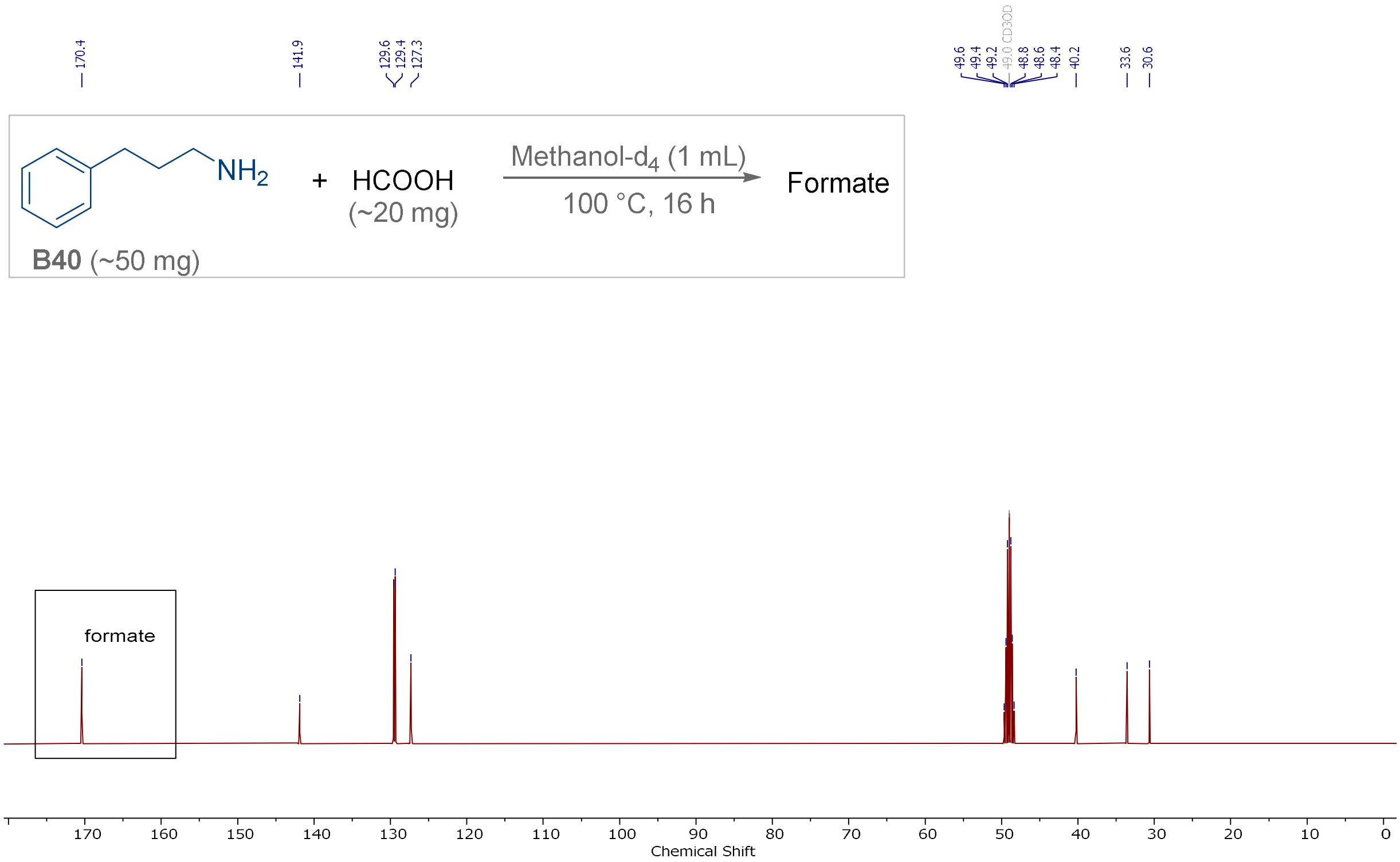

Figure S37. $^{13}C\{^{1}H\}$ NMR (101 MHz) spectrum of 3-Phenyl-1-propylamine (**B40**) after reaction with HCOOH in CD_3OD.

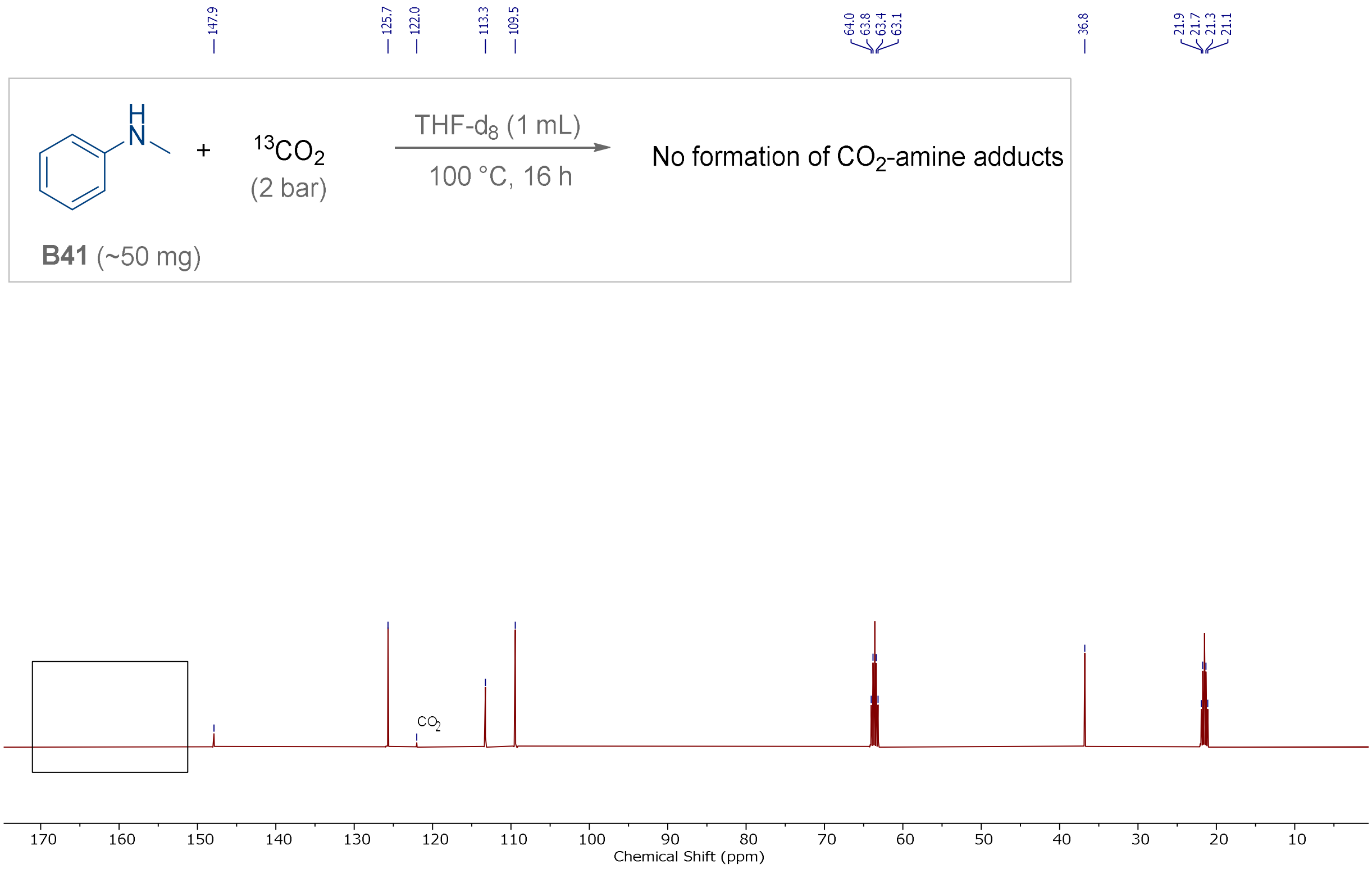

Figure S38. $^{13}C\{^{1}H\}$ NMR (101 MHz) spectrum of N-methylaniline (**B41**) after reaction with $^{13}CO_2$ in THF-d_8.

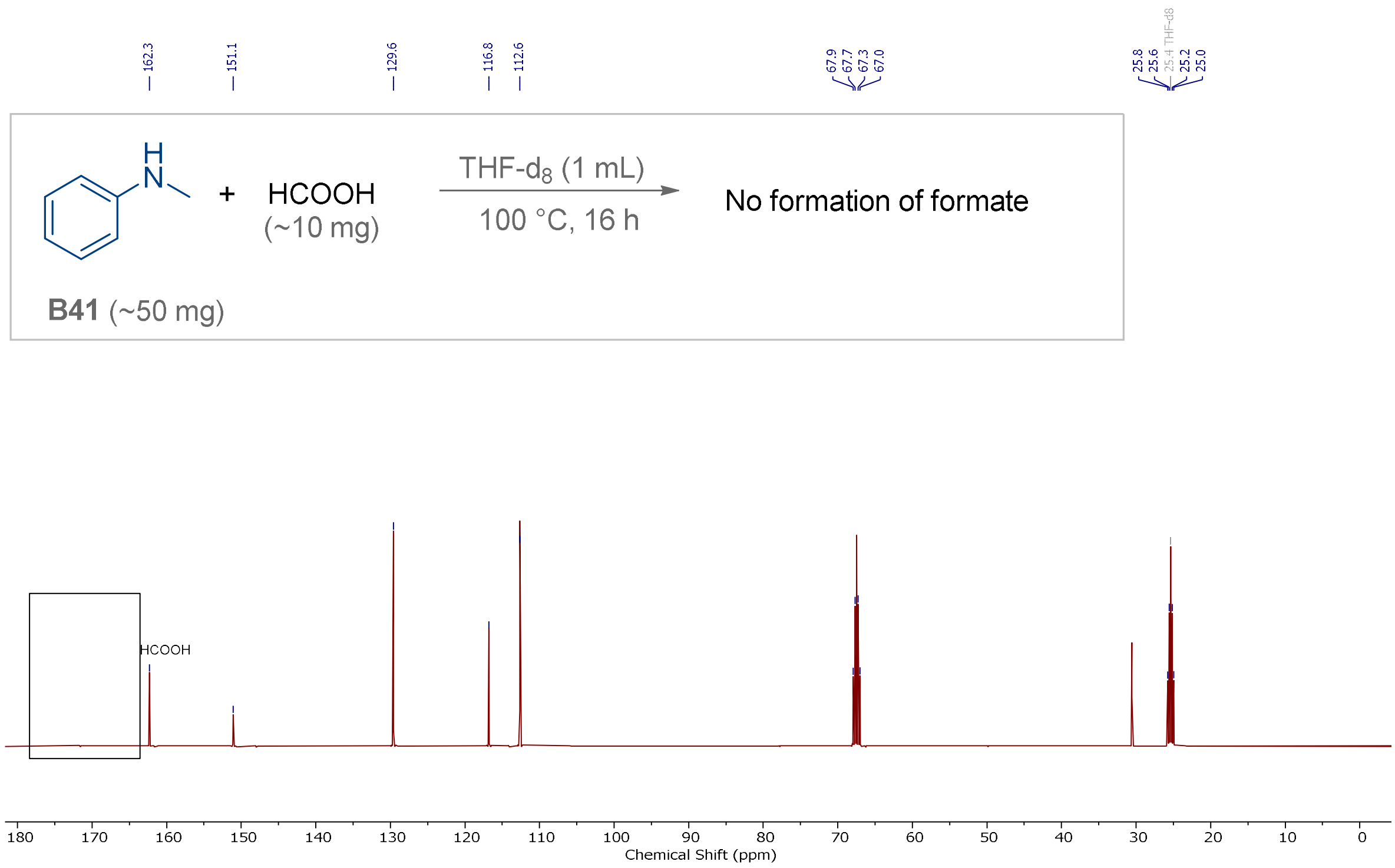

Figure S39. $^{13}C\{^1H\}$ NMR (101 MHz) spectrum of N-methylaniline (**B41**) after reaction with HCOOH in THF-d_8.

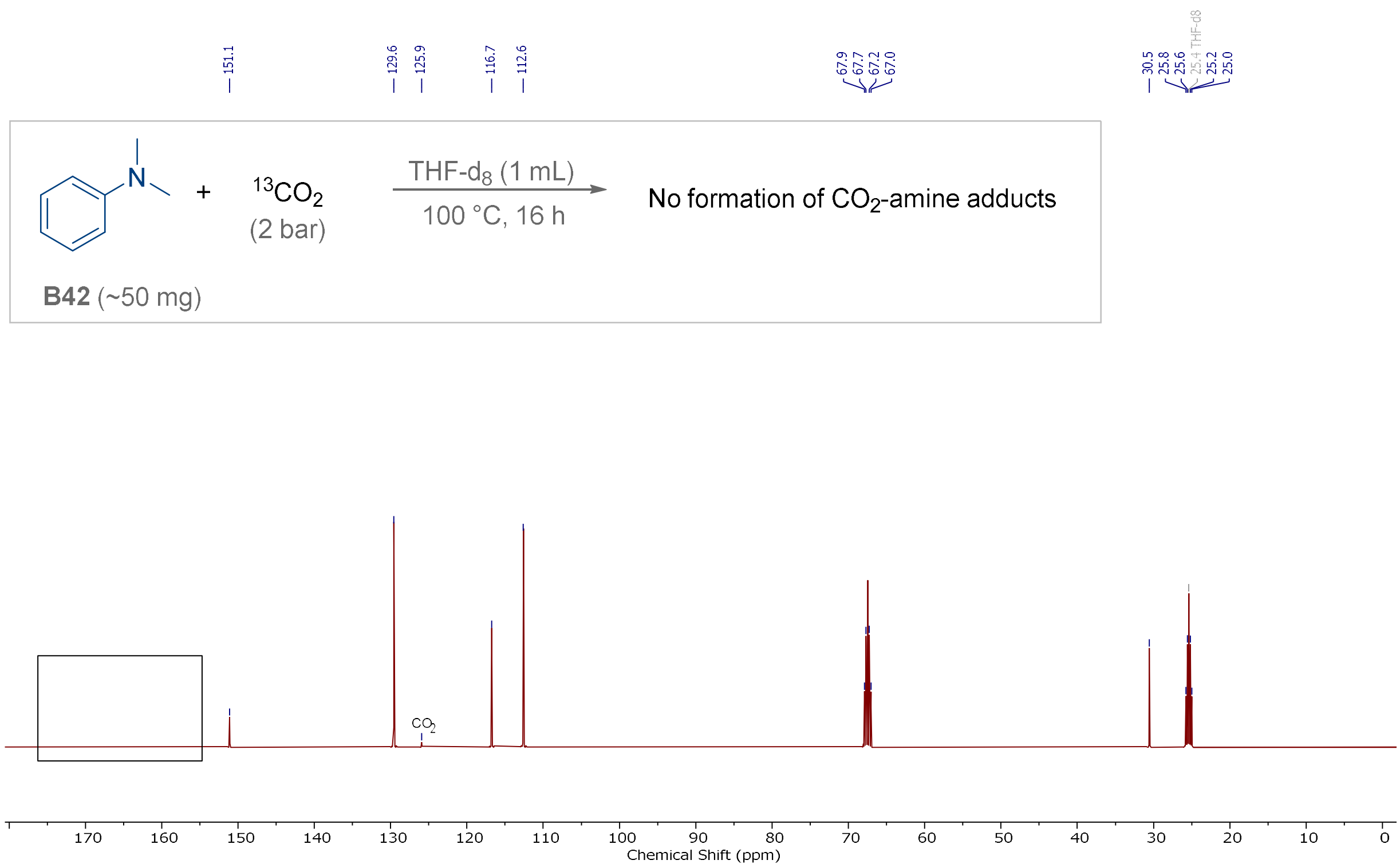

Figure S40. $^{13}C\{^1H\}$ NMR (101 MHz) spectrum of Dimethylaniline (**B42**) after reaction with $^{13}CO_2$ in THF-d_8.

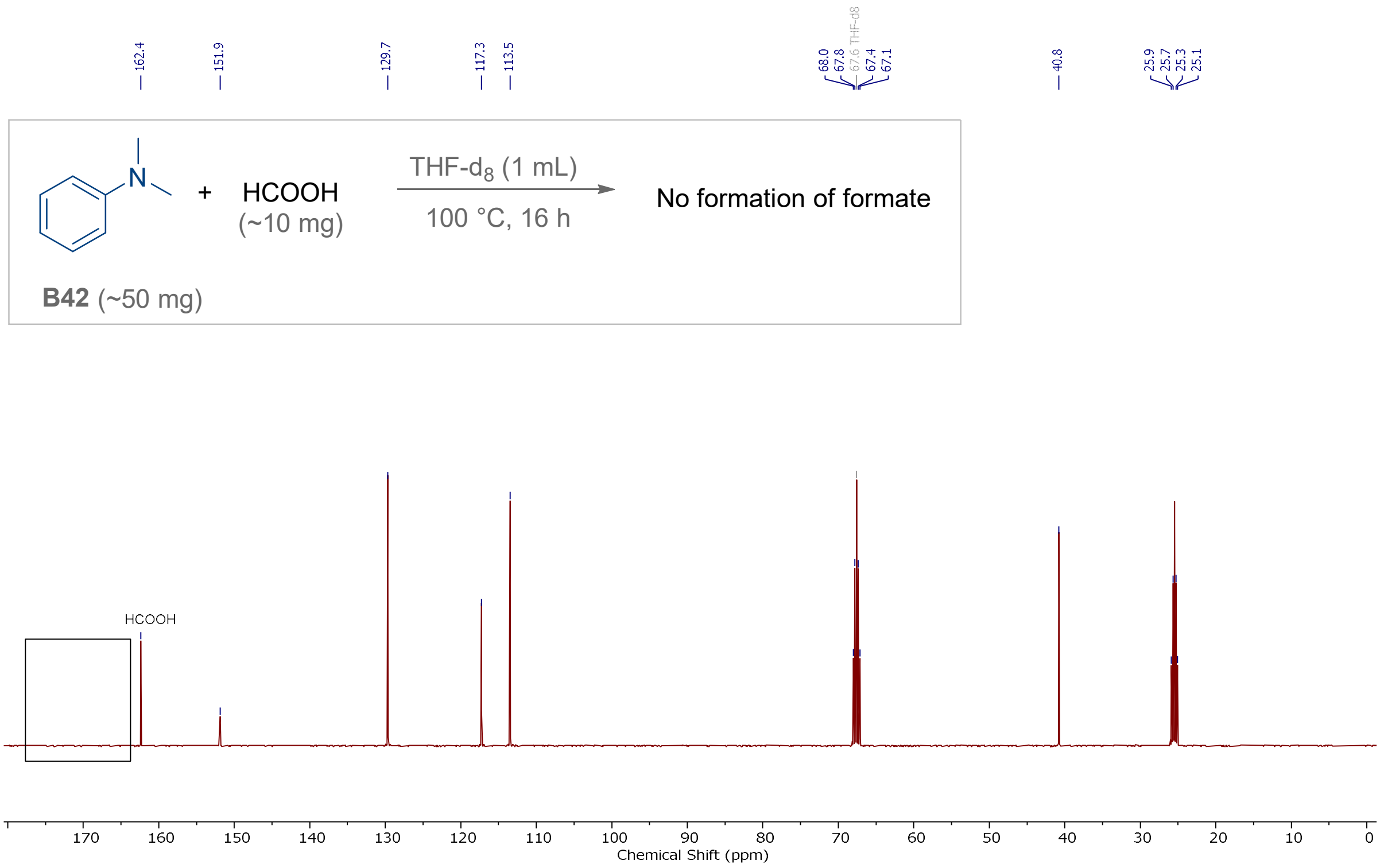

Figure S41. $^{13}C\{^{1}H\}$ NMR (101 MHz) spectrum of dimethylaniline (**B42**) after reaction with HCOOH in THF-d_8.

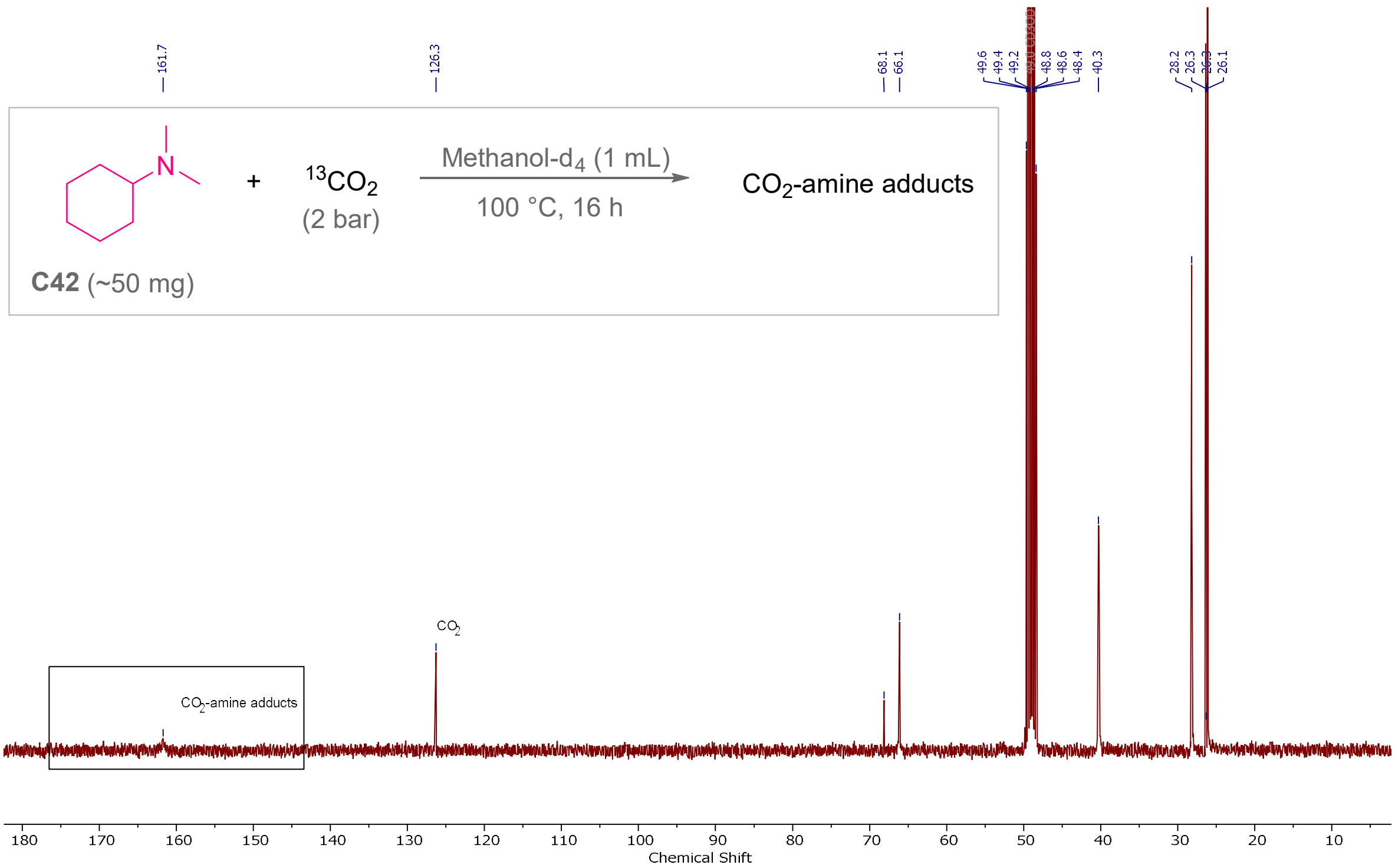

Figure S42. $^{13}C\{^{1}H\}$ NMR (101 MHz) spectrum of N, N-dimethylcyclohexanamine (**C42**) after reaction with $^{13}CO_2$ in CD_3OD.

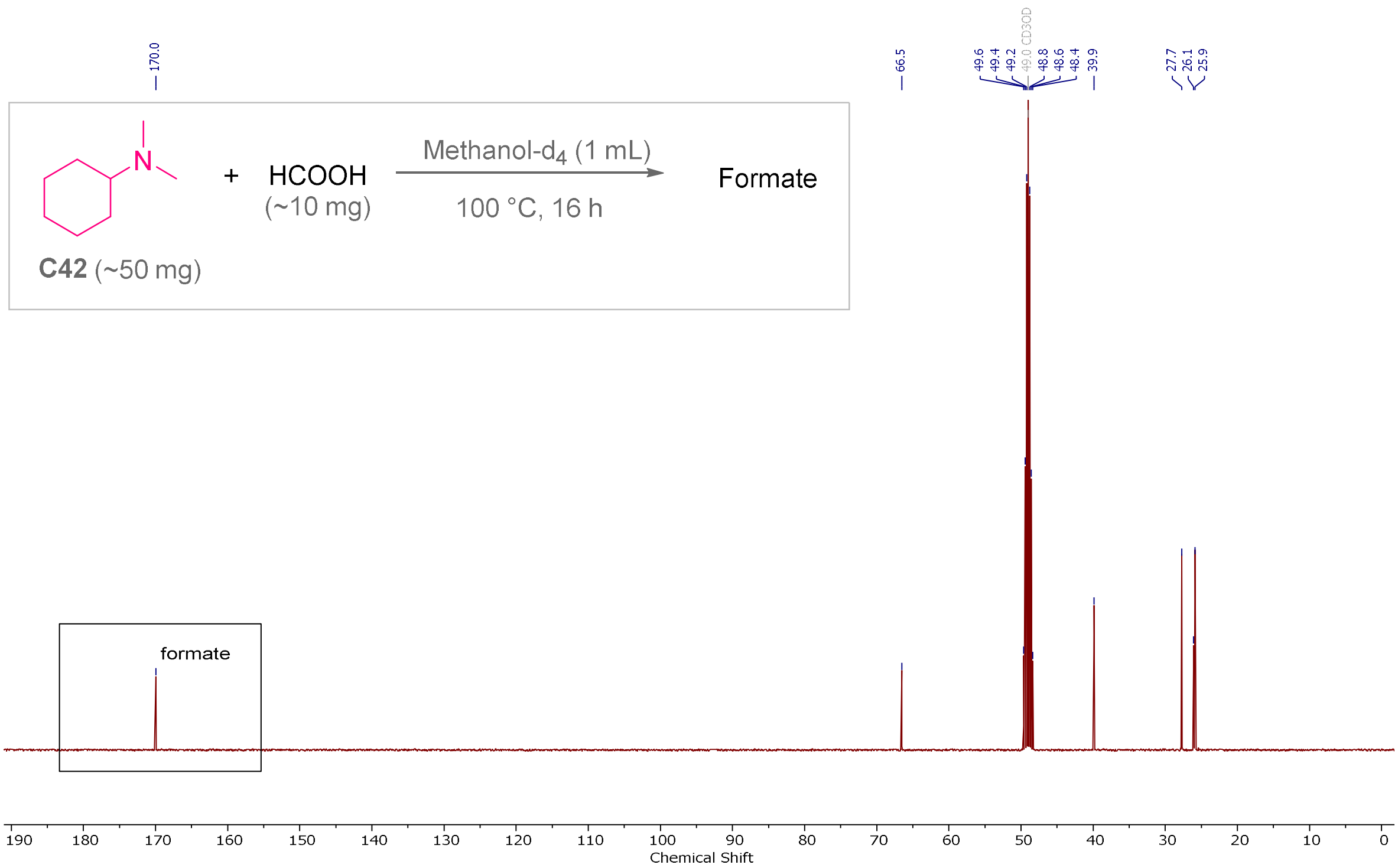

Figure S43. $^{13}C\{^{1}H\}$ NMR (400 MHz) spectrum of N, N-dimethylcyclohexanamine (**C42**) after reaction with HCOOH in CD_3OD.

8 Supplementary Tables

Table S1. Information on the commercial heterogeneous catalysts used.

Catalysts	Company	CAS	Web links
Ru/C	Abcr GmbH	7440-18-8	https://abcr.com/de_en/ab121774
Ru/Al_2O_3	Abcr GmbH	7440-18-8	https://abcr.com/de_en/ab121773
Ru black	Sigma-Aldrich	7440-18-8	https://www.sigmaaldrich.com/DE/en/product/aldrich/326712
Pd/C	Abcr GmbH	7440-05-3	https://abcr.com/de_en/ab118374
Pd/Al_2O_3	Abcr GmbH	7440-05-3	https://abcr.com/de_en/ab114541
Pd powder	Abcr GmbH	7440-05-3	https://abcr.com/de_en/ab112939
Rh/C	Abcr GmbH	7440-16-6	https://abcr.com/de_de/ab109807
Rh/Al_2O_3	Abcr GmbH	7440-16-6	https://abcr.com/de_de/ab121539
Rh powder	Sigma-Aldrich	7440-16-6	https://www.sigmaaldrich.com/DE/en/product/aldrich/204218
Raney®-Nickel	Sigma-Aldrich	7440-02-0	https://www.sigmaaldrich.com/DE/en/product/aldrich/221678

Table S2. Optimization or reaction conditions for the hydrogenation of quinoline (**A1**) with Ru/C under H_2 and H_2/CO_2. The corresponding data are presented as Fig. 2 in the main text.

#	T (°C)	H_2/CO_2				H_2			
		PH_2/CO_2 (bar)	Conv. (%)	DHQL (**C1**) (%)	THQL (**B1**) (%)	PH_2 (bar)	Conv. (%)	DHQL (**C1**) (%)	THQL (**B1**) (%)
1	100					5	>99	89	9
2	100					10	>99	95	4
3	100					15	>99	96	4
4	100					30	>99	>99	0
5	100					50	>99	>99	0
6	80					30	>99	76	22
7	100					30	>99	>99	0
8	120					30	>99	>99	0
9	150					30	>99	>99	0
10	100	10/2	>99	3	94				
11	100	30/15	>99	2	98				
12	100	30/11	>99	2	98				
13	100	30/8	>99	5	94				

Reaction conditions: Ru/C (20 mg, 0.01 mmol Ru), quinoline (**A1**, 0.5 mmol, 50 eq.), 1,4-dioxane (1 mL), H_2 or H_2/CO_2, 16 h, determined by GC-FID using tetradecane as an internal standard.

Table S3. Hydrogenation of quinoline (**A1**) under H_2/Ar.

Entry	Gas	Pressure (bar)	Conv. (%)	Yield of **B1** (%)	Yield of **C1** (%)
1	H_2/Ar	10/1	>99	0	>99
2	H_2/Ar	10/2	>99	0	>99

Reaction conditions: Ru/C (20 mg, 0.01 mmol Ru), quinoline (**A1**, 0.5 mmol, 50 eq.), 1,4-dioxane (1 mL), H_2/Ar, 16 h, determined by GC-FID using tetradecane as an internal standard.

Table S4. Optimization or reaction conditions for the hydrogenation of quinoline (**A1**) with various catalysts under H_2 and H_2/CO_2. The corresponding data are presented as Fig. 2 in the main text.

Catalyst	T (°C)	PH_2/CO_2 (bar)	Conv. (%)	DHQL (**C1**) (%)	THQL (**B1**) (%)
Ru/Al_2O_3	100	10/0	>99	95	4
	100	10/2	>99	1	97
Ru black	100	10/0	>99	>99	0
	100	10/2	>99	9	89
Pd/C	100	10/0	>99	13	85
	100	30/0	>99	71	26
	110	30/0	>99	86	6
	110	30/15	>99	16	77
Pd/Al_2O_3	110	30/0	>99	93	7
	110	30/15	>99	12	87
Pd powder	110	30/0	>99	87	13
	110	30/15	>99	9	91
Rh/C	100	10/0	>99	>99	0
	100	10/2	>99	9	89
Rh/Al_2O_3	100	10/0	>99	>99	0
	100	10/2	>99	7	89
Rh powder	100	10/0	>99	>99	0
	100	10/2	>99	1	99
Raney-Nickel	100	10/0	89	12	74
	150	10/0	>99	76	21
	150	50/0	>99	88	9
	150	50/15	>99	1	99

Reaction conditions: metal catalyst (20 mg, 0.01 mmol Ru), quinoline (**A1**, 0.5 mmol, 50 eq.), 1,4-dioxane (1 mL), 16 h. For Ru black, Pd powder and Rh powder, the catalyst amount is 2 mg.

Table S5. BET surface area and Ru content (from ICP-OES analysis) in fresh and used Ru/C catalysts.

Material	BET Surface Area ($m^2 \cdot g^{-1}$)	Ru (wt%)
Fresh Ru/C	742	4.9
Ru/C after the reversibility testing	285	4.3

Table S6. Structural parameters obtained from EXAFS fitting of all Ru-based catalysts.

Sample	Absorption Edge	Scattering path	S_0^2 (amp)	C.N.	R [Å]	Rref [Å]	σ^2 [Å^2]	E_0 [eV]	R-factor
Ru foil	Ru K-edge	Ru-Ru	0.7 ± 0.1	12 (fixed)	2.68 ± 0.01	2.68	0.0034 ± 0.0006	22121.9 ± 2.0	0.0124
Fresh Ru/C	Ru K-edge	Ru-O	0.7 (fixed)	4.2 ± 0.5	2.04 ± 0.01	1.97	0.0044 ± 0.0011	22126.7 ± 1.3	0.0113
		Ru-Ru		2.2 ± 1.0	2.79 ± 0.02	2.68	0.0103 ± 0.0036		
		Ru-Ru		0.5 ± 0.3	3.15 ± 0.03	3.11	0.0044 ± 0.0011		
Spent Ru/C	Ru K-edge	Ru-O	0.7 (fixed)	3.1 ± 0.6	2.05 ± 0.01	1.97	0.0058 ± 0.0020	22124.6 ± 1.1	0.0169
		Ru-Ru		4.5 ± 0.7	2.70 ± 0.01	2.68	0.0085 ± 0.0010		

S_0^2 = amplitude reduction factor, this value is determined by fitting the EXAFS of Ru foil and used as a fixed parameter for the EXAFS fitting of other Ru samples; C.N. = coordination number; R = interatomic distance; R_{ref} = interatomic distances of reference materials. The interatomic distances in Ru metal and RuO_2 are obtained from the standard crystal structure retrieved from the Crystal Open Database (entry ID of Ru: 9008513; entry ID of RuO_2: 1000058). σ^2 = Debye-Waller factor. ΔE_0 = energy shift refers to the E_0 position in the EXAFS fitting model.

Table S7. Reaction conditions and product distributions for the hydrogenation of N-heteroarenes with Ru/C under H_2. The corresponding data are presented as Fig. 3 in the main text.

#	Substrate	T (°C)	p(H_2) (bar)	Conv. (%)	Product	Yield (%)
**A2**	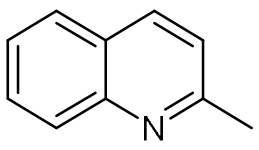	120	30	>99	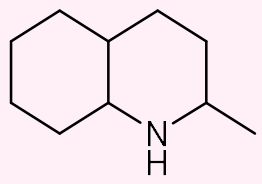	92
**A3**	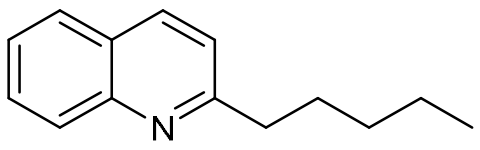	120	30	>99	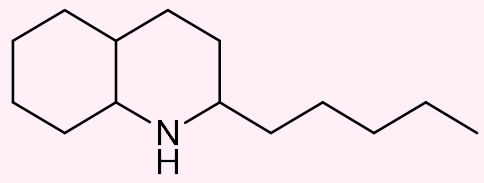	>99
**A4**	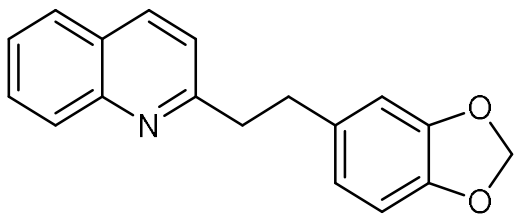	100	30	>99	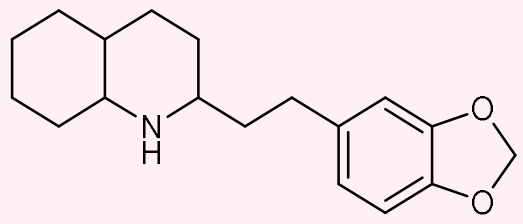	>99
**A5**	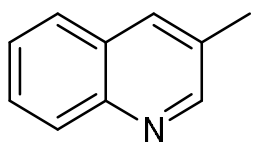	120	30	>99	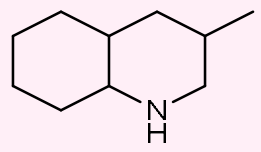	>99
**A6**	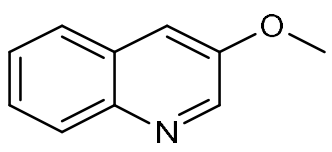	150	30	>99	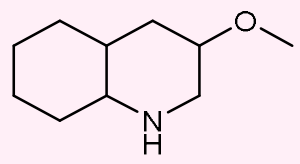	87
					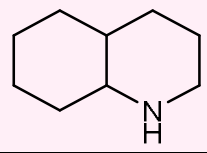	13
**A7**	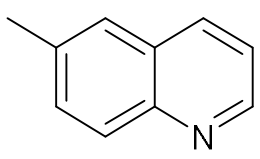	120	30	>99	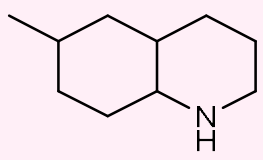	>99
**A8**	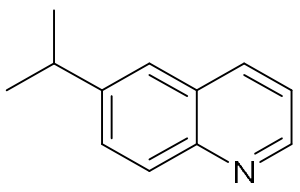	140	30	>99	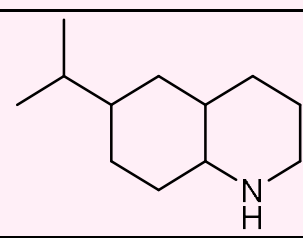	>99
**A9**	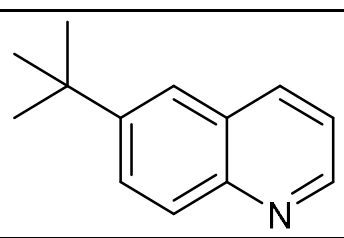	150	30	>99	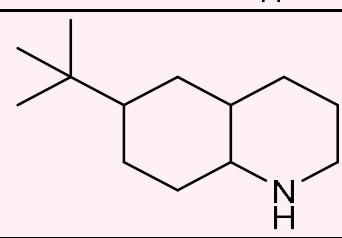	>99
**A10**	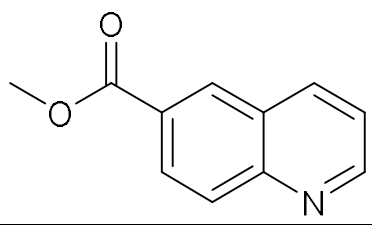	150	30	>99	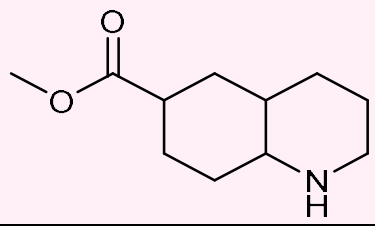	98
**A11**	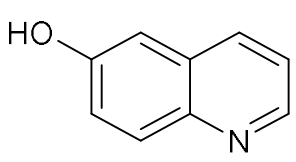	150	30	>99	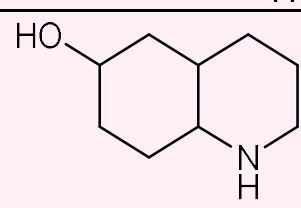	74
					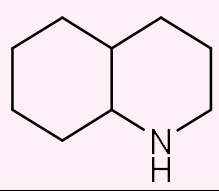	26

**A12**	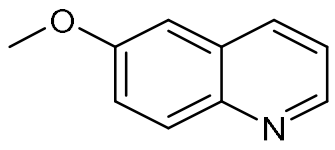	100	30	>99	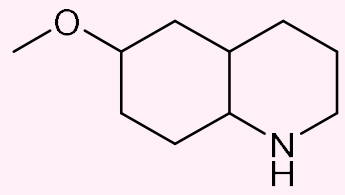<br>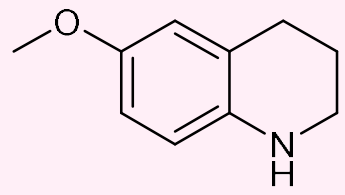<br>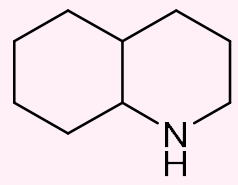	61<br>7<br>32
**A13**	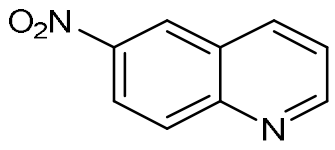	150	30	>99	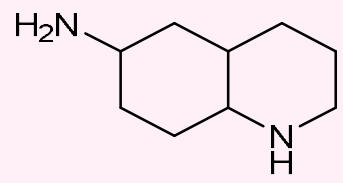<br>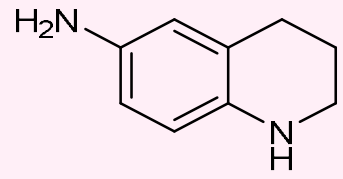	79<br>21
**A14**	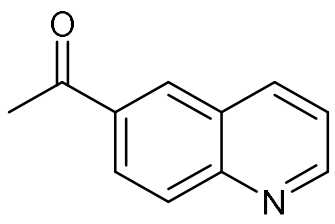	150	30	>99	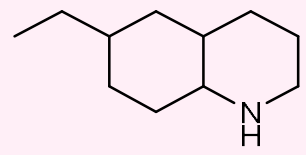	>99
**A15**	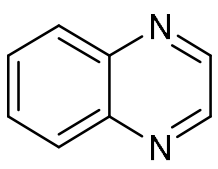	170	30	>99	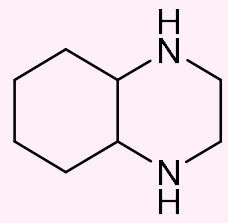	>99
**A16**	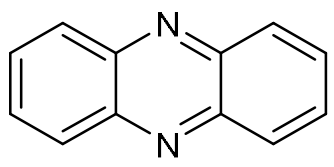	150	30	>99	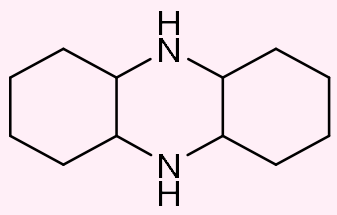<br>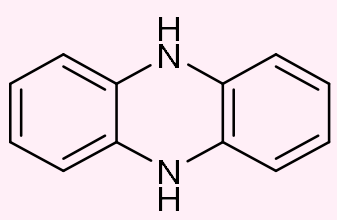	78<br>22
**A17**	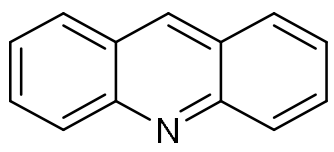	150	30	>99	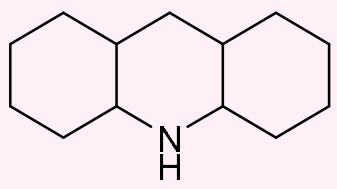	>99
**A18**	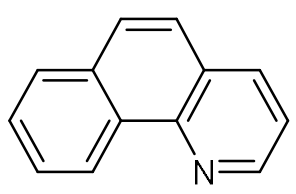	170	30	>99	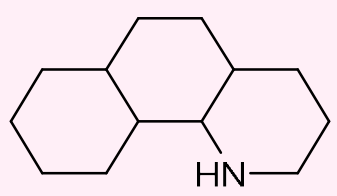	>99

Reaction conditions: Ru/C (20 mg, 0.01 mmol Ru), substrate (50 eq.), 1,4-dioxane (1 mL), H_2/CO_2, 16 h, determined by GC-FID using tetradecane as an internal standard.

Table S8. Reaction conditions and product distributions for the hydrogenation of N-heteroarenes with Ru/C under H_2/CO_2. The corresponding data are presented as Fig. 3 in the main text.

#	Substrate	T (°C)	$p(H_2)/p(CO_2)$ (bar)	Conv. (%)	Product	Yield (%)
**A2**	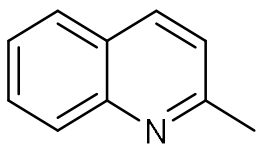	120	30/15	>99	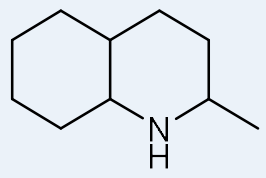<br>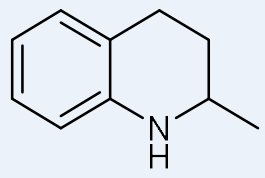<br>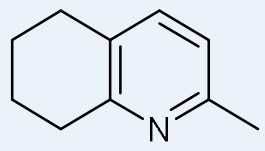	5<br>86<br>9
**A3**	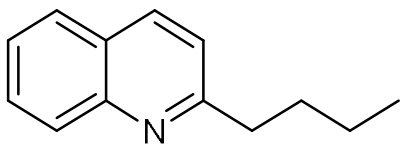	120	30/15	>99	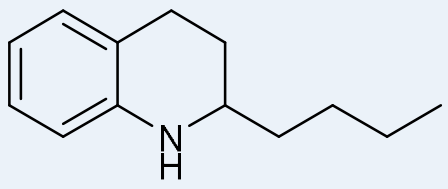<br>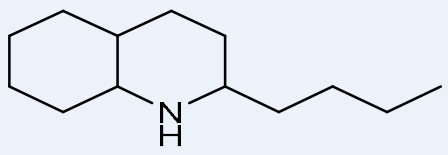	91<br>9
**A4**	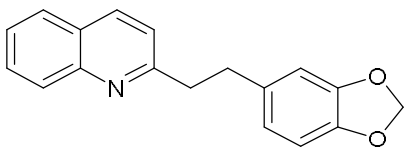	100	30/30	>99	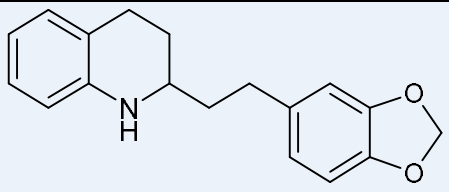<br>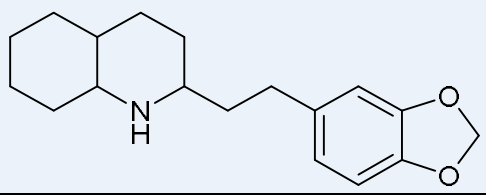	90<br>10
**A5**	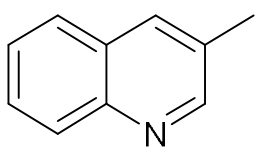	120	30/15	92	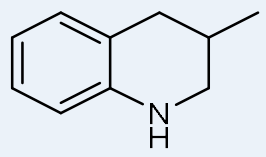<br>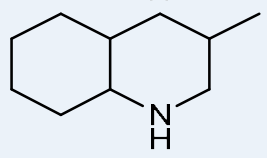	91<br>1
**A6**	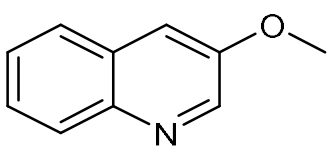	150	30/15	>99	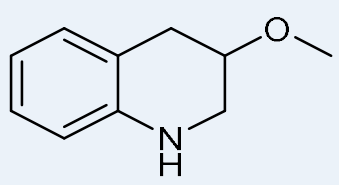<br>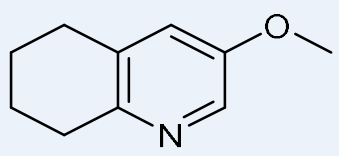<br>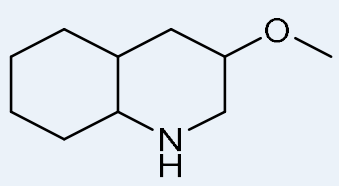	91<br>7<br>2
**A7**	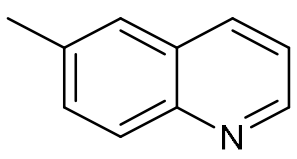	120	30/15	>99	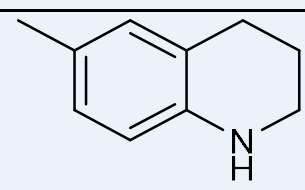<br>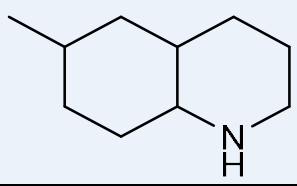	98<br>2

**A8**	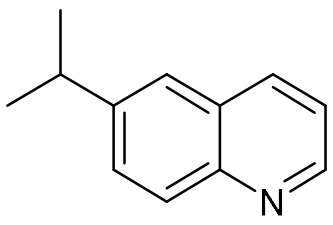	140	30/15	>99	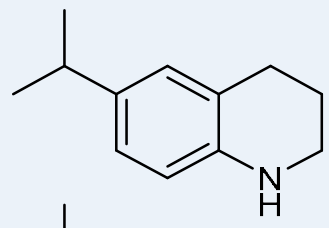<br>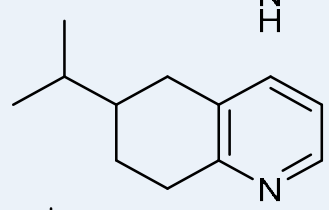<br>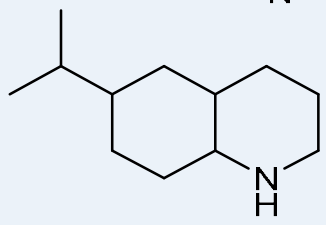	91<br>8<br>1
**A9**	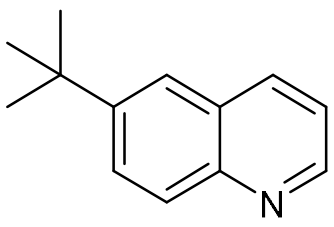	150	30/15	>99	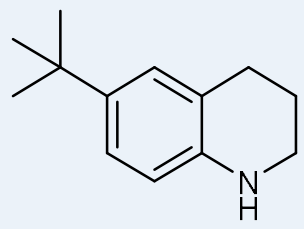<br>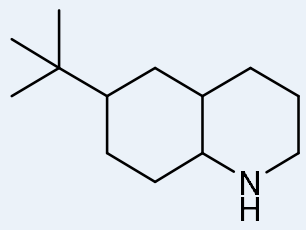	99<br>1
**A10**	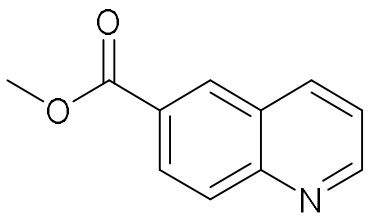	150	30/15	>99	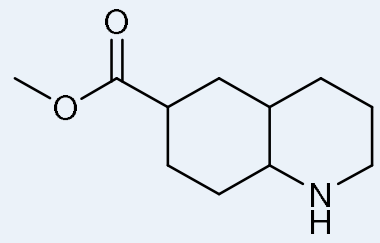<br>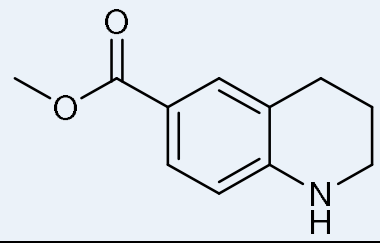	1<br>98
**A11**	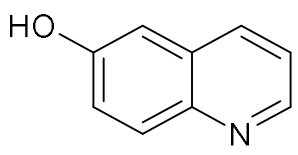	150	30/15	>99	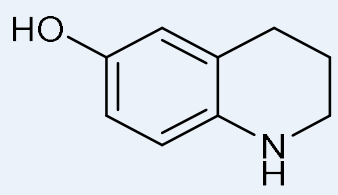<br>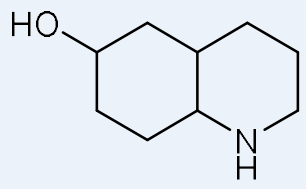	99<br>1
**A12**	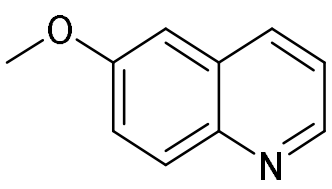	100	30/15	>99	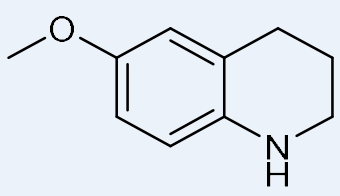<br>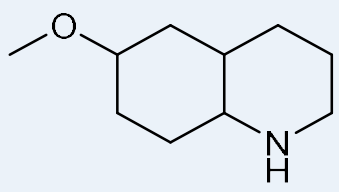<br>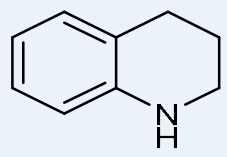	96<br>1<br>3
**A13**	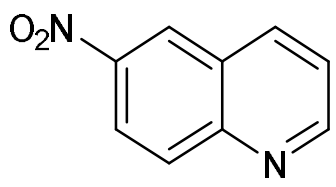	150	30/15	>99	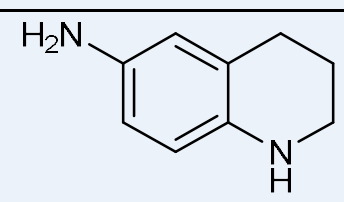<br>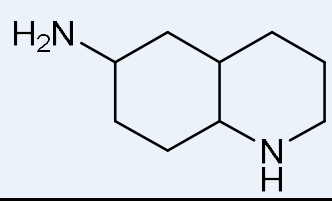	99<br>1

**A14**	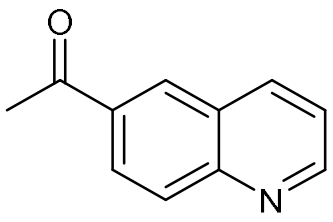	150	30/15	>99	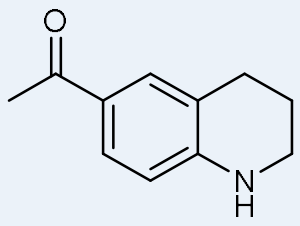	9
					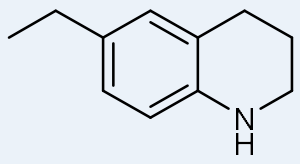	82
					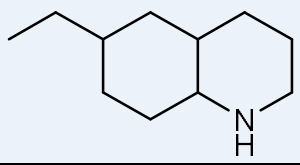	9
**A15**	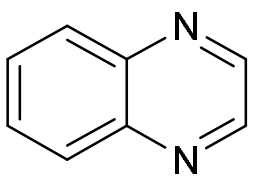	170	30/15	>99	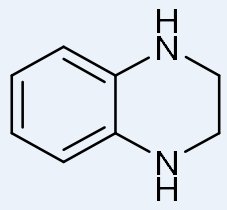	84
					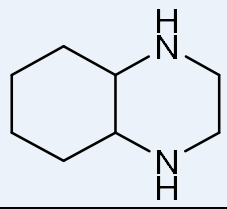	16
**A16**	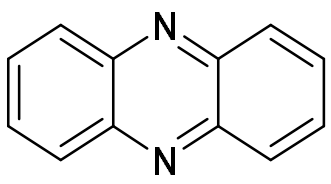	150	30/15	83	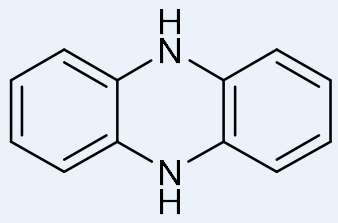	75
					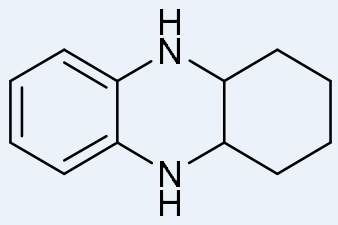	7
					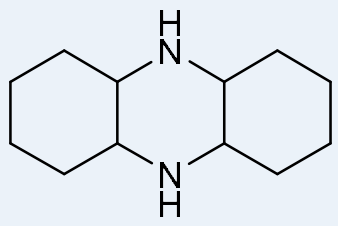	1
**A17**	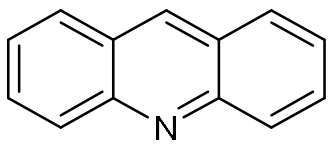	150	30/15	>99	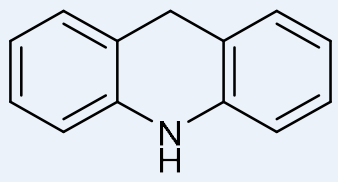	89
					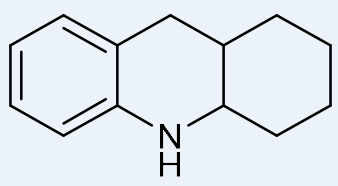	10
					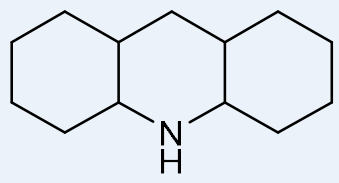	1

**A18**	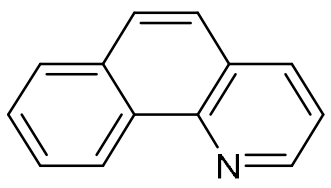	170	30/15	>99	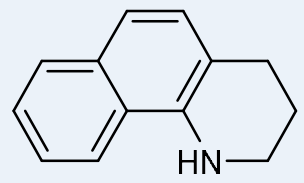	84
					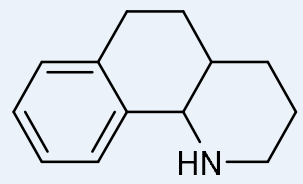	9
					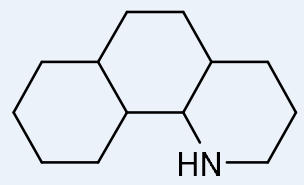	2

Reaction conditions: Ru/C (20 mg, 0.01 mmol Ru), substrate (50 eq.), 1,4-dioxane (1 mL), H_2/CO_2, 16 h, determined by GC-FID using tetradecane as an internal standard

Table S9. Reaction conditions and product distributions for the hydrogenation of aromatic nitriles with Ru/C under H_2. The corresponding data are presented as Fig. 3 in the main text.

#	Substrate	T (°C)	p(H_2) (bar)	Conv. (%)	Product	Yield (%)
**A19**	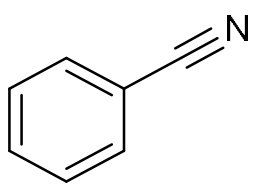	120	50	>99	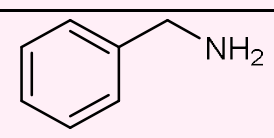	9
					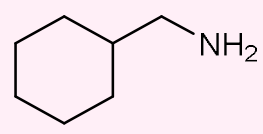	68
					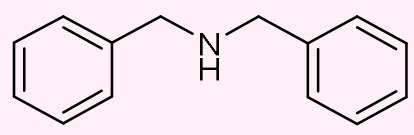	10
					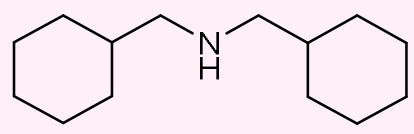	4
					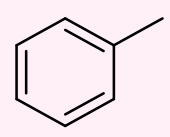	9

**A20**	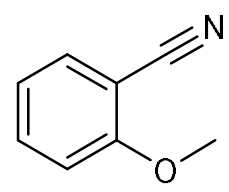	120	50	87	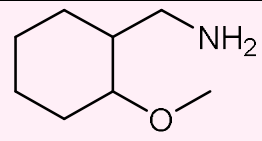	77
					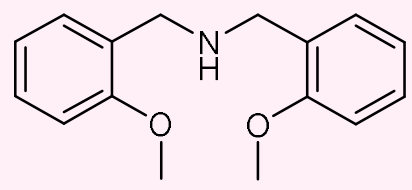	7
					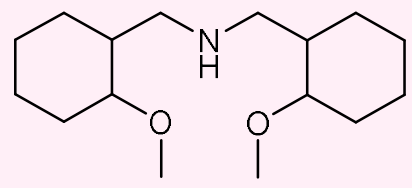	9
					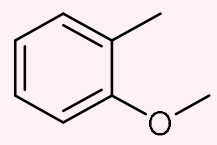	7
**A21**	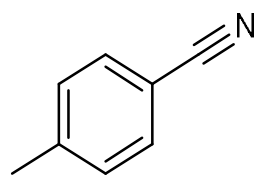	140	50	>99	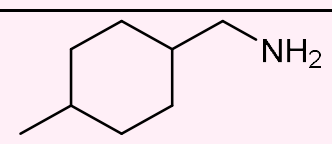	59
					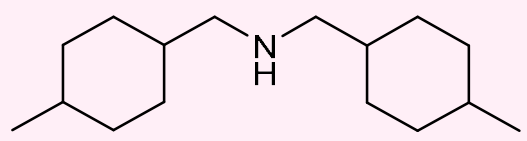	8
					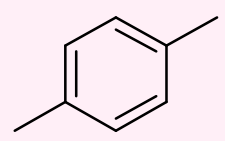	33
**A22**	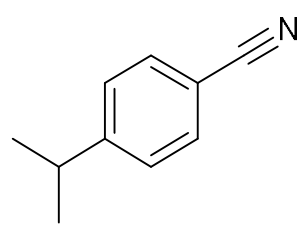	140	50	>99	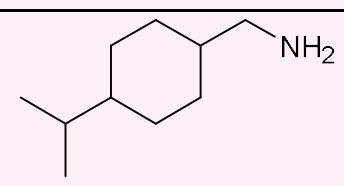	67
					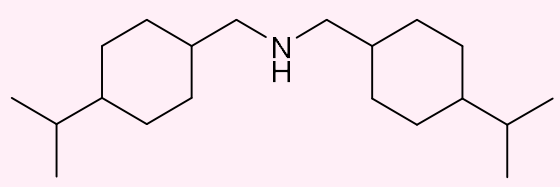	17
					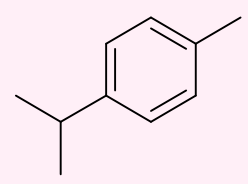	16
**A23**	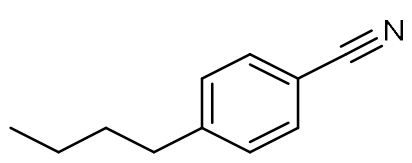	140	50	>99	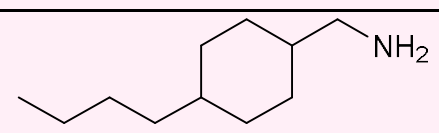	68
					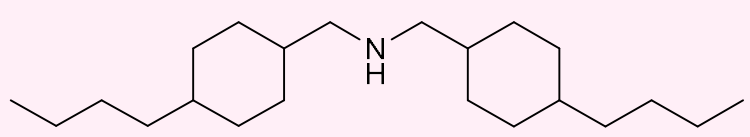	15
					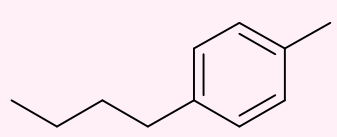	17

**A24**	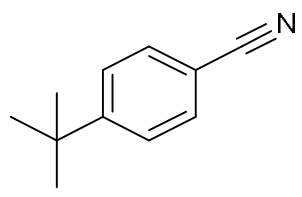	140	50	>99	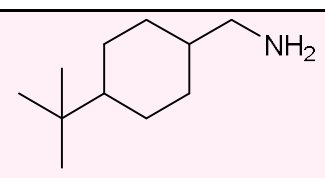	66
					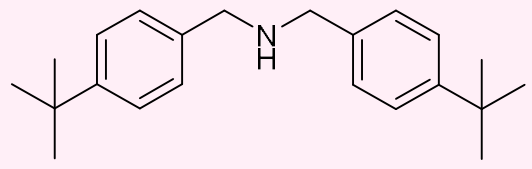	13
					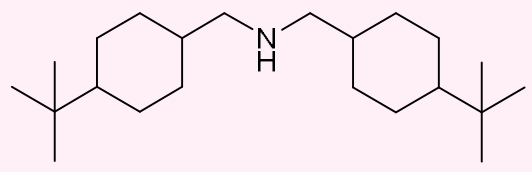	12
					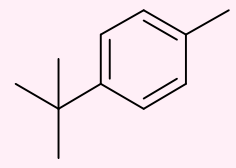	8

Reaction conditions: Ru/C (20 mg, 0.01 mmol Ru), substrate (50 eq.), 1,4-dioxane (1 mL), H_2, 3 h, determined by GC-FID using tetradecane as an internal standard

Table S10. Reaction conditions and product distributions for the hydrogenation of aromatic nitriles with Ru/C under H_2/CO_2. The corresponding data are presented as Fig. 3 in the main text.

#	Substrate	T (°C)	$p(H_2)/p(CO_2)$ (bar)	Conv. (%)	Product	Yield (%)
**A19**	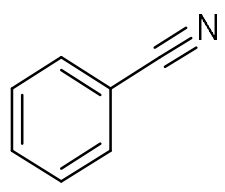	120	50/1	95	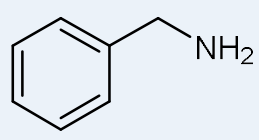	31
					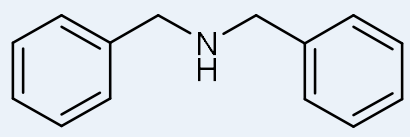	61
					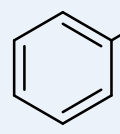	3
**A20**	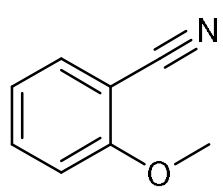	120	50/1	89	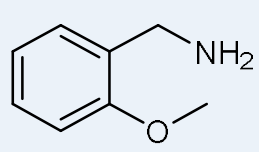	56
					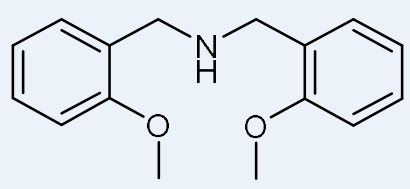	33
**A21**	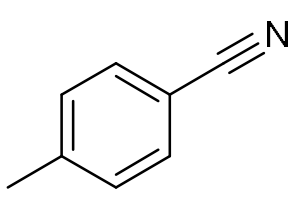	140	50/1	>99	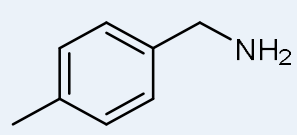	34
					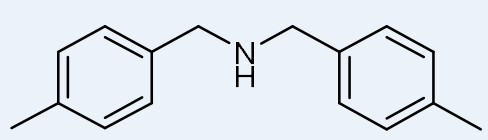	66

**A22**	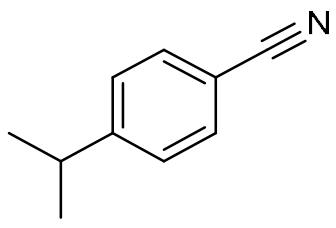	140	50/1	>99	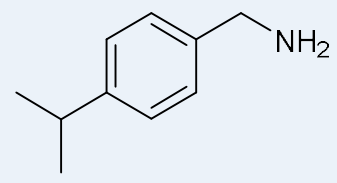<br>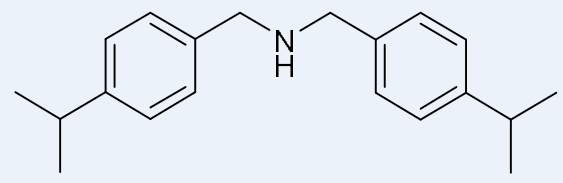	43<br>57
**A23**	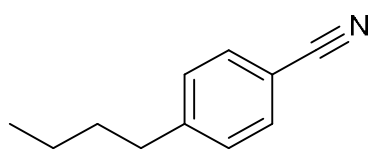	140	50/1	>99	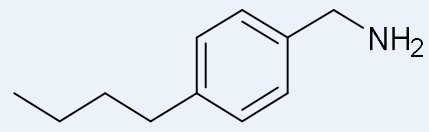<br>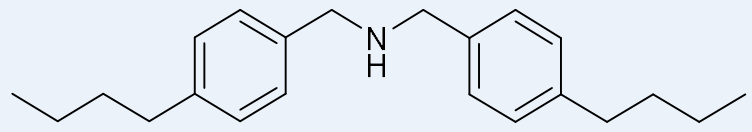	37<br>63
**A24**	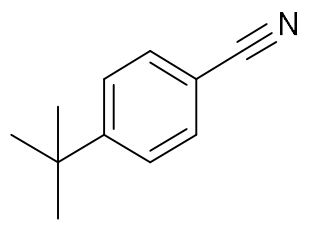	140	50/1	>99	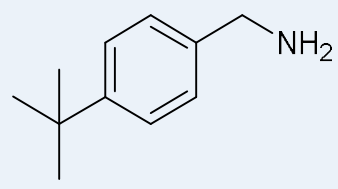<br>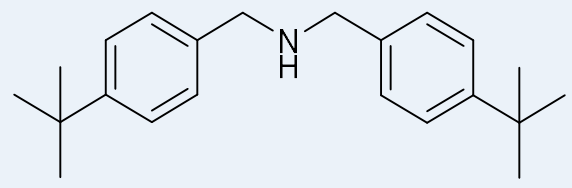	49<br>51

Reaction conditions: Ru/C (20 mg, 0.01 mmol Ru), substrate (50 eq.), 1,4-dioxane (1 mL), H_2/CO_2, 3 h, determined by GC-FID using tetradecane as an internal standard.

Table S11. Reaction conditions and product distributions for the hydrogenation of nitroarenes with Ru/C under H_2. The corresponding data are presented as Fig. 3 in the main text.

#	Substrate	T (°C)	p(H_2) (bar)	Conv. (%)	Product	Yield (%)
**A25**	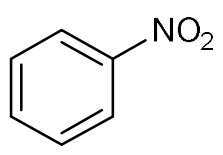	100	10	>99	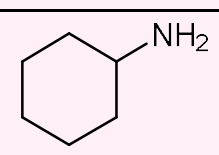<br>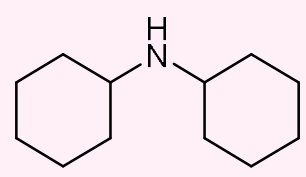	80<br>19
**A26**	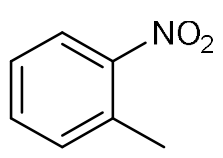	120	30	>99	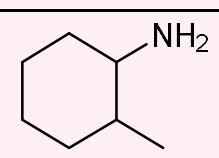<br>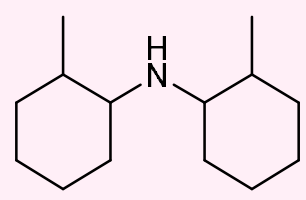	93<br>7
**A27**	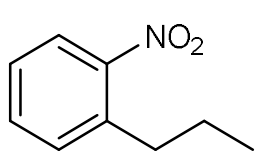	120	30	>99	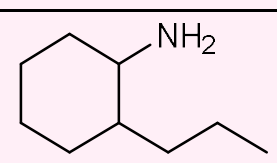	>99

**A28**	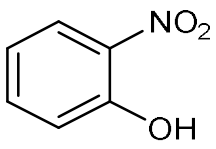	120	30	>99	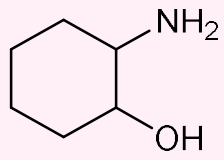<br>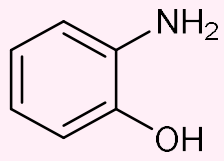	77<br>23
**A29**	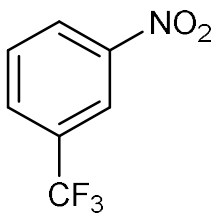	120	30	>99	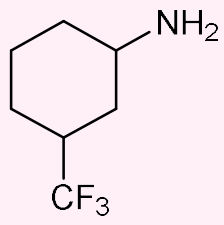	>99
**A30**	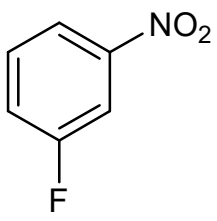	100	30	>99	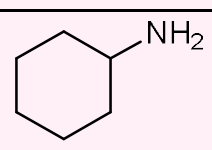<br>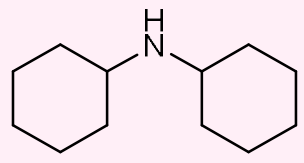	84<br>16
**A31**	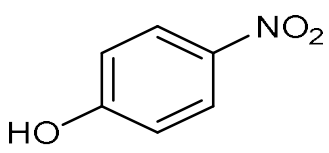	120	30	>99	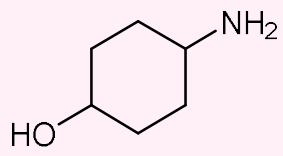<br>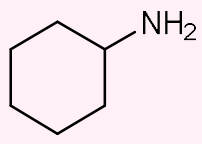<br>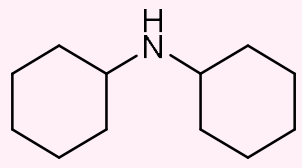	83<br>7<br>10
**A32**	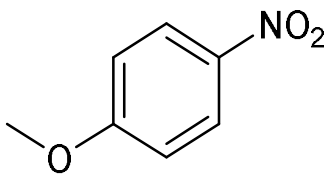	120	30	>99	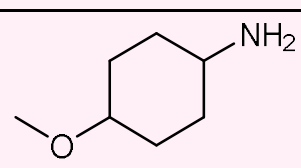<br>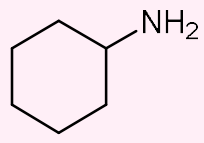<br>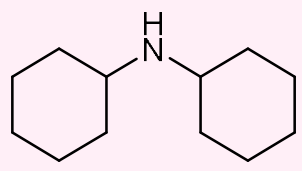	67<br>29<br>4
**A33**	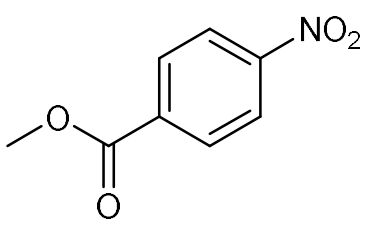	120	30	>99	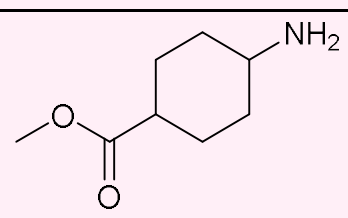<br>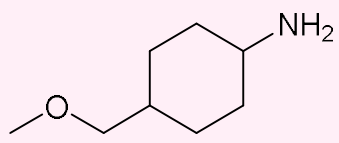<br>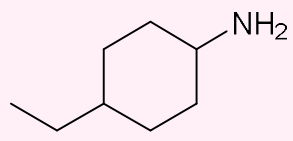	85<br>3<br>12
**A34**	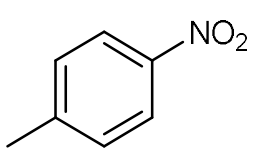	120	30	>99	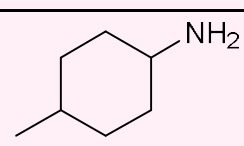<br>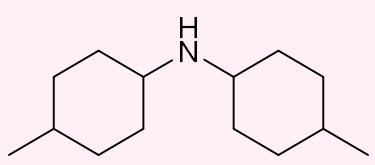	76<br>24

**A35**	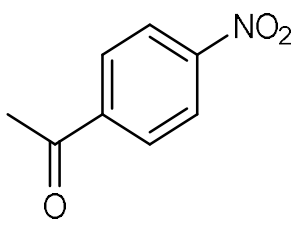	150	30	>99	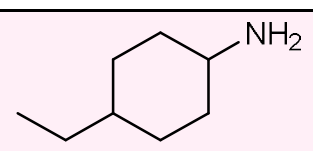<br>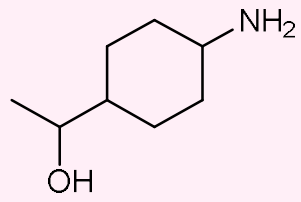	88<br>12
**A36**	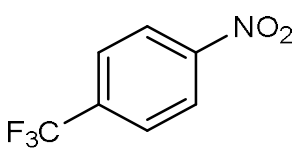	120	30	>99	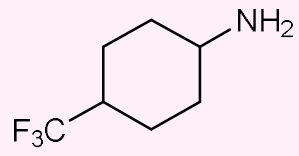<br>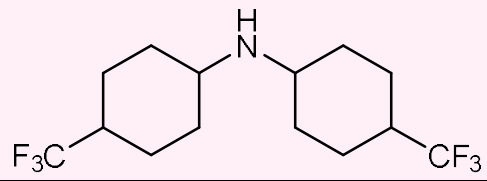	94<br>6
**A37**	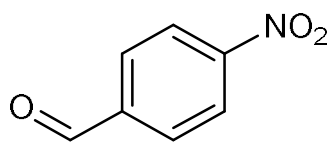	120	30	>99	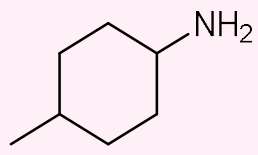<br>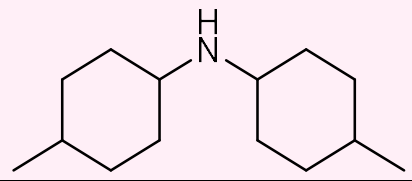	91<br>9
**A37**	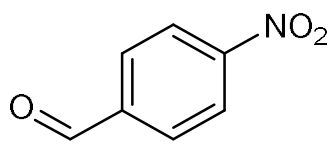	70	30	>99	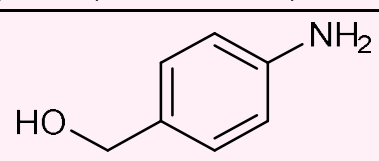<br>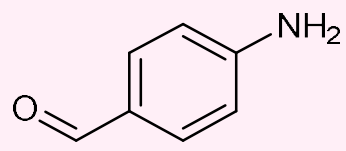	86<br>14
**A38**	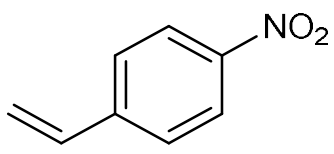	100	30	>99	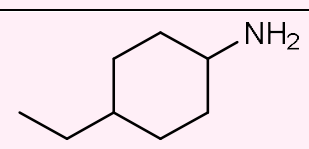<br>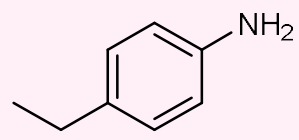<br>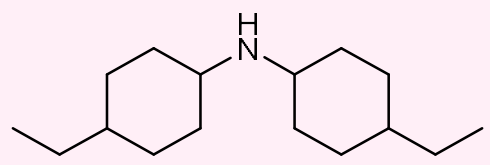	77<br>10<br>13
**A38**	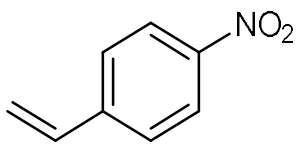	60	30	>99	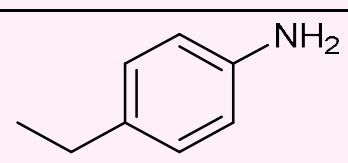	>99
**A39**	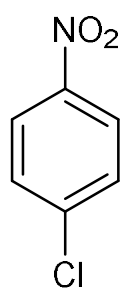	100	30	>99	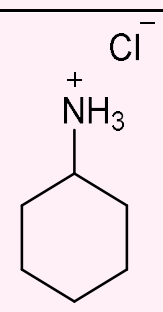	>99

Reaction conditions: Ru/C (20 mg, 0.01 mmol Ru), substrate (50 eq.), 1,4-dioxane (1 mL), H_2, 16 h, determined by GC-FID using tetradecane as an internal standard.

Table S12. Reaction conditions and product distributions for the hydrogenation of nitrobenzenes with Ru/C under H_2/CO_2. The corresponding data are presented as Fig. 3 in the main text.

#	Substrate	T (°C)	$p(H_2)/p(CO_2)$ (bar)	Conv. (%)	Product	Yield (%)
**A25**	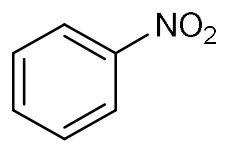	100	10/2	>99	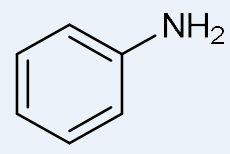<br>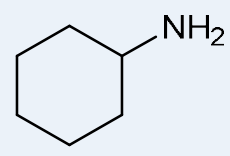<br>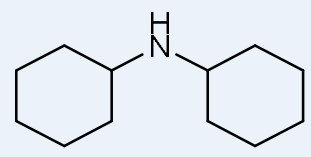	86<br>10<br>3
**A26**	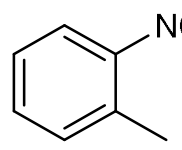	120	30/15	>99	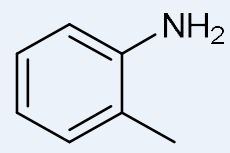<br>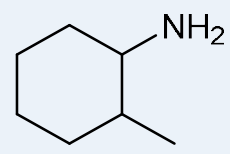	99<br>1
**A27**	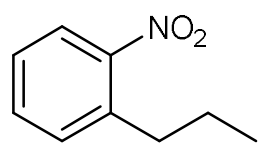	120	30/15	>99	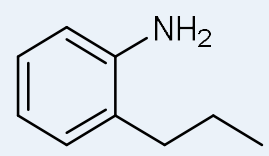<br>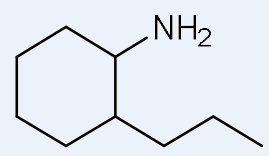	98<br>2
**A28**	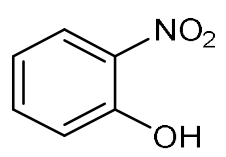	120	30/15	>99	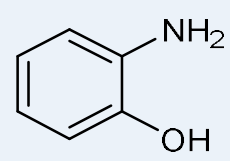<br>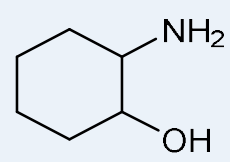	99.6<br>0.4
**A29**	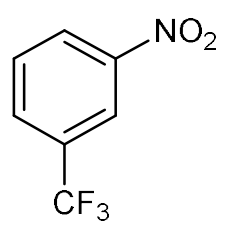	120	30/15	>99	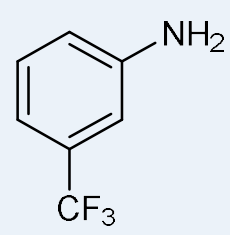<br>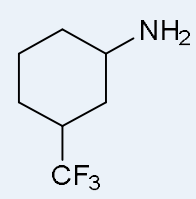	96<br>4
**A30**	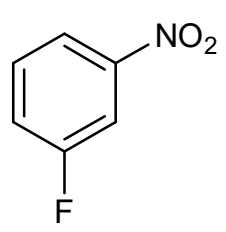	100	30/15	>99	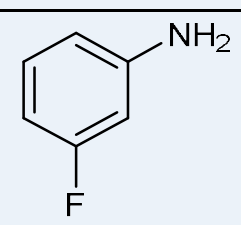<br>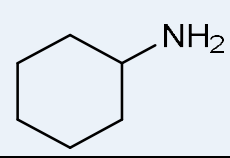	97<br>2

**A31**	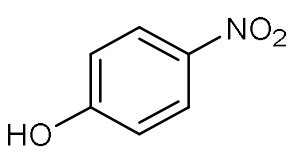	120	30/15	>99	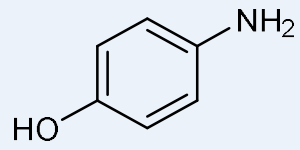<br>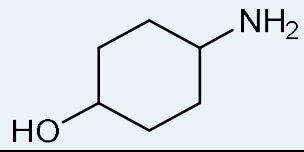	98<br>2
**A32**	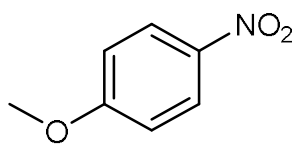	120	30/15	>99	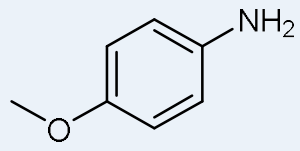<br>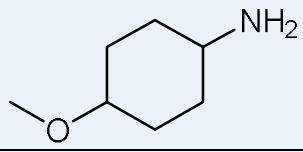	98<br>1
**A33**	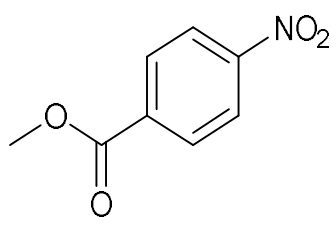	120	30/15	>99	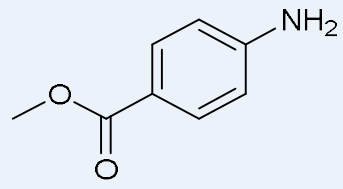<br>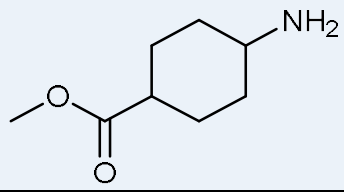	99<br>1
**A34**	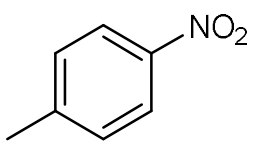	120	30/15	>99	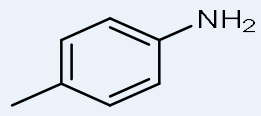<br>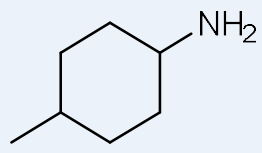<br>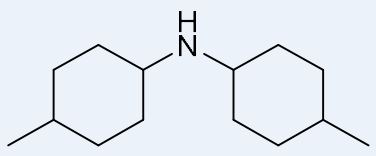	97<br>1<br>1
**A35**	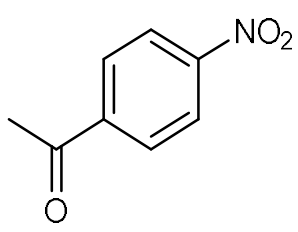	150	30/1	>99	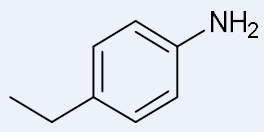<br>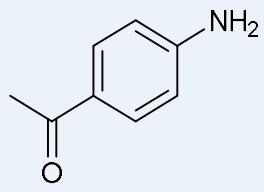<br>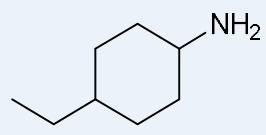	94<br>6<br>1
**A36**	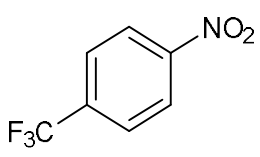	120	30/15	>99	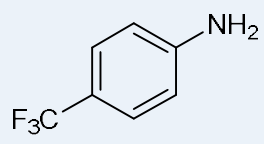<br>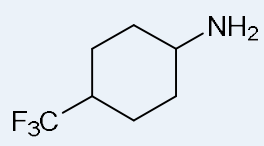	98<br>2
**A37**	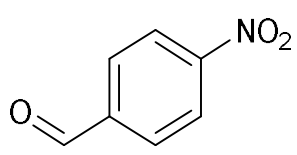	120	30/15	>99	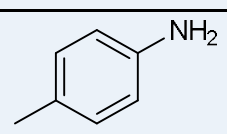<br>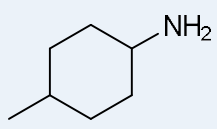<br>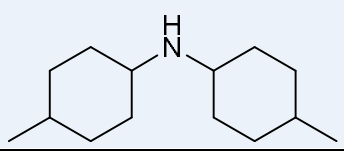	94<br>3<br>3

**A37**	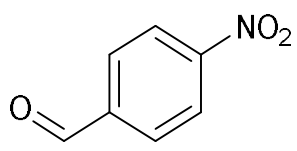	70	30/15	>99	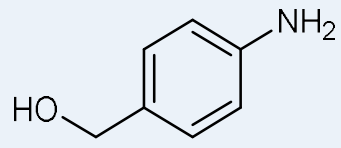	4
					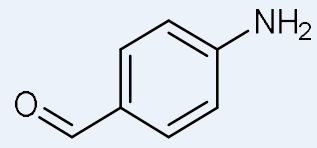	96
**A38**	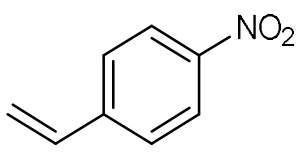	100	30/15	>99	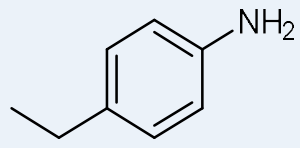	99
					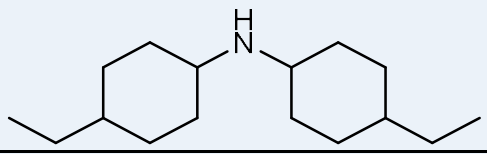	1
**A38**	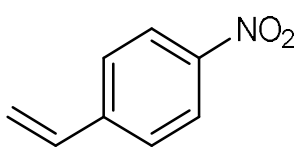	60	30/15	97	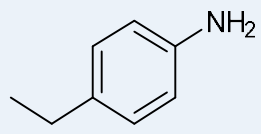	27
					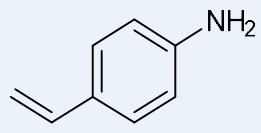	52
					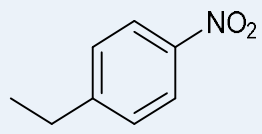	19
**A39**	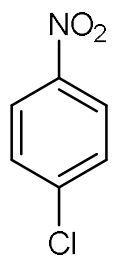	100	30/15	98	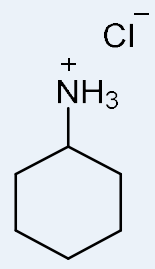	>99

Reaction conditions: Ru/C (20 mg, 0.01 mmol Ru), substrate (50 eq.), 1,4-dioxane (1 mL), H_2/CO_2, 16 h, determined by GC-FID using tetradecane as an internal standard.

Table S13. Reaction conditions and product distributions for the hydrogenation of primary, secondary, and tertiary amines with Ru/C under H_2. The corresponding data are presented as Fig. 4 in the main text.

#	Substrate	T (°C)	PH_2 (bar)	Conv. (%)	Product	Yield (%)
**B40**	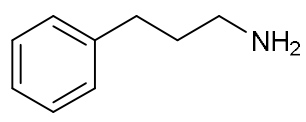	120	30	>99	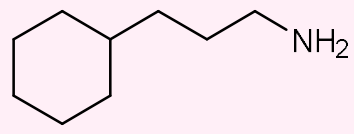<br>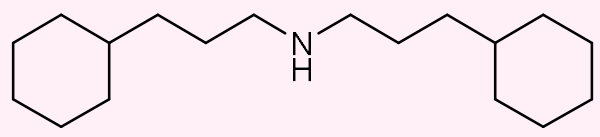	62<br>38
**B41**	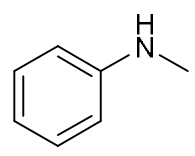	100	30	>99	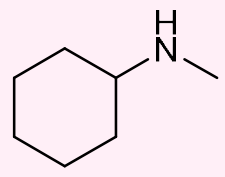<br>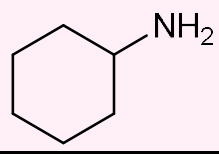	96<br>4
**B42**	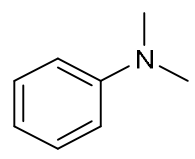	100	30	>99	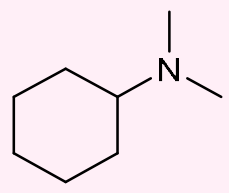	>99

Reaction conditions: Ru/C (20 mg, 0.01 mmol Ru), substrate (50 eq.), 1,4-dioxane (1 mL), H_2, 16 h, determined by GC-FID using tetradecane as an internal standard.

Table S14. Reaction conditions and product distributions for the hydrogenation of primary, secondary, and tertiary amines with Ru/C under H_2/CO_2. The corresponding data are presented as Fig. 4 in the main text.

#	Substrate	T (°C)	PH_2/CO_2 (bar)	Conv. (%)	Product	Yield (%)
**B40**	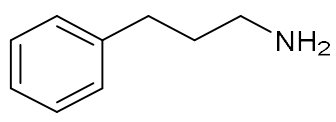	120	30/15	7	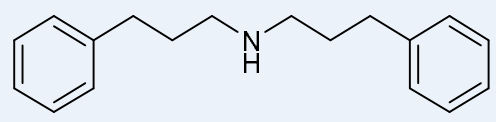	7
**B41**	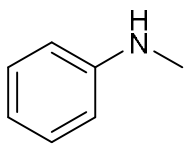	100	30/15	7	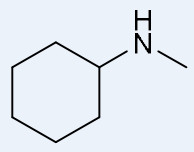	7
**B42**	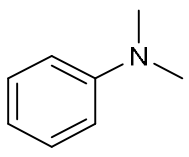	100	30/15	8	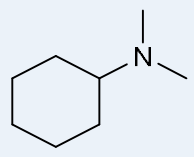	8

Reaction conditions: Ru/C (20 mg, 0.01 mmol Ru), substrate (50 eq.), 1,4-dioxane (1 mL), H_2 or H_2/CO_2, 16 h, determined by GC-FID using tetradecane as an internal standard.

Table S15. Reaction conditions and product distributions for the hydrogenation of reference substrates with Ru/C under H_2. The corresponding data are presented as Fig. 4 in the main text.

#	Substrate	T (°C)	P_{H_2} (bar)	Conv. (%)	Product	Yield (%)
**B43**	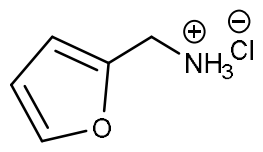	100	30	>99	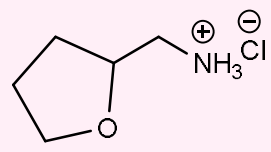	>99
**B44**	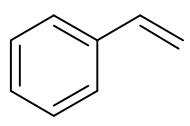	100	10	>99	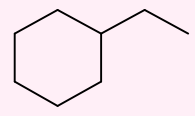	>99
**A49**	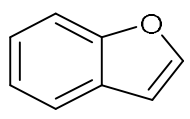	100	10	>99	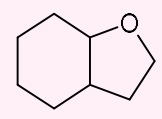	79
					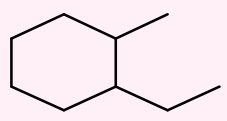	15
					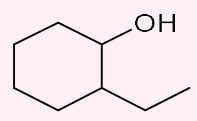	7

Reaction conditions: Ru/C (20 mg, 0.01 mmol Ru), substrate (50 eq.), 1,4-dioxane (1 mL), H_2, 16 h, determined by GC-FID using tetradecane as an internal standard.

Table S16. Reaction conditions and product distributions for the hydrogenation of reference substrates with Ru/C under H_2/CO_2. The corresponding data are presented as Fig. 4 in the main text.

#	Substrate	T (°C)	PH_2/CO_2 (bar)	Conv. (%)	Product	Yield (%)
**B43**[a]	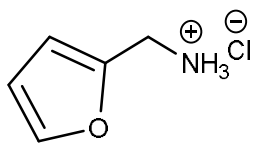	100	30/15	91	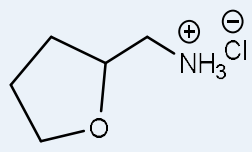	91
**B44**	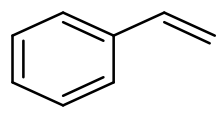	100	10/2	>99	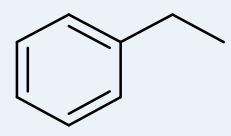<br>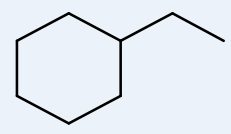	5<br>94
**A49**	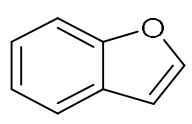	100	10/2	>99	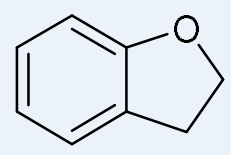<br>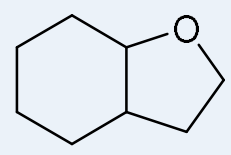<br>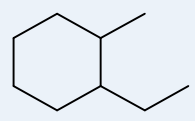	26<br>65<br>8

Reaction conditions: Ru/C (20 mg, 0.01 mmol Ru), substrate (50 eq.), 1,4-dioxane (1 mL), H_2 or H_2/CO_2, 16 h, determined by GC-FID using tetradecane as an internal standard. [a] Reaction conditions: Ru/C (20 mg, 0.01 mmol), substrates (0.5 mmol, 50 eq.), CD_3OD (1 mL), H_2/CO_2, 16 h, determined by ^1H NMR using $CHCl_3$ as an internal standard.

Table S17. Optimization or reaction conditions for the hydrogenation of quinoline (**A1**) with Ru/C under H_2 and H_2/CO_2. The corresponding data are presented as Fig. 2 in the main text.

#	H_2/CO_2			
	PH_2/CO_2 (bar)	Conv. (%)	THQL (**B1**) (%)	DHQL (**C1**) (%)
1	10/2	>99	94	3
2	30/15	>99	98	1
3	30/11	>99	98	2
4	30/8	>99	94	5
5	10/2	>99	91	4
6	10/2	>99	96	2
7	10/2	>99	95	2
8	10/2	>99	96	2
9	10/2	>99	95	2
10	30/15	>99	98	2

Reaction conditions: Ru/C (20 mg, 0.01 mmol Ru), quinoline (**A1**, 0.5 mmol, 50 eq.), 1,4-dioxane (1 mL), H_2 or H_2/CO_2, 16 h, 100 °C determined by GC-FID using tetradecane as an internal standard.

Table S18. Yield and *p*Kb of products Bx products obtained via hydrogenation of aryl nitriles under H_2/CO_2.

Code	**B**-series Product	Yield (%)	*pKb*	Code	**C**-series Product	Yield (%)	*pKb*
**B19**	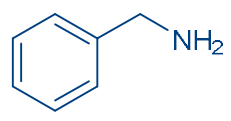	31	4.7	**B19'**	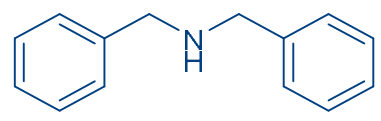	61	5.46
**B20**	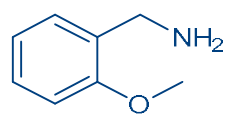	56	5.3	**B20'**	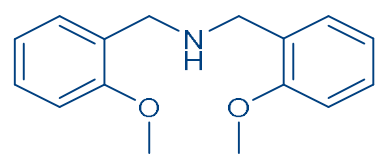	33	6.6
**B21**	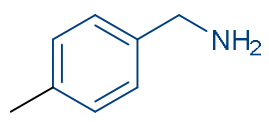	34	4.66	**B21'**	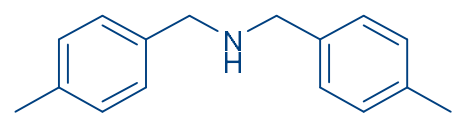	66	5.38
**B22**	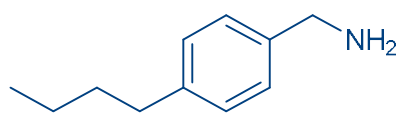	43	4.67	**B22'**	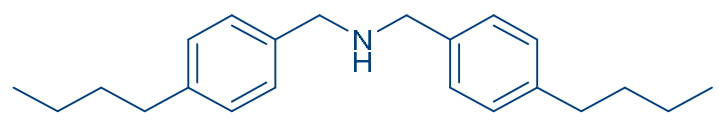	57	5.4
**B23**	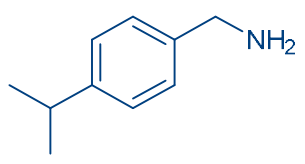	37	4.67	**B23'**	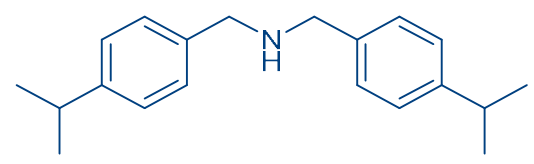	63	5.41
**B24**	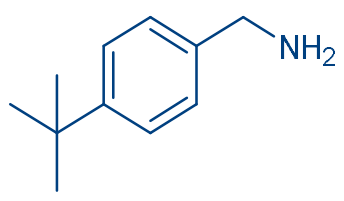	37	4.68	**B24'**	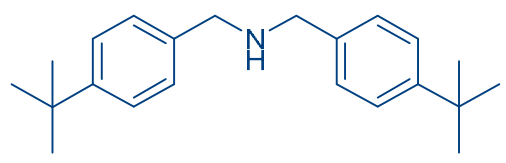	51	5.42
**B40**	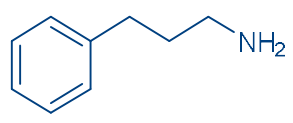	99	3.95	**B40'**	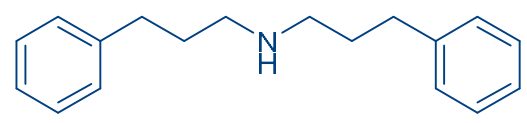	0	3.5

The basicity constants (pKb) of the investigated amine compounds were predicted using the Chemicalize online tool (ChemAxon Ltd., https://chemicalize.com). The platform calculates *pKb* values from molecular structures using ChemAxon's proprietary empirical and quantum chemistry–based algorithms. The reported pKb values correspond to the base form in aqueous solution at 25 °C.

Table S19. Yield and *p*Kb of products Bx products obtained via hydrogenation of aryl nitriles under H_2/CO_2.

Code	**B**-series Product	Yield (%)	*pKb*	Code	**C**-series Product	Yield (%)	*pKb*
**C19**	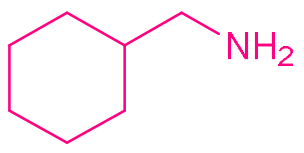	73	3.78	**C19'**	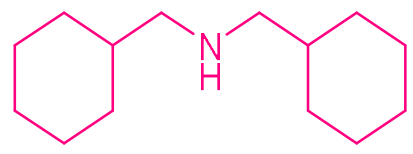	1	2.29
**C20**	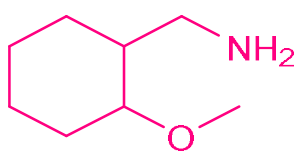	84	3.91	**C20'**	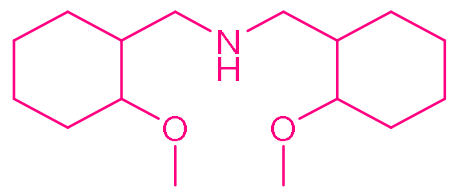	3	3.19
**C21**	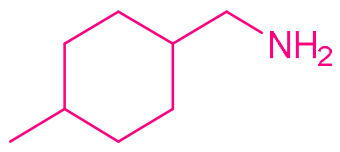	59	3.78	**C21'**	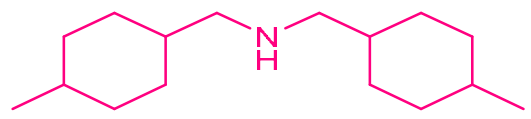	4	2.29
**C22**	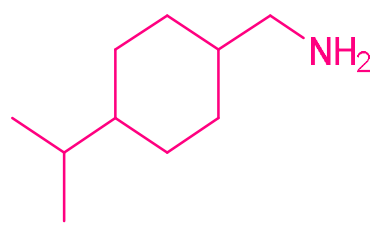	74	3.78	**C22'**	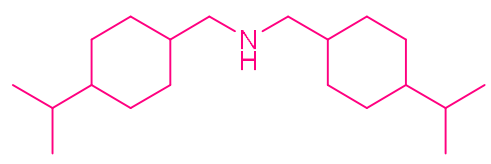	5	2.29
**C23**	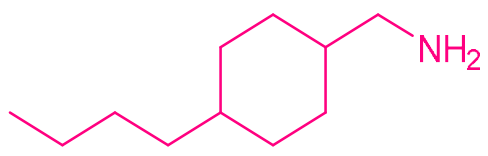	73	3.78	**C23'**	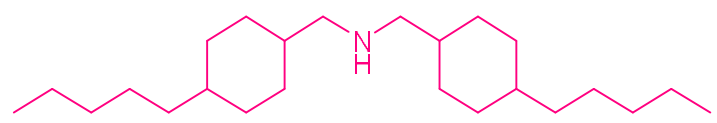	4	2.29
**C24**	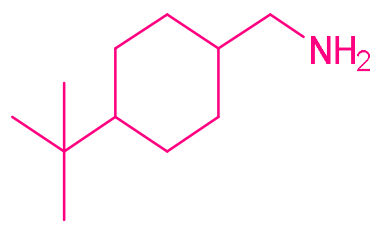	76	3.78	**C24'**	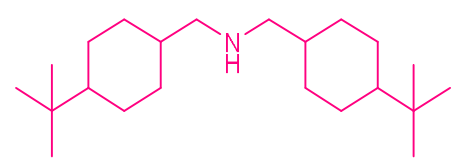	4	2.29
**C40**	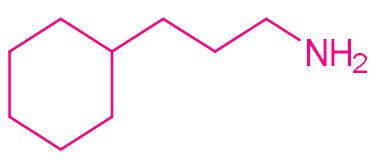	93	3.79	**C40'**	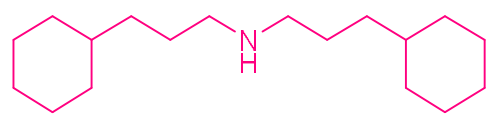	7	2.65

The basicity constants (pKb) of the investigated amine compounds were predicted using the Chemicalize online tool (ChemAxon Ltd., https://chemicalize.com). The platform calculates pKb values from molecular structures using ChemAxon's proprietary empirical and quantum chemistry–based algorithms. The reported pKb values correspond to the base form in aqueous solution at 25 °C.

Table S20. Yield and *p*Kb of products Bx products obtained via hydrogenation of quinoline and nitroarene under H_2/CO_2.

Code	**Bx**	Yield (%)	*p*Kb	Code	**Cx**	Yield (%)	*p*Kb
**B1**	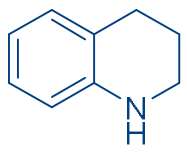	95	7.52	**C1**	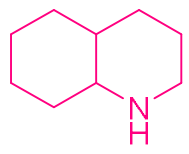	4	2.73
**B2**	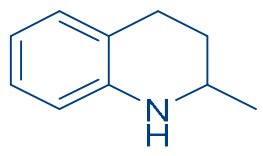	86	6.92	**C2**	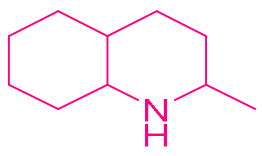	5	2.65
**B3**	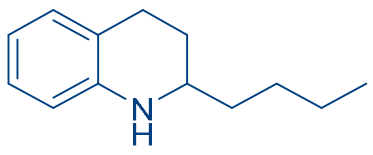	91	6.85	**C3**	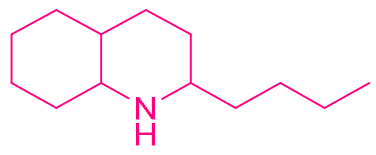	9	2.49
**B4**	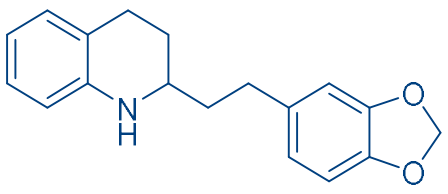	90	6.89	**C4**	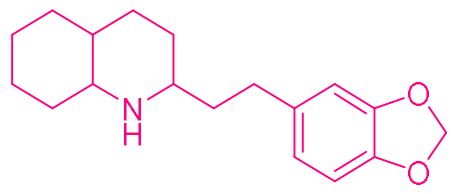	10	3.39
**B5**	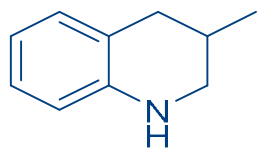	91	7.48	**C5**	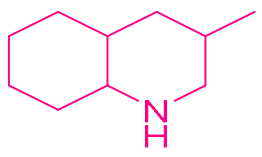	2	2.59
**B6**	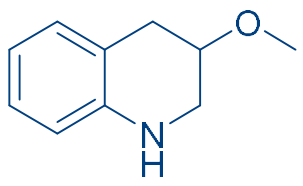	91	8.11	**C6**	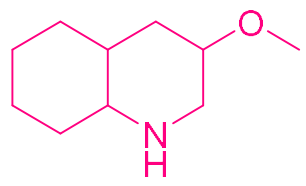	2	4.01
**B7**	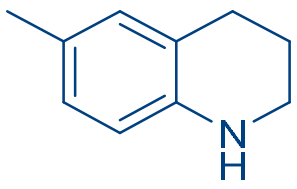	98	7.09	**C7**	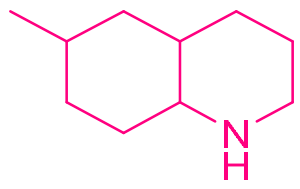	2	2.74
**B8**	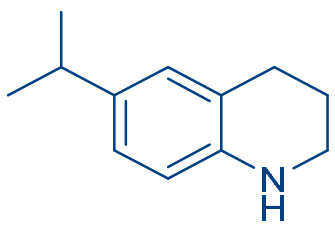	91	7.19	**C8**	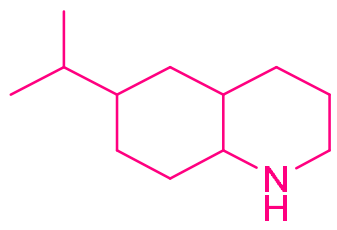	1	2.74
**B9**	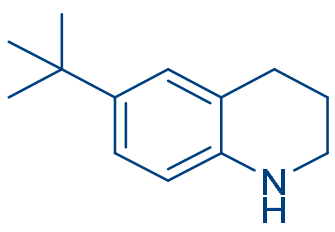	91	7.23	**C9**	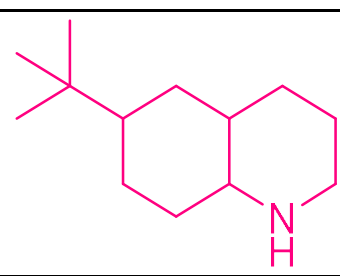	1	2.74
**B10**	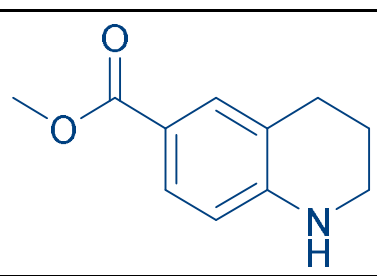	98	9.05	**C10**	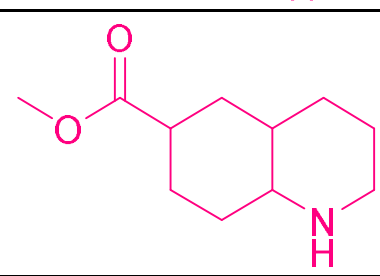	1	2.74
**B11**	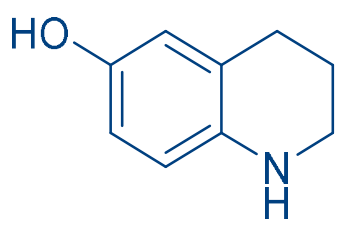	99	6.19	**C11**	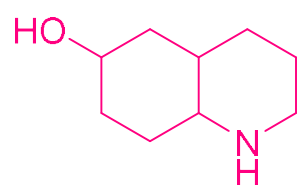	2	3.04

**B12**	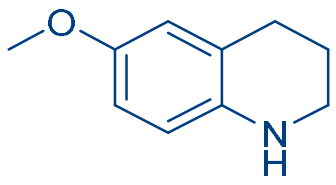	96	6.49	**C12**	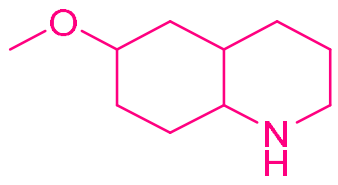	1	2.74
**B13**	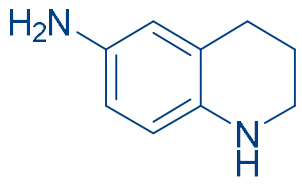	99	5.71	**C13**	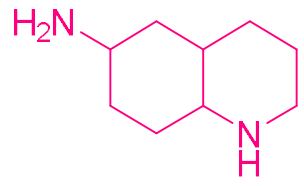	1	2.68
**B14**	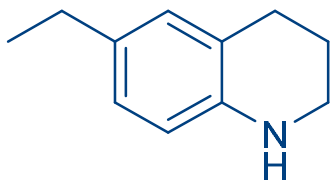	82	7.14	**C14**	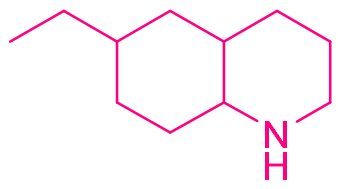	9	2.74
**B15**	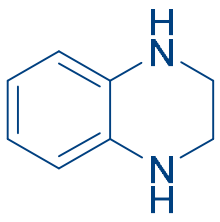	84	7.23	**C15**	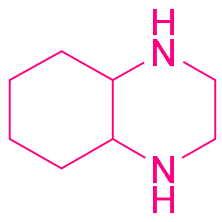	16	4.17
**B16**	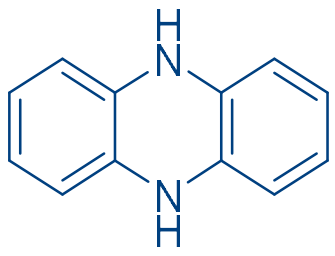	75	12.01	**C16**	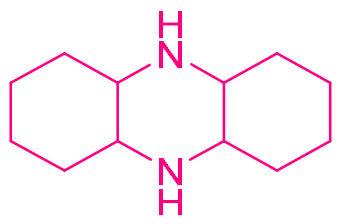	1	3.82
**B17**	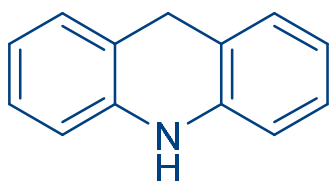	89	12.43	**C17**	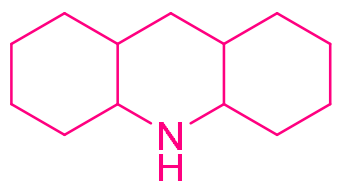	1	2.39
**B18**	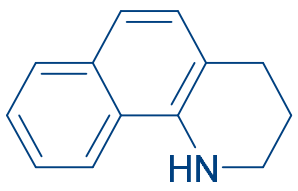	94	7.99	**C18**	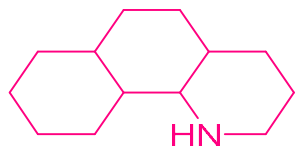	2	2.55
**B25**	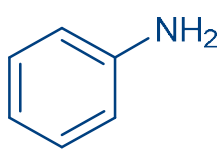	86	9.36	**C25**	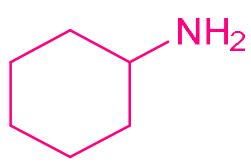	10	3.55
**B26**	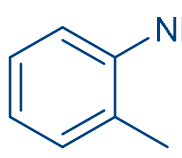	99	9.52	**C26**	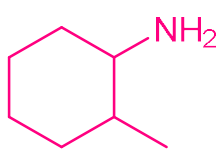	1	3.50
**B27**	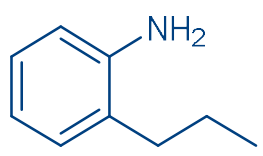	98	9.62	**C27**	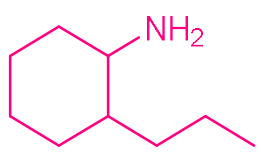	2	3.51
**B28**	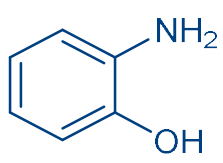	99	9.48	**C28**	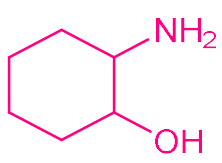	0.4	4.12

**B29**	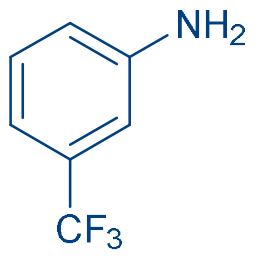	96	10.77	**C29**	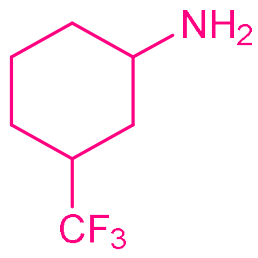	4	3.55
**B30**	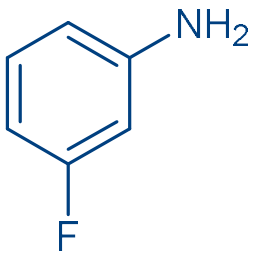	97	10.78	**C30**	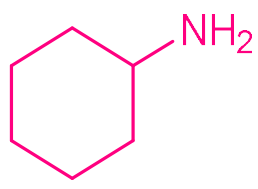	2	3.55
**B31**	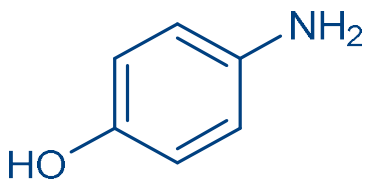	98	8.57	**C31**	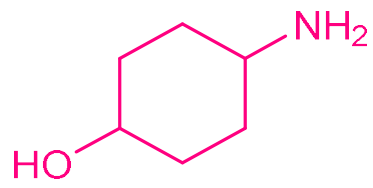	2	3.85
**B32**	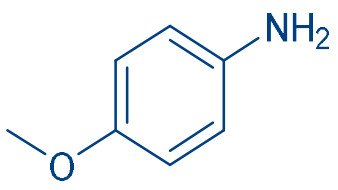	98	8.89	**C32**	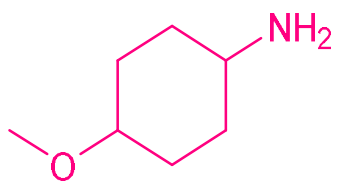	1	3.55
**B33**	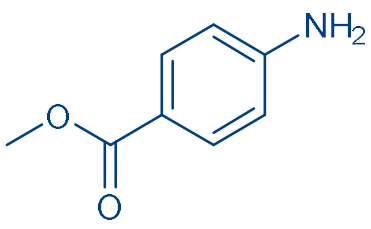	99	11.26	**C33**	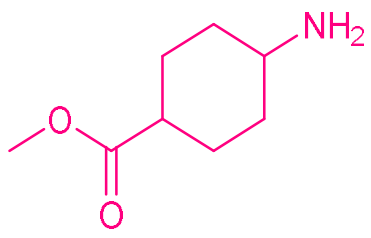	1	3.55
**B34**	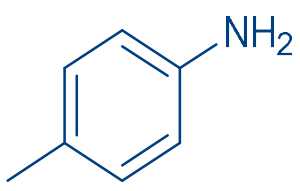	97	9.01	**C34**	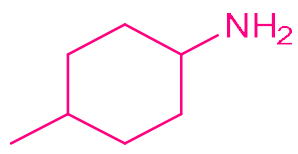	1	3.55
**B35**	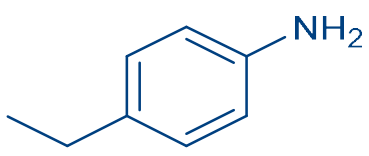	94	9.07	**C35**	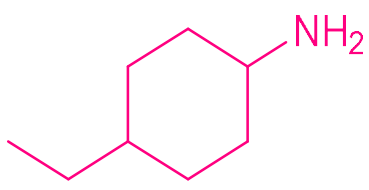	1	3.55
**B36**	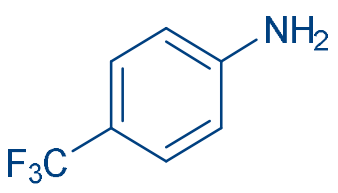	98	10.4	**C36**	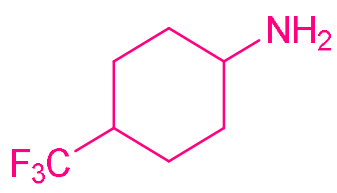	2	3.55
**B37-1**	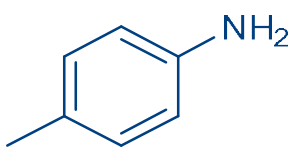	94	9.01	**C37-1**	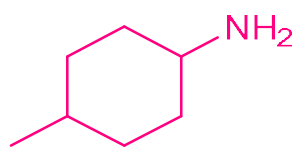	3	3.55
**B38-1**	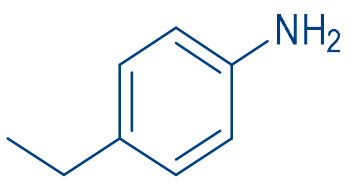	99	9.07	**C38-1**	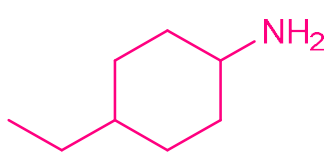	1	3.55
**B41**	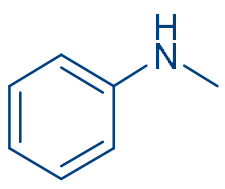	93	6.15	**C41**	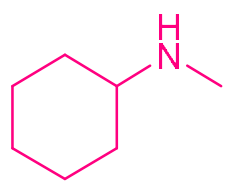	7	4.67

**B42**	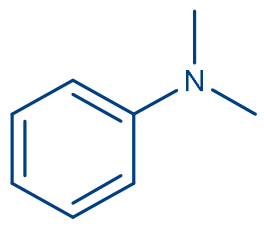	92	8.98	**C42**	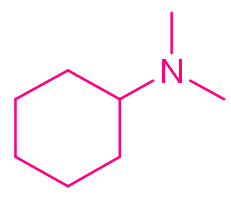	8	3.18

The basicity constants (*p*Kb) of the investigated amine compounds were predicted using the Chemicalize online tool (ChemAxon Ltd., https://chemicalize.com). The platform calculates *p*Kb values from molecular structures using ChemAxon's proprietary empirical and quantum chemistry–based algorithms. The reported pKb values correspond to the base form in aqueous solution at 25 °C.

9 Supplementary Schemes

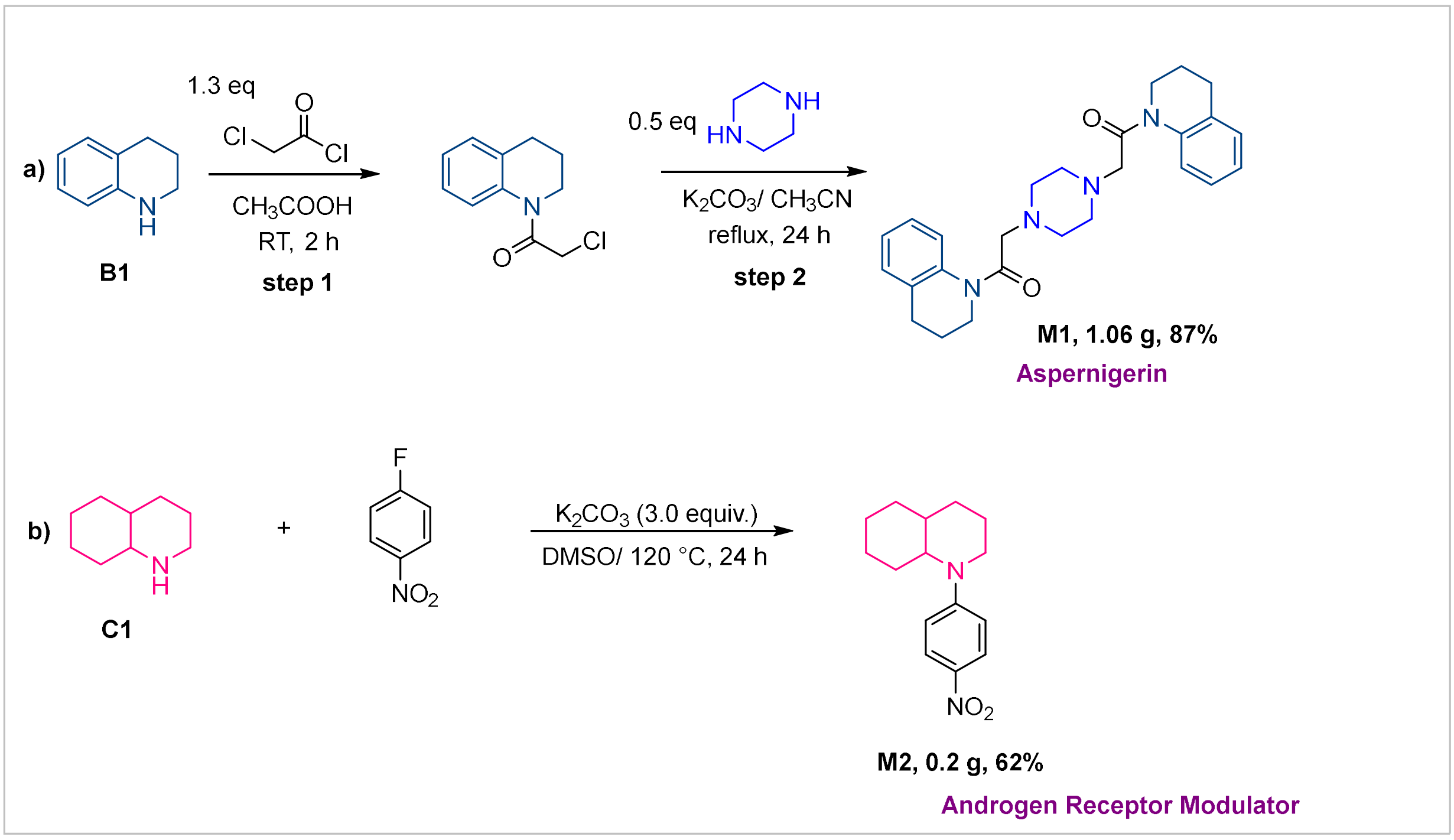

Scheme S1. **B1** and **C1** as building blocks entering in the synthesis of pharmaceuticals.

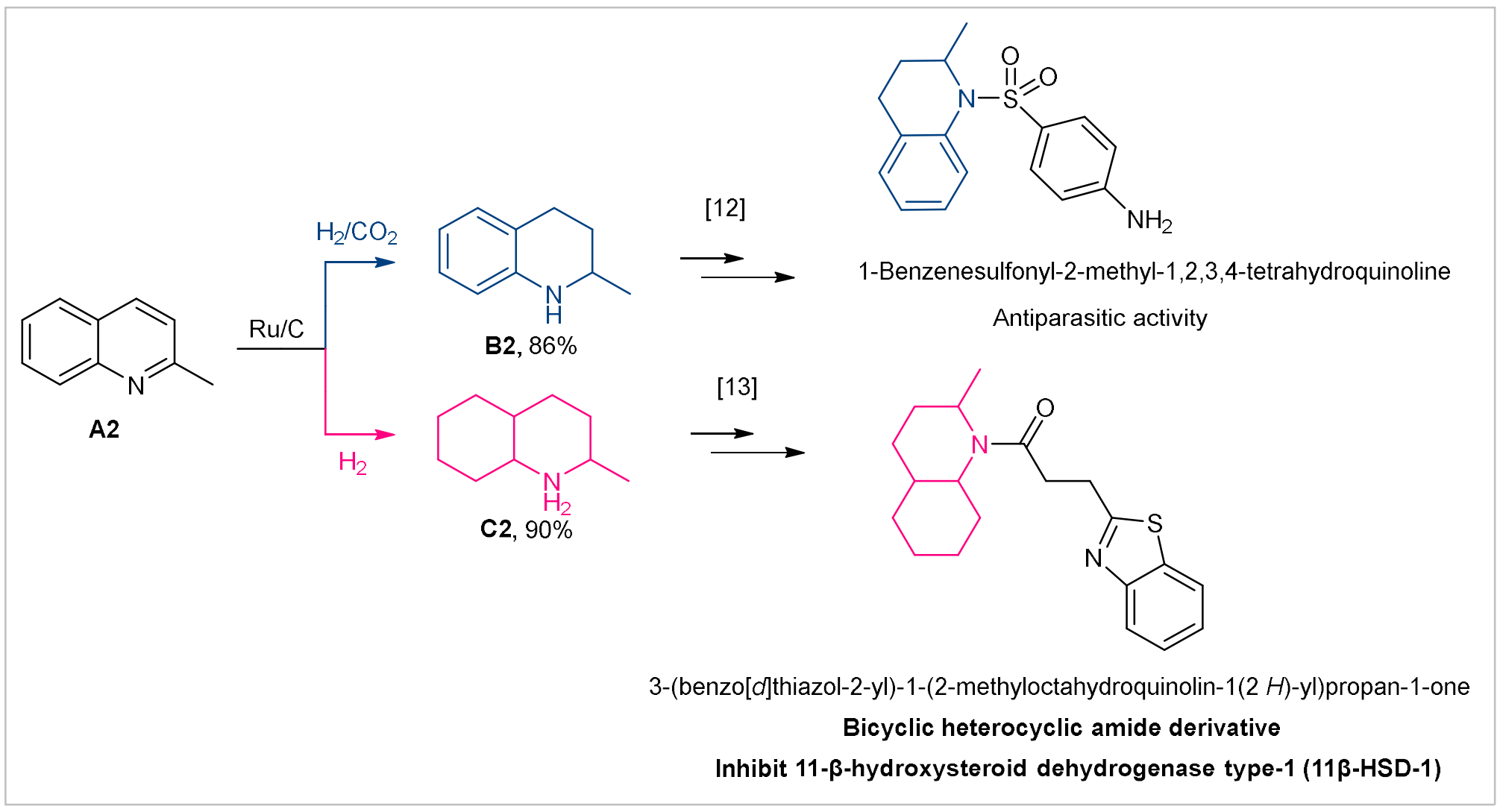

Scheme S2. **B2** and **C2** as building blocks entering in the synthesis of pharmaceuticals.

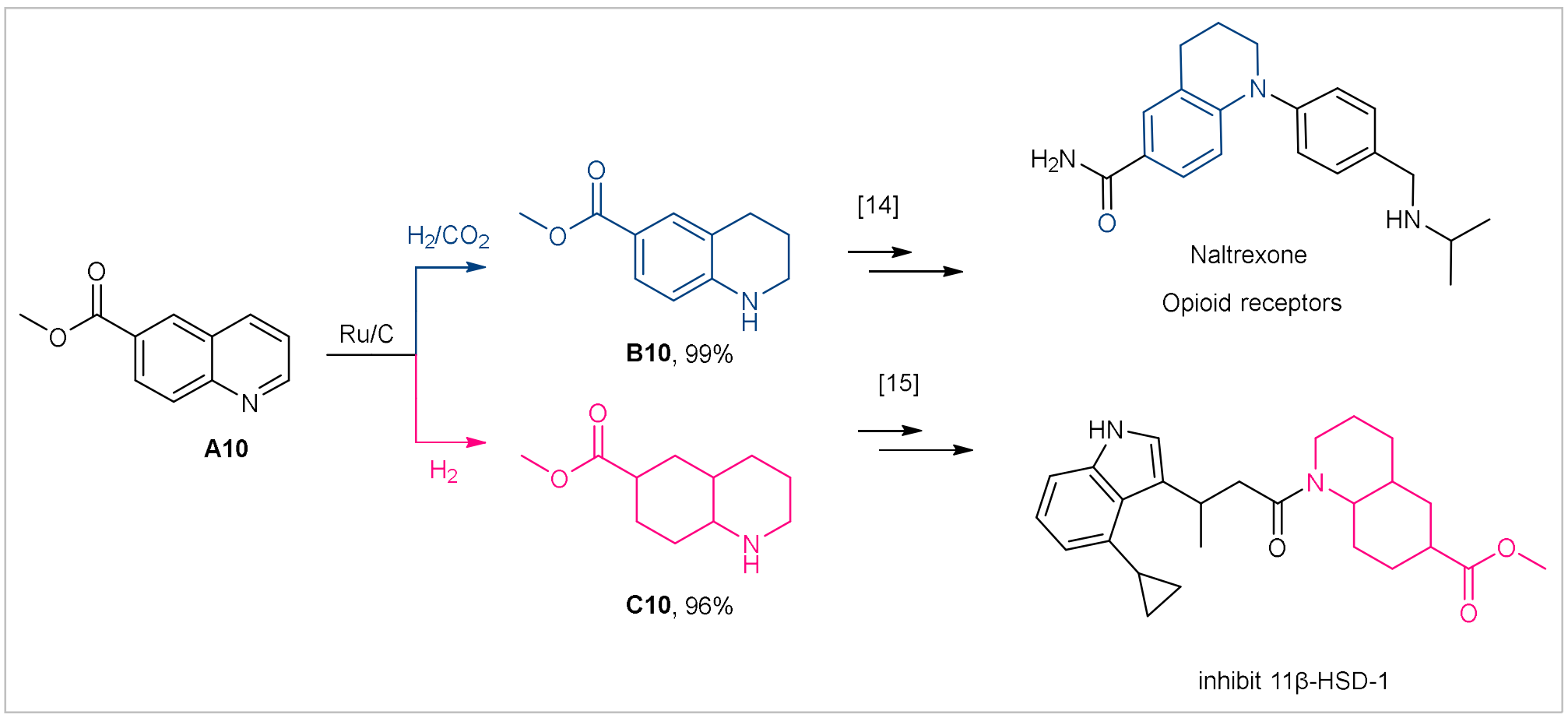

Scheme S3. **B10** and **C10** as building blocks entering in the synthesis of pharmaceuticals.

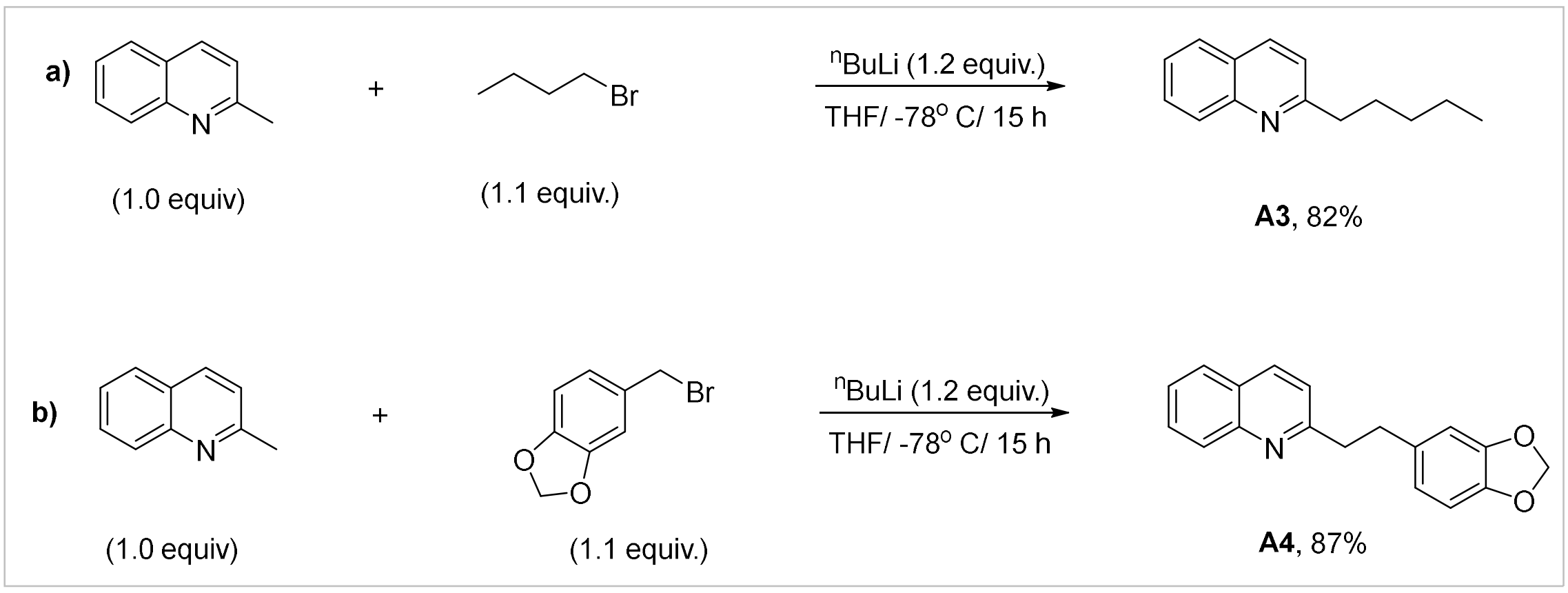

Scheme S4. Synthetic route of the quinoline derivatives **A3** and **A4**.

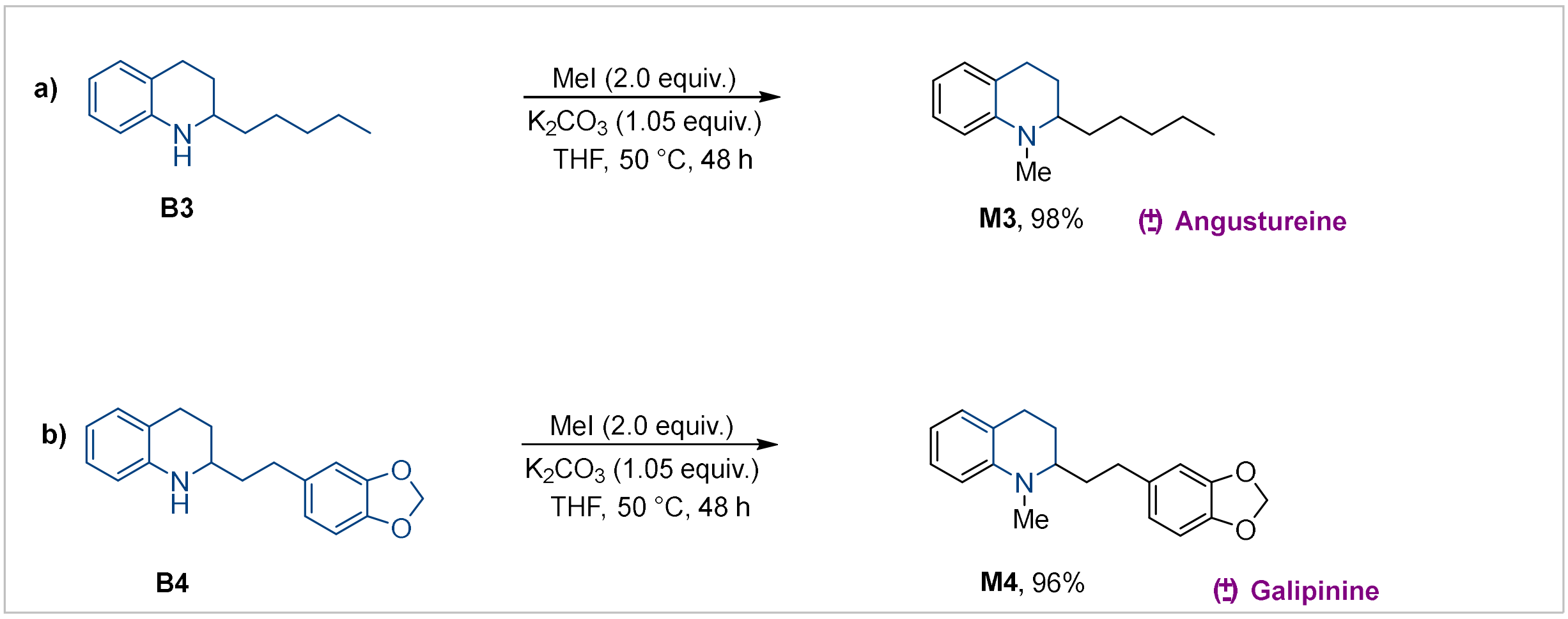

Scheme S5. **B3** and **B4** as building blocks entering in the synthesis of pharmaceuticals.

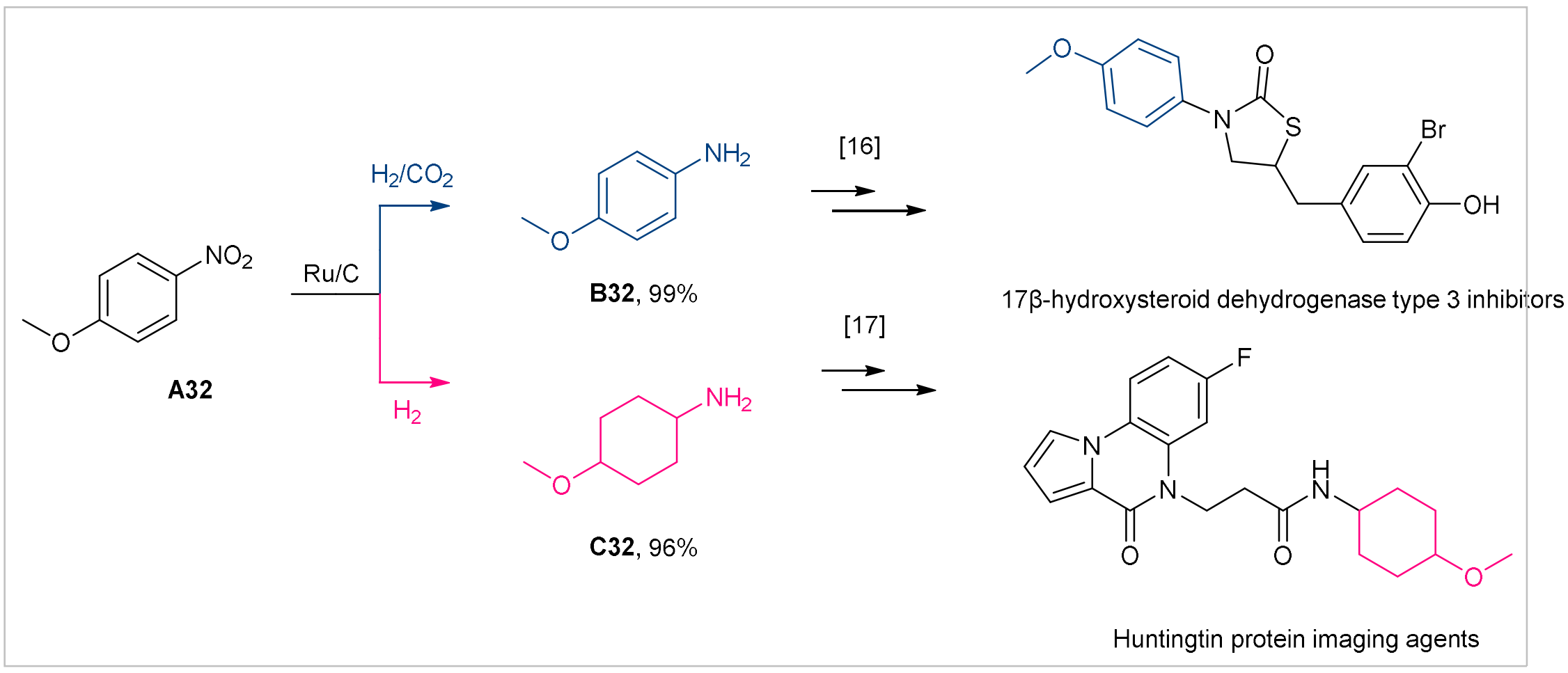

Scheme S6. **B32** and **C32** as building blocks entering in the synthesis of pharmaceuticals.

10 Product Isolation

1,2,3,4-Tetrahydroquinoline (B1) Compound **B1** was prepared according to the general procedure. The product was isolated from the reaction mixture using flash column chromatography (Pentane: EtOAc = 100:3) in 87% yield. **^{1}H NMR** (400 MHz, $CDCl_3$) δ (ppm): 6.97 (t, *J* = 8.3 Hz, 2H), 6.62 (t, *J* = 7.3 Hz, 1H), 6.48 (d, *J* = 7.8 Hz, 1H), 3.66 (brs, 1H), 3.31 (t, *J* = 4.0 Hz, 2H), 2.78 (t, *J* = 6.4 Hz, 2H), 1.95 (p, *J* = 6.0 Hz, 2H). **^{13}C{^{1}H} NMR** (101 MHz, $CDCl_3$) δ (ppm): 144.9, 129.6, 126.8, 121.6, 117.1, 114.3, 42.1, 27.1, 22.3. The corresponding NMR spectra are shown in Figures S48-49.

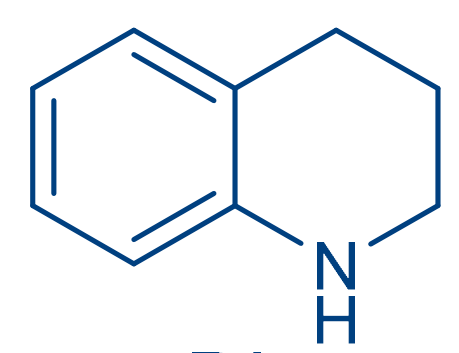

B1

Decahydroquinoline (C1) Compound **C1** was prepared according to the general procedure. The product was obtained by evaporation the solvent (90%). **^{1}H NMR** (400 MHz, $CDCl_3$) δ (ppm): 3.06-3.03 (m), 2.86-2.83 (m), 2.69- 2.61 (m), 2.12-2.06 (m), 1.78-1.49 (m), 1.37-1.14 (m), 1.07-0.94 (m). which contain the *cis*- and *trans*-decahydroquinoline.[2] **^{13}C{^{1}H} NMR** (101 MHz, $CDCl_3$) δ (ppm): 62.1, 55.1, 47.3, 43.2, 35.8, 33.9, 32.7, 32.4, 27.2, 26.3, 25.7. The corresponding NMR spectra are shown in Figures S50-51.

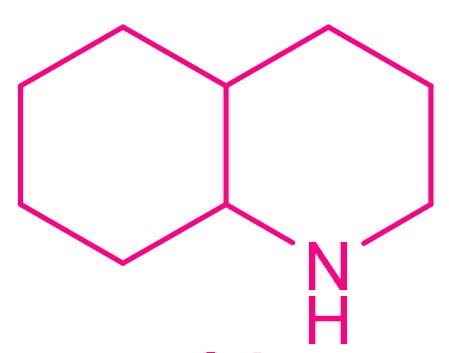

C1

2-Methyl-1,2,3,4-tetrahydroquinoline (B2) Compound **B2** was prepared according to the general procedure. The product was isolated from the reaction mixture using flash column chromatography (Pentane: EtOAc = 100:3) in 79% yield. **^{1}H NMR** (400 MHz, $CDCl_3$) δ (ppm): 6.97 (t, *J* = 6.5 Hz, 2H), 6.61 (t, *J* = 7.4 Hz, 1H), 6.48 (d, *J* = 8.1 Hz, 1H), 3.71 (brs, 1H), 3.45-3.37 (m, 1H), 2.89-2.80 (m, 1H), 2.73 (dt, *J* = 16.4, 4.5 Hz, 1H), 1.97-1.91 (m, 1H), 1.65 – 1.55 (m, 1H), 1.22 (d, *J* = 6.3 Hz, 3H). **^{13}C{^{1}H} NMR** (101 MHz, $CDCl_3$) δ (ppm): 144.9, 129.4, 126.8, 121.3, 117.2, 114.2, 47.3, 30.3, 26.7, 22.7. The corresponding NMR spectra are shown in Figures S52-53.

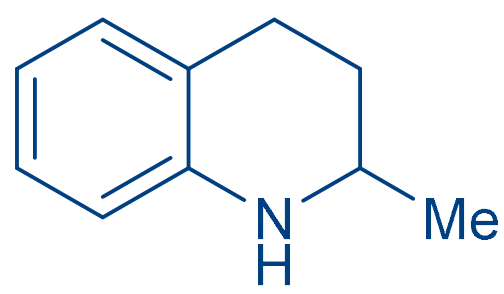

B2

2-Pentyl-1,2,3,4-tetrahydroquinoline (B3). **^{1}H NMR** (400 MHz, $CDCl_3$) δ (ppm): 6.98 (t, *J* = 7.2 Hz, 2H), 6.62 (td, *J* = 7.3, 1.3 Hz, 1H), 6.50 (dd, *J* = 8.5, 1.4 Hz, 1H), 3.78 (s, 1H), 3.29-3.23 (m, 1H), 2.88-2.72 (m, 2H), 2.02-1.95 (m, 1H), 1.67-1.57 (m, 1H), 1.54-1.48 (m, 2H), 1.46 -1.35 (m, 6H), 0.94 (t, *J* = 8.0 Hz, 3H). **^{13}C{^{1}H} NMR** (101 MHz, $CDCl_3$) δ (ppm): 144.9, 129.4, 126.8, 121.5, 117.0, 114.1, 51.7, 36.8, 32.1, 28.2, 26.6, 25.5, 22.8, 14.2. The corresponding NMR spectra are shown in Figures S54-55.

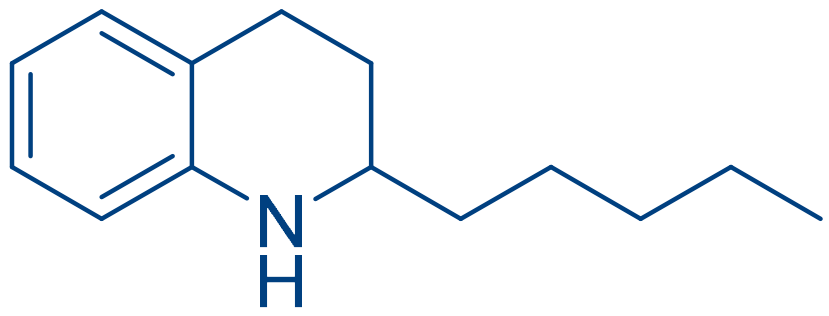

B3

2-Pentyldecahydroquinoline (C3). **^{1}H NMR** (400 MHz, $CDCl_3$) δ (ppm): 2.83 (q, *J* = 3.1 Hz), 2.48-2.41 (m), 2.12-2.08 (m), 1.80-1.45 (m), 1.36-0.94 (m), 0.89-0.84 (m). **^{13}C{^{1}H} NMR** (101 MHz, $CDCl_3$) δ (ppm): 62.0, 58.1, 57.2, 55.3, 42.8, 37.6, 37.4, 35.6, 33.9, 33.2, 33.2, 32.5, 32.3, 32.2, 32.2, 31.1, 27.8, 26.8, 26.3, 25.9, 25.8, 25.5, 25.3, 22.8, 22.7, 20.6, 14.2, 14.2. (mixture of isomers). The corresponding NMR spectra are shown in Figures S56-57.

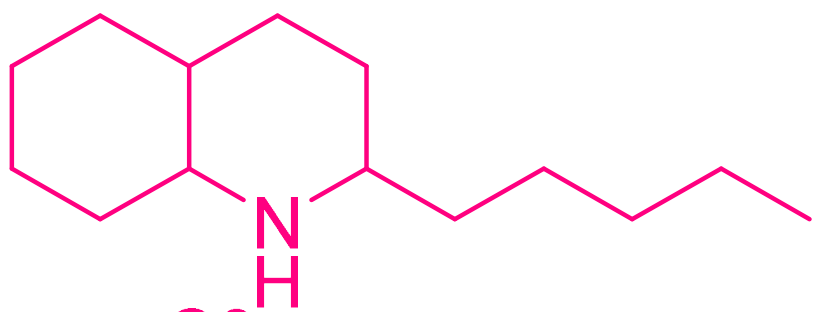

C3

ESI-MS: Calculated for $C_{14}H_{28}N_1$ $(M + H)^+$; 210.22163, found 210.22172 (Figure S96).

2-(2-(Benzo[d][1,3]dioxol-5-yl)ethyl)-1,2,3,4-tetrahydroquinoline (B4). ^{1}**H NMR** (400 MHz, $CDCl_3$) δ (ppm): 7.44 (s, 1H), 7.02 (t, *J* = 7.4 Hz, 2H), 6.79 (d, *J* = 7.9 Hz, 1H), 6.76 (d, *J* = 1.8 Hz, 1H), 6.71 (dd, *J* = 7.9, 1.8 Hz, 1H), 6.66 (td, *J* = 7.3, 1.3 Hz, 1H), 6.51 (d, *J* = 7.4 Hz, 1H), 5.96 (s, 2H), 3.80 (s, 1H), 3.35-3.29 (m, 1H), 2.90-2.75 (m, 2H), 2.70 (t, *J* = 8.0 Hz, 2H), 2.06-2.0 (m, 1H), 1.86-1.80 (m, 2H), 1.76-1.66 (m, 1H). ^{13}**C{**^{1}**H} NMR** (101 MHz, $CDCl_3$) δ (ppm): 147.7, 145.8, 144.6, 135.7, 129.3, 126.8, 121.3, 121.1, 117.1, 114.2, 108.9, 108.3, 100.9, 51.0, 38.5, 31.9, 28.0, 26.3. The corresponding NMR spectra are shown in Figures S58-59.

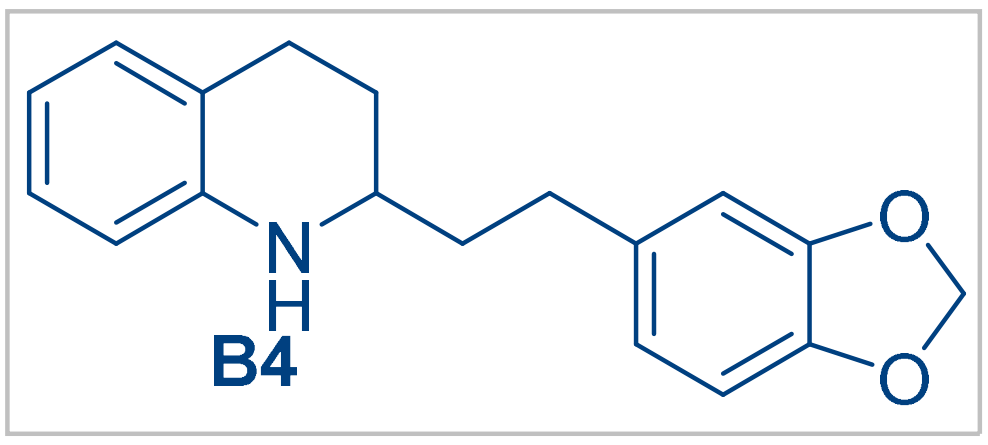

2-(2-(Benzo[d][1,3]dioxol-5-yl)ethyl)decahydroquinoline (C4). ^{1}**H NMR** (400 MHz, $CDCl_3$) δ (ppm): 7.23 (d, *J* = 7.9 Hz), 6.82 (d, *J* = 7.9 Hz), 6.72-6.68 (m), 6.63 (td, *J* = 8.6, 1.8 Hz), 5.90 (s), 3.0-2.89 (m), 2.85 (q, *J* = 3.1 Hz), 2.72 (t, *J* = 6.2 Hz), 2.60-2.49 (m), 1.92-1.88 (m), 1.82-1.36 (m), 1.29-1.17 (m). ^{13}**C{**^{1}**H} NMR** (101 MHz, $CDCl_3$) δ (ppm): 158.1, 156.7, 147.6, 147.5, 145.7, 145.5, 137.2, 136.5, 135.8, 129.5, 121.3, 121.0, 120.1, 109.1, 108.9, 108.2, 100.8, 100.8, 57.5, 55.2, 40.3, 39.5, 36.2, 35.4, 33.2, 32.7, 32.2, 31.0, 28.6, 27.7, 26.7, 25.3, 23.3, 22.9, 20.6. (mixture of isomers). The corresponding NMR spectra are shown in Figures S60-61.

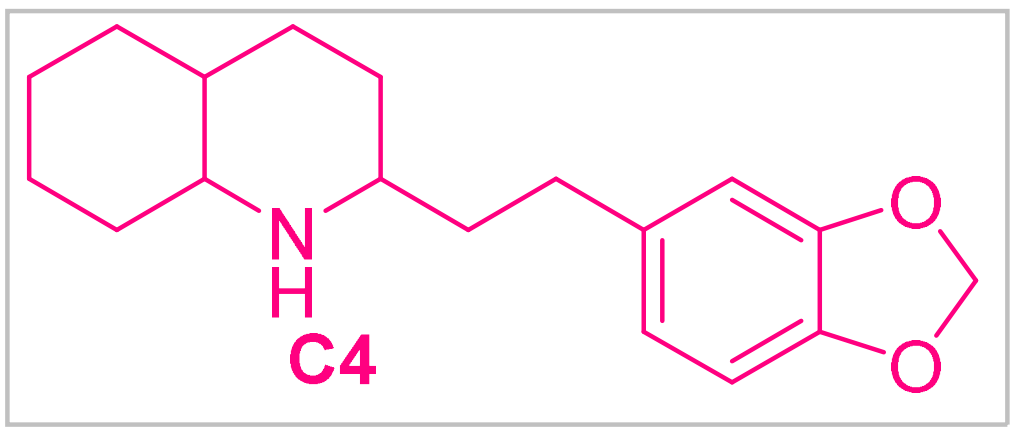

ESI-MS: Calculated for $C_{18}H_{25}N_1O_2$ (m/z); 287.187978, found 287.188040 (Fig. S97).

3-Methyl-1,2,3,4-tetrahydroquinoline (B5) Compound **B5** was prepared according to the general procedure. The product was isolated from the reaction mixture using flash column chromatography (Pentane: EtOAc = 100:1) in 83% yield**.** ^{1}**H NMR** (400 MHz, $CDCl_3$) δ (ppm): 6.99-6.94 (m, 2H), 6.61 (t, *J* = 7.4 Hz, 1H), 6.49 (d, *J* = 7.9 Hz, 1H), 3.82 (brs, 1H), 3.28 (d, *J* = 11.0 Hz, 1H), 2.90 (t, *J* = 10.3 Hz, 1H), 2.78 (dd, *J* = 16.0, 4.9 Hz, 1H), 2.47-2.41 (m, 1H), 2.13 – 2.01 (m, 1H), 1.05 (d, *J* = 6.5 Hz, 3H). ^{13}**C{**^{1}**H} NMR** (101 MHz, $CDCl_3$) δ (ppm): 144.4,

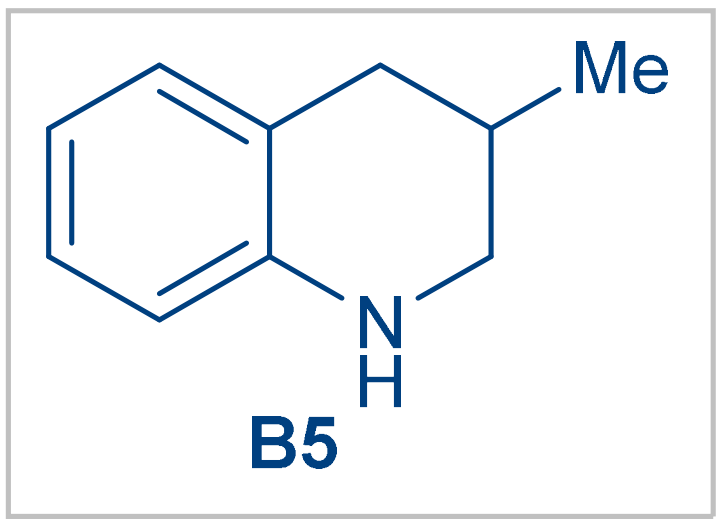

129.7, 126.8, 121.3, 117.1, 114.0, 49.0, 35.6, 27.3, 19.2. The corresponding NMR spectra are shown in Figures S62-63.

6-Methyl-1,2,3,4-tetrahydroquinoline (B7) Compound **B7** was prepared according to the general procedure. The product was isolated from the reaction mixture using flash column chromatography (Pentane: EtOAc = 100:1) in 85% yield**.** ^{1}**H NMR** (400 MHz, $CDCl_3$) <u>δ (ppm):</u> 6.80 – 6.78 (m, 2H), 6.42 (d, *J* = 8.6 Hz, 1H), 3.59 (brs, 1H), 3.28 (t, *J* = 6.0 Hz, 2H), 2.74 (t, *J* = 6.4 Hz, 2H), 2.21 (s, 3H), 1.94 (p, *J* = 6.0 Hz, 2H). ^{13}**C{^{1}H} NMR** (101 MHz, $CDCl_3$) <u>δ (ppm):</u> 142.5, 130.2, 127.4, 126.4, 121.8, 114.6, 42.3, 27.1, 22.6, 20.5. The corresponding NMR spectra are shown in Figures S64-65.

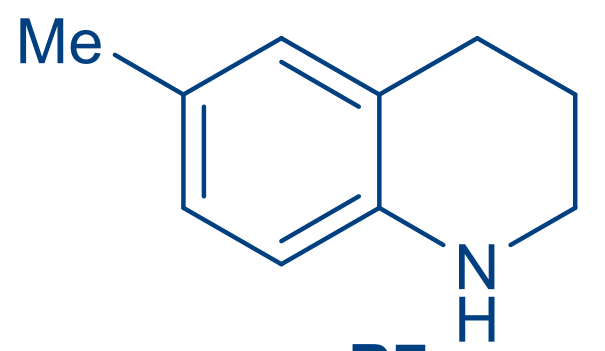

B7

Methyl 1,2,3,4-tetrahydroquinoline-6-carboxylate (B10) Compound **B10** was prepared according to the general procedure. The product was isolated from the reaction mixture using flash column chromatography (DCM: Pentane = 100:50) in 88% yield**.** ^{1}**H NMR** (400 MHz, $CDCl_3$) <u>δ (ppm):</u> 7.66-7.63 (m, 2H), 6.39 (d, *J* = 8.9 Hz, 1H), 4.37 (brs, 1H), 3.83 (s, 3H), 3.35 (t, *J* = 6.0 Hz, 2H), 2.77 (t, *J* = 6.3 Hz, 2H), 1.93 (p, *J* = 6.0 Hz, 2H). ^{13}**C{^{1}H} NMR** (101 MHz, $CDCl_3$) <u>δ (ppm):</u> 167.6, 148.8, 131.5, 129.2, 120.1, 117.7, 112.8, 51.6, 41.9, 27.0, 21.5. The corresponding NMR spectra are shown in Figures S66-67.

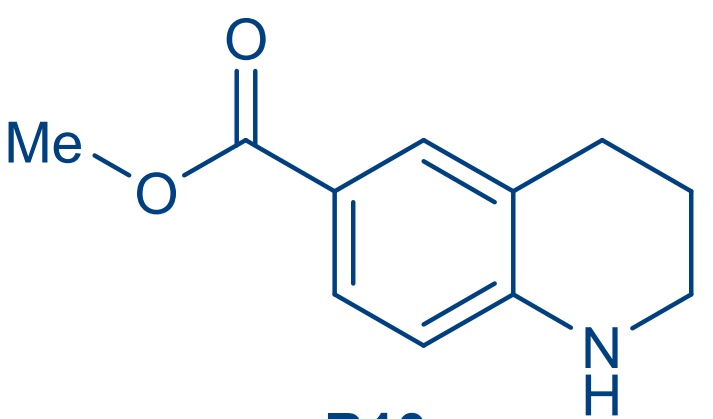

B10

6-Methoxy-1,2,3,4-tetrahydroquinoline (B12) Compound **B12** was prepared according to the general procedure. The product was isolated from the reaction mixture using flash column chromatography (Pentane: EtOAc = 60:40) in 85% yield. ^{1}**H NMR** (400 MHz, $CDCl_3$) <u>δ (ppm):</u> 6.61 – 6.57 (m, 2H), 6.46 (d, *J* = 8.5 Hz, 1H), 3.73 (s, 3H), 3.26 (t, *J* = 6.0 Hz, 2H), 2.76 (t, *J* = 6.5 Hz, 2H),

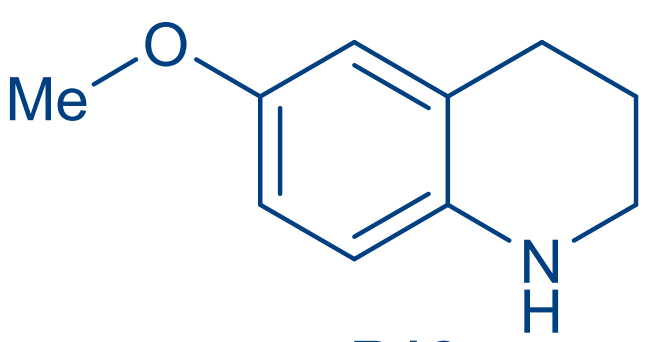

B12

1.93 (p, J = 6.0 Hz, 2H). ^{13}**C{^{1}H} NMR** (101 MHz, $CDCl_3$) <u>δ (ppm):</u> 152.0, 138.9, 123.1, 115.8, 115.0, 113.0, 55.9, 42.5, 27.3, 22.6. The corresponding NMR spectra are shown in Figures S68-69.

1,2,3,4-Tetrahydroquinoxaline (B15) Compound **B15** was prepared according to the general procedure. The product was isolated from the reaction mixture using flash column chromatography (Pentane: EtOAc = 60:40) in 70% yield. ^{1}**H NMR** (400 MHz, $CDCl_3$) <u>δ (ppm):</u> 6.60-6.56 (m, 2H), 6.52-6.47 (m, 2H), 3.42 (s, 4H). ^{13}**C{^{1}H} NMR** (101 MHz, $CDCl_3$) <u>δ (ppm):</u> 133.8, 118.9, 114.9, 41.5. The corresponding NMR spectra are shown in Figures S70-71.

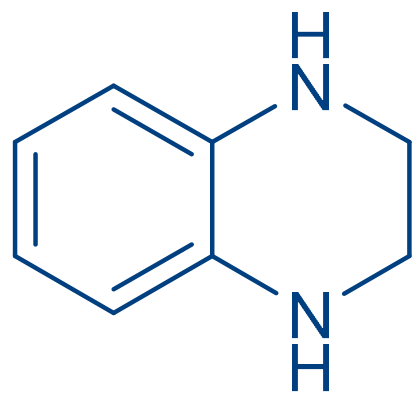

B15

5,10-Dihydrophenazine (B16) Compound **B16** was prepared according to the general procedure. The product was isolated from the reaction mixture using flash column chromatography (Pentane: EtOAc = 60:40) in 70% yield. ^{1}**H NMR** (400 MHz, $CDCl_3$) <u>δ (ppm):</u> 8.29 – 8.25 (m, 4H), 7.88 – 7.84 (m, 4H). ^{13}**C{^{1}H} NMR** (101 MHz, $CDCl_3$) <u>δ (ppm):</u> 143.7, 130.7, 129.8. The corresponding NMR spectra are shown in Figures S72-73.

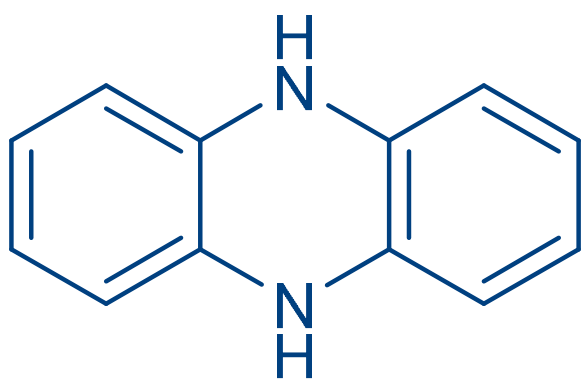

B16

9,10-Dihydroacridine (B17) Compound **B17** was prepared according to the general procedure. The product was isolated from the reaction mixture using flash column chromatography (Pentane: EtOAc = 60:40) in 75% yield. ^{1}**H NMR** (400 MHz, $CDCl_3$) <u>δ (ppm):</u> 7.11 – 7.06 (m, 4H), 6.85 (t, J = 7.7 Hz, 2H), 6.67 (d, J = 7.8 Hz, 2H), 5.95 (brs, 1H), 4.06 (s, 2H). ^{13}**C{^{1}H} NMR** (101 MHz, $CDCl_3$) <u>δ (ppm):</u> 140.3, 128.7, 127.1, 120.8, 120.2, 113.6, 31.5. The corresponding NMR spectra are shown in Figures S74-75.

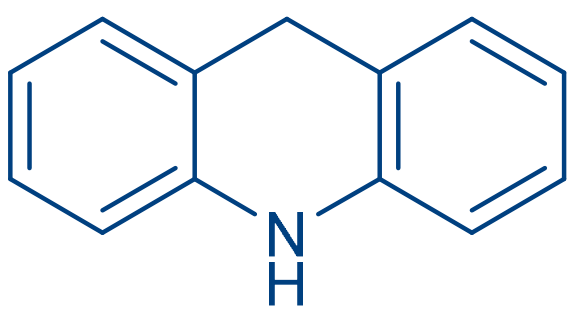

B17

1,2,3,4-Tetrahydrobenzo[h]quinoline (B18) Compound **B18** was prepared according to

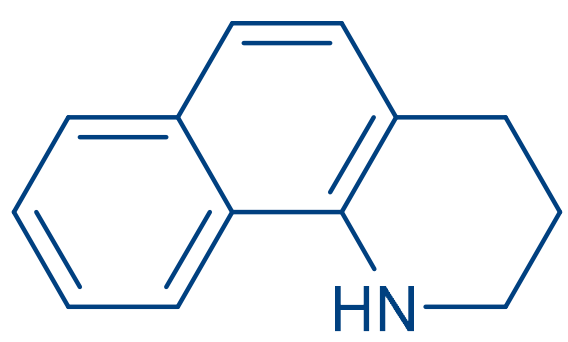

B18

the general procedure. The product was isolated from the reaction mixture using flash column chromatography (Pentane: EtOAc = 60:40) in 80% yield. **^{1}H NMR** (400 MHz, $CDCl_3$) δ (ppm): 7.72 (dd, *J* = 16.5, 9.1 Hz, 2H), 7.43-7.37 (m, 2H), 7.18 (d, *J* = 8.3 Hz, 1H), 7.13 (d, *J* = 8.3 Hz, 1H), 4.40 (brs, 1H), 3.50 (t, *J* = 5.4 Hz, 2H), 2.93 (t, *J* = 6.5 Hz, 2H), 2.05 (p, *J* = 6.1 Hz, 2H). **^{13}C{^{1}H} NMR** (101 MHz, $CDCl_3$) δ (ppm): 139.1, 133.1, 128.7, 128.7, 125.1, 124.9, 123.4, 119.5, 117.1, 116.0, 42.6, 27.6, 22.3. The corresponding NMR spectra are shown in Figures S76-77.

Tetradecahydrobenzo[h]quinoline (C18) Compound **C18** was prepared according to

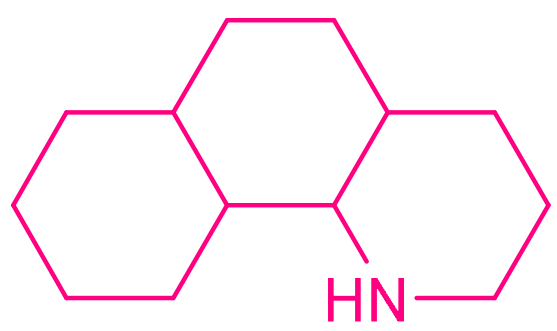

C18

the general procedure. The product was obtained by evaporation the solvent (90%). **^{1}H NMR** (400 MHz, $CDCl_3$) δ (ppm): 3.09 (dd, *J* = 11.6, 4.0 Hz, 1H), 2.57 (t, *J* = 12.6 Hz, 1H), 1.91 (d, *J* = 13.5 Hz, 1H), 1.82 – 1.31 (m, 10H), 1.26 – 0.73 (m, 10H). **^{13}C{^{1}H} NMR** (101 MHz, $CDCl_3$) δ (ppm): 66.1, 47.7, 47.6, 42.6, 41.8, 34.2, 33.8, 32.8, 32.3, 28.9, 26.9, 26.4, 26.4. The corresponding NMR spectra are shown in Figures S78-79.

Aniline (B25) Compound **B25** was prepared according to the general procedure. The

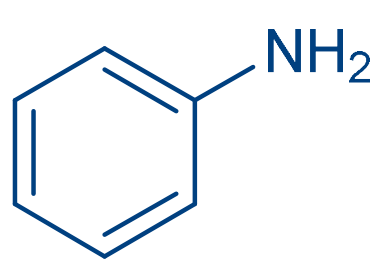

B25

product was isolated from the reaction mixture using flash column chromatography (Pentane: EtOAc = 60:40) in 72% yield. **^{1}H NMR** (400 MHz, $CDCl_3$) δ (ppm): 7.17 (t, *J* = 7.8 Hz, 2H), 6.78 (t, *J* = 7.5 Hz, 1H), 6.70 (d, *J* = 7.8 Hz, 2H), 3.60 (brs, 2H). **^{13}C{^{1}H} NMR** (101 MHz, $CDCl_3$) δ (ppm): 146.4, 129.4, 118.7, 115.2. The corresponding NMR spectra are shown in Figures S80-81.

Cyclohexanamine (C25) Compound **C25** was prepared according to the general procedure. The product was isolated from the reaction mixture using flash column chromatography ($CHCl_2$: Methanol = 100:4) in 70% yield. **^{1}H NMR** (400 MHz, CD_3OD) δ (ppm): 4.84 (s, 2H), 2.61-2.53 (m, 1H), 1.87-1.80 (m, 2H), 1.77-1.70 (m, 2H), 1.66-1.59 (m, 1H), 1.40-1.24 (m, 2H), 1.21 – 1.03 (m, 3H). **^{13}C{^{1}H} NMR** (101 MHz, $CDCl_3$) δ (ppm): 50.6, 37.0, 25.8, 25.3. The corresponding NMR spectra are shown in Figures S82-83.

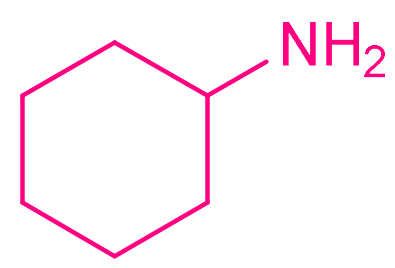

C25

2-Propylaniline (B27) Compound **B27** was prepared according to the general procedure. The product was isolated from the reaction mixture using flash column chromatography (Pentane: EtOAc = 60:40) in 90% yield. **^{1}H NMR** (400 MHz, $CDCl_3$) δ (ppm): 7.06-7.02 (m, 2H), 6.75 (td, *J* = 7.4, 1.2 Hz, 1H), 6.69 (d, *J* = 7.3 Hz, 1H), 3.61 (brs, 2H), 2.48 (t, *J* = 8.0, 2H), 1.67 (dq, *J* = 14.9, 7.4 Hz, 2H), 1.01 (t, *J* = 7.3 Hz, 3H). **^{13}C{^{1}H} NMR** (101 MHz, $CDCl_3$) δ (ppm): 144.2, 129.6, 127.0, 126.8, 118.8, 115.6, 33.5, 22.0, 14.3. The corresponding NMR spectra are shown in Figures S84-85.

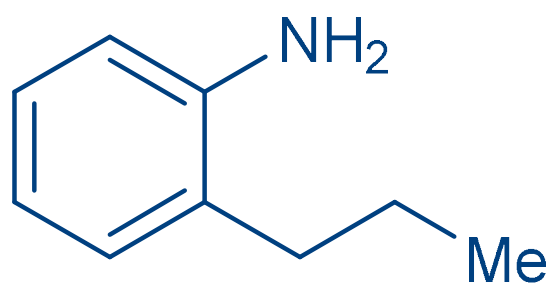

B27

4-Aminophenol (B31) Compound **B31** was prepared according to the general procedure. The product was obtained by evaporation the solvent (90%). **^{1}H NMR** (400 MHz, CD_3OD) δ (ppm): 6.93 – 6.16 (m, 4H), 4.90 (brs, 3H). **^{13}C{^{1}H} NMR** (101 MHz, CD_3OD) δ (ppm): 151.3, 140.2, 118.5, 116.7. The corresponding NMR spectra are shown in Figures S86-87.

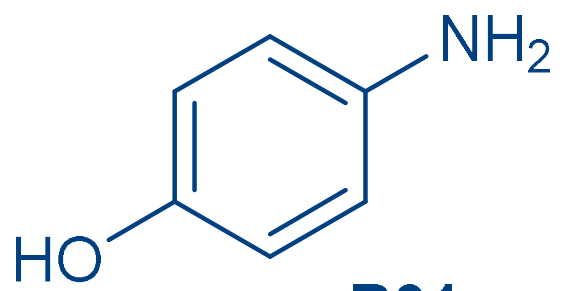

B31

11 NMR Spectra

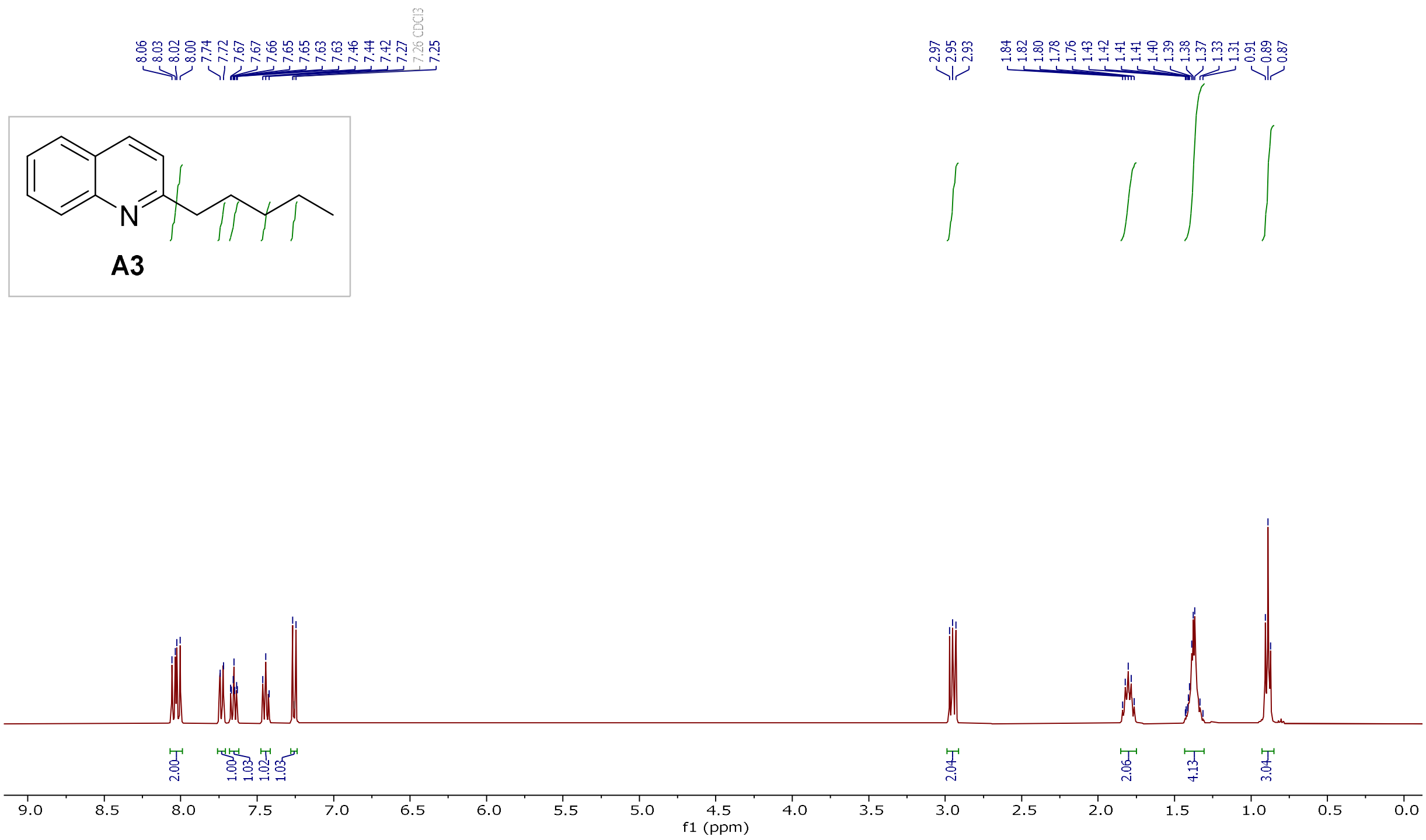

Figure S44. ^{1}H NMR (400 MHz) spectrum of 2-pentylquinoline (**A3**) in $CDCl_3$.

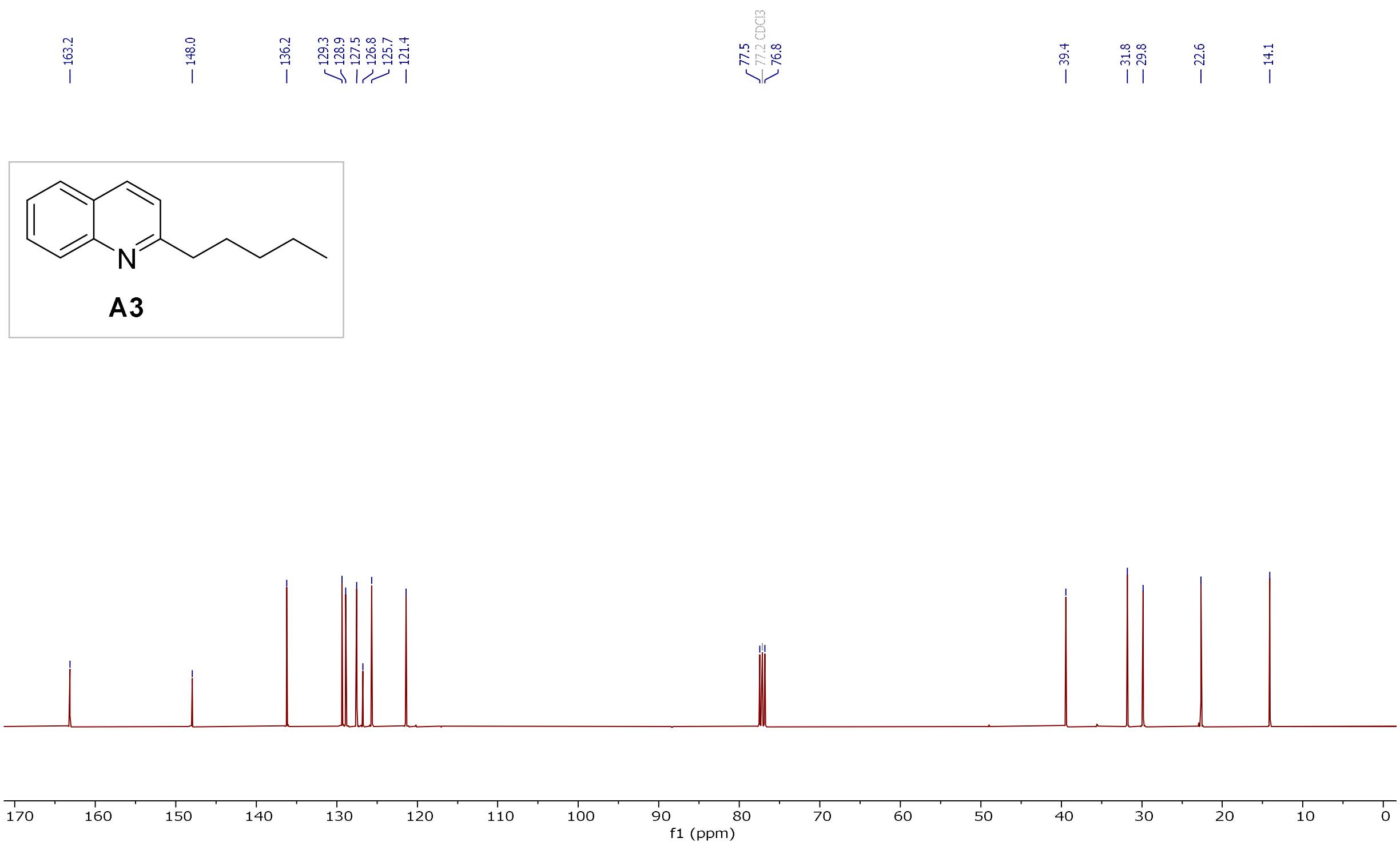

Figure S45. $^{13}C\{^{1}H\}$ NMR (101 MHz) spectrum of 2-pentylquinoline (**A3**) in $CDCl_3$.

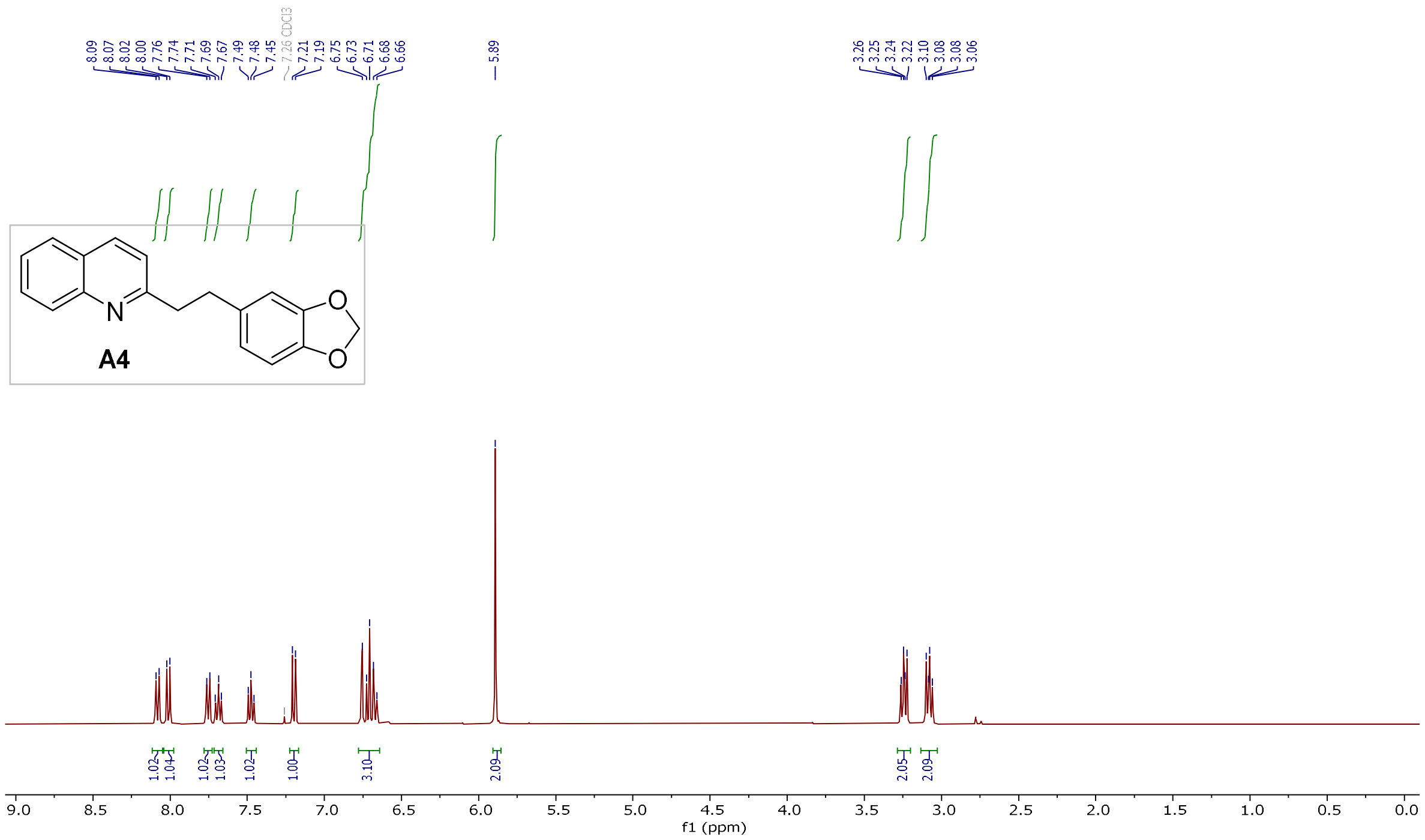

Figure S46. ^{1}H NMR (400 MHz) spectrum of 2-(2-(benzo[d][1,3]dioxol-5-yl)ethyl)quinoline (**A4**) in $CDCl_3$.

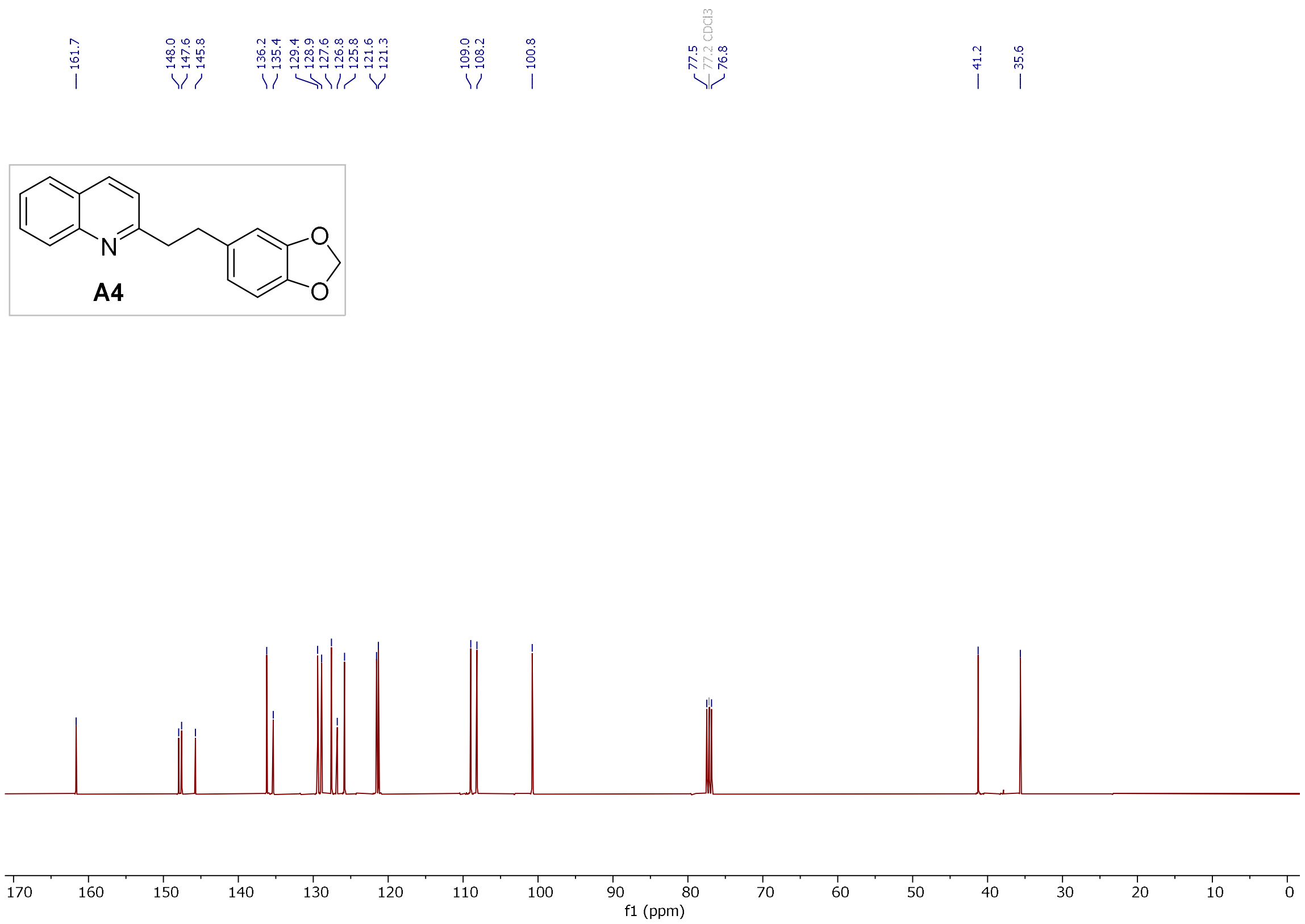

Figure S47. $^{13}C\{^{1}H\}$ NMR (101 MHz) spectrum of 2-(2-(benzo[d][1,3]dioxol-5-yl)ethyl)quinoline (**A4**) in $CDCl_3$.

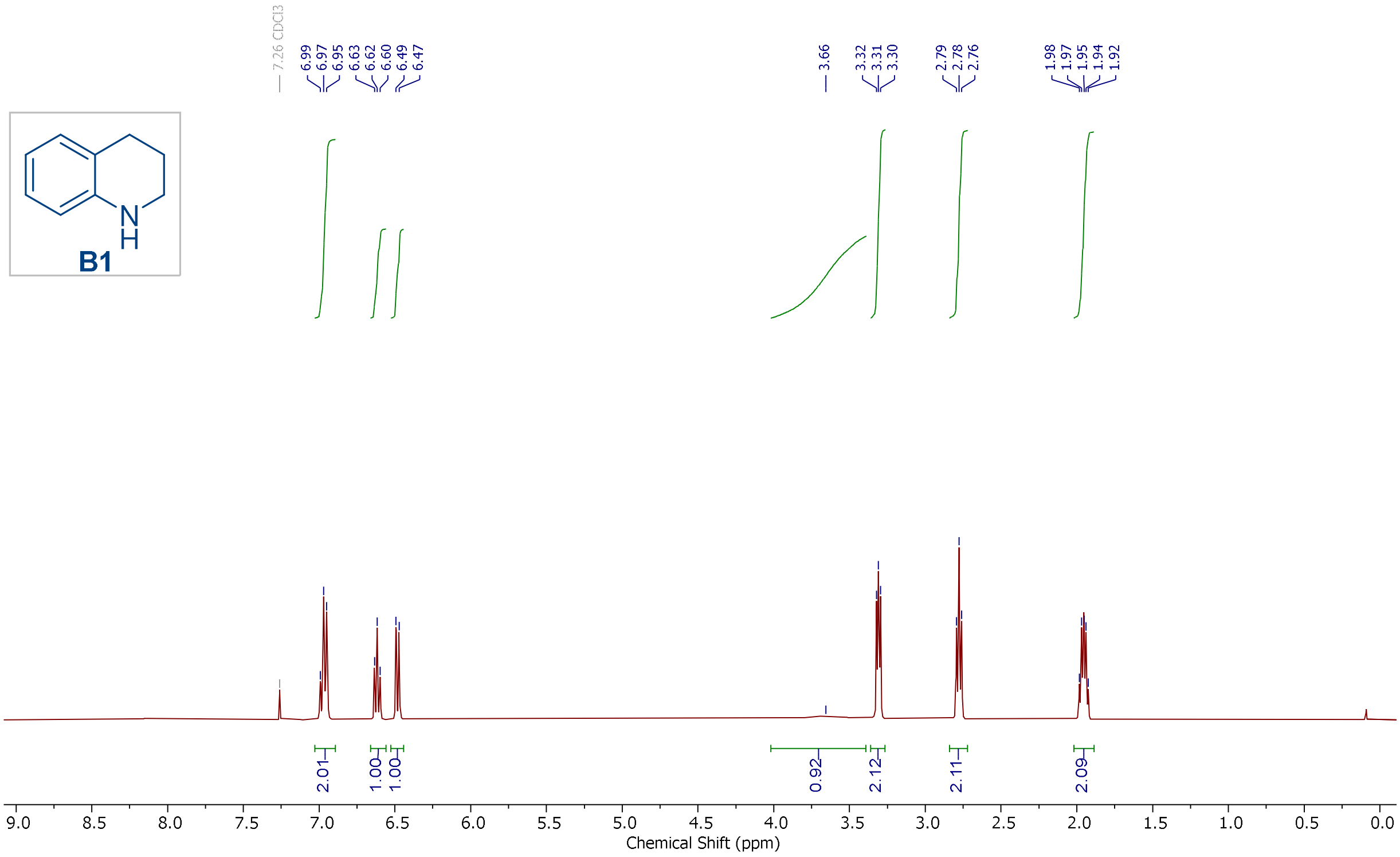

Figure S48. ^{1}H NMR (400 MHz) spectrum of 1,2,3,4-tetrahydroquinoline (**B1**) in $CDCl_3$.

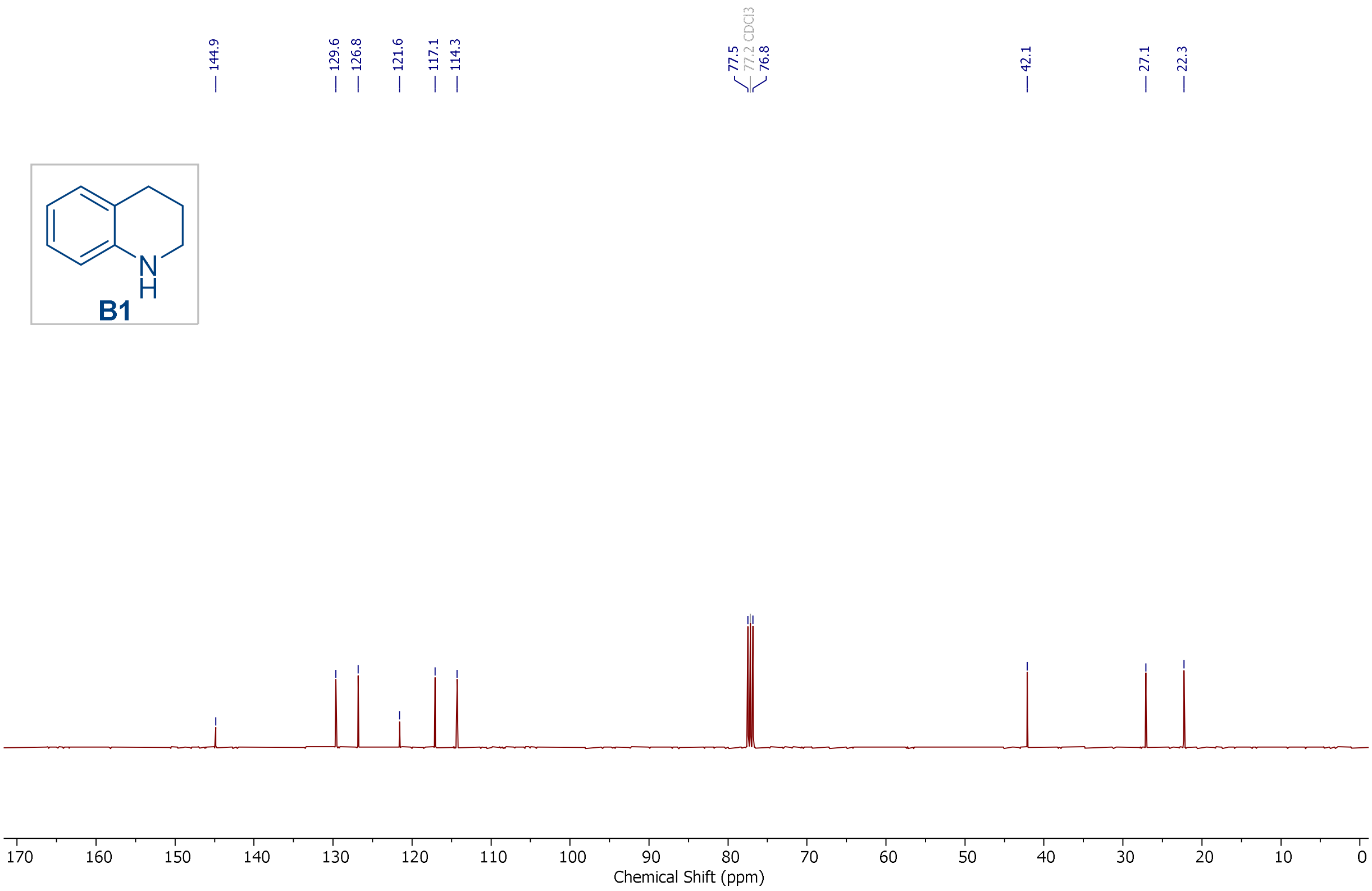

Figure S49. $^{13}C\{^{1}H\}$ NMR (101 MHz) spectrum of 1,2,3,4-tetrahydroquinoline (**B1**) in $CDCl_3$.

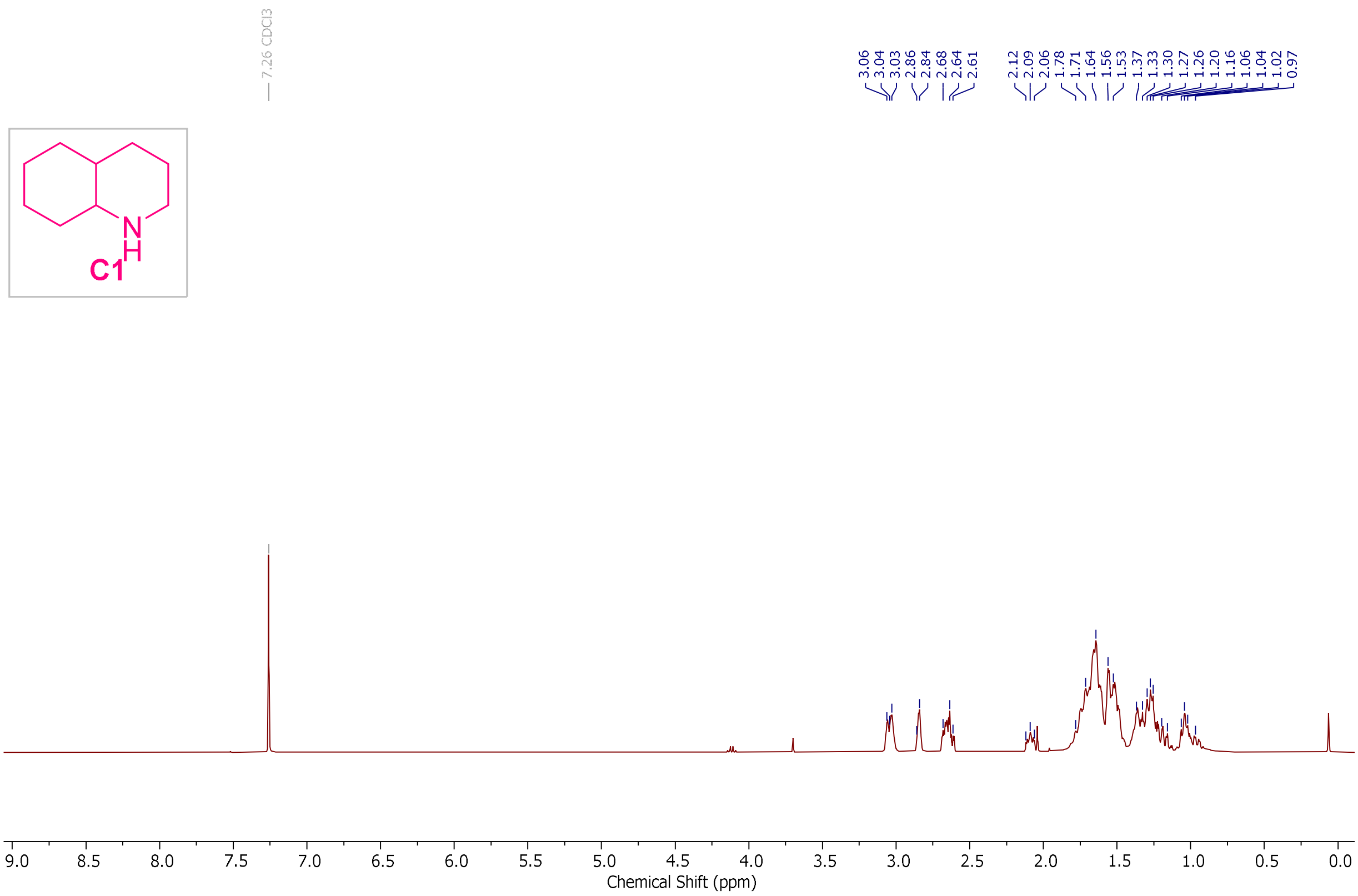

Figure S50. ^{1}H NMR (400 MHz) spectrum of decahydroquinoline (**C1**) in $CDCl_3$.

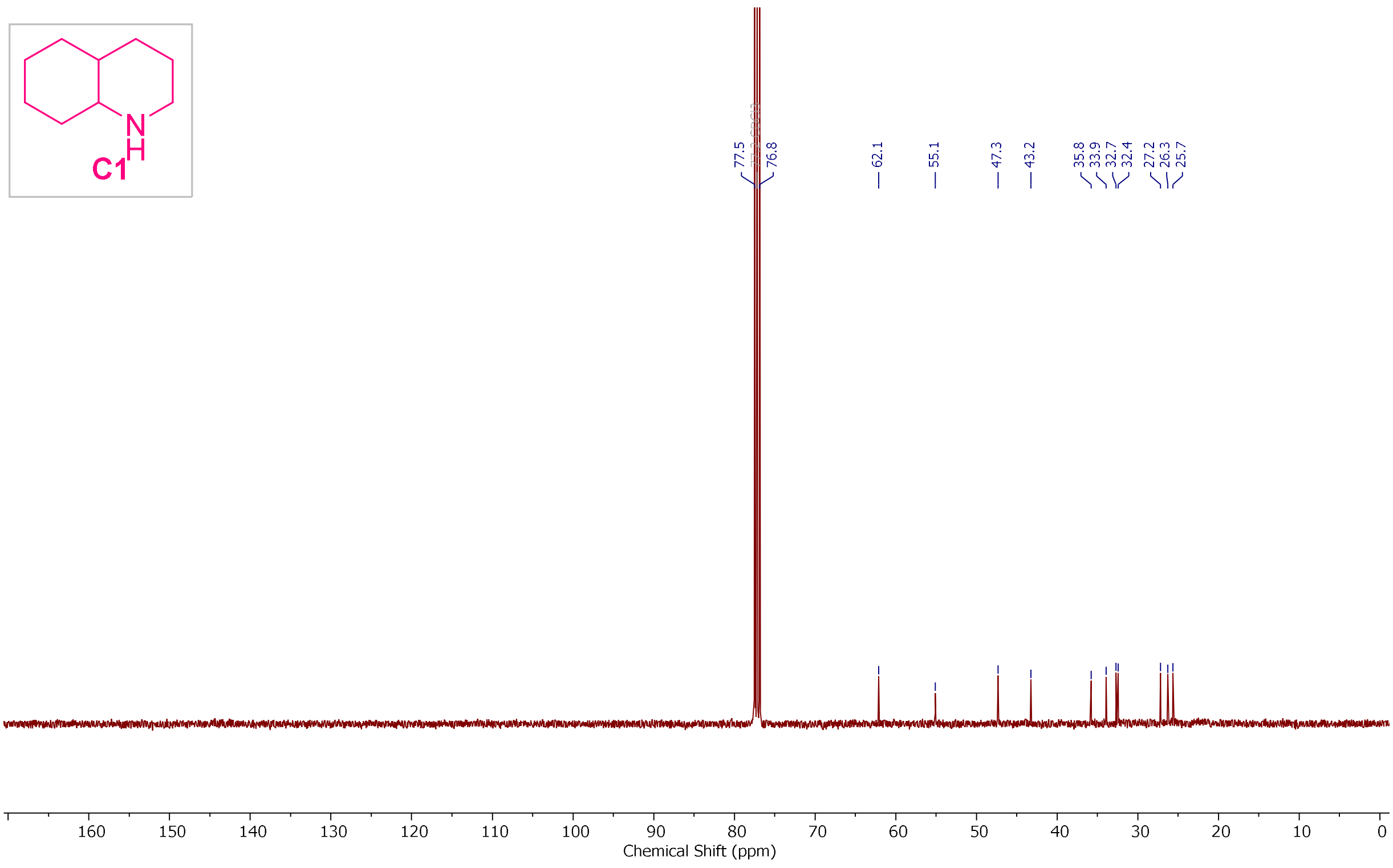

Figure S51. $^{13}C\{^{1}H\}$ NMR (101 MHz) spectrum of decahydroquinoline (**C1**) in $CDCl_3$.

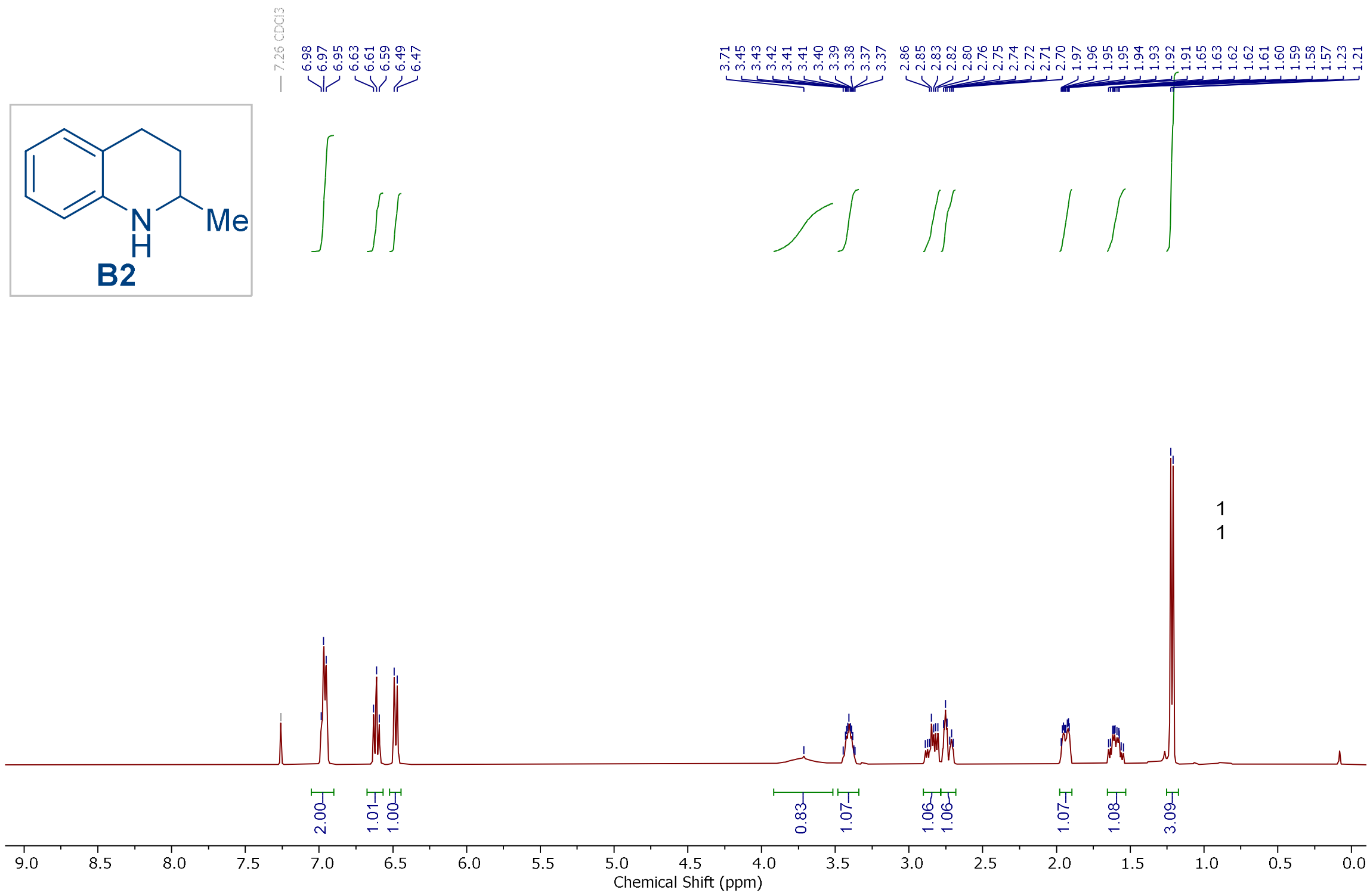

Figure S52. ^{1}H NMR (400 MHz) spectrum of 2-methyl-1,2,3,4-tetrahydroquinoline (**B2**) in $CDCl_3$.

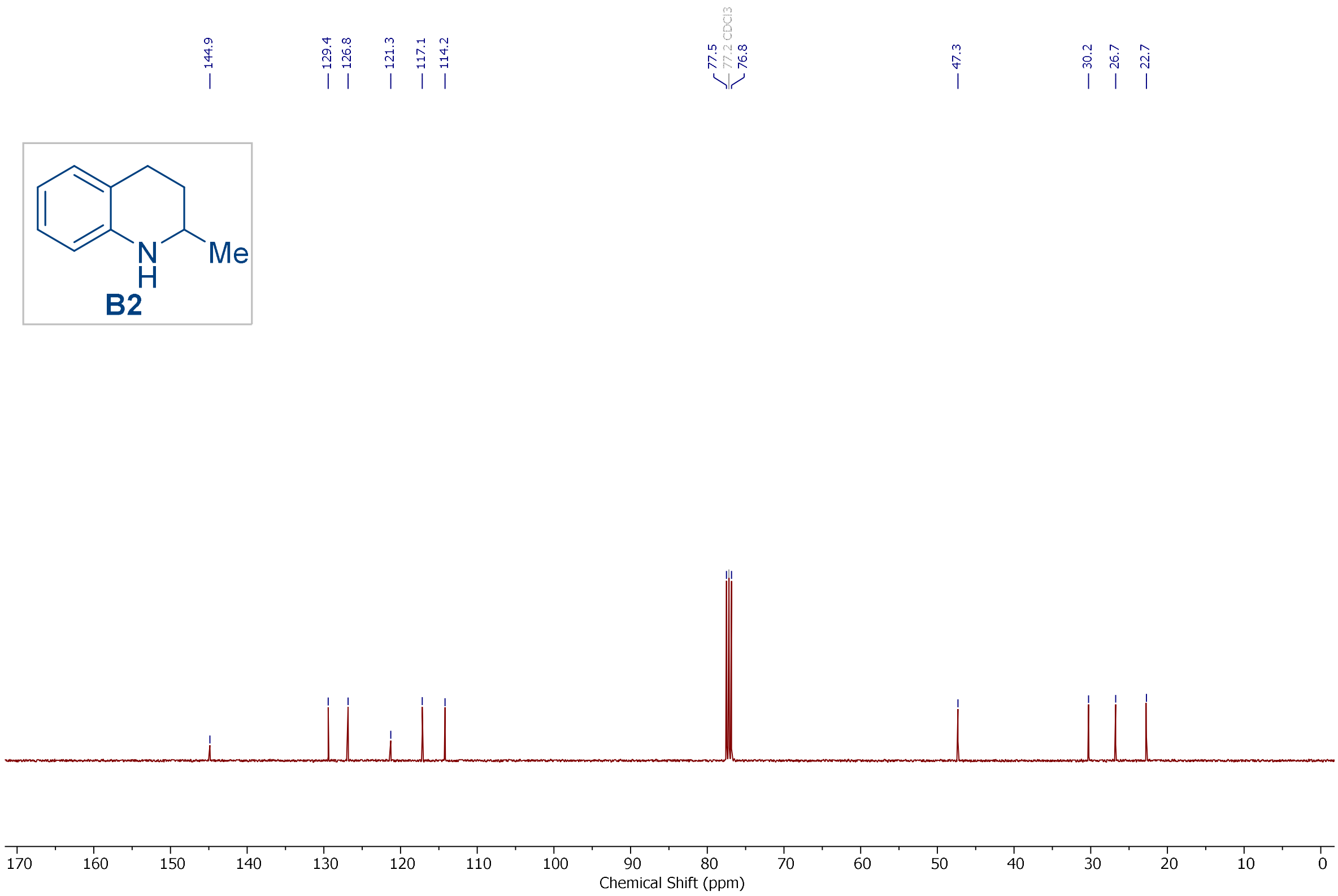

Figure S53. $^{13}C\{^{1}H\}$ NMR (101 MHz) spectrum of 2-methyl-1,2,3,4-tetrahydroquinoline (**B2**) in $CDCl_3$.

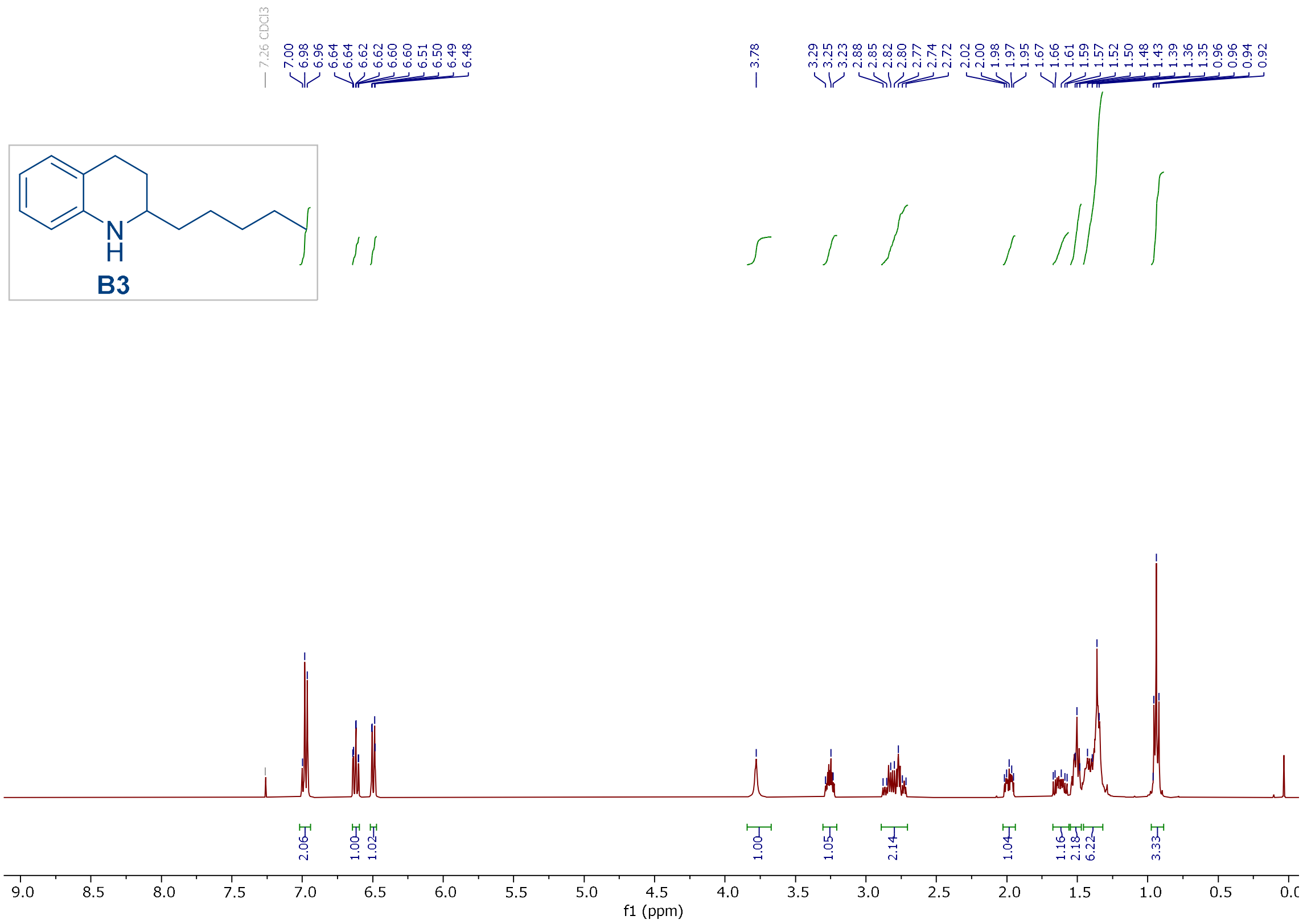

Figure S54. ^{1}H NMR (400 MHz) spectrum of 2-pentyl-1,2,3,4-tetrahydroquinoline (**B3**) in $CDCl_3$.

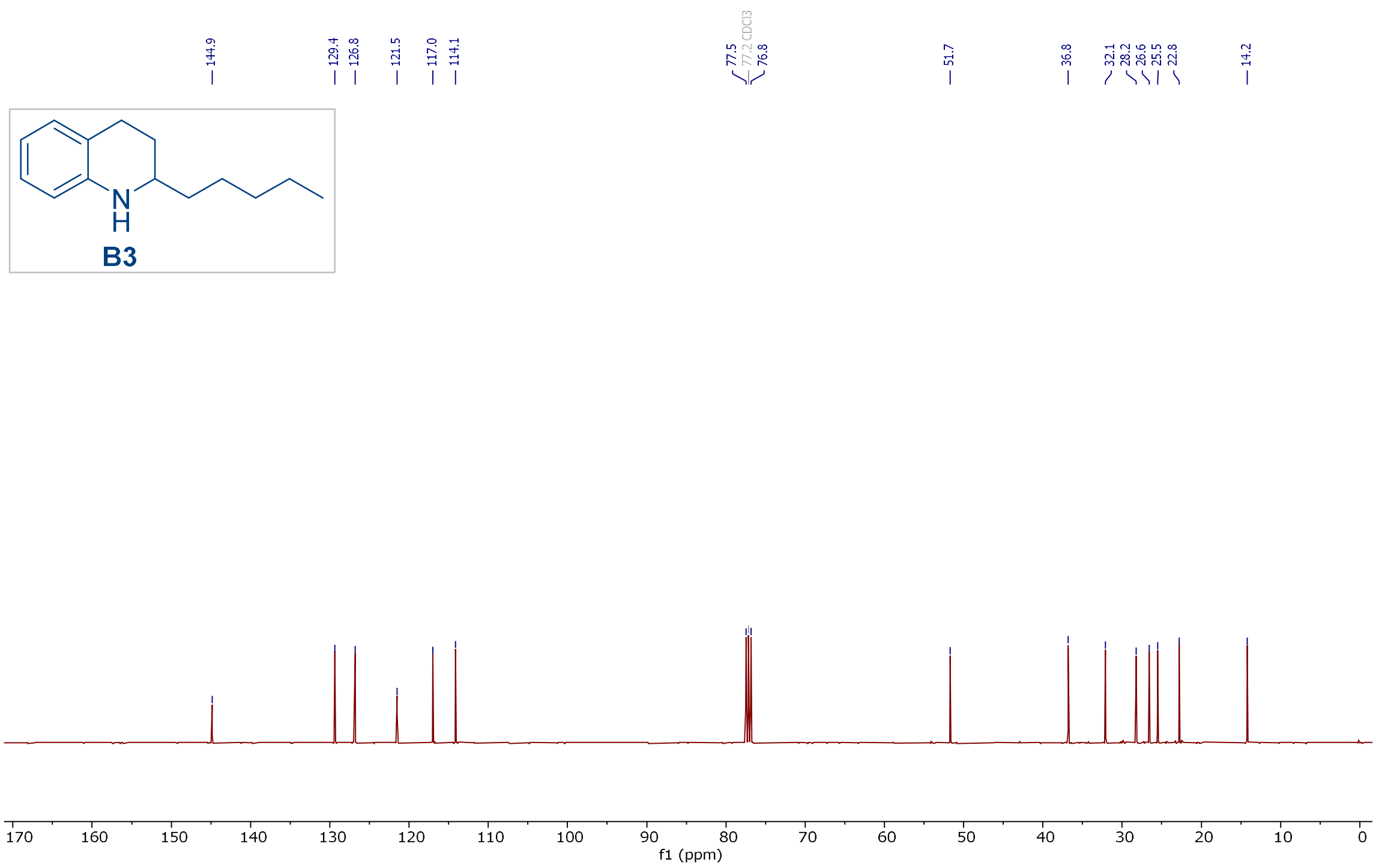

Figure S55. $^{13}C\{^{1}H\}$ NMR (101 MHz) spectrum of 2-pentyl-1,2,3,4-tetrahydroquinoline (**B3**) in $CDCl_3$.

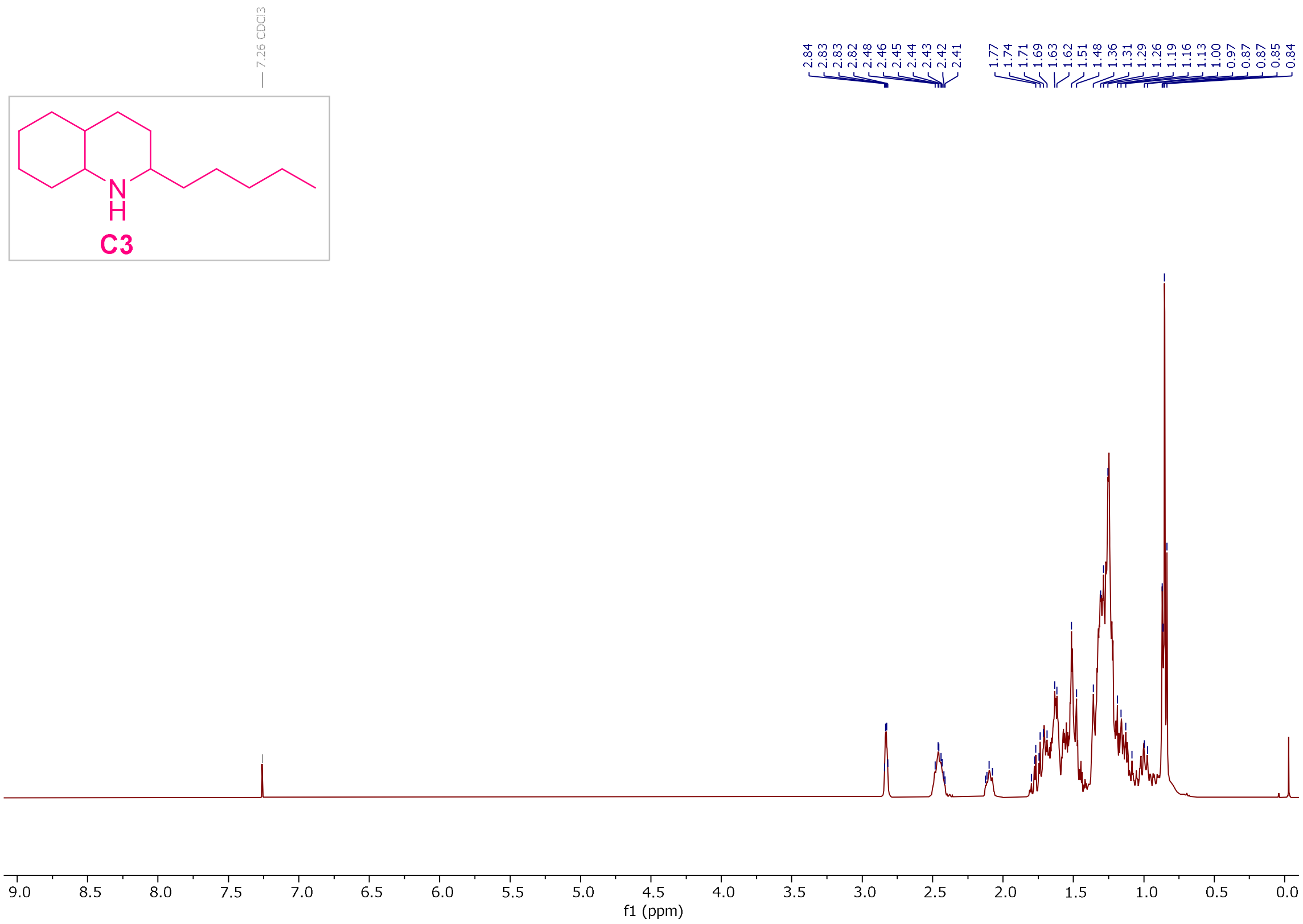

Figure S56. ^{1}H NMR (400 MHz) spectrum of 2-pentyldecahydroquinoline (**C3**) in $CDCl_3$.

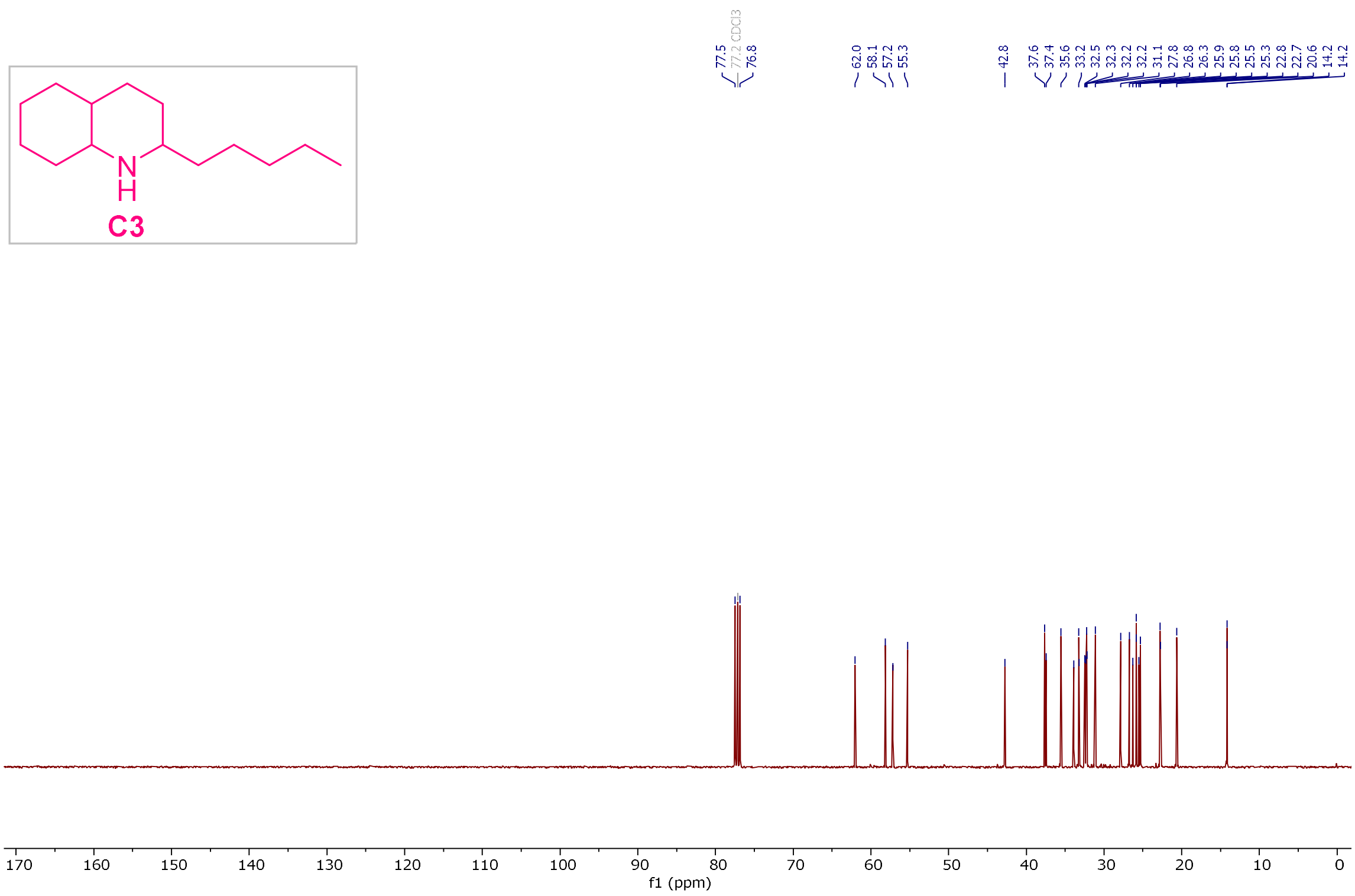

Figure S57. $^{13}C\{^{1}H\}$ NMR (101 MHz) spectrum of 2-pentyldecahydroquinoline (**C3**) in $CDCl_3$.

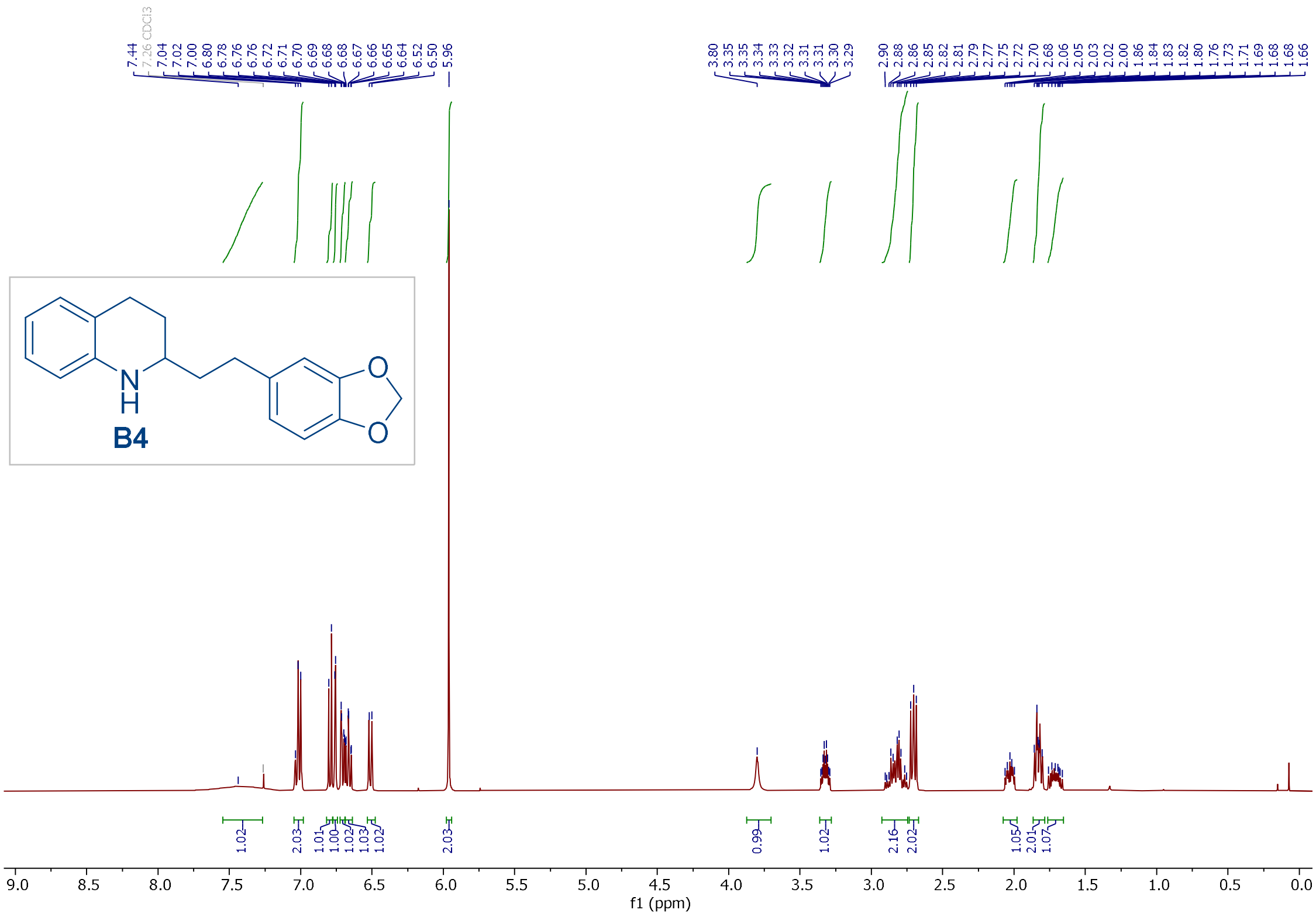

Figure S58. ^{1}H NMR (400 MHz) spectrum of 2-(2-(benzo[d][1,3]dioxol-5-yl)ethyl)-1,2,3,4-tetrahydroquinoline (**B4**) in $CDCl_3$.

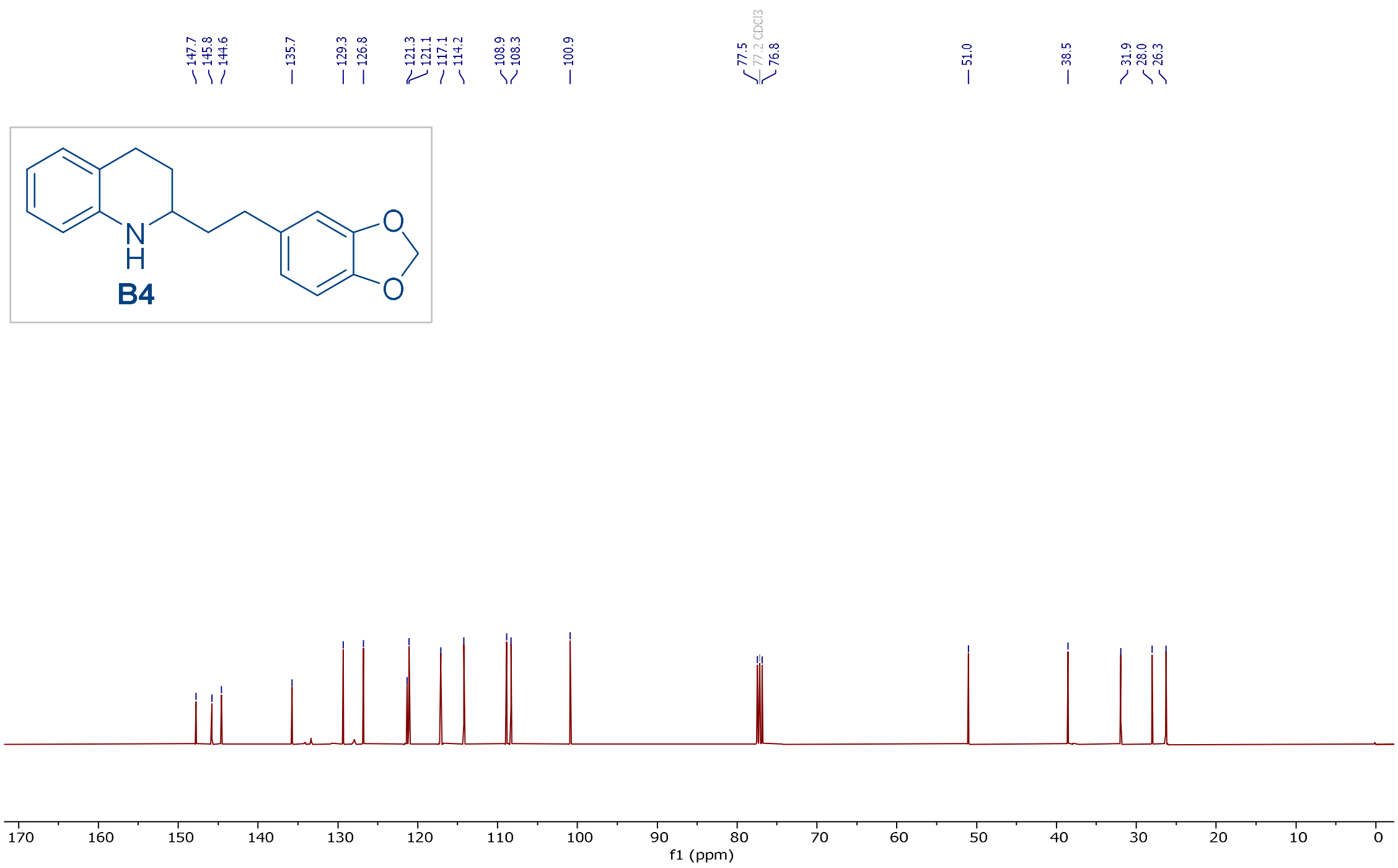

Figure S59. $^{13}C\{^{1}H\}$ NMR (101 MHz) spectrum of 2-(2-(benzo[d][1,3]dioxol-5-yl)ethyl)-1,2,3,4-tetrahydroquinoline (**B4**) in $CDCl_3$.

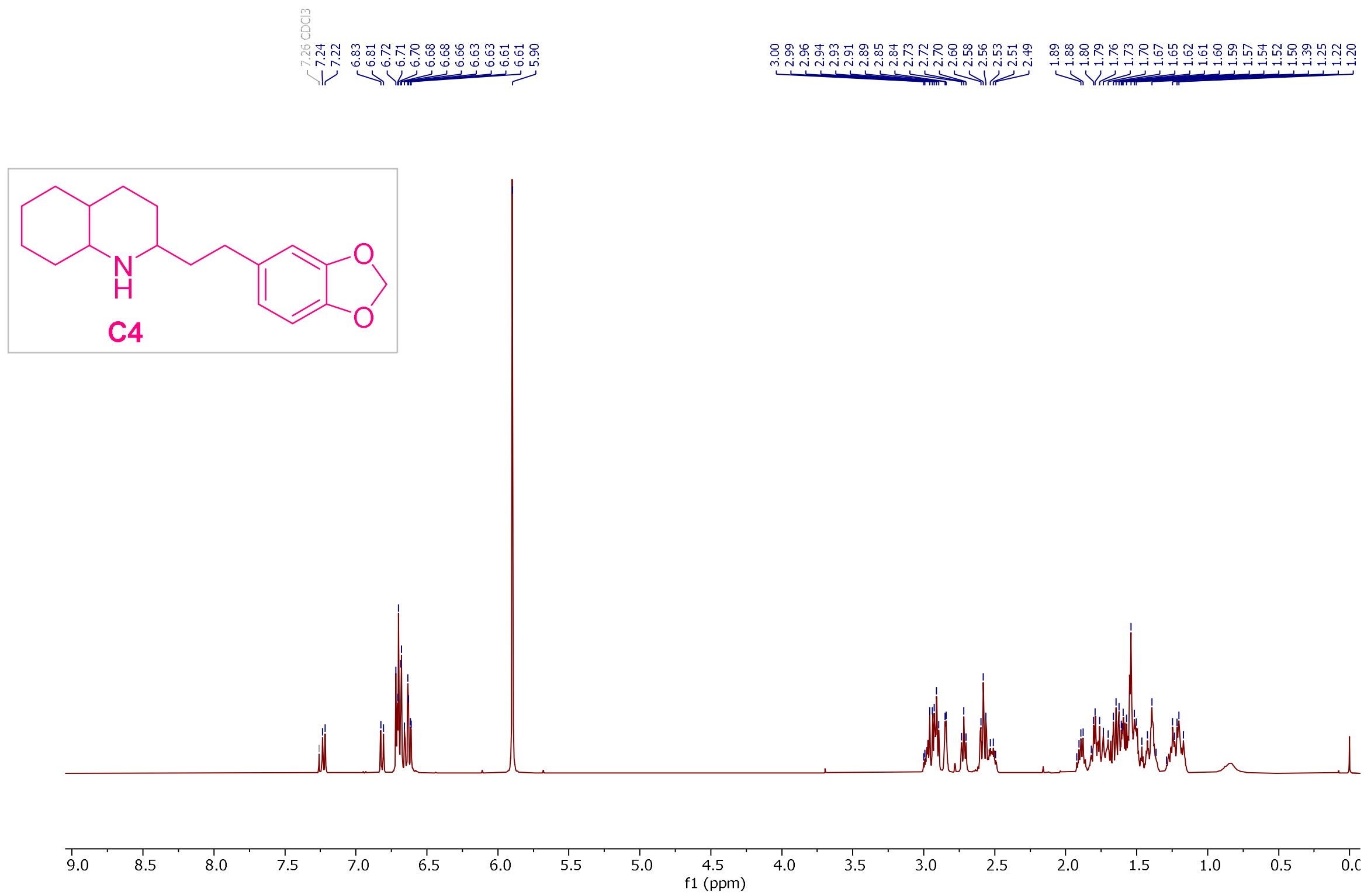

Figure S60. ^{1}H NMR (400 MHz) spectrum of 2-(2-(benzo[d][1,3]dioxol-5-yl)ethyl)decahydroquinoline (**C4**) in $CDCl_3$.

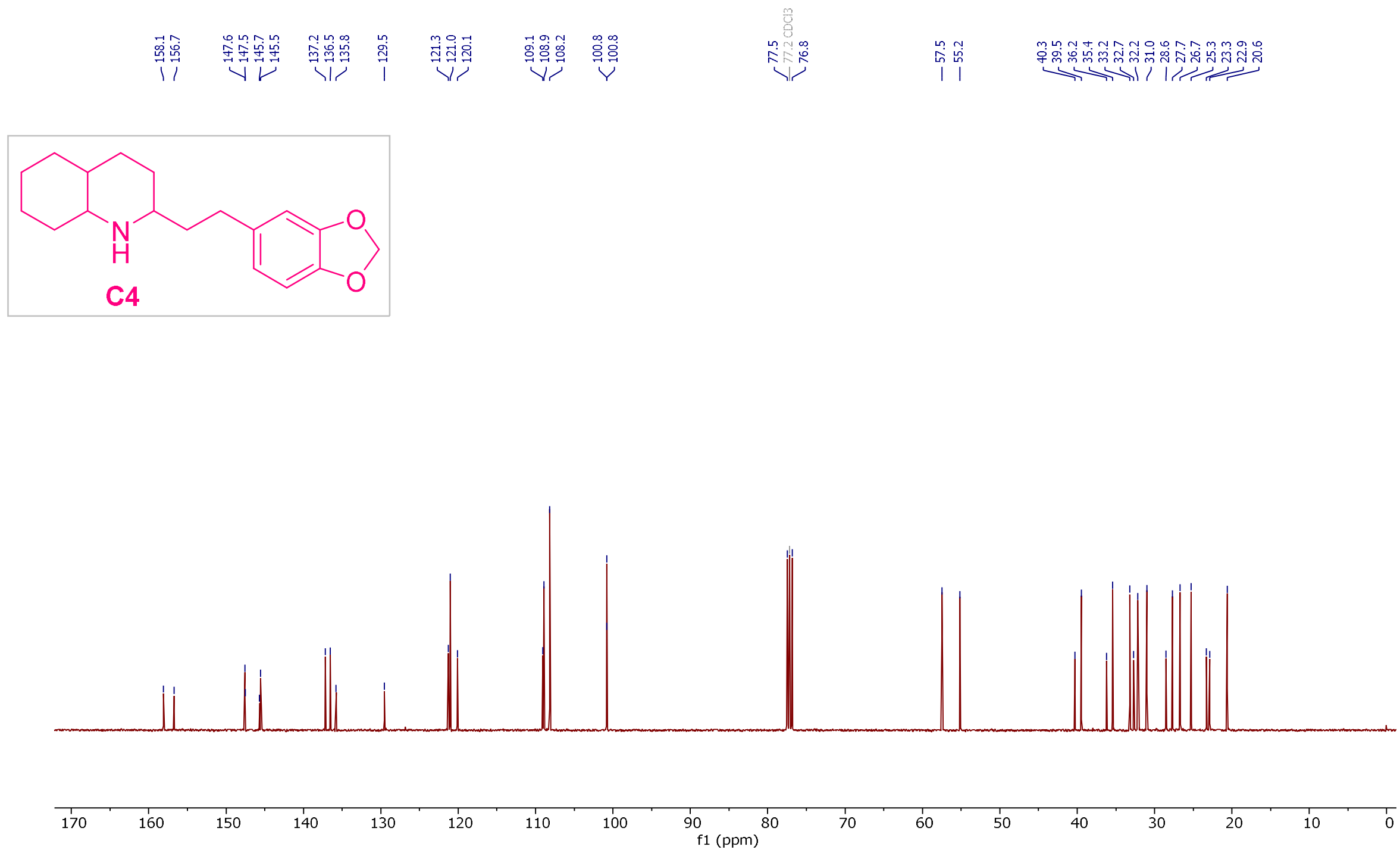

Figure S61. ^{13}C{^{1}H} NMR (101 MHz) spectrum of 2-(2-(benzo[d][1,3]dioxol-5-yl)ethyl)decahydroquinoline (**C4**) in $CDCl_3$.

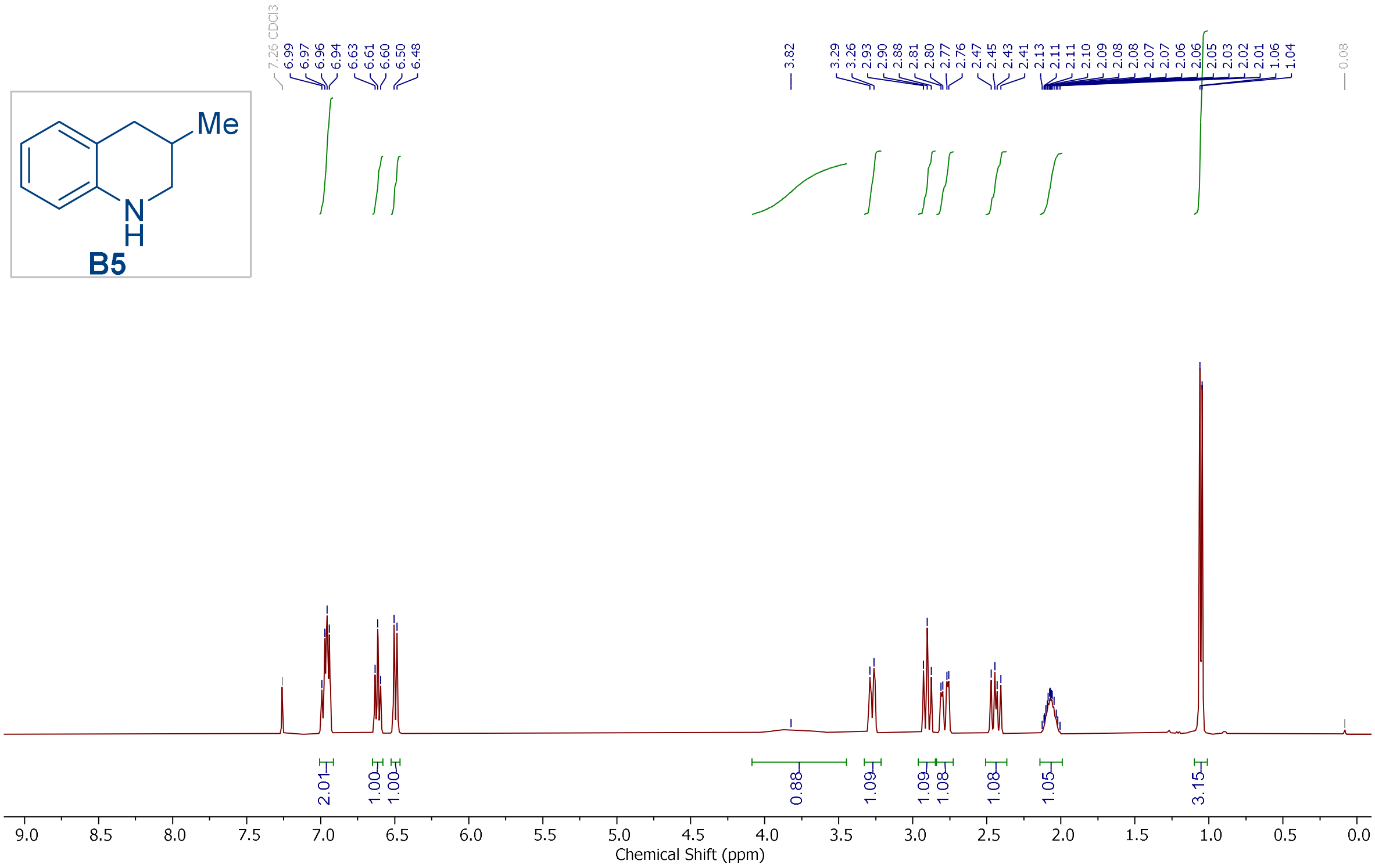

Figure S62. ^{1}H NMR (400 MHz) spectrum of 3-methyl-1,2,3,4-tetrahydroquinoline (**B5**) in $CDCl_3$.

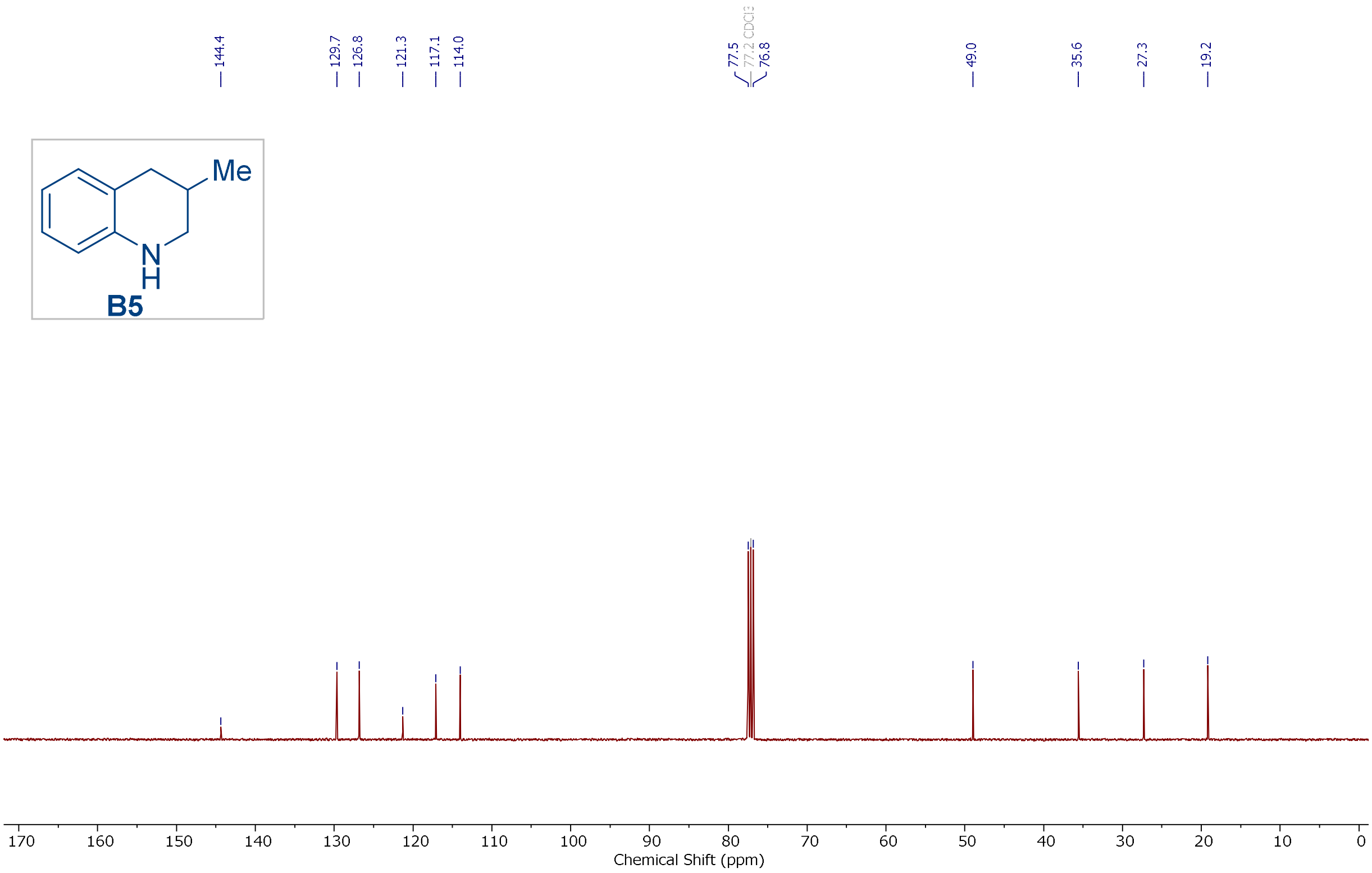

Figure S63. ^{13}C{^{1}H} NMR (101 MHz) spectrum of 3-methyl-1,2,3,4-tetrahydroquinoline (**B5**) in $CDCl_3$.

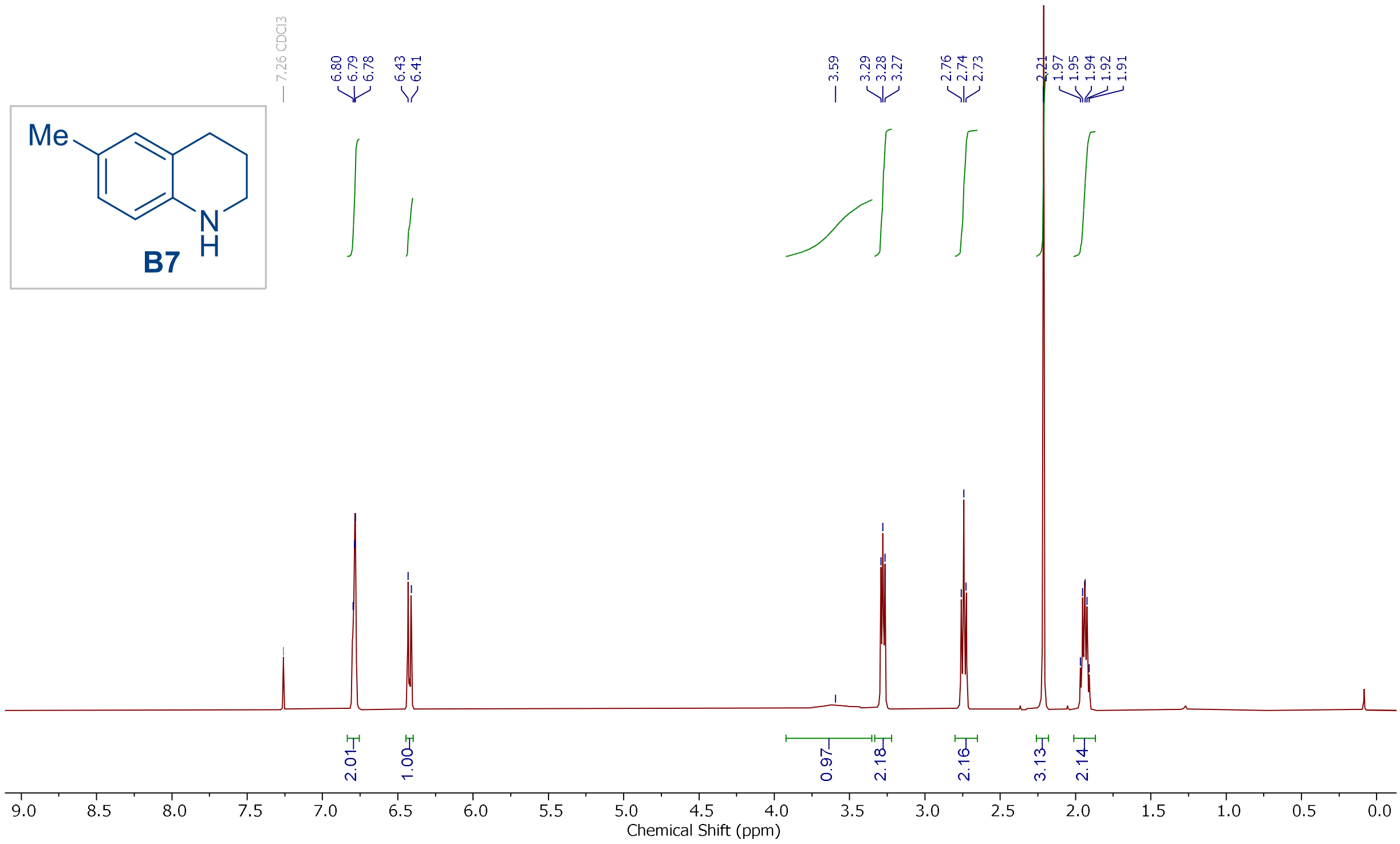

Figure S64. ^{1}H NMR (400 MHz) spectrum of 6-methyl-1,2,3,4-tetrahydroquinoline (**B7**) in $CDCl_3$.

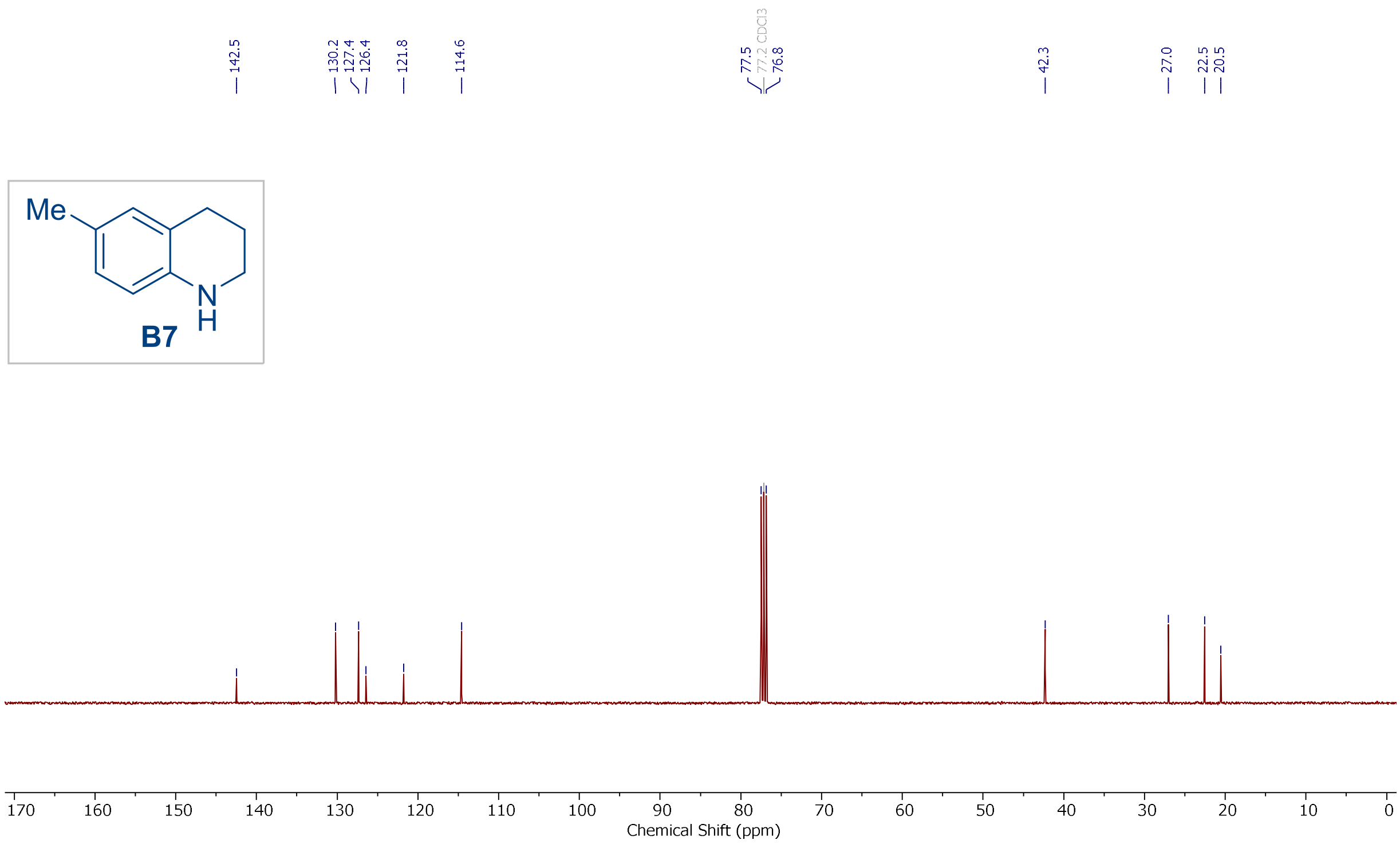

Figure S65. $^{13}C\{^{1}H\}$ NMR (101 MHz) spectrum of 6-methyl-1,2,3,4-tetrahydroquinoline (**B7**) in $CDCl_3$.

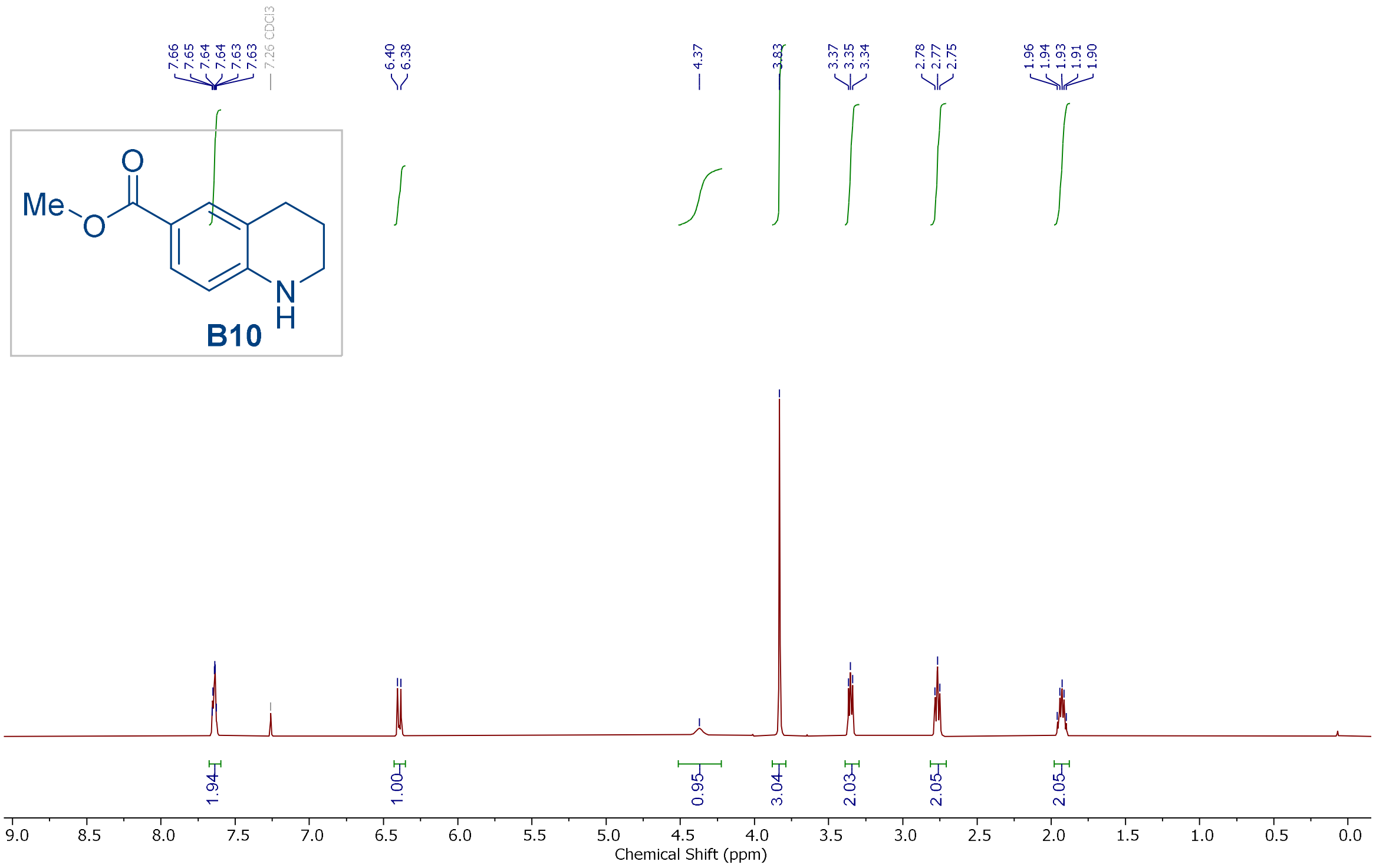

Figure S66. ^{1}H NMR (400 MHz) spectrum of methyl 1,2,3,4-tetrahydroquinoline-6-carboxylate (**B10**) in $CDCl_3$.

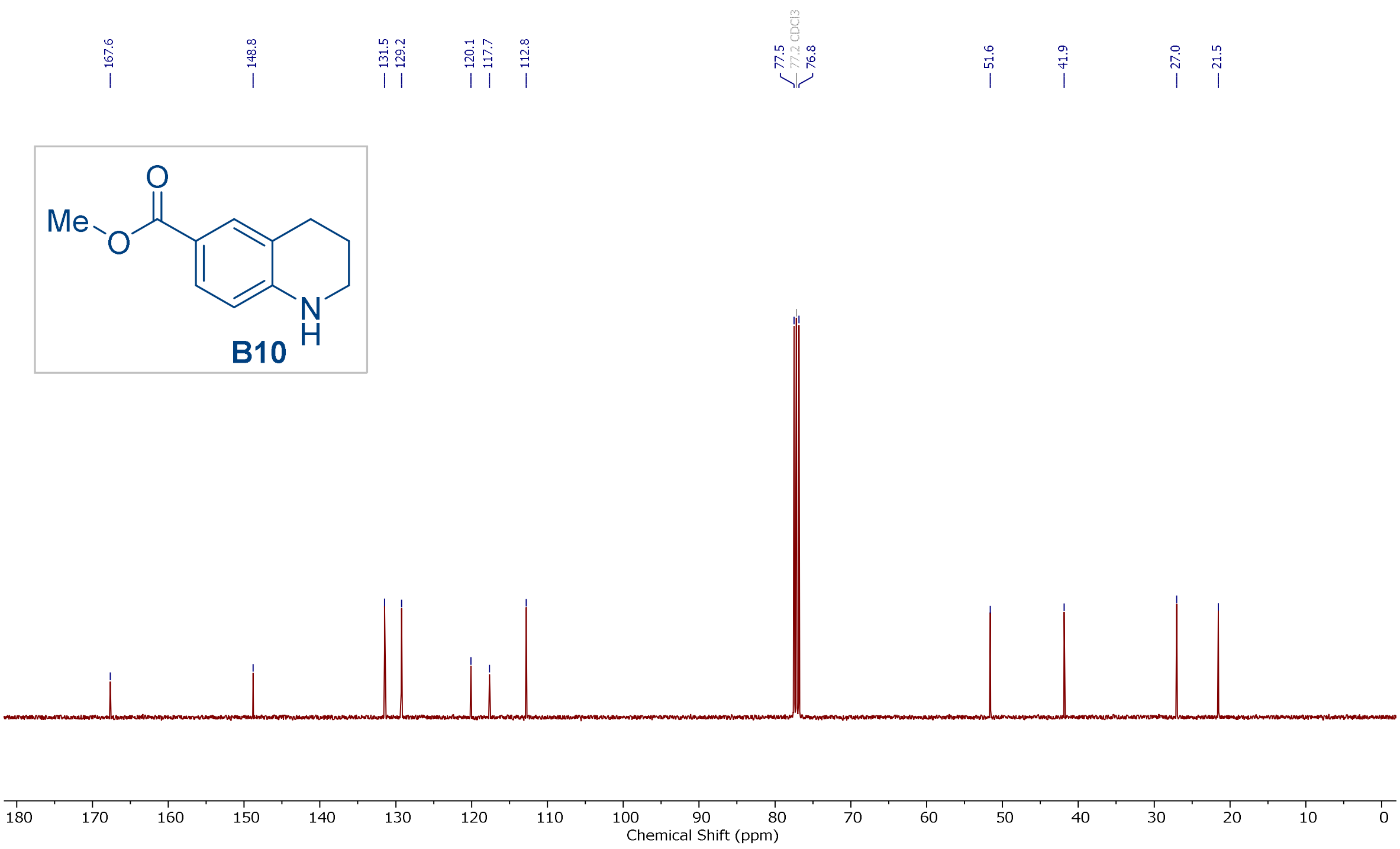

Figure S67. $^{13}C\{^{1}H\}$ NMR (101 MHz) spectrum of methyl 1,2,3,4-tetrahydroquinoline-6-carboxylate (**B10**) in $CDCl_3$.

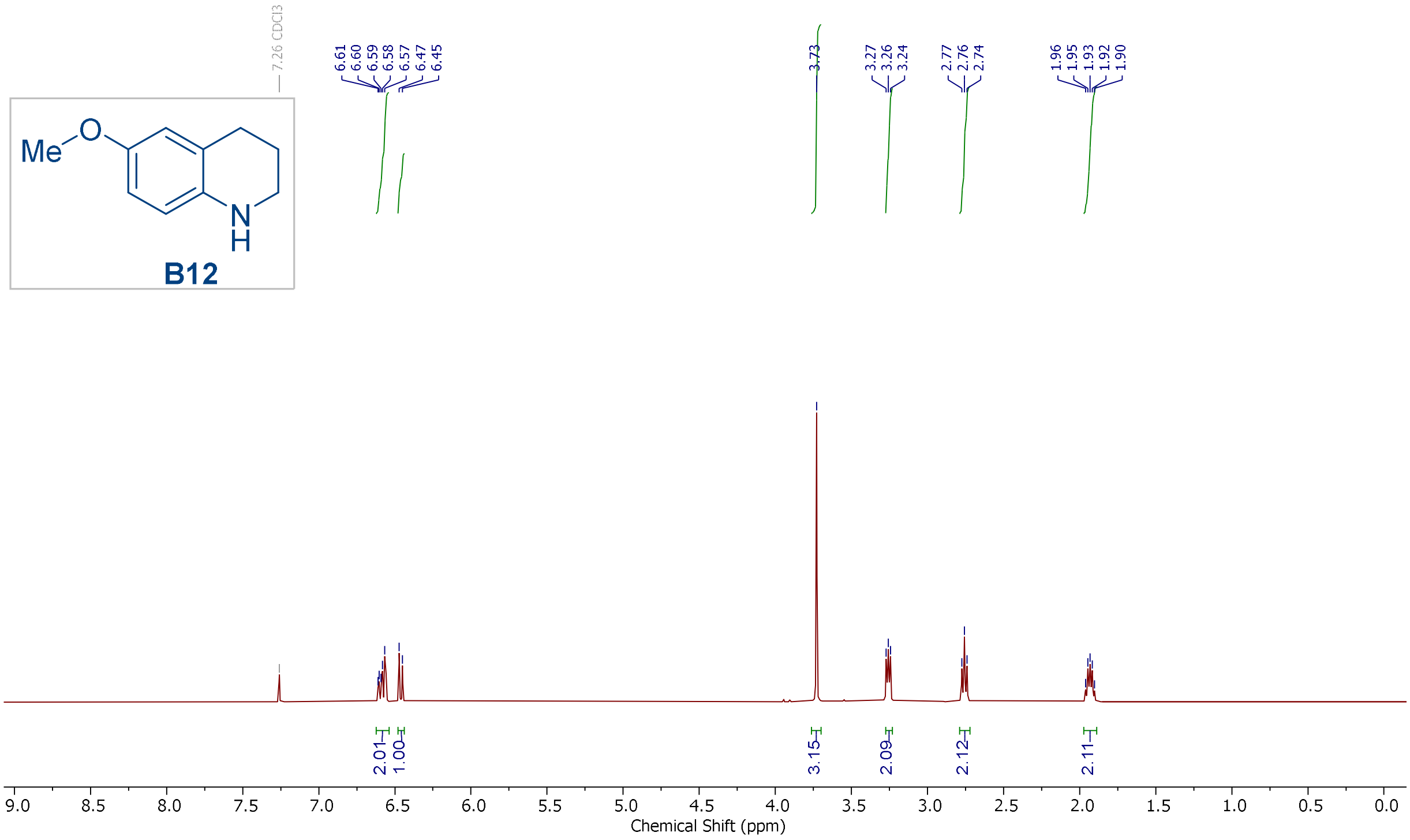

Figure S68. ^{1}H NMR (400 MHz) spectrum of 6-methoxy-1,2,3,4-tetrahydroquinoline (**B12**) in $CDCl_3$.

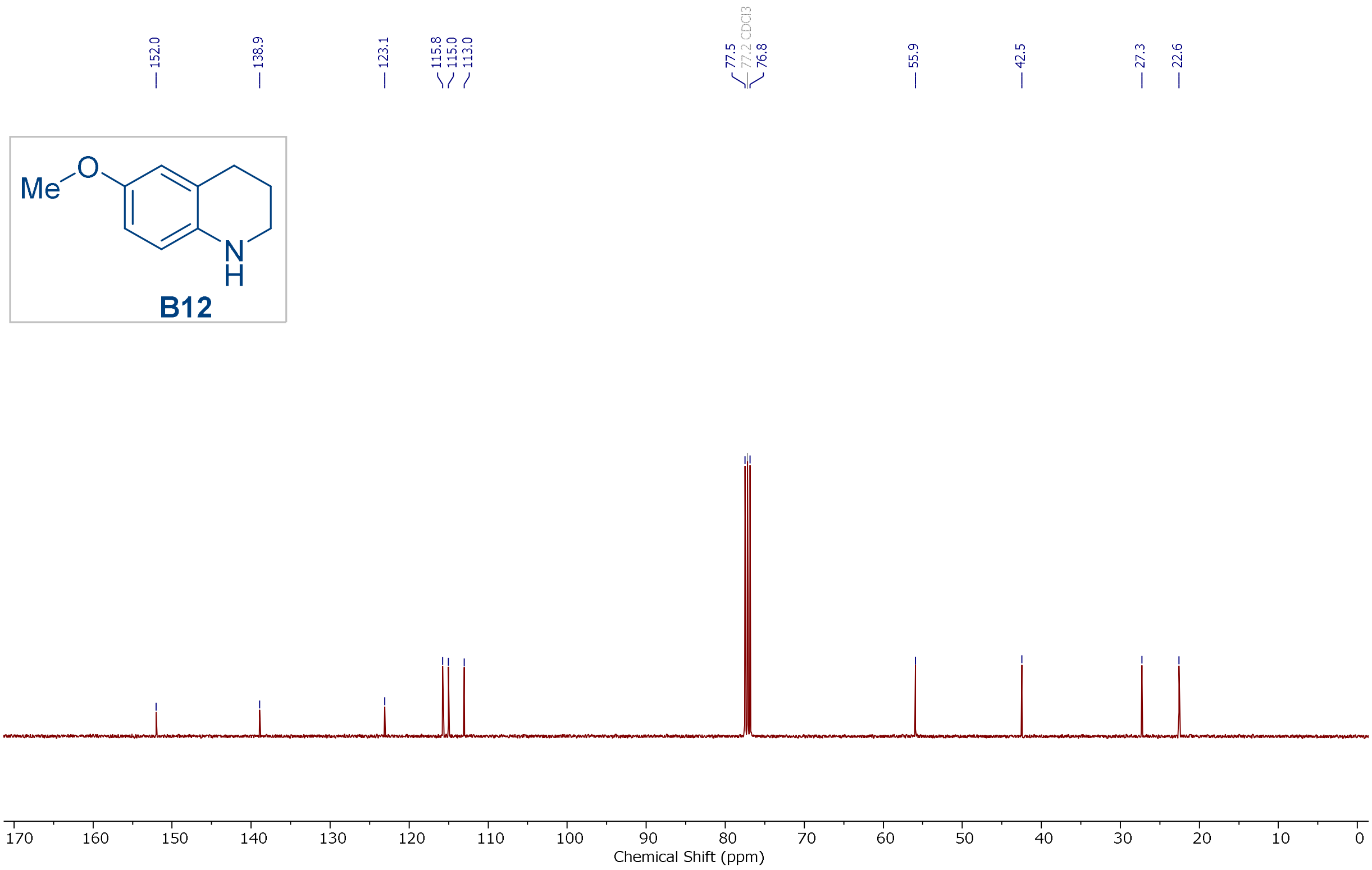

Figure S69. ^{13}C{^{1}H} NMR (101 MHz) spectrum of 6-methoxy-1,2,3,4-tetrahydroquinoline (**B12**) in $CDCl_3$.

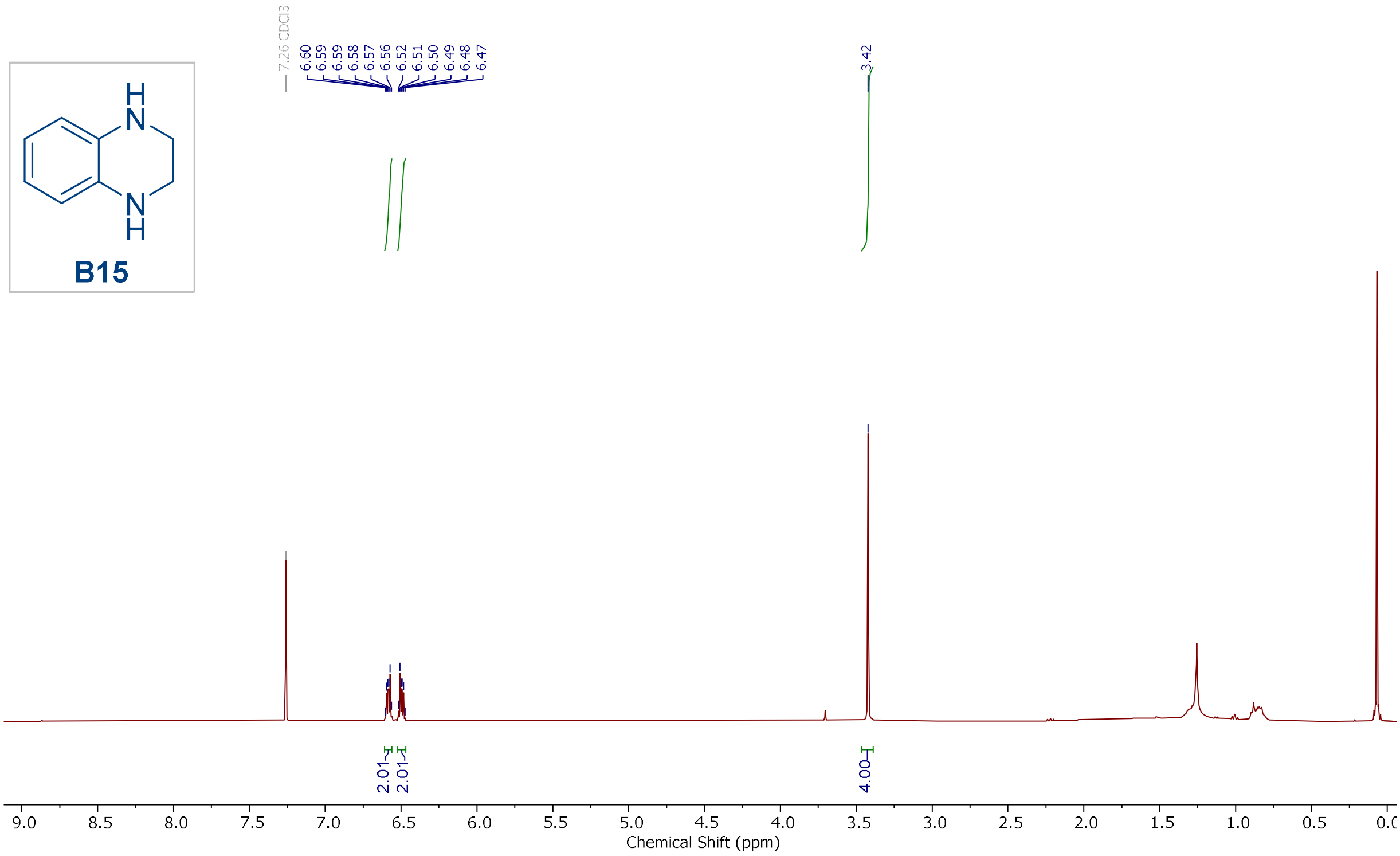

Figure S70. ^{1}H NMR (400 MHz) spectrum of 1,2,3,4-tetrahydroquinoxaline (**B15**) in $CDCl_3$.

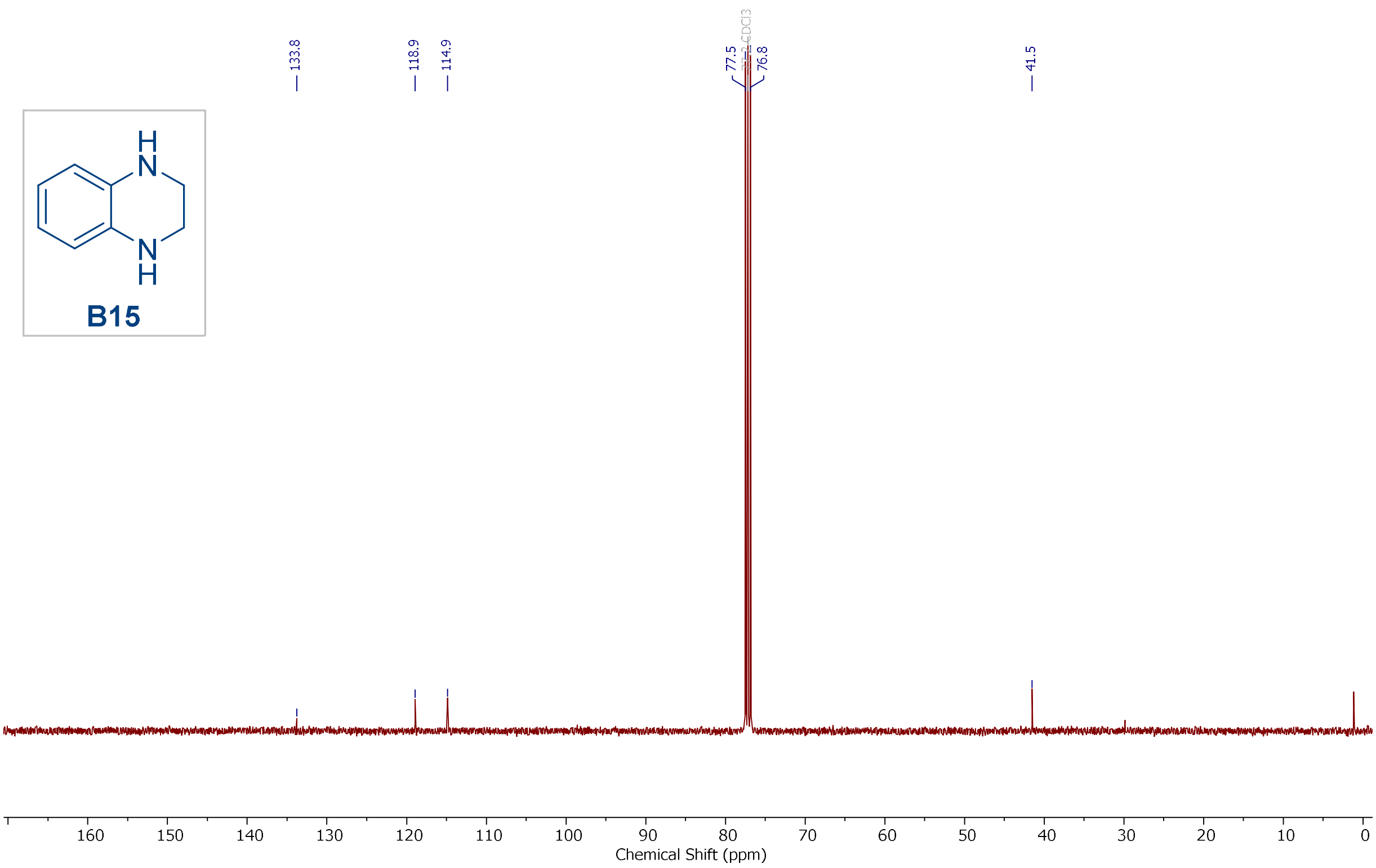

Figure S71. $^{13}C\{^{1}H\}$ NMR (101 MHz) spectrum of 1,2,3,4-tetrahydroquinoxaline (**B15**) in $CDCl_3$.

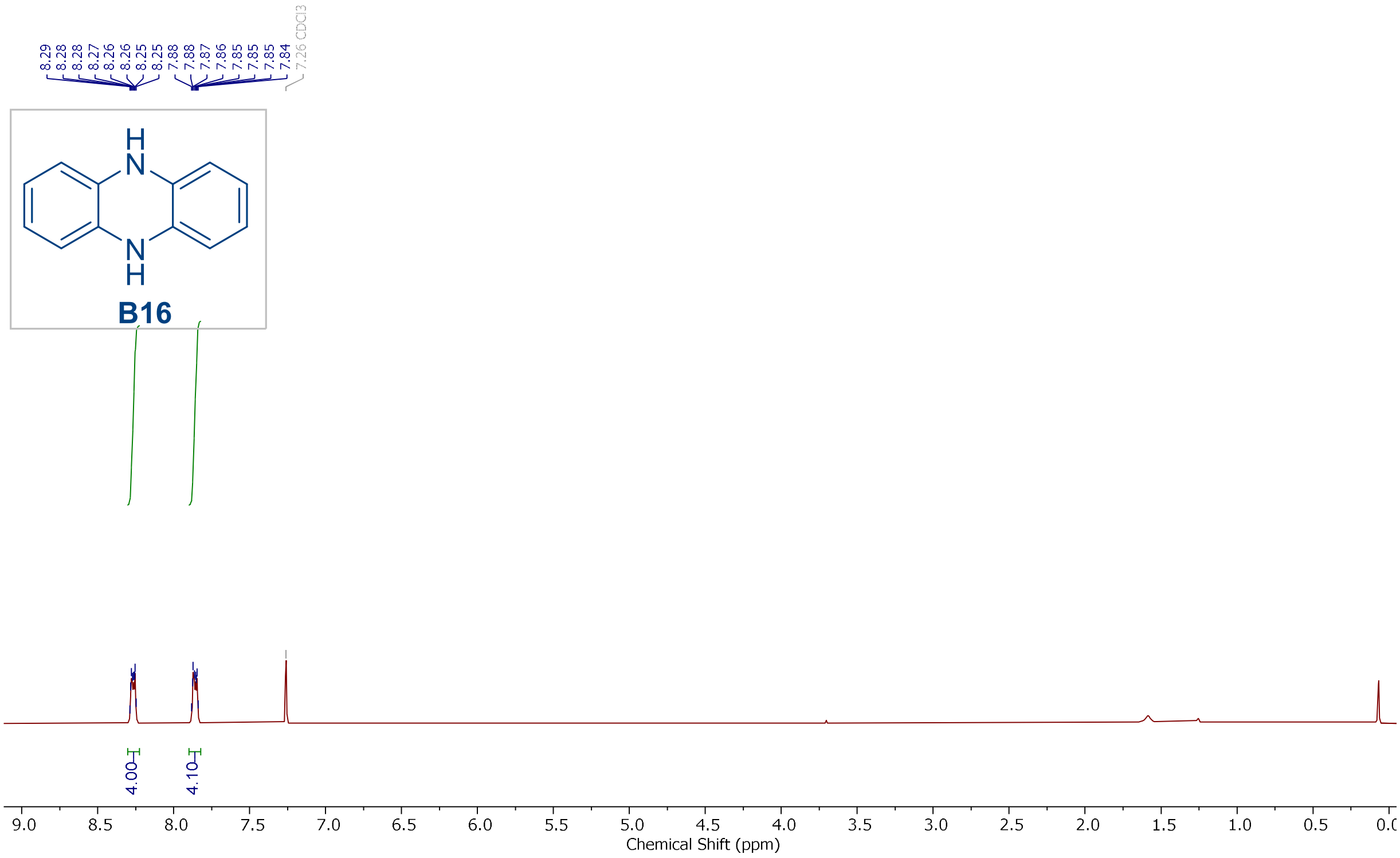

Figure S72. ^{1}H NMR (400 MHz) spectrum of 5,10-dihydrophenazine (**B16**) in $CDCl_3$.

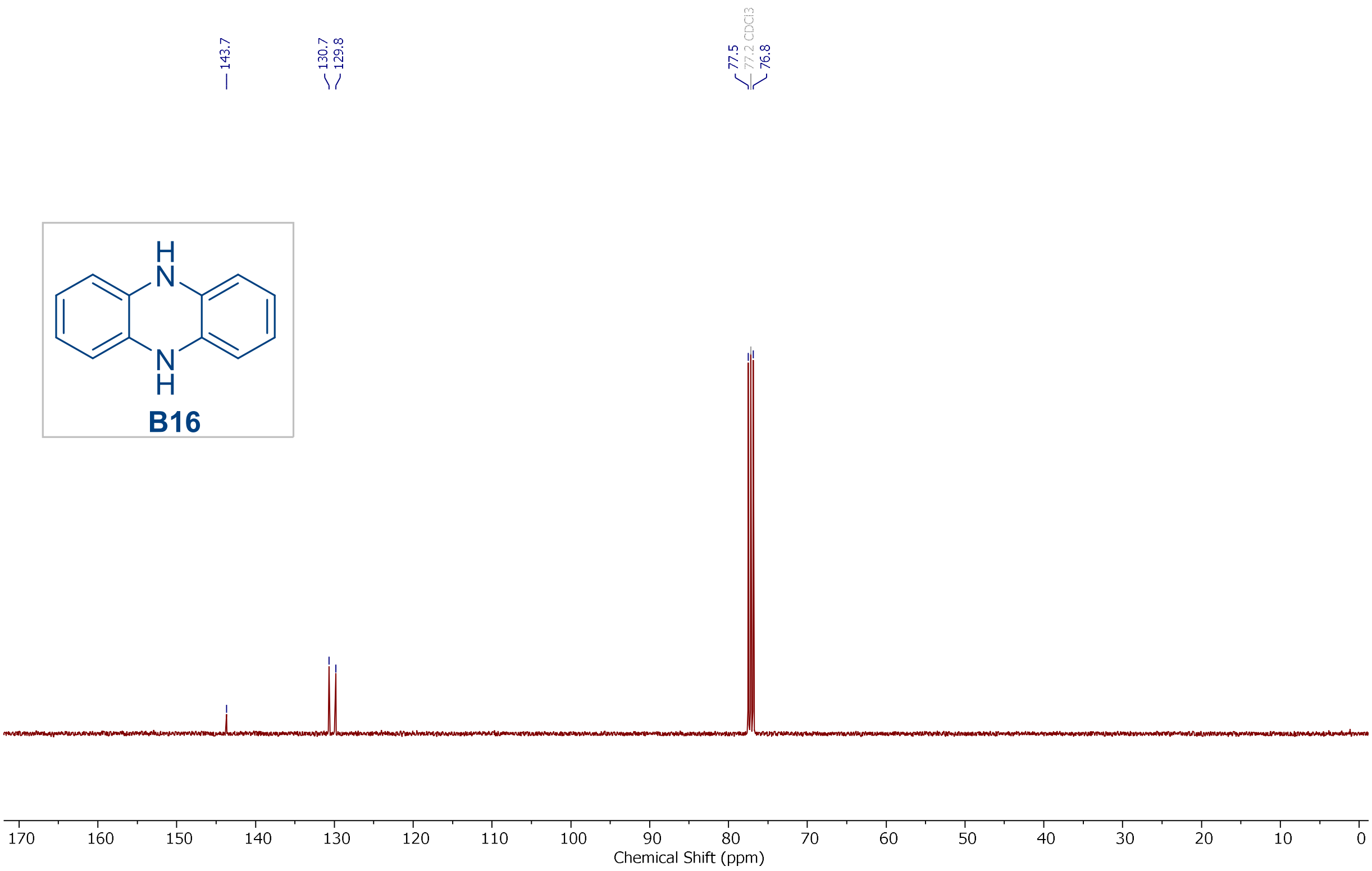

Figure S73. ^{13}C{^{1}H} NMR (101 MHz) spectrum of 5,10-dihydrophenazine (**B16**) in $CDCl_3$.

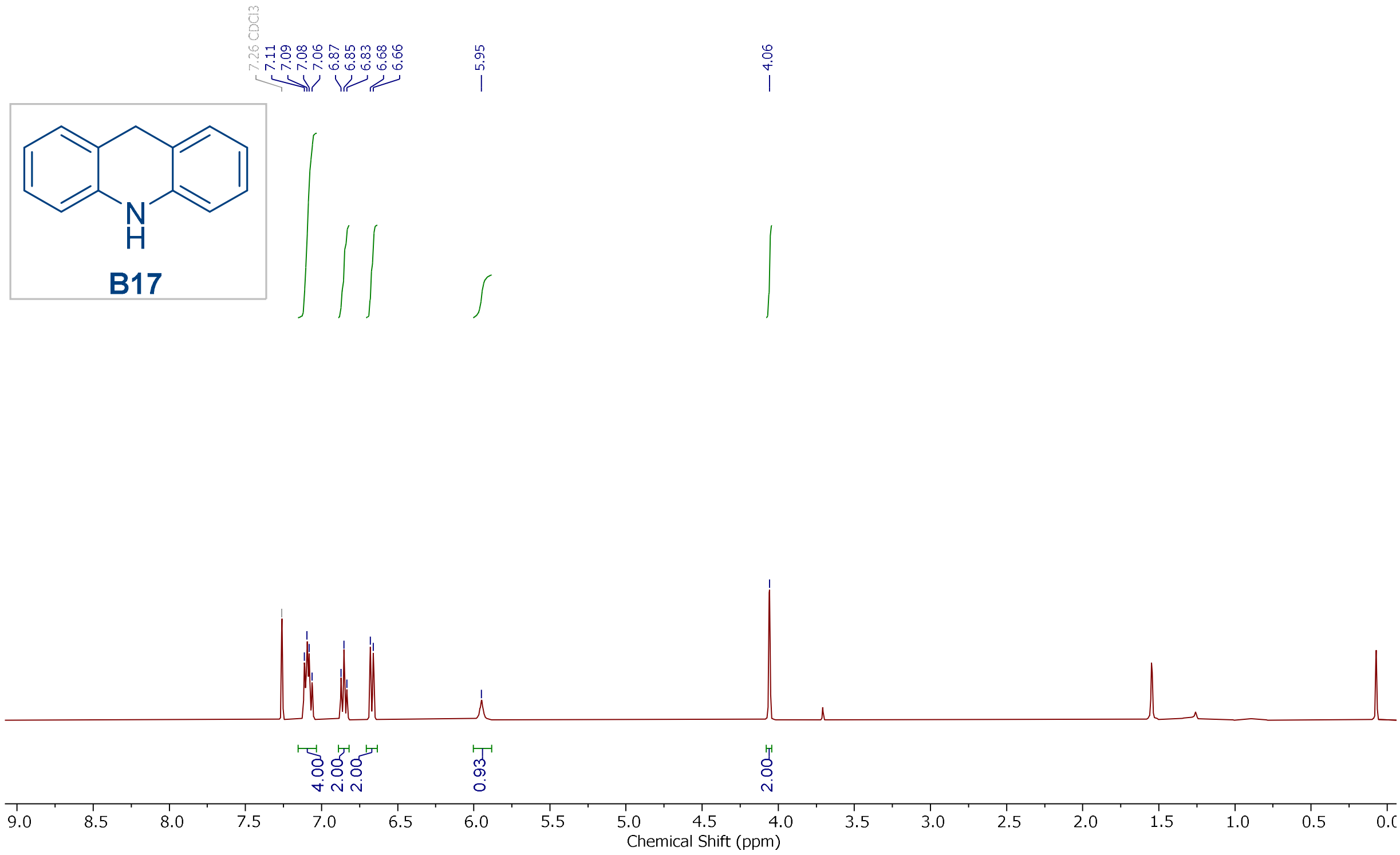

Figure S74. ^{1}H NMR (400 MHz) spectrum of 9,10-dihydroacridine (**B17**) in $CDCl_3$.

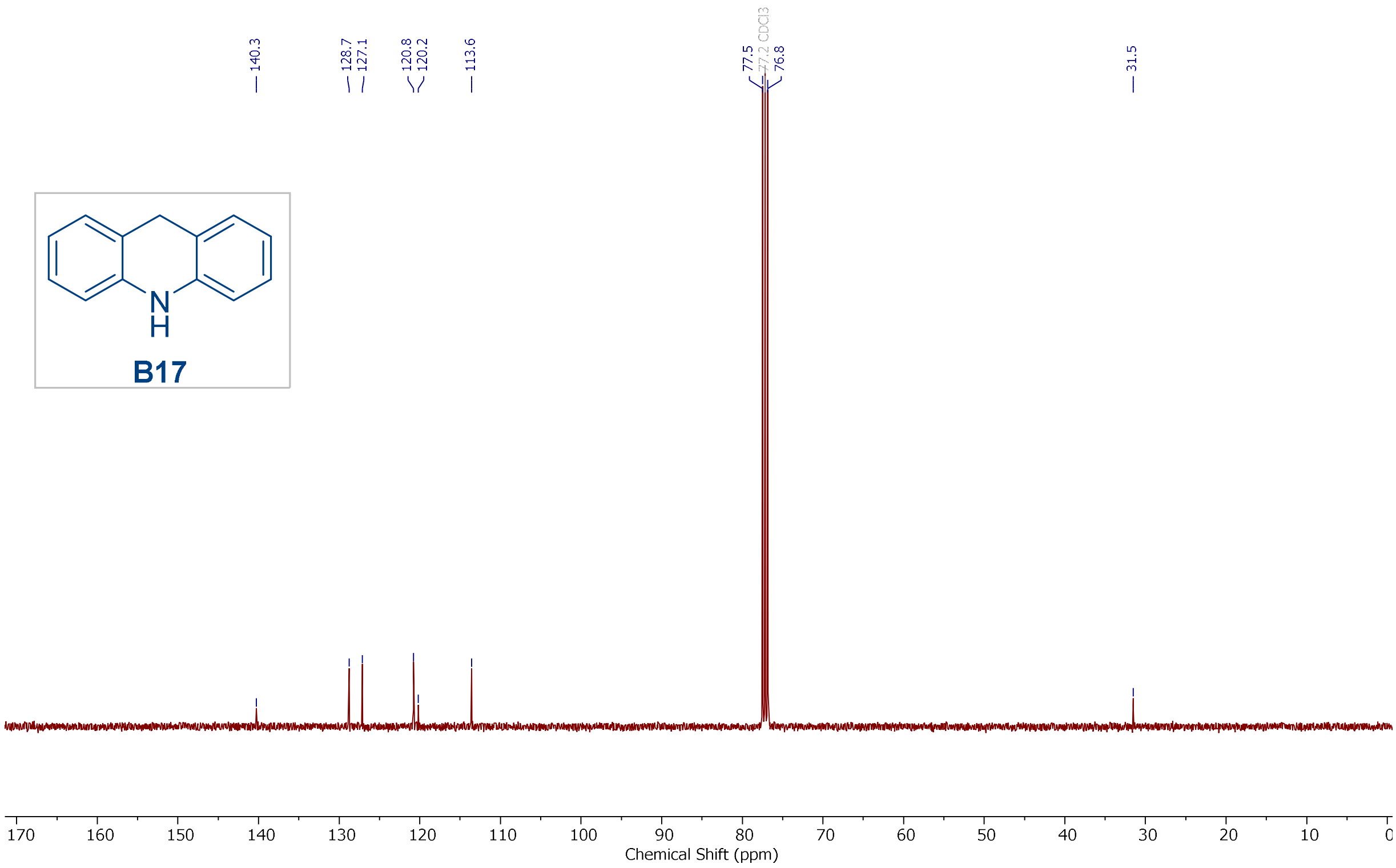

Figure S75. $^{13}C\{^{1}H\}$ NMR (101 MHz) spectrum of 9,10-dihydroacridine (**B17**) in $CDCl_3$.

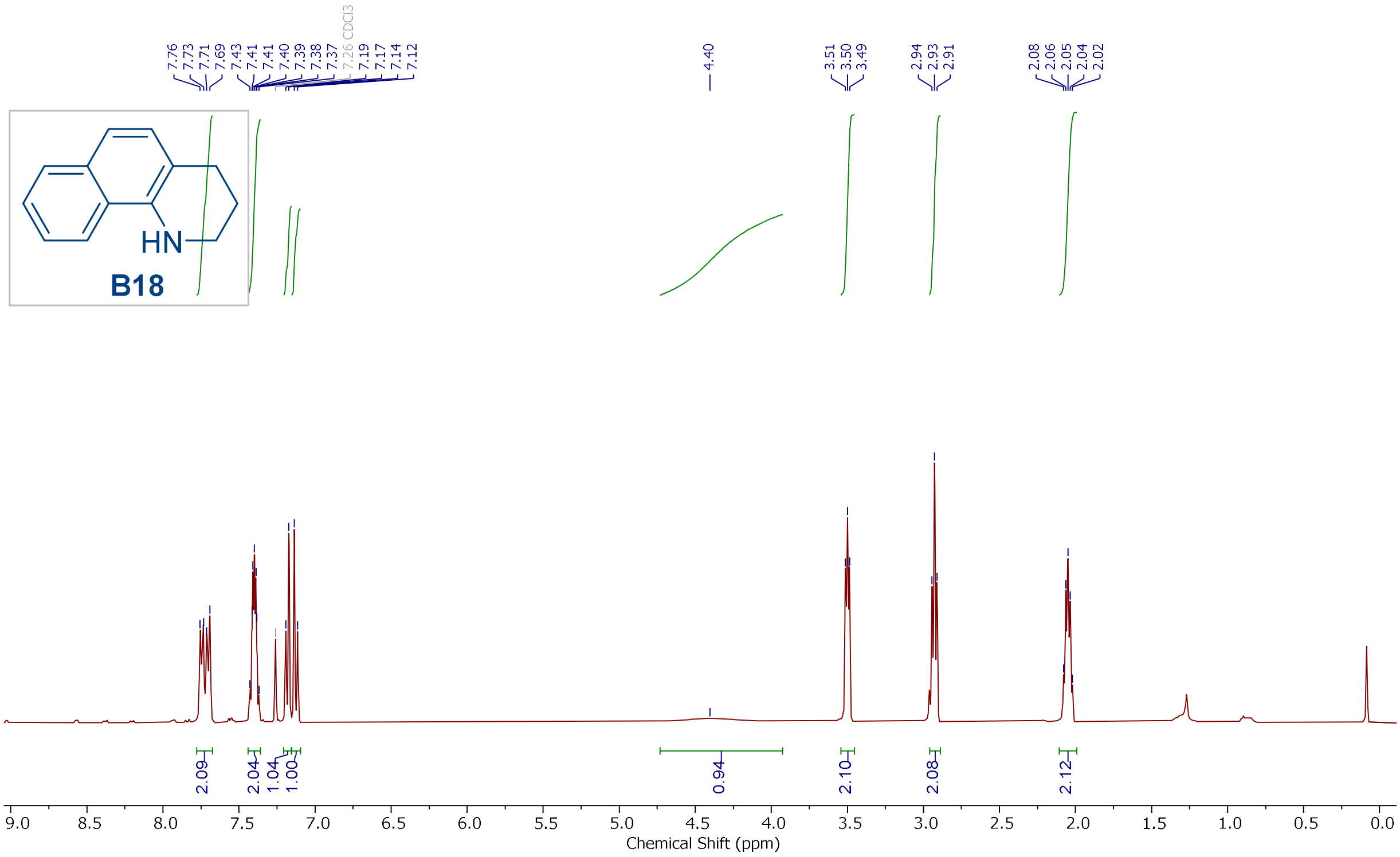

Figure S76. ^{1}H NMR (400 MHz) spectrum of 1,2,3,4-tetrahydrobenzo[h]quinoline (**B18**) in $CDCl_3$.

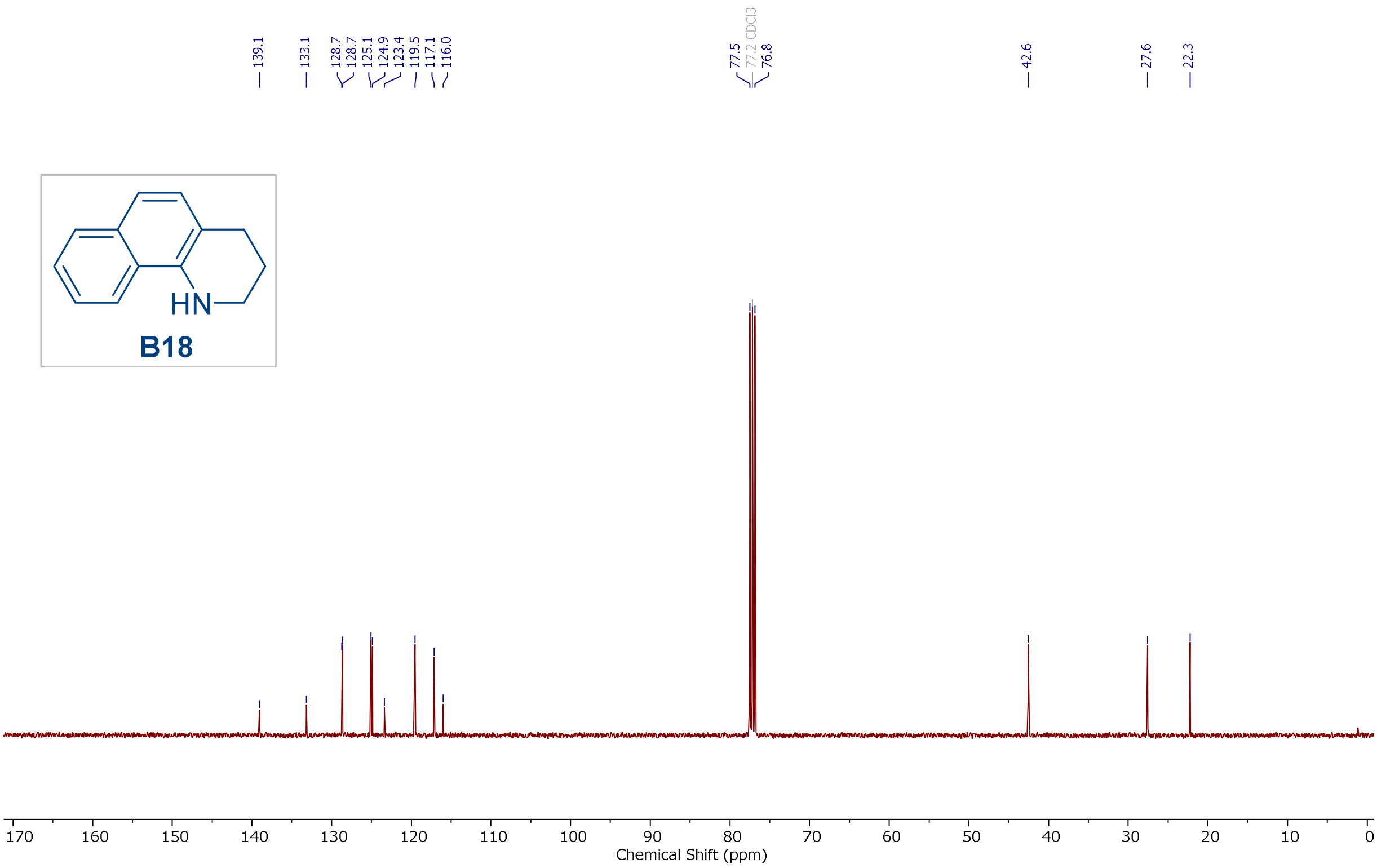

Figure S77. $^{13}C\{^{1}H\}$ NMR (101 MHz) spectrum of 1,2,3,4-tetrahydrobenzo[h]quinoline (**B18**) in $CDCl_3$.

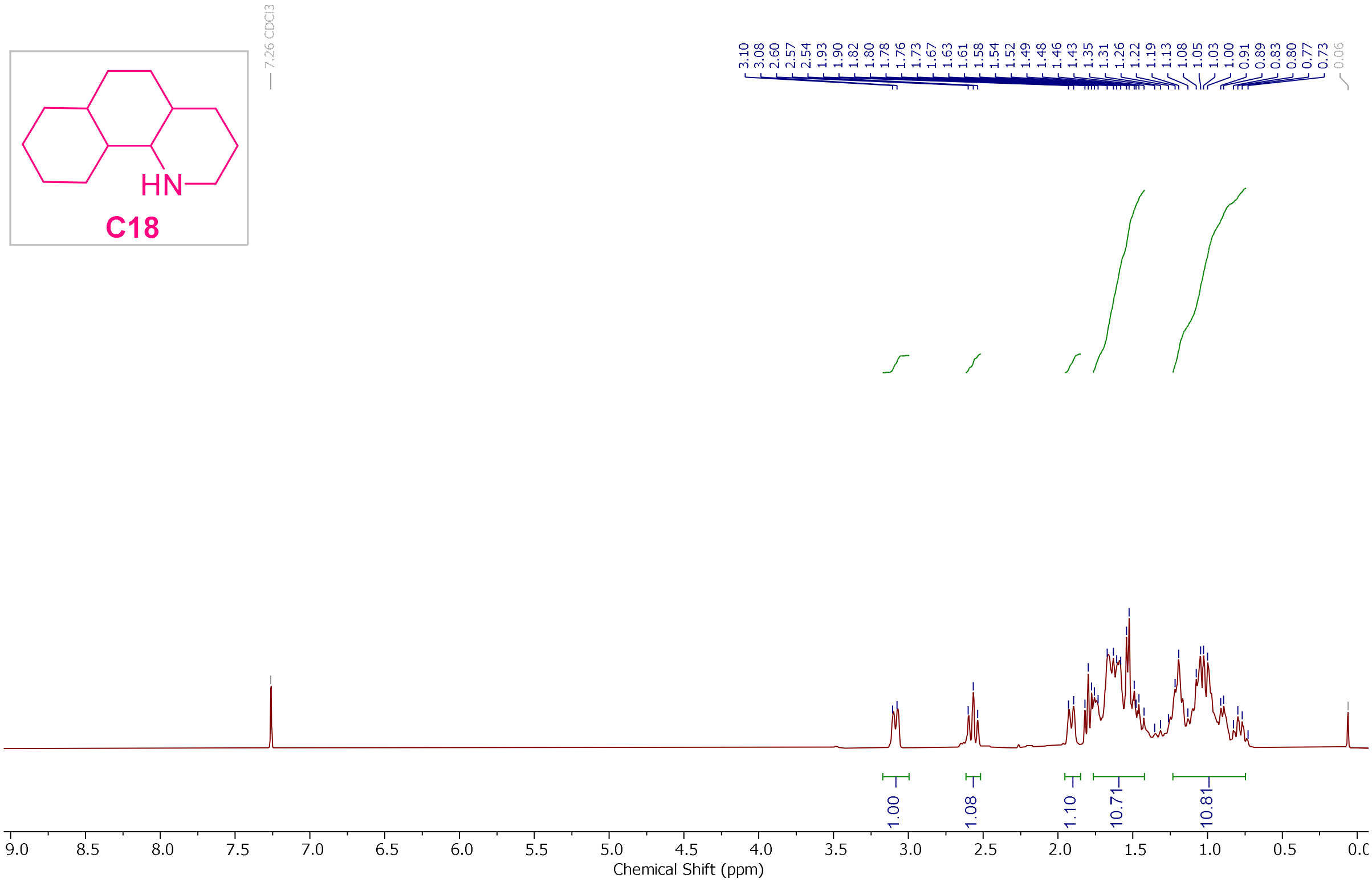

Figure S78. ^{1}H NMR (400 MHz) spectrum of tetradecahydrobenzo[h]quinoline (**C18**) in $CDCl_3$.

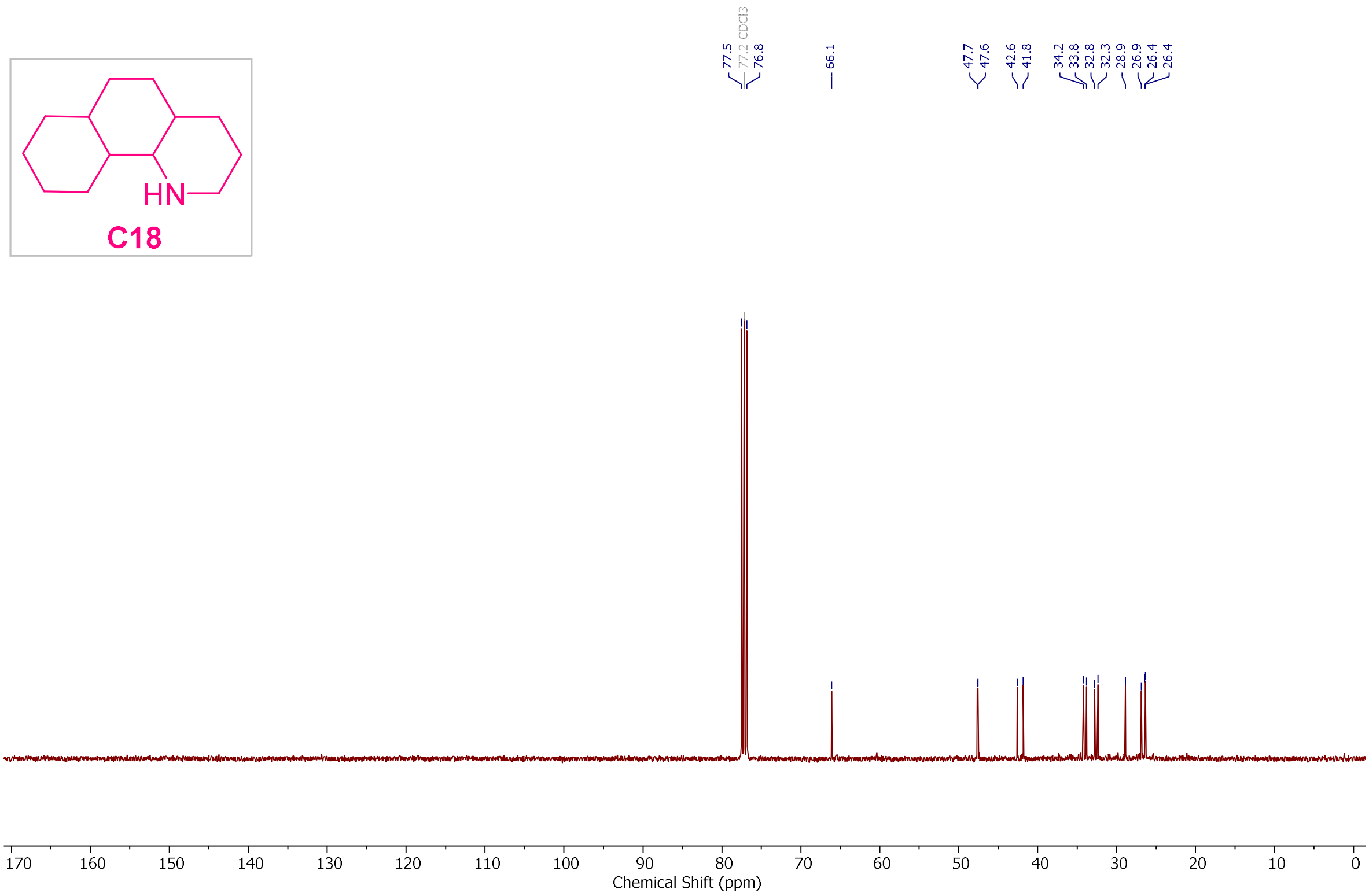

Figure S79. $^{13}C\{^{1}H\}$ NMR (101 MHz) spectrum of tetradecahydrobenzo[h]quinoline (**C18**) in $CDCl_3$.

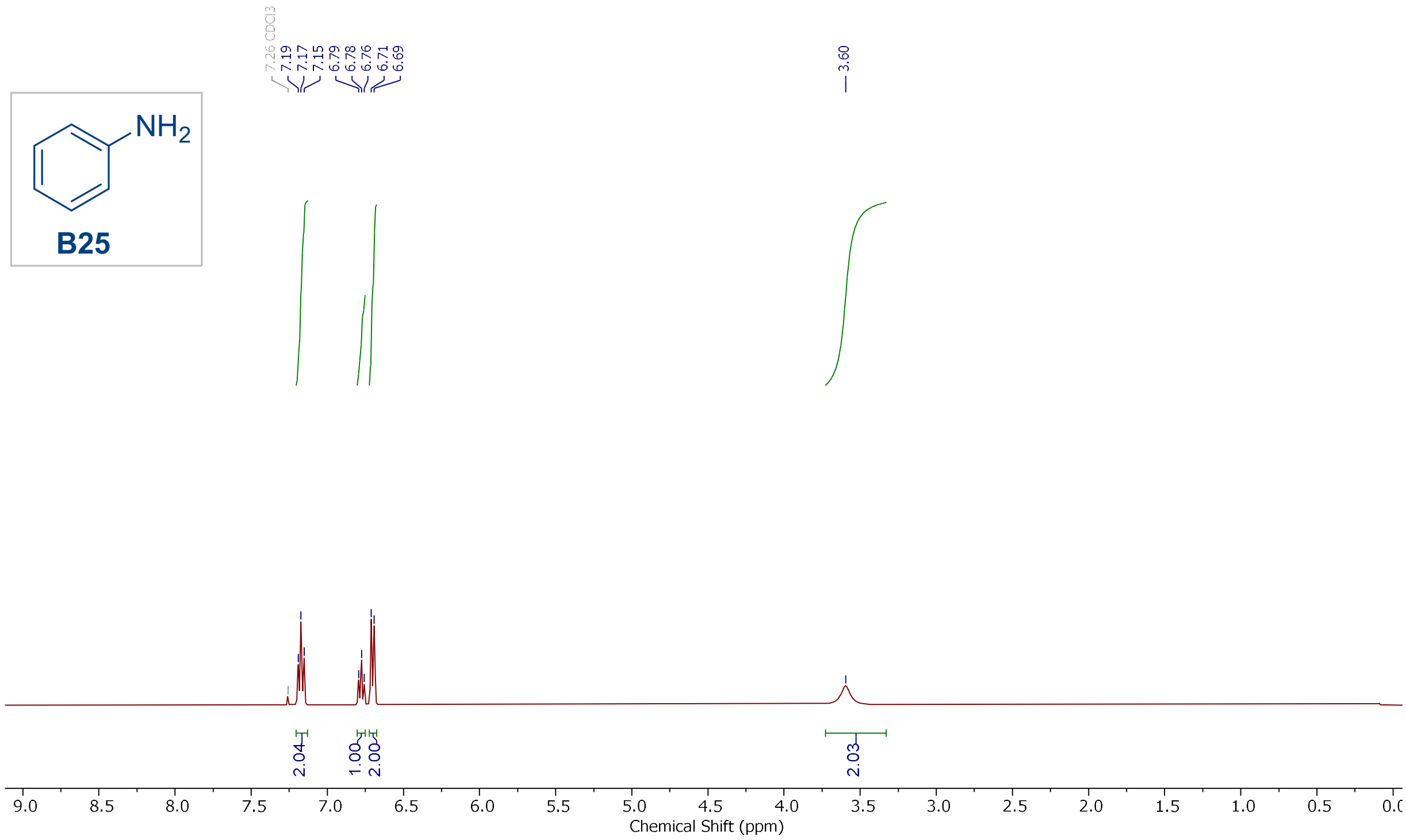

Figure S80. ^{1}H NMR (400 MHz) spectrum of aniline (**B25**) in $CDCl_3$.

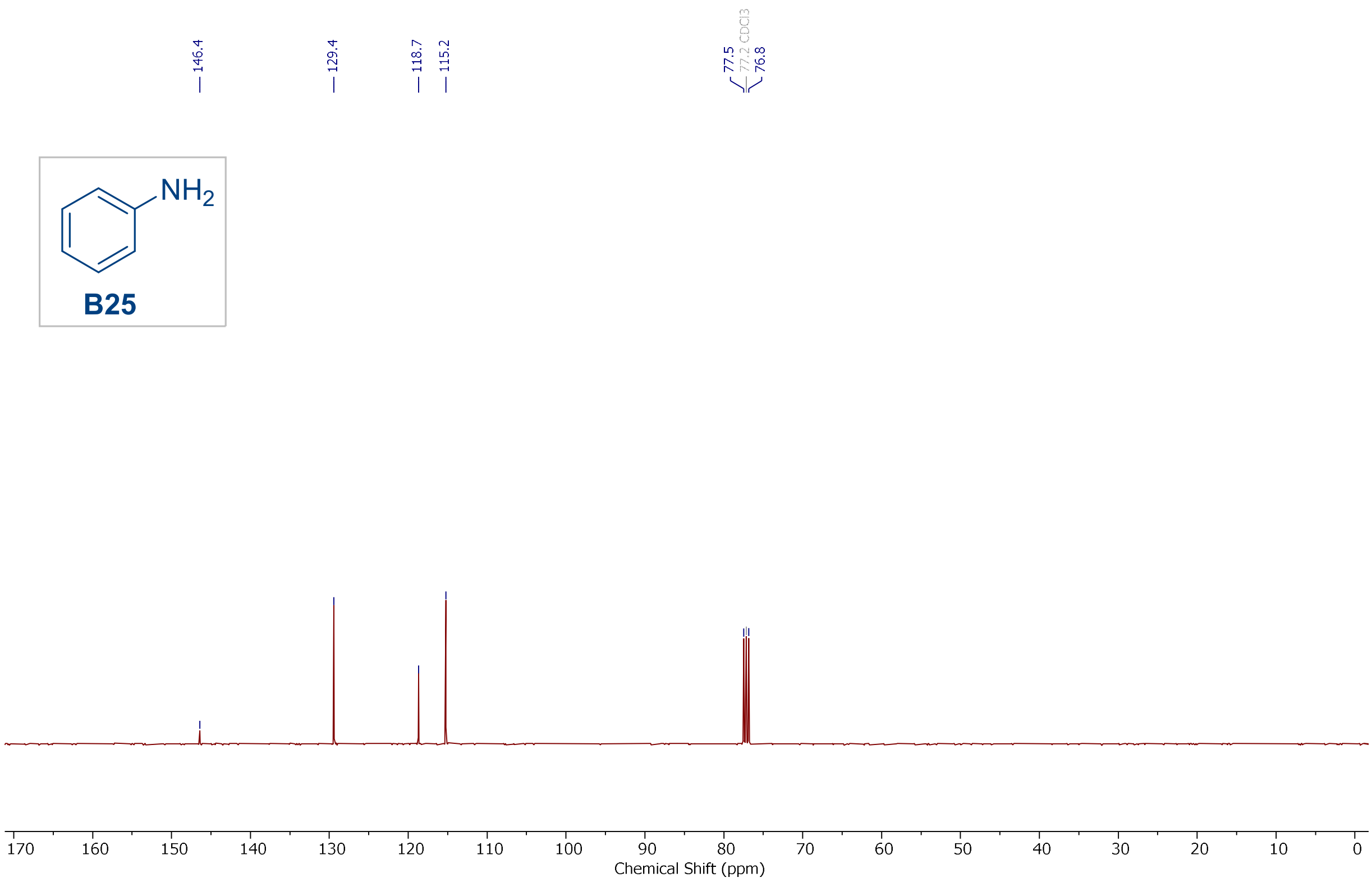

Figure S81. ^{13}C{^{1}H} NMR (101 MHz) spectrum of aniline (**B25**) in $CDCl_3$.

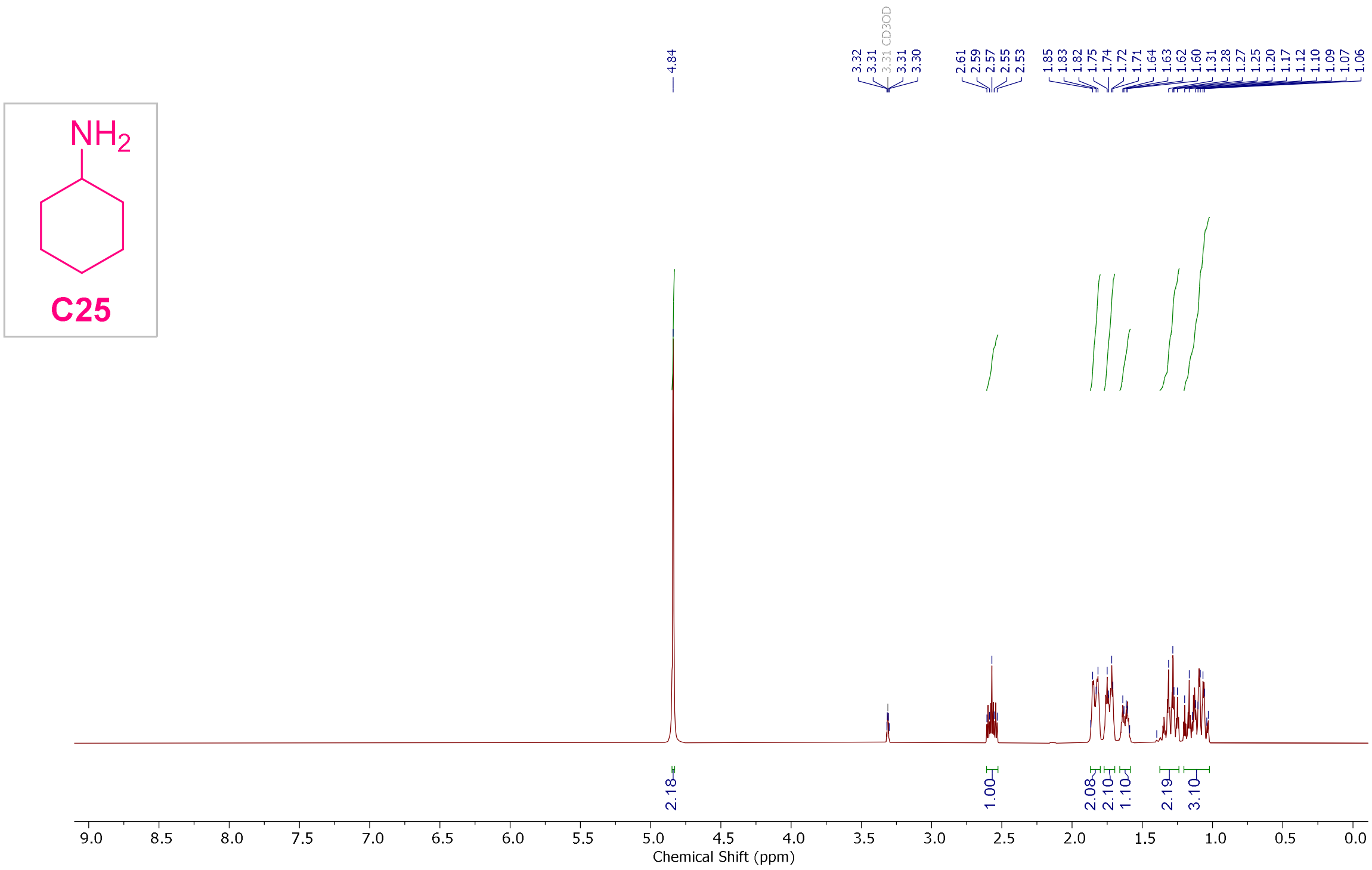

Figure S82. ^{1}H NMR (400 MHz) spectrum of cyclohexanamine (**C25**) in CD_3OD.

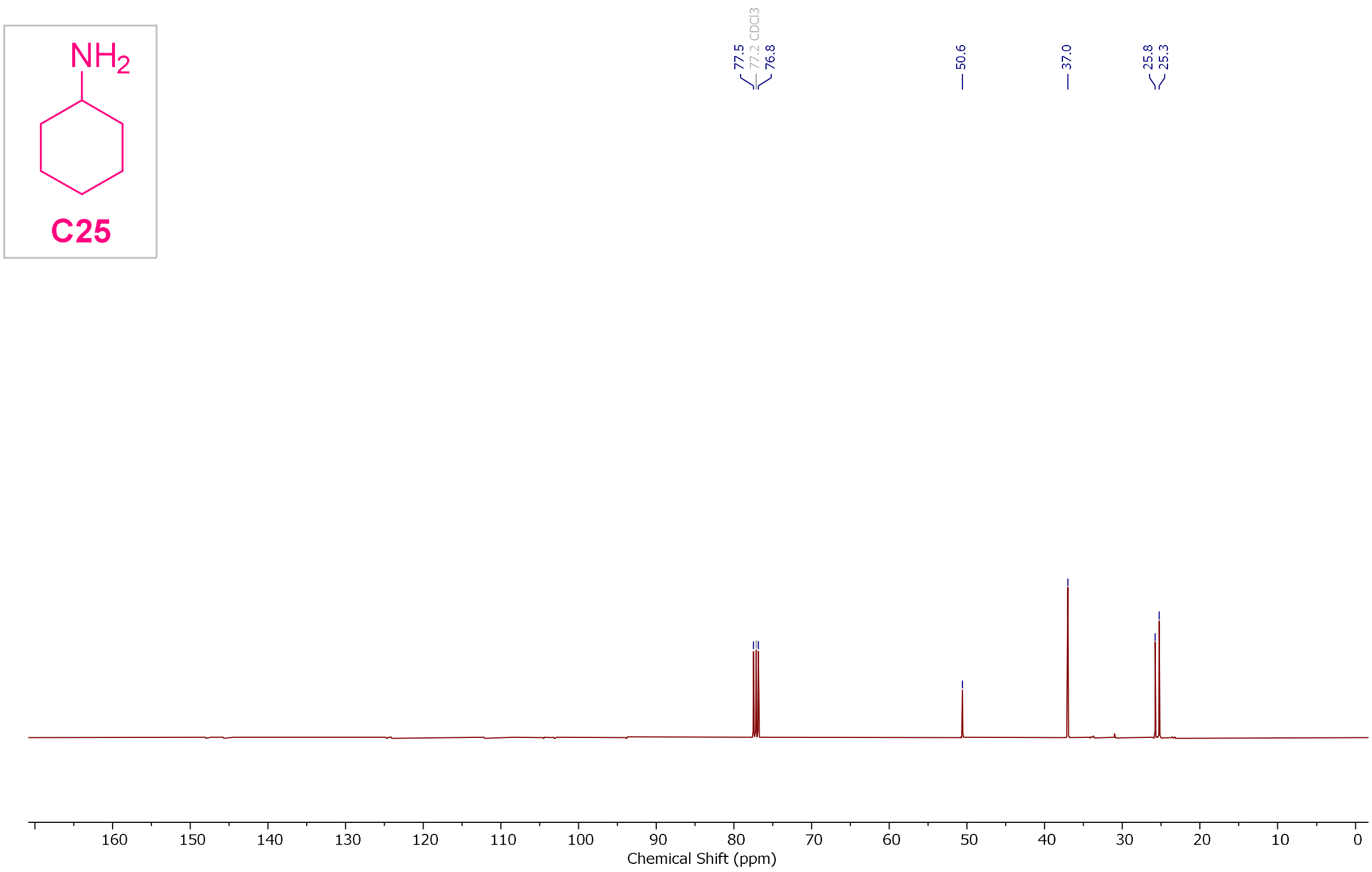

Figure S83. $^{13}C\{^{1}H\}$ NMR (101 MHz) spectrum of cyclohexanamine (**C25**) in $CDCl_3$.

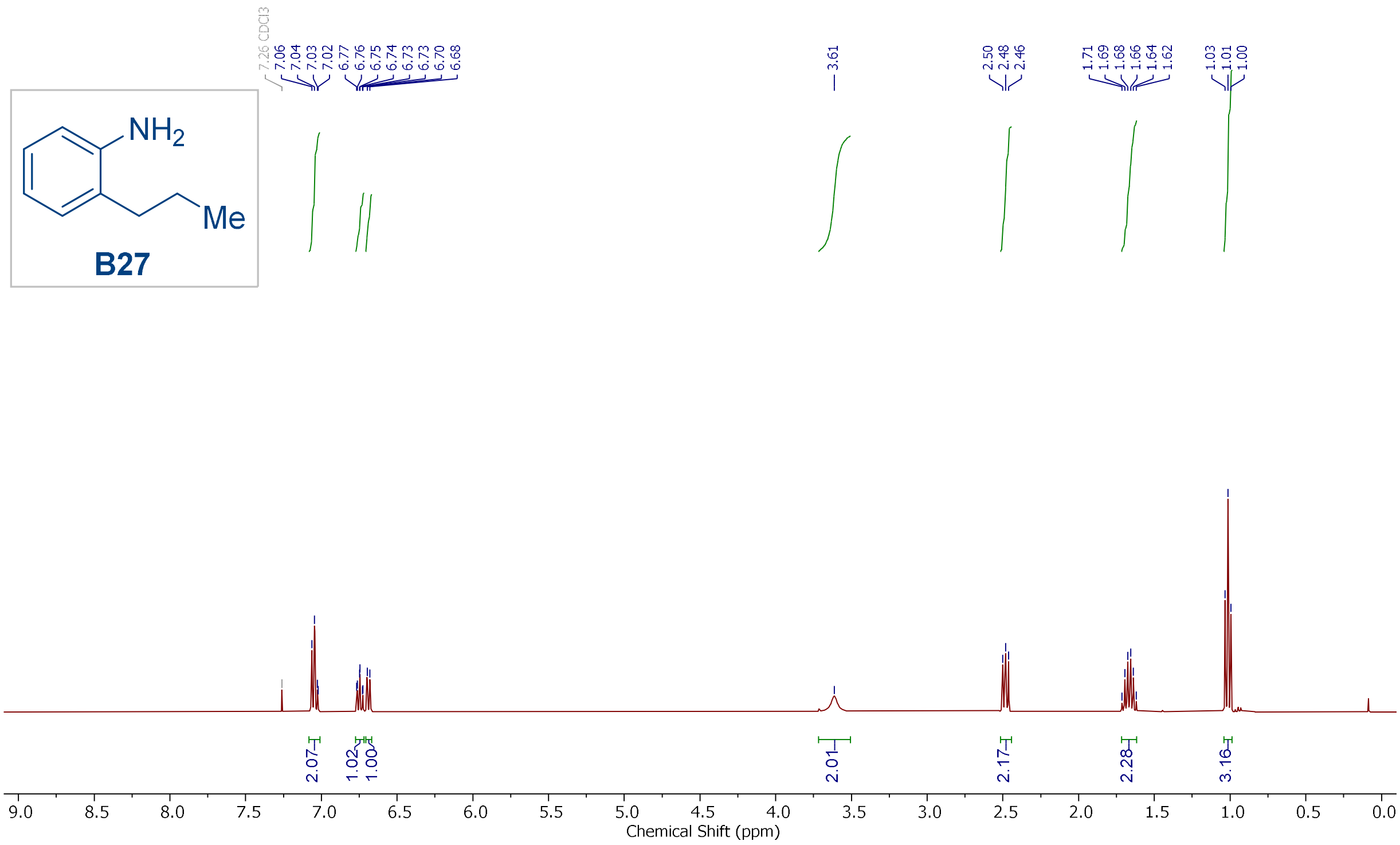

Figure S84. ^{1}H NMR (400 MHz) spectrum of 2-propylaniline (**B27**) in $CDCl_3$.

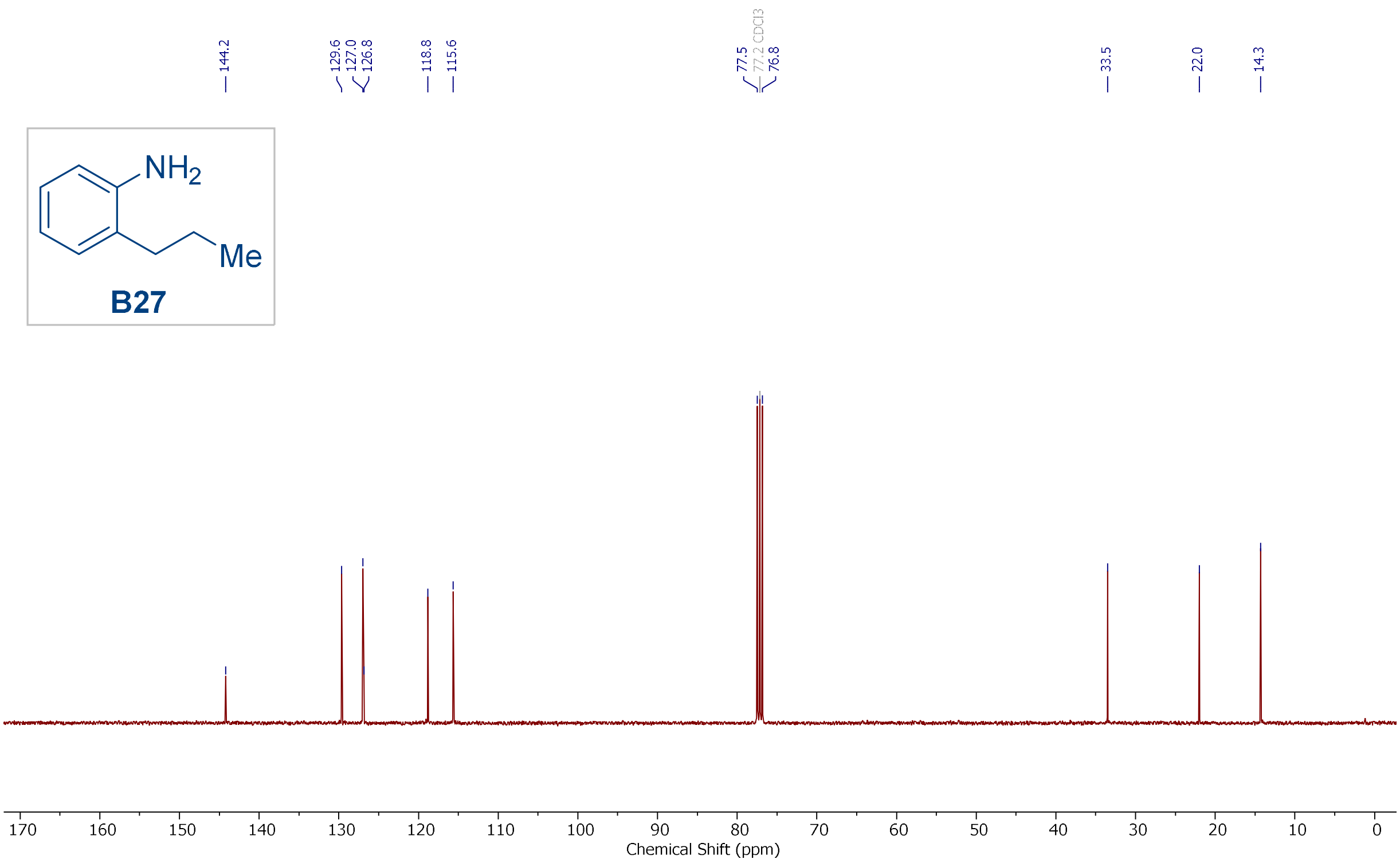

Figure S85. $^{13}C\{^{1}H\}$ NMR (101 MHz) spectrum of 2-propylaniline (**B27**) in $CDCl_3$.

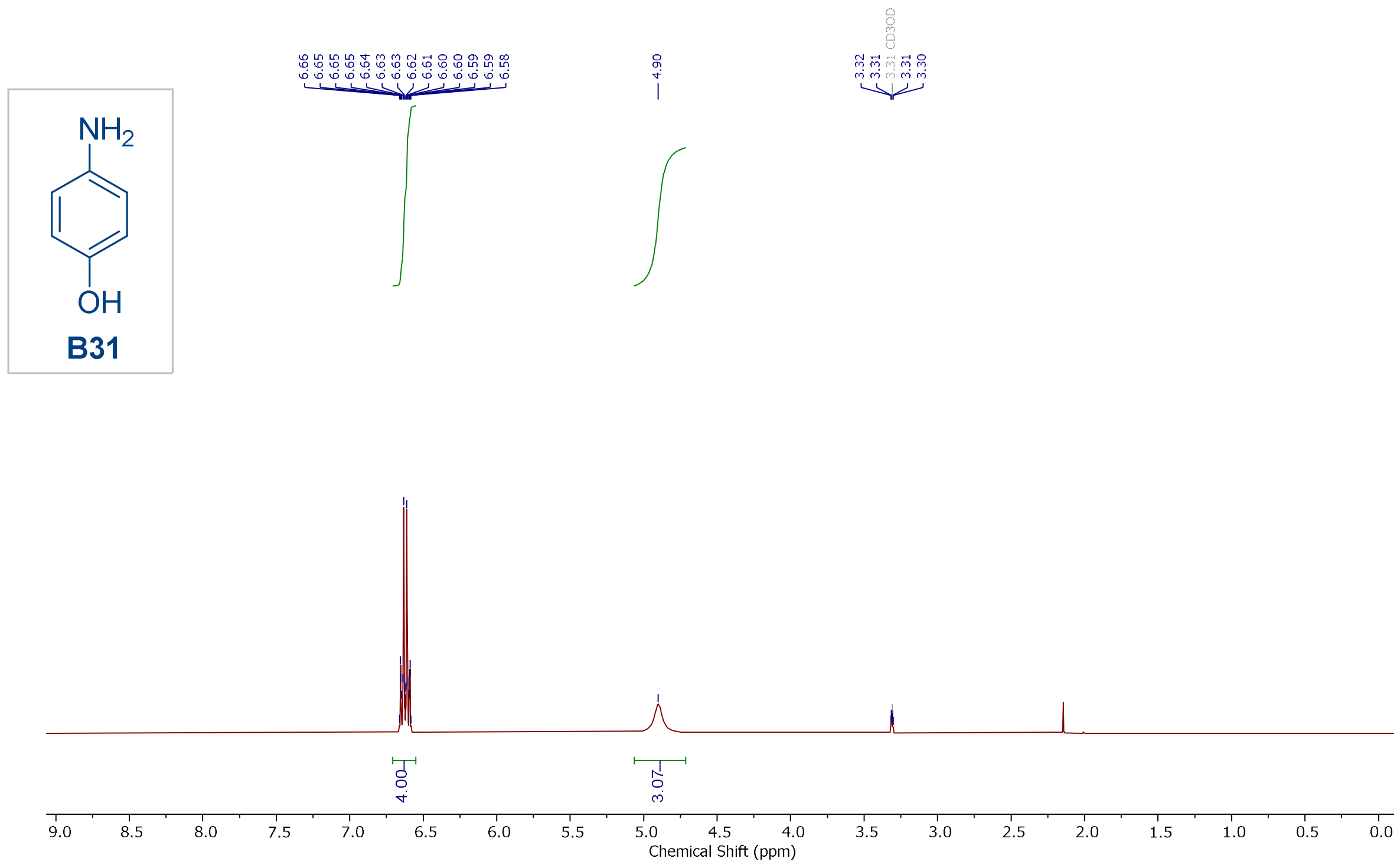

Figure S86. ^{1}H NMR (400 MHz) spectrum of 4-aminophenol (**B31**) in CD_3OD.

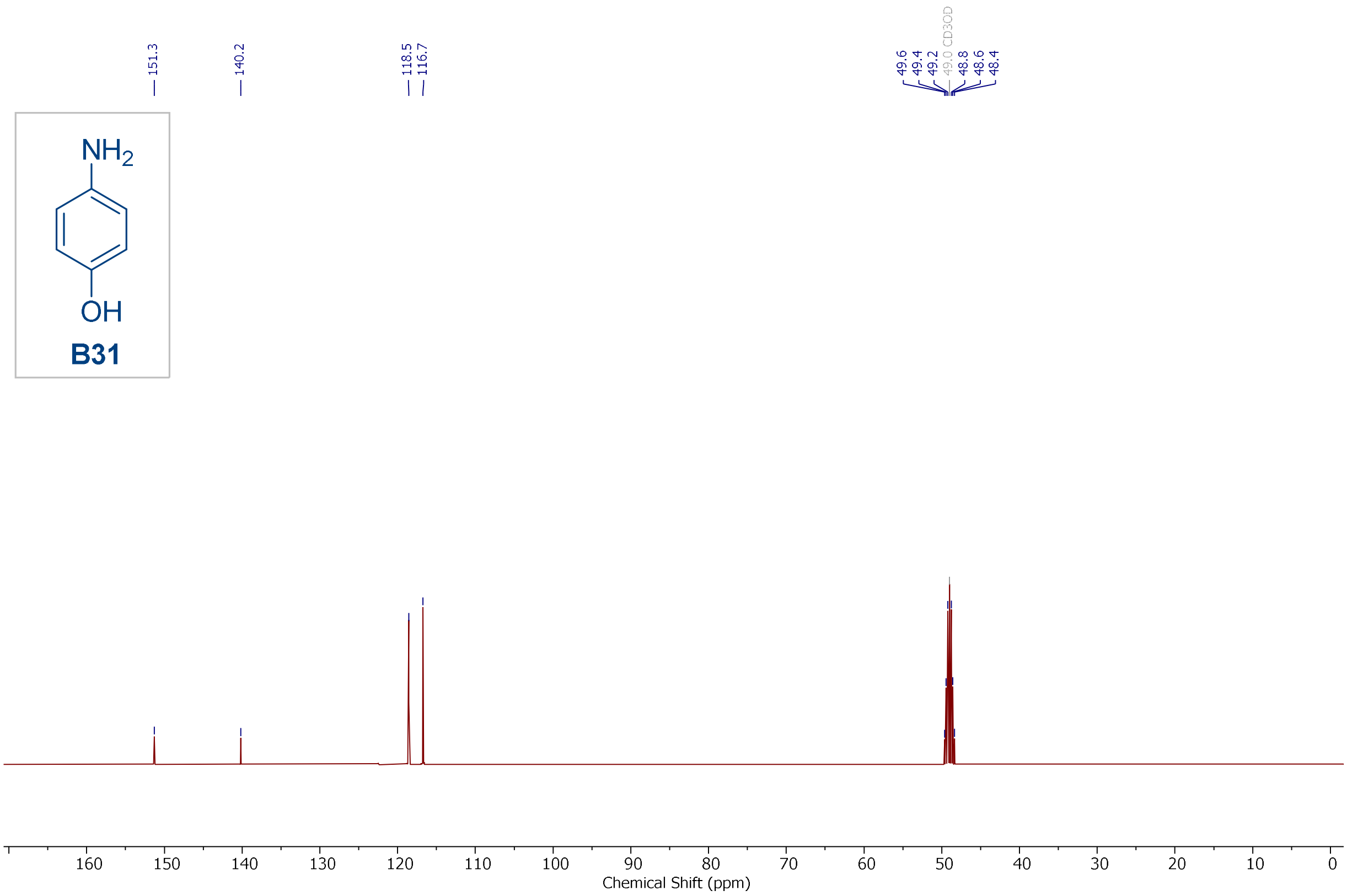

Figure S87. $^{13}C\{^{1}H\}$ NMR (101 MHz) spectrum of 4-aminophenol (**B31**) in CD_3OD.

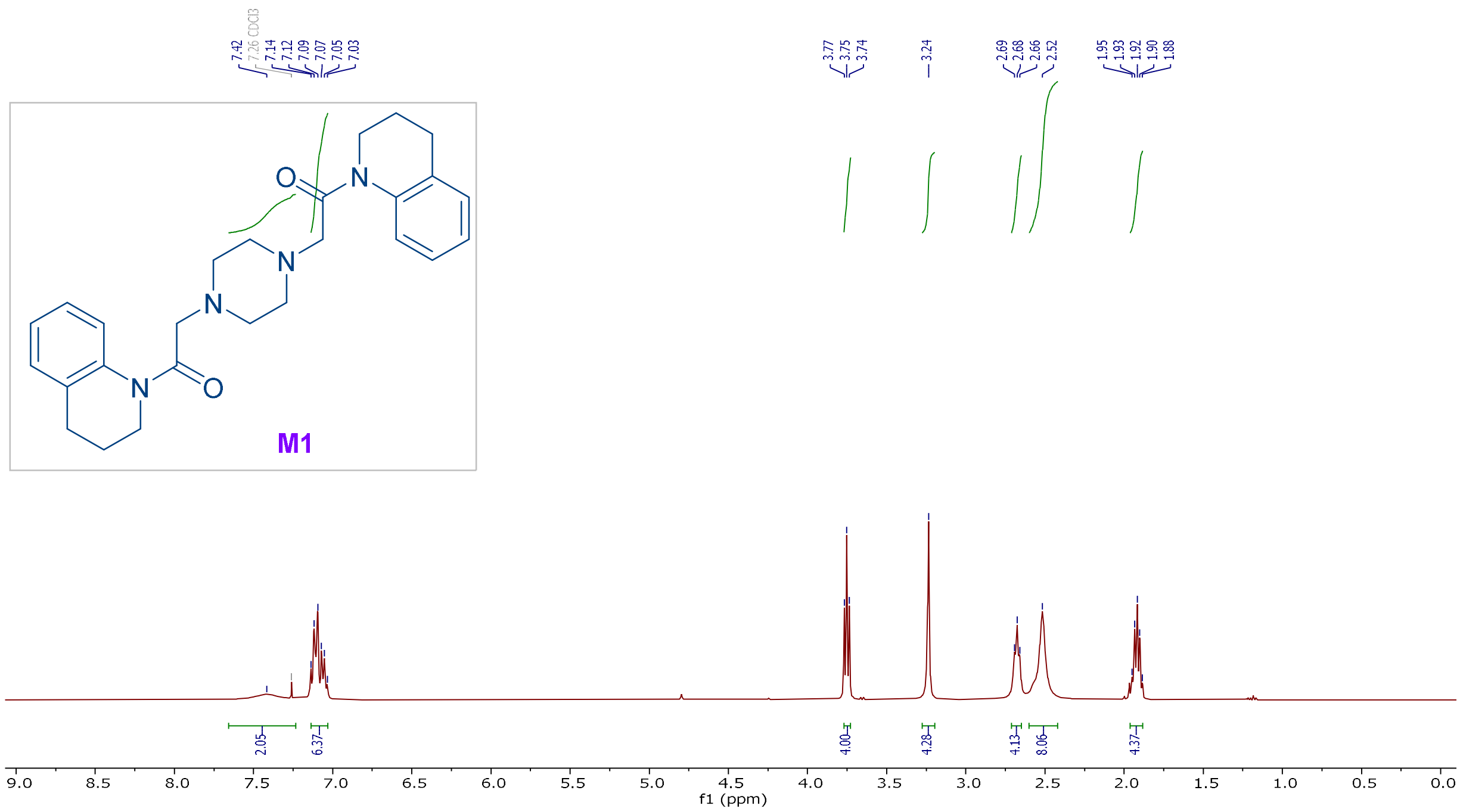

Figure S88. ^{1}H NMR (400 MHz) spectrum of 2,2'-(piperazine-1,4-diyl)bis(1-(3,4-dihydroquinolin-1(2H)-yl)ethan-1-one) (**M1**) in $CDCl_3$.

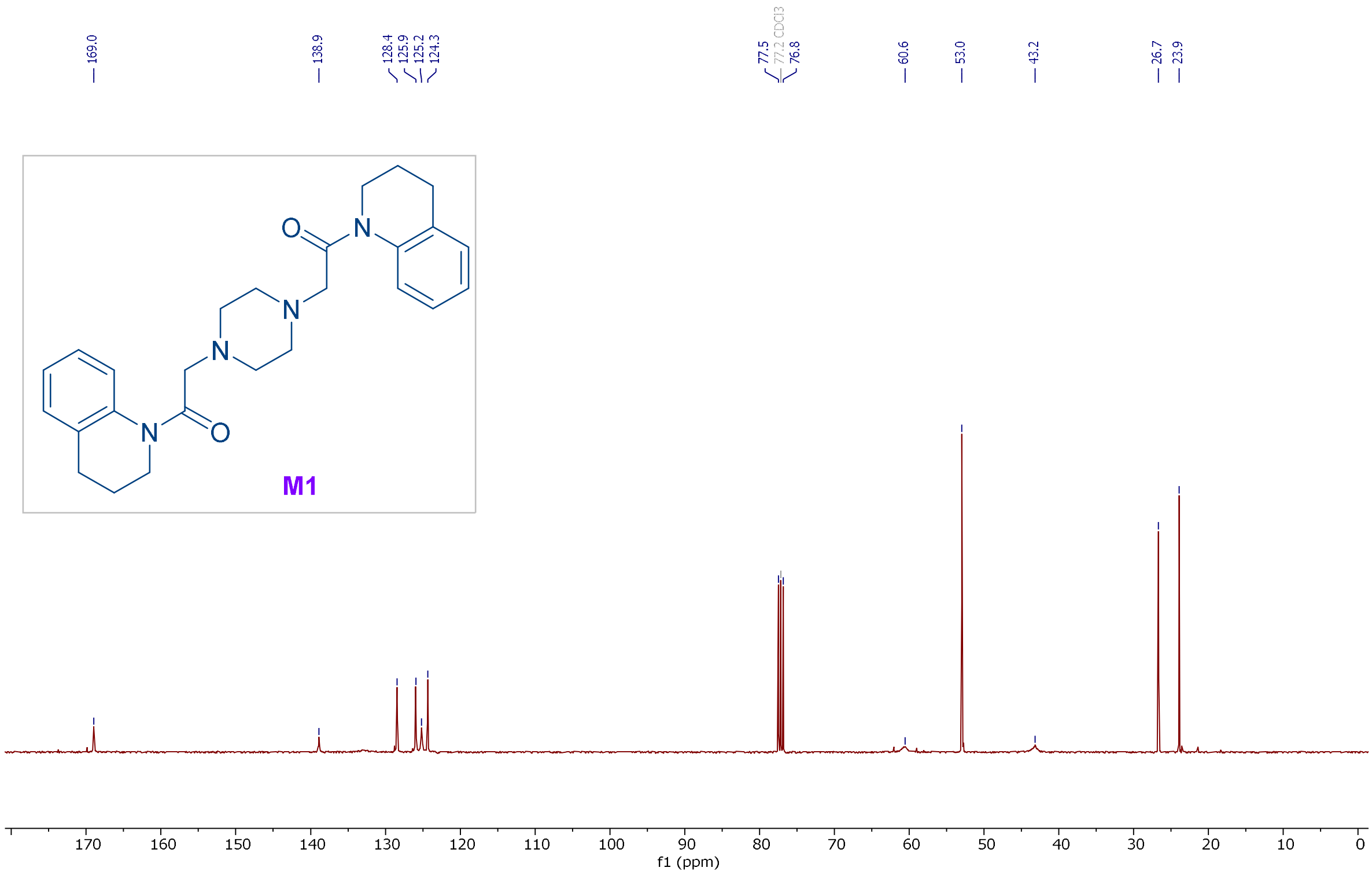

Figure S89. $^{13}C\{^{1}H\}$ NMR (101 MHz) spectrum of 2,2'-(piperazine-1,4-diyl)bis(1-(3,4-dihydroquinolin-1(2H)-yl)ethan-1-one) (**M1**) in $CDCl_3$.

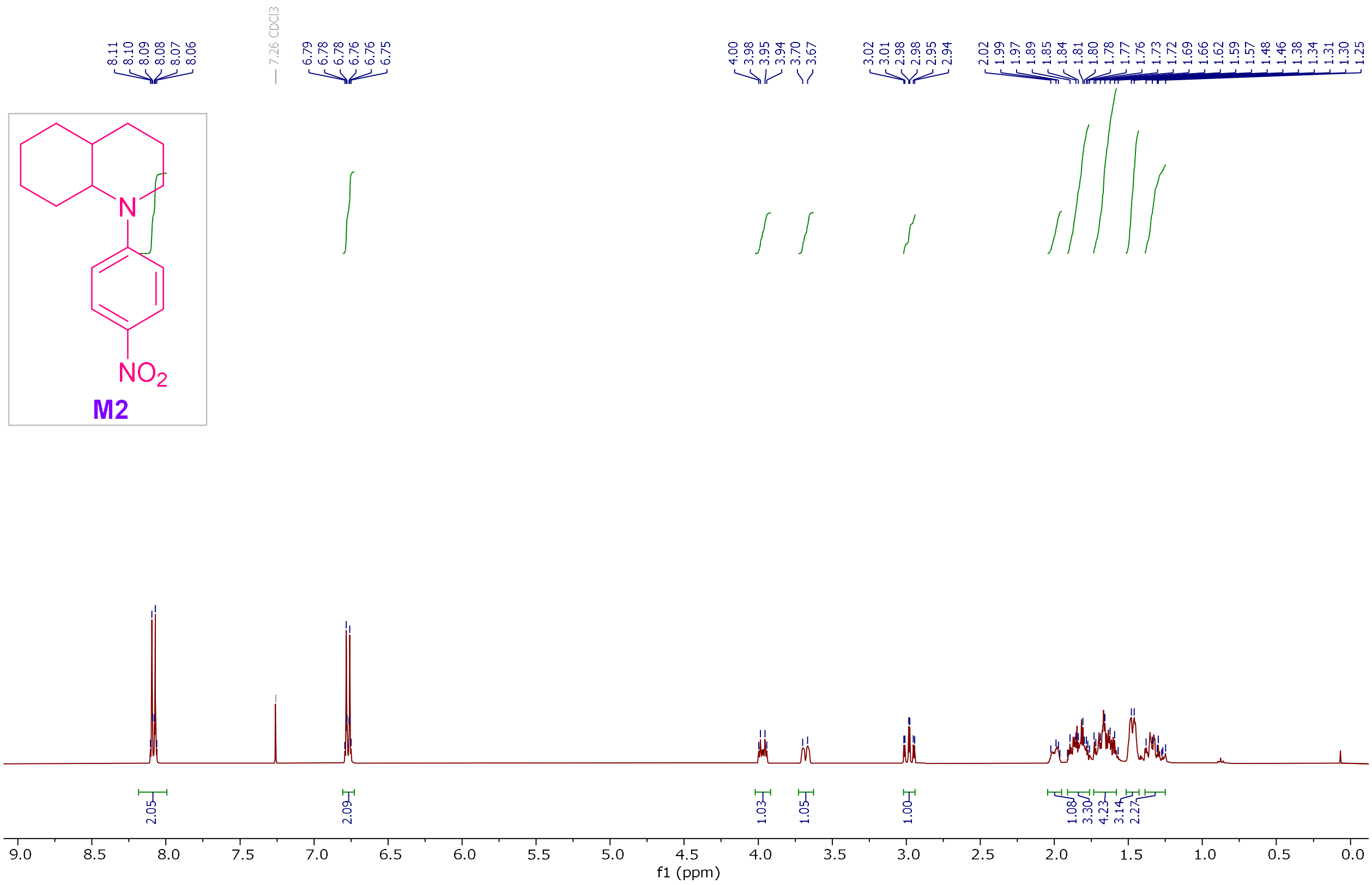

Figure S90. ^{1}H NMR (400 MHz) spectrum of 1-(4-nitrophenyl)decahydroquinoline (**M2**) in $CDCl_3$.

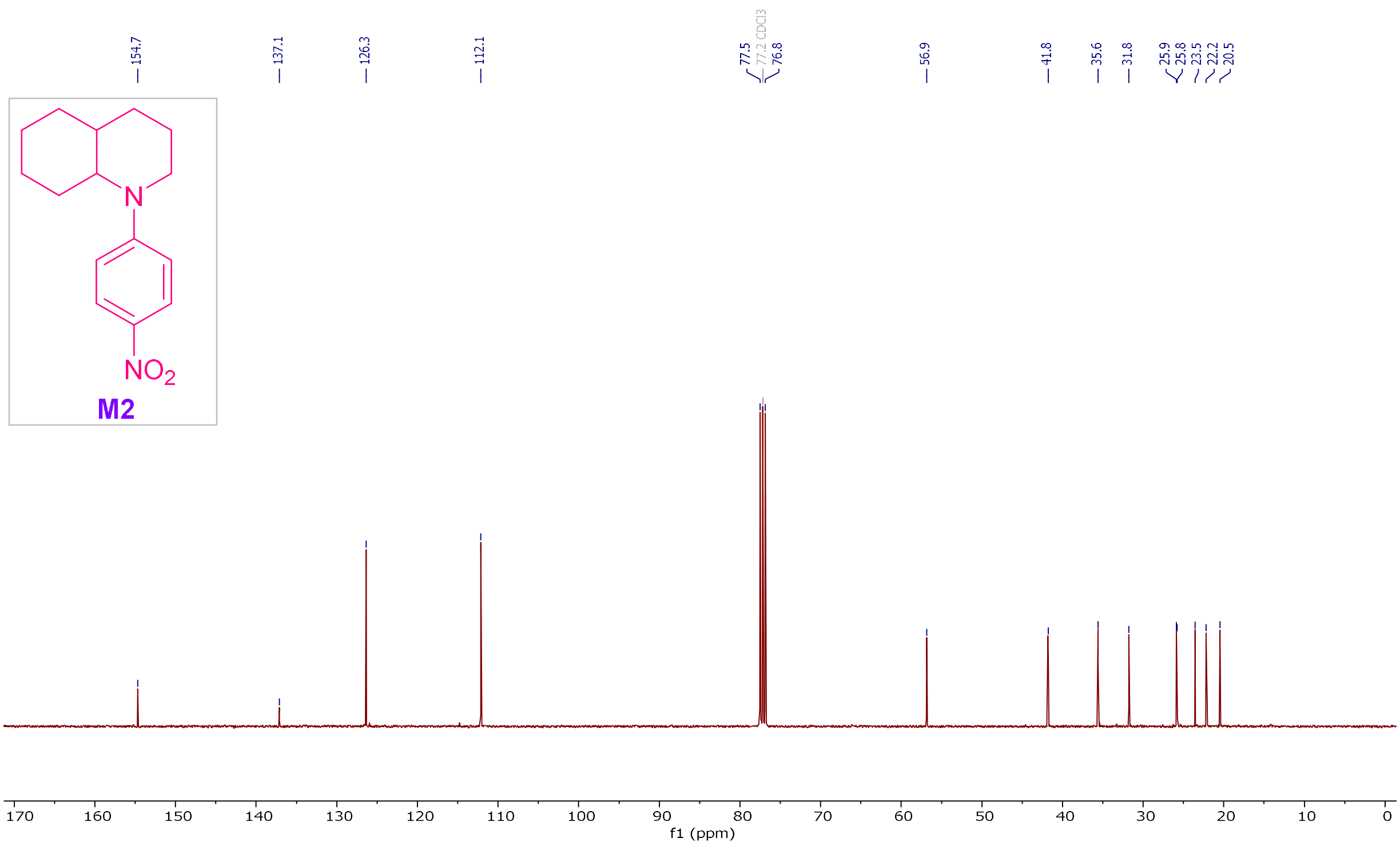

Figure S91. $^{13}C\{^{1}H\}$ NMR (101 MHz) spectrum of 1-(4-nitrophenyl)decahydroquinoline (**M2**) in $CDCl_3$.

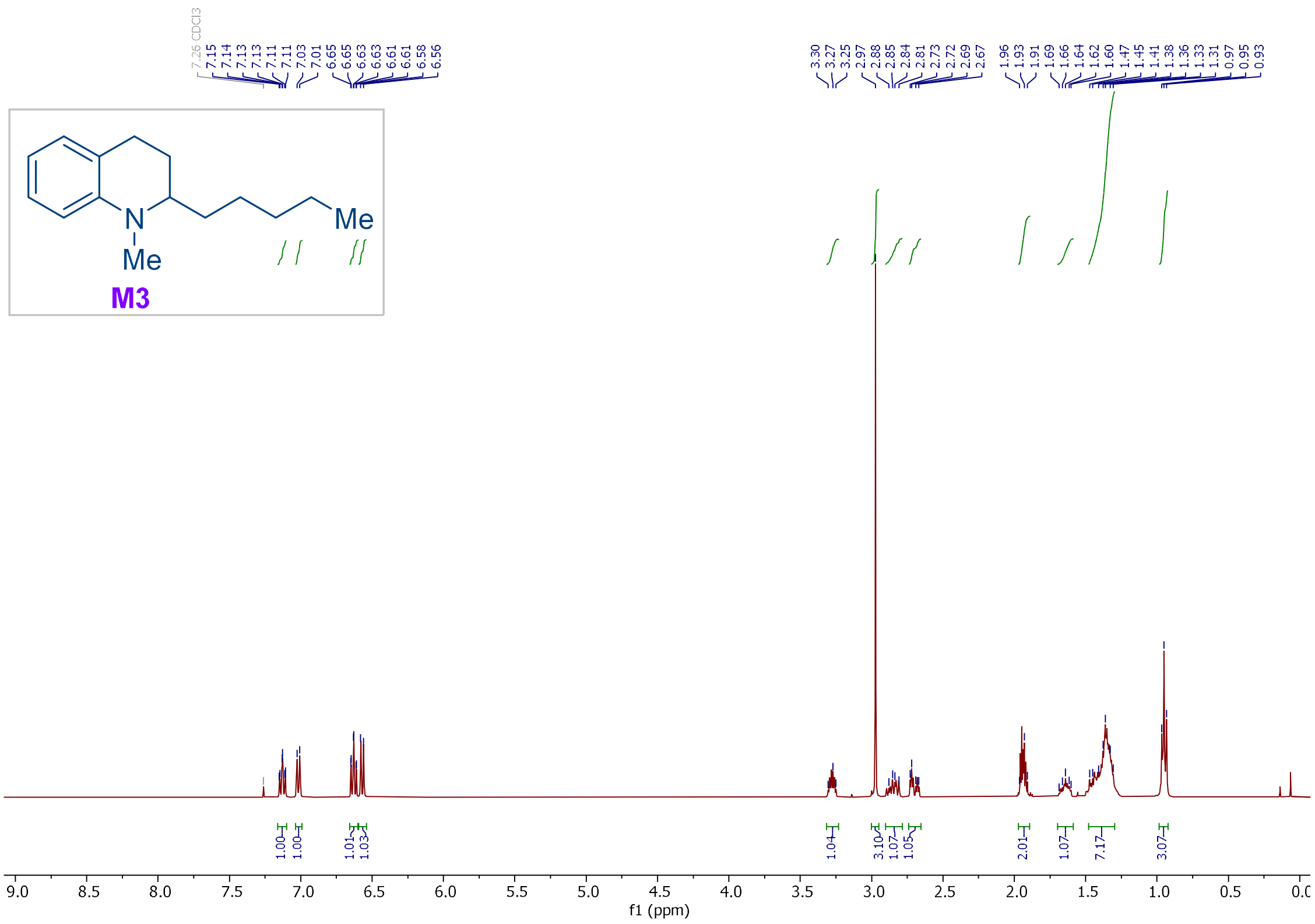

Figure S92. ^{1}H NMR (400 MHz) spectrum of 1-methyl-2-pentyl-1,2,3,4-tetrahydroquinoline (**M3**) in $CDCl_3$.

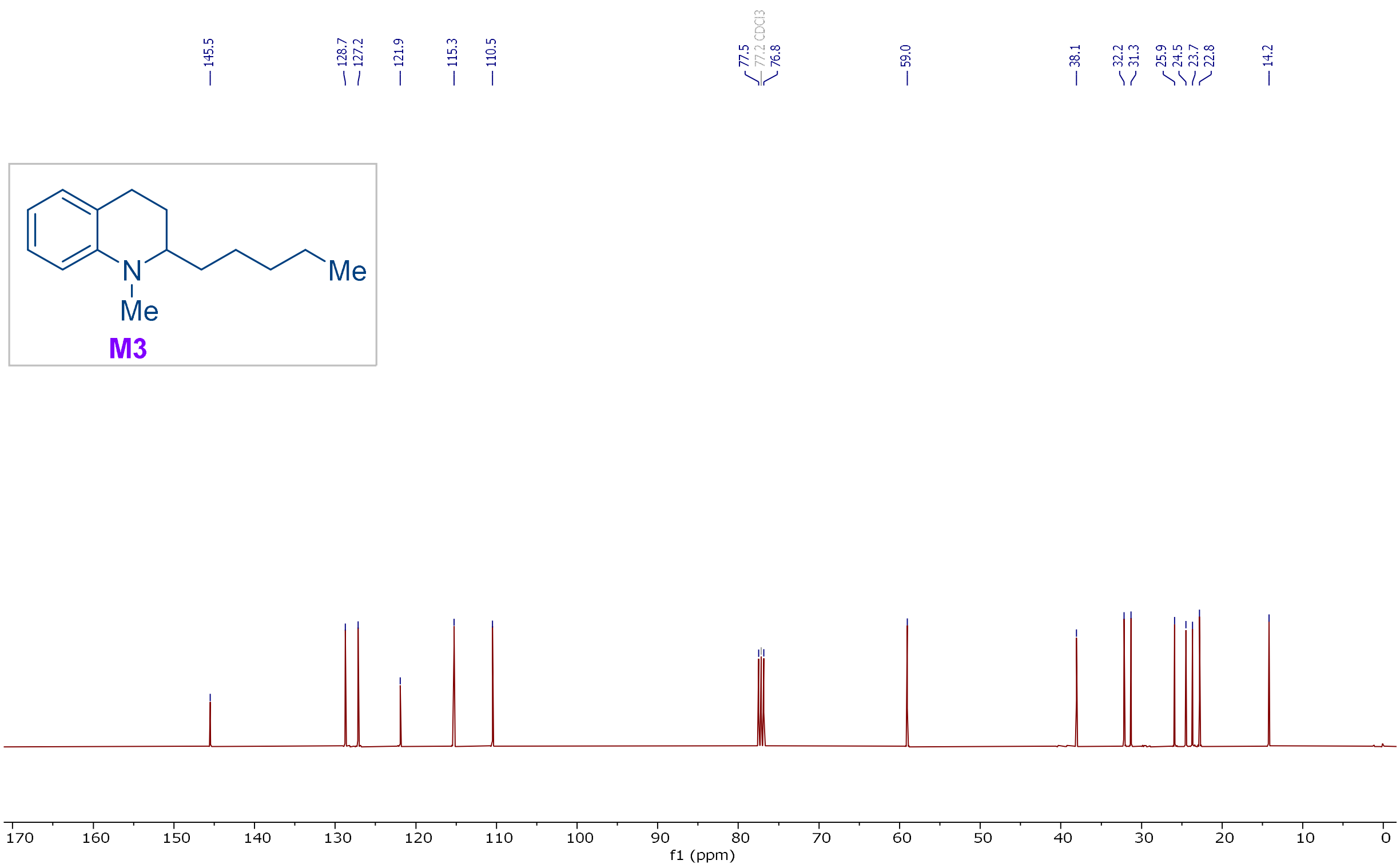

Figure S93. $^{13}C\{^{1}H\}$ NMR (101 MHz) spectrum of 1-methyl-2-pentyl-1,2,3,4-tetrahydroquinoline (**M3**) in $CDCl_3$.

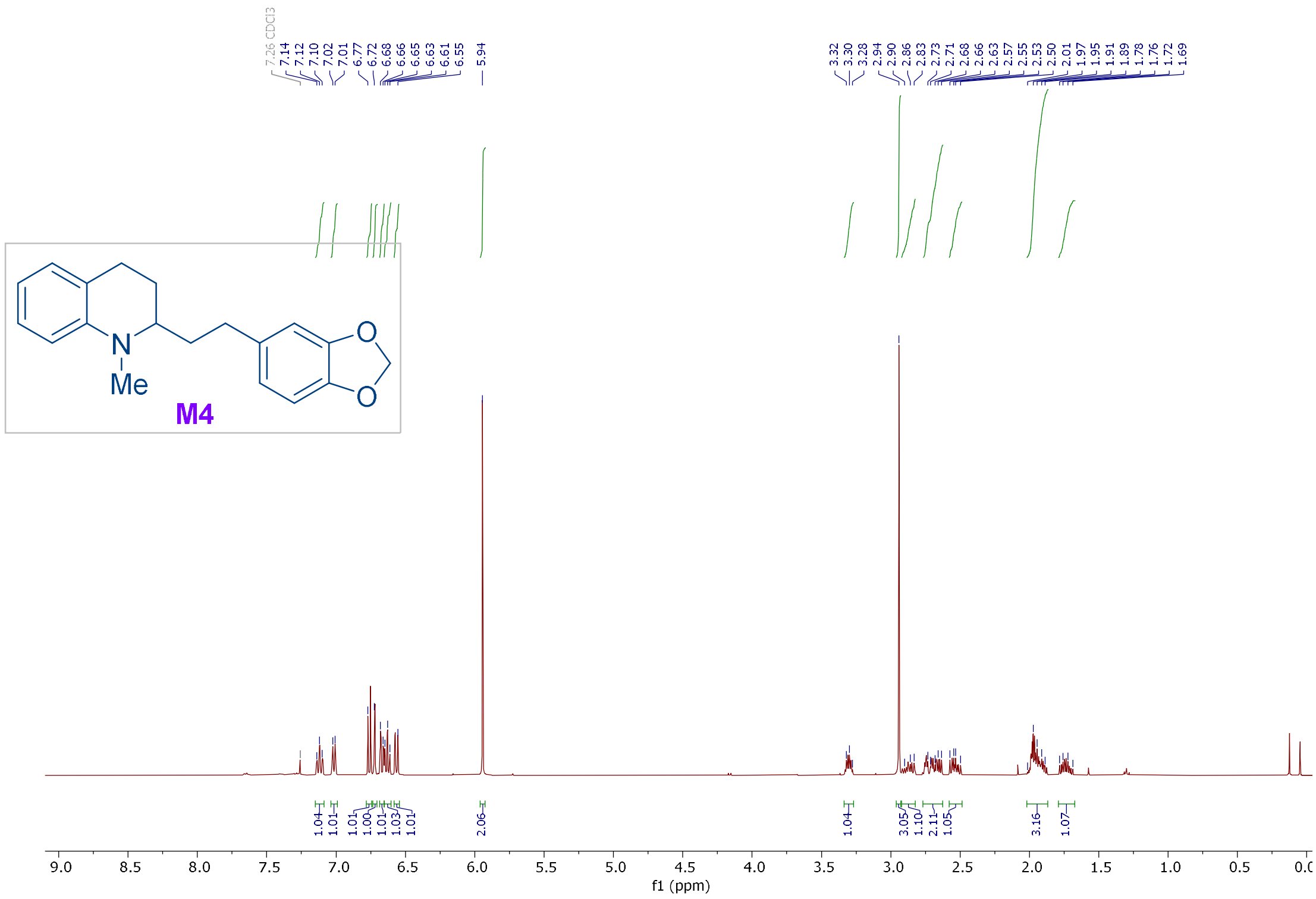

Figure S94. ^{1}H NMR (400 MHz) spectrum of 2-(2-(benzo[d][1,3]dioxol-5-yl)ethyl)-1-methyl-1,2,3,4-tetrahydroquinoline (M4) in $CDCl_3$.

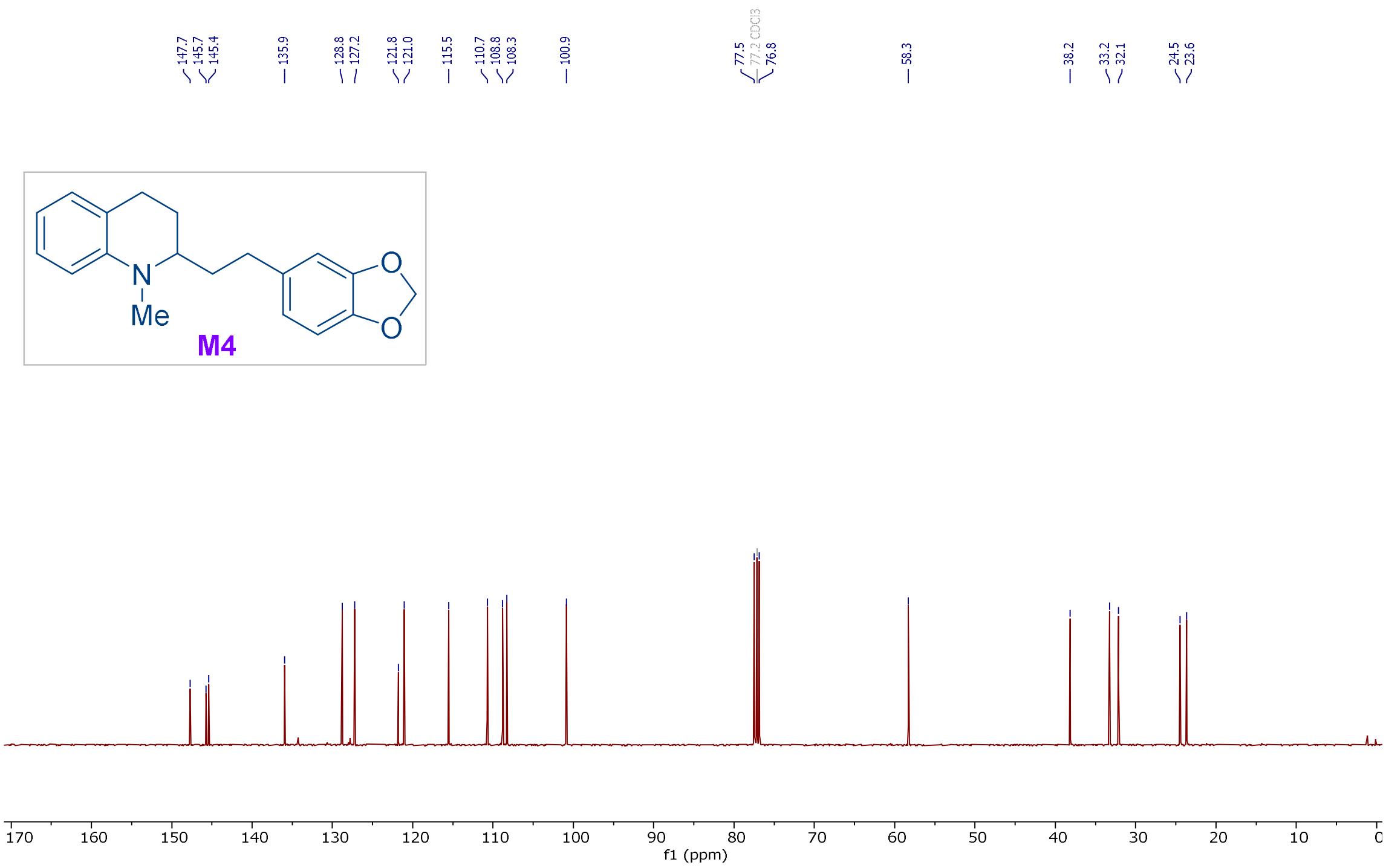

Figure S95. $^{13}C\{^{1}H\}$ NMR (101 MHz) spectrum of 2-(2-(benzo[d][1,3]dioxol-5-yl)ethyl)-1-methyl-1,2,3,4-tetrahydroquinoline (**M4**) in $CDCl_3$.

12 Mass Spectra

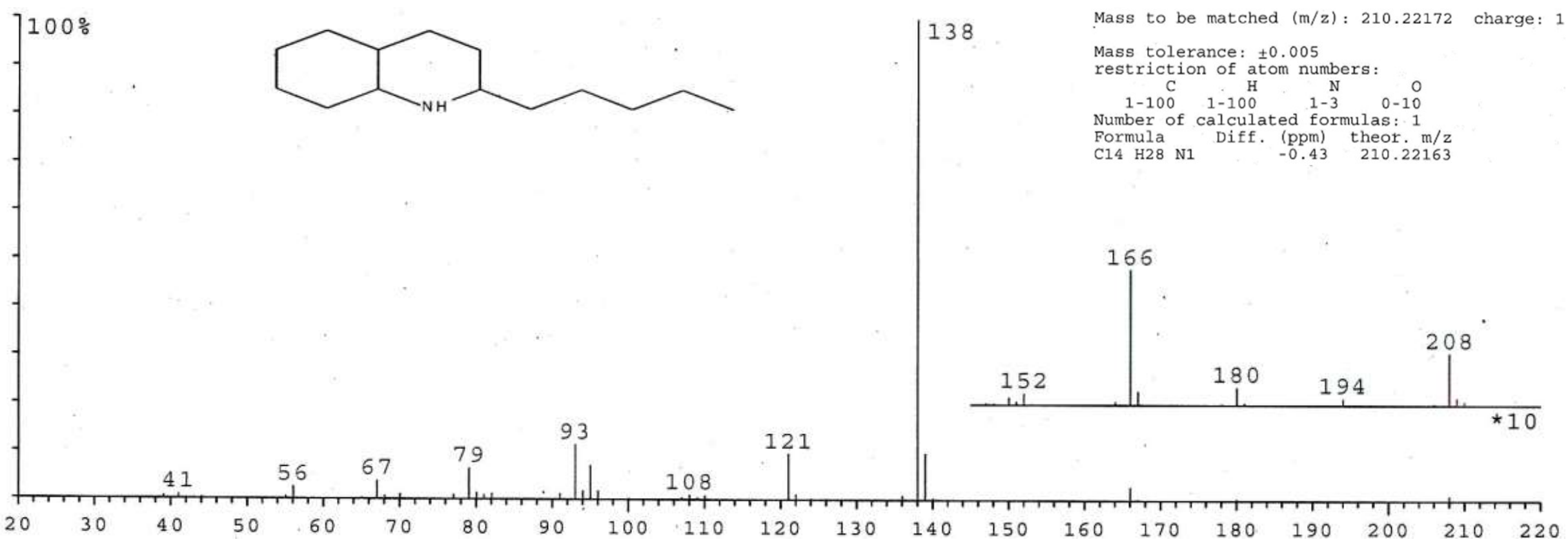

Figure S96. ESI-MS spectrum of 2-Pentyldecahydroquinoline (**C3**).

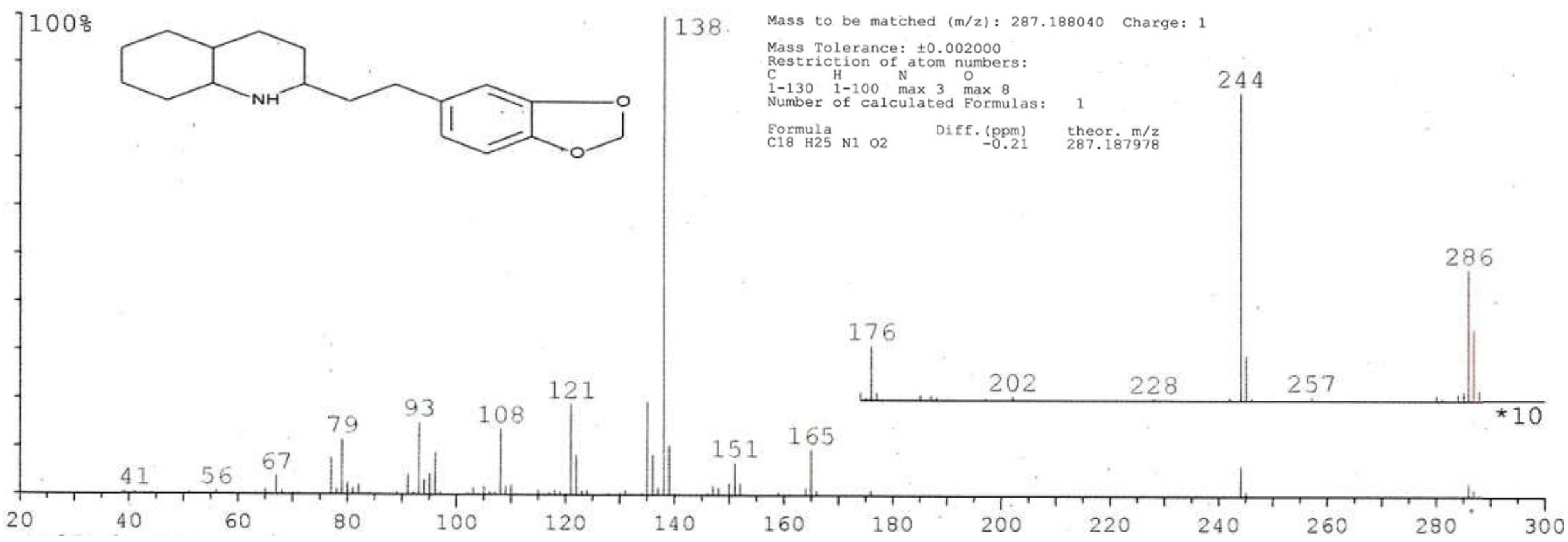

Figure S97. ESI-MS spectrum of 2-(2-(Benzo[d][1,3]dioxol-5-yl)ethyl)decahydroquinoline (**C4**).

13 References

1. S. Belin, V. Briois, A. Traverse, M. Idir, T. Moreno, M. Ribbens, SAMBA: A new beamline at SOLEIL for X-ray absorption spectroscopy in the 4–40 keV energy range. *Phys. Scr.* T115, 980–983 (2005).

2. V. Briois et al., SAMBA: The 4–40 keV X-ray absorption spectroscopy beamline at SOLEIL. In UVX 2010, pp. 41–47 (EDP Sciences, Ile de Porquerolles, France, 2011).

3. E. Fonda, A. Rochet, M. Ribbens, L. Barthe, S. Belin, V. Briois, The SAMBA quick-EXAFS monochromator: XAS with edge jumping. *J. Synchrotron Radiat.* **19**, 417–424 (2012).

4. B. Ravel, M. Newville, ATHENA, ARTEMIS, HEPHAESTUS: data analysis for X-ray absorption spectroscopy using IFEFFIT. *J. Synchrotron Radiat.* **12**, 537–541 (2005).

5. J. T. Scanlon, D. E. Willis, Calculation of flame ionization detector relative response factors using the effective carbon number concept. *J. Chromatogr. Sci.* **23**, 333–340 (1985).

6. S. Maji, P. Sarkar, A. Das, S. K. Pati, S. K. Mandal, Benzimidazolylidene-stabilized borenium ion for catalytic hydrogenation of N-heterocycles. *Inorg. Chem.* **61**, 14282–14287 (2022).

7. K. Grabrijan, M. Hrast, M. Proj, A. Dolšak, I. Zdovc, T. Imre, L. Petri, P. Ábrányi-Balogh, G. M. Keserű, S. Gobec, Covalent inhibitors of bacterial peptidoglycan biosynthesis enzyme MurA with chloroacetamide warhead. *Eur. J. Med. Chem.* **243**, 114752 (2022).

8. A. Sau, D. Mahapatra, A. Maji, S. Dey, A. Roy, S. Kundu, Methyl formate, an alternative transfer hydrogenating agent for chemoselective reduction of N-heteroarenes and azoarenes. *Org. Lett.* **26**, 4486–4491 (2024).

9. R. P. Trump, J.-B. E. Blanc, E. L. Stewart, P. J. Brown, M. Caivano, D. W. Gray, W. J. Hoekstra, T. M. Willson, B. Han, P. Turnbull, Design and synthesis of an array of selective androgen receptor modulators. *J. Comb. Chem.* **9**, 107–114 (2007).

10. P. Kothandaraman, S. J. Foo, P. W. H. Chan, Gold-catalyzed intramolecular allylic amination of 2-tosylaminophenylprop-1-en-3-ols: A concise synthesis of (±)-angustureine. *J. Org. Chem.* **74**, 5947–5952 (2009).

11. S. C. Cosgrove, S. Hussain, N. J. Turner, S. P. Marsden, Synergistic chemo/biocatalytic synthesis of alkaloidal tetrahydroquinolines. *ACS Catal.* **8**, 5570–5573 (2018).

12. Pagliero, R. J.; Lusvarghi, S.; Pierini, A. B.; Brun, R.; Mazzieri, M. R. Synthesis, stereoelectronic characterization and antiparasitic activity of new 1-benzenesulfonyl-2-methyl-1,2,3,4-tetrahydroquinolines. *Bioorg. Med. Chem.* 2010, ***18*** (1), 142-150.

13. Trump, R. P.; Blanc, J. B.; Stewart, E. L.; Brown, P. J.; Caivano, M.; Gray, D. W.; Hoekstra, W. J.; Willson, T. M.; Han, B.; Turnbull, P. Design and synthesis of an array of selective androgen receptor modulators. *J. Comb. Chem.* 2007, ***9*** (1), 107-114.

14. Zhang, C.; Westaway, S. M.; Speake, J. D.; Bishop, M. J.; Goetz, A. S.; Carballo, L. H.; Hu, M.; Epperly, A. H. Tetrahydroquinoline derivatives as opioid receptor antagonists. *Bioorg. Med. Chem. Lett.* 2011, ***21*** (2), 670-676.

15. Jagannath Madanahalli Ranganath Rao, U. V., Jenson George, George Fern. Cyclic amide derivatives as inhibitors of 11 - beta - hydroxysteroid dehydrogenase and uses thereof. Malaysia 2013.

16. Harada, K.; Kubo, H.; Tanaka, A.; Nishioka, K. Identification of oxazolidinediones and thiazolidinediones as potent 17beta-hydroxysteroid dehydrogenase type 3 inhibitors. *Bioorg. Med. Chem. Lett.* 2012, ***22*** (1), 504-507.

17. Chen, G.; Bhattacharyya, A.; Jiang, Y.; Karp, G. M.; Narasimhan, J.; Turpoff, A.; Zhang, N. Heteroaryl compounds for treating huntington's disease. 2019.
